# Mechanistic Insights into the Formation of 1-Alkylidene/Arylidene-1,2,4-triazolinium Salts: A Combined NMR/Density Functional Theory Approach

**DOI:** 10.1021/acs.joc.1c02327

**Published:** 2022-01-03

**Authors:** Johann Pann, Kevin Erharter, Daniel Langerreiter, Gabriel Partl, Thomas Müller, Herwig Schottenberger, Michael Hummel, Thomas S. Hofer, Christoph Kreutz, Lukas Fliri

**Affiliations:** † Institute of General, Inorganic Chemistry and Theoretical Chemistry, Faculty of Chemistry and Pharmacy, University of Innsbruck, Innrain 80-82, 6020 Innsbruck, Austria; ‡ Institute of Organic Chemistry and Center for Molecular Bioscience Innsbruck (CMBI), Faculty of Chemistry and Pharmacy, University of Innsbruck, Innrain 80-82, 6020 Innsbruck, Austria; § Department of Bioproducts and Biosystems, Aalto University, P.O. Box 16300, 0076 Aalto, Finland

## Abstract

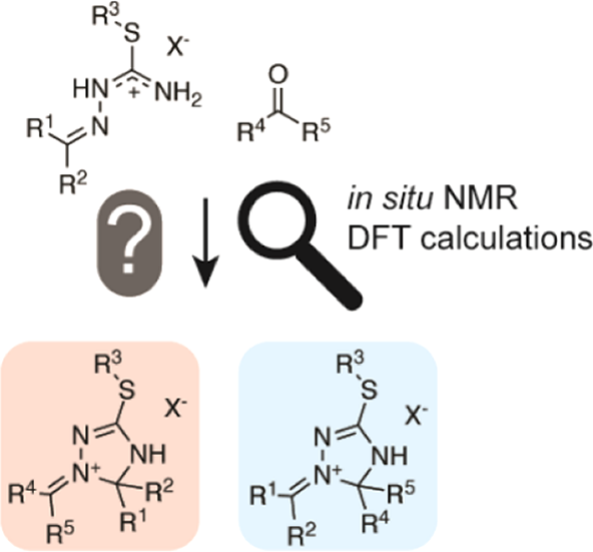

In a recent report on the synthetic approach to the novel substance class of 1-alkylidene/arylidene-1,2,4-triazolinium salts, a reaction mechanism suggesting a regioselective outcome was proposed. This hypothesis was tested via a combined NMR and density functional theory (DFT) approach. To this end, three experiments with ^13^C-labeled carbonyl reactants were monitored *in situ* by solution-state NMR. In one experiment, an intermediate as described in the former mechanistic proposal was observed. However, incorporation of ^13^C isotope labels into multiple sites of the heterocycle could not be reconciled with the “regioselective mechanism”. It was found that an unproductive reaction pathway can lead to ^13^C scrambling, along with metathetical carbonyl exchange. According to DFT calculations, the concurring reaction pathways are connected *via* a thermodynamically controlled cyclic 1,3-oxazetidine intermediate. The obtained insights were applied in a synthetic study including aliphatic ketones and para-substituted benzaldehydes. The mechanistic peculiarities set the potential synthetic scope of the novel reaction type.

## Introduction

**Scheme 1:**
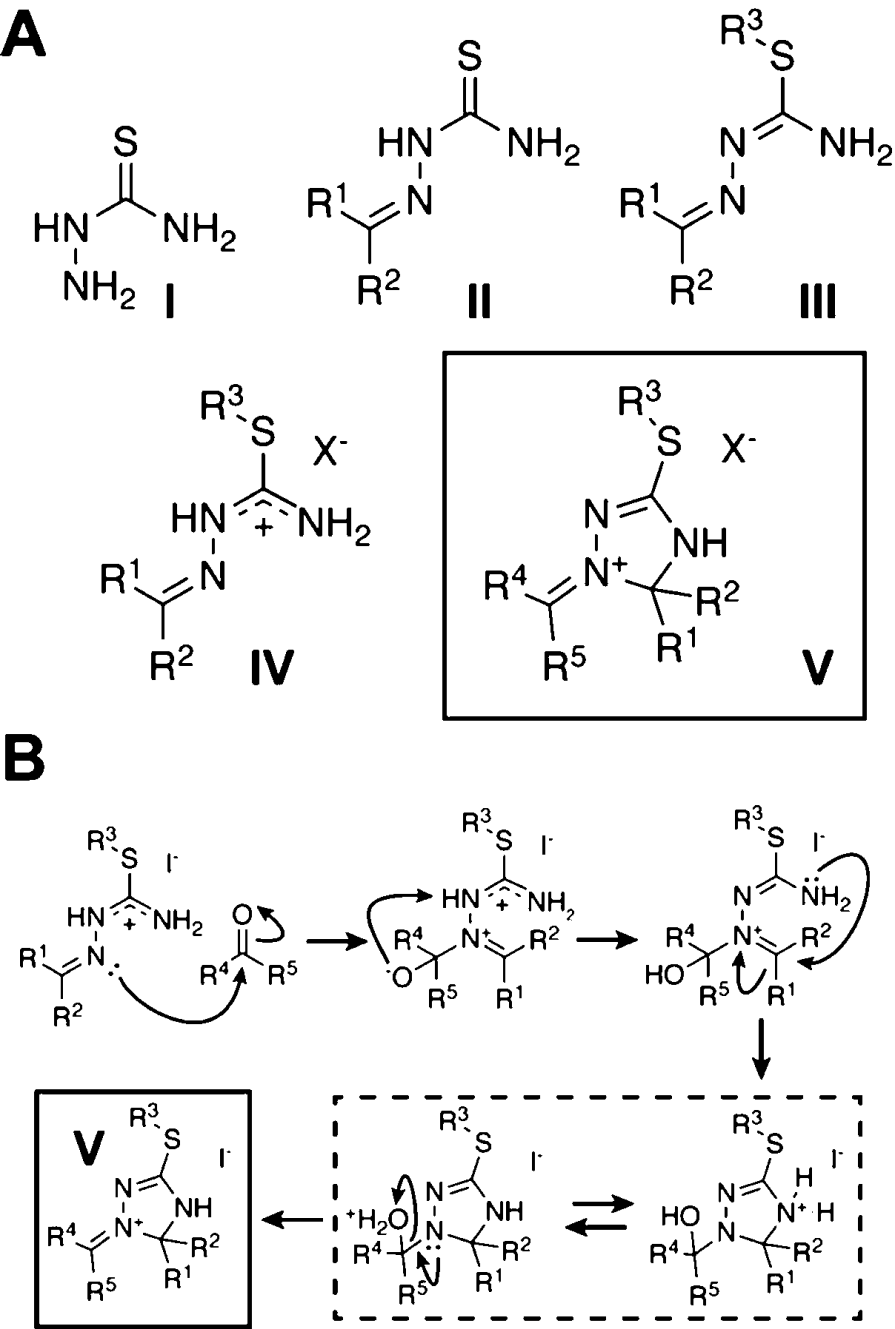
Substance Classes and Reaction Mechanism in the Focus of This Work; (A) Overview of the Molecular Structures of Thiosemicarbazide (**I**), Thiosemicarbazones (**II**), Isothiosemicarbazones (**III**), and Isothiosemicarbazonium Salts (**IV**) and of 1-Alkylidene/Arylidene-1,2,4-triazolinium Salts (**V**, Highlighted with a Box), Which Were Studied in This Work; (B) Initially Proposed Reaction Mechanism for the Formation of **V**; the Reaction Mechanism Was Tested *via* NMR Spectroscopy and DFT Calculations; and R^1^–R^5^ = Alkyl, Aryl, or H

Thiosemicarbazide (**I**) and the closely related thiosemicarbazones (**II**) and isothiosemicarbazones (**III**) have been synthetically exploited over decades (Scheme 1A). After early applications in the qualitative analysis of aldehydes and ketones,^1^ the applicative focus later shifted toward their use as ligands in coordination chemistry.^2−4^ Compounds **I**–**III** were also soon identified as versatile starting materials in organic synthesis offering four distinct nucleophilic centers and thus allowing cascade reactions to give various heterocyclic compounds.^5−8^ Even after more than 100 years of research,^9^ unprecedented reaction outcomes of these starting materials have still been recently reported.^10^ In contrast, isothiosemicarbazonium salts (**IV**; Scheme 1A)—key intermediates for the preparation of the intensively studied isothiosemicarbazones (**III**)^11−16^—were thus far not extensively investigated for their use in cyclization reactions. Merely, their ring—chain tautomerism in solution was studied *via* NMR spectroscopy.^17,18^

In a preliminary communication, one of our research groups recently reported that these isothiosemicarbazonium salts (**IV**) show a distinct reactivity toward aldehydes and ketones, when subjected to slightly acidic conditions.^19^ Thus, the access to the substance class of 1-alkylidene/arylidene-1,2,4-triazolinium salts (**V**) was established, which hitherto was only obtained as a side product.^20^ Notably, the respective structure motif was unambiguously confirmed by X-ray crystallography.^19^ Although the combination of a highly reactive iminium functionality embedded in a somewhat unstable 1,2,4-triazoline ring might suggest a rapid refragmentation of the heterocycle, the six isolated substances proved to be robust and easy to handle.^21,22^ Even after more than one year of storage in closed, light-protected containers under ambient conditions, no signs of degradation were observable for the iodide salts.^19^ We were tempted to pursue further research in this area, not only because of the unprecedented structure with respect to heterocyclic chemistry. Potential applications in pharmaceutical chemistry and in materials science are known for other 1,2,4-triazoline derivatives^23,24^ and especially for their oxidized 1,2,4-triazole congeners.^25−27^ To establish a starting point for future synthetic explorations, the initially proposed reaction mechanism (Scheme 1B) for the formation of compounds of type **V** was tested by a combined NMR/density functional theory (DFT) approach. Furthermore, we were interested in a better understanding of the observed side reaction of educts **IV** leading to a metathetical carbonyl exchange, which was recently termed as transazination or transalkylidation.^19,28^ The mechanistic details of the formation of **V** were probed by *in situ* NMR spectroscopy^29−31^ using stable ^13^C isotope-labeled carbonyl components: 2-^13^C-acetone and benzaldehyde-α-^13^C. Complementarily, DFT calculations were performed on the NMR-detected reaction intermediates.^32,33^ In the following sections, the originally proposed reaction mechanism was challenged by new findings from NMR and DFT. This illustrates that even seemingly “simple” reaction mechanisms can be more complex than originally presumed. Using a combination of *in situ* NMR and DFT calculations, novel insights were obtained. These methods are generally applicable to obtain a detailed picture of a reaction mechanism.

## Results and Discussion

### ^13^C-Isotopic Scrambling Cannot Be Reconciled with the Originally Proposed Reaction Mechanism

**Figure 1:**
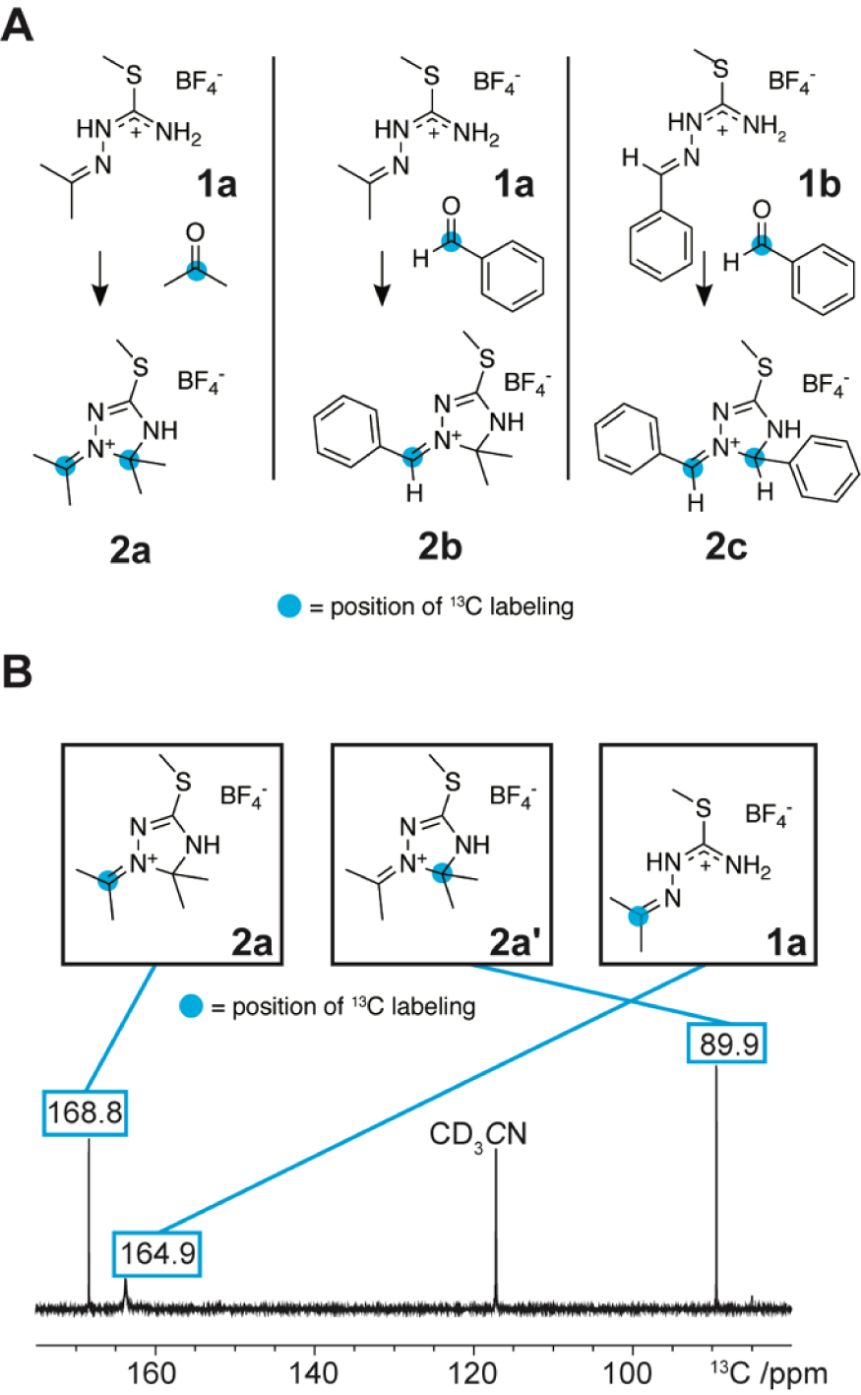
Insights into the ^13^C labeling pattern of the reaction using 2-^13^C labeled educts. (A) Overview of reaction mixture compositions to elucidate the reaction mechanism details. Left: Acetone/acetone-starting material composition. Middle: Acetone/benzaldehyde starting material composition. Right: Benzaldehyde/benzaldehyde starting material composition. ^13^C labels in the starting carbonyl compounds and the observed ^13^C labeling pattern in the products are highlighted with a blue dot. (B) 1D-^13^C-*in situ* NMR spectrum of the acetone/acetone starting material composition: the experimentally observed ^13^C labeling pattern confirms ^13^C-isotopic scrambling, resulting in compounds **2a** and **2a′**. The blue dot indicates incorporated ^13^C labels from 2-^13^C-acetone.

Our initial efforts focused on the confirmation of the originally proposed reaction mechanism. To this end, we used *in situ* NMR spectroscopy and 2-^13^C-labeled acetone and benzaldehyde-α-^13^C to directly follow product formation with three different starting material compositions leading to structure motifs already determined by single-crystal X-ray spectroscopy (Figure 1A; the different starting material compositions are hereafter denoted as acetone/acetone, acetone/benzaldehyde, or benzaldehyde/benzaldehyde. The first part represents the carbonyl compound present in the isothiosemicarbazonium educt, and the second represents the carbonyl compound added for cyclization). First, isothiosemicarbazonium tetrafluoroborate (**1a** or **1b**) was dissolved in deuterated acetonitrile. To this solution, pivalic acid/*N*,*N*-diisopropyl-*N*-ethylamine buffer was added.^19^ An aliquot of this solution was transferred into a standard 5 mm NMR tube. Then, the educt was characterized by ^1^H and ^13^C NMR spectroscopy at 50 °C. Subsequently, the reaction was initiated by the addition of 2-^13^C-acetone or benzaldehyde-α-^13^C (1.3 equiv with respect to **1a** or **1b**), and the NMR tube was again inserted into the NMR spectrometer pre-heated at 50 °C. The reaction progress was monitored *via* acquisition of 1D ^1^H and ^13^C for the next 5 h. It is noteworthy that in contrast to the reported reaction conditions, no molecular sieves, only a slight molar excess of the carbonyl reactants and milder temperatures were used to facilitate the *in situ* NMR monitoring.

Additionally, the anion was exchanged from iodide to the weakly coordinating tetrafluoroborate to avoid solubility issues in the CD_3_CN medium. As the reaction was readily conductible without the nucleophilic iodide, an influence of the anion on the reaction mechanism can be excluded. To our surprise, we did not observe the expected sole product formation of **2a** with the incorporation of the ^13^C label selectively in the iminium position of the heterocycle (Figure 1A, left and Figure 1B). Instead, we found ^13^C-isotopic scrambling, leading to ^13^C incorporation into the starting material **1a** and in two positions of product **2a** with an estimated distribution between C(5) of the triazoline and the iminium carbon of almost 50:50. The positions of the ^13^C labels are highlighted with blue dots (Figure 1B). The same isotopic scrambling was obtained in the experiment with the benzaldehyde/benzaldehyde starting material composition, leading to **2c** (Figure 1A, right). In the case of the unlabeled starting material **1a** and using benzaldehyde-α-^13^C as a reagent, however, we solely observed ^13^C incorporation in the exocyclic iminium position, giving product **2b** (Figure 1A, middle). In addition, the carbonyl exchange side reaction was strongly suppressed, showing only a minimal amount of ^13^C-labeled **1b** and unlabeled acetone. Under the assumption that all three experiments follow the same mechanistic details, the observations stand in contradiction to the originally presumed reaction mechanism (Scheme 1B).

### Expanded Reaction Mechanism from NMR Spectroscopy and DFT Calculations

**Figure 2:**
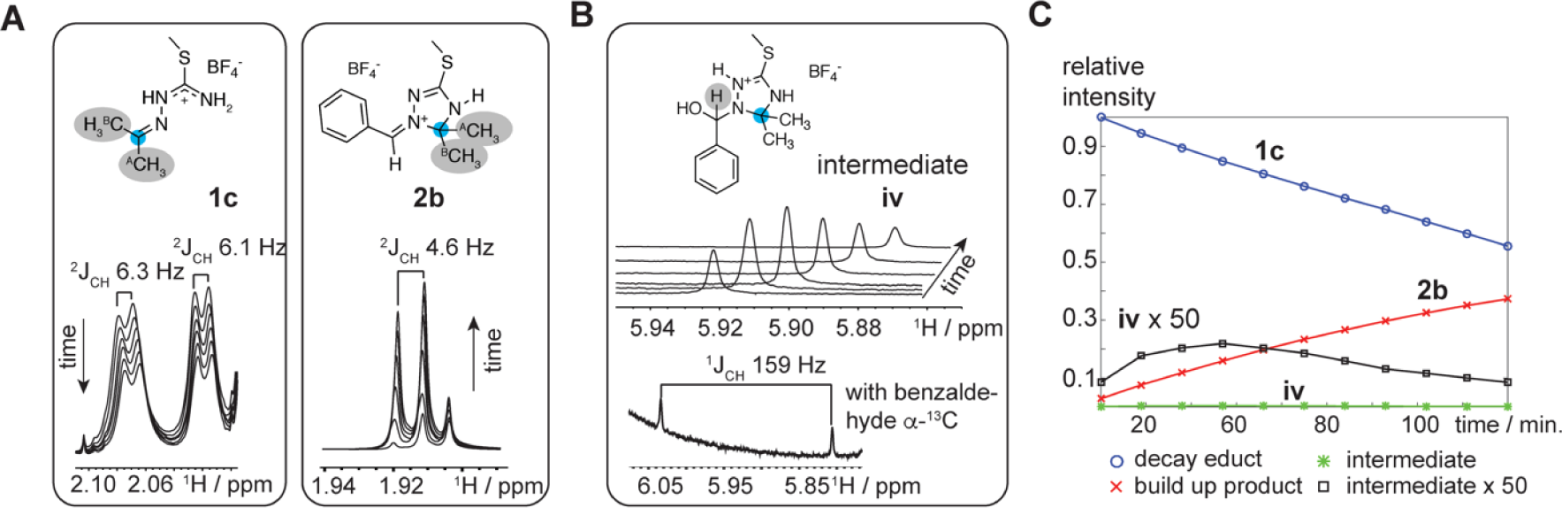
Tracking and identification of reaction intermediates by ^13^C labeling and NMR spectroscopy. (A) Decay and build-up of educt and product ^1^H resonances of **1a** and **2b**, respectively. For the 2-^13^C-acetone/benzaldehyde starting material composition, no ^13^C isotope scrambling was observed. Stacked plots show methyl group resonances in educt **1c** (decay over time) and product **2b** (build-up over time). (B) NMR spectroscopic characterization of cyclic carbinolamine reaction intermediate **iv**. The stacked plot shows the transient build-up and decay of the ^1^H resonances at 5.92 ppm assigned to reaction intermediate **iv**. When using benzaldehyde-α-^13^C as a reactant, the proton resonance at 5.92 ppm shows a ^1^*J*_CH_ coupling of 159 Hz. (C) Relative intensities of educt, product, and intermediate proton resonances. For clarity, a 50×-enhanced plot of the intermediate resonance is shown.

**Scheme 2:**
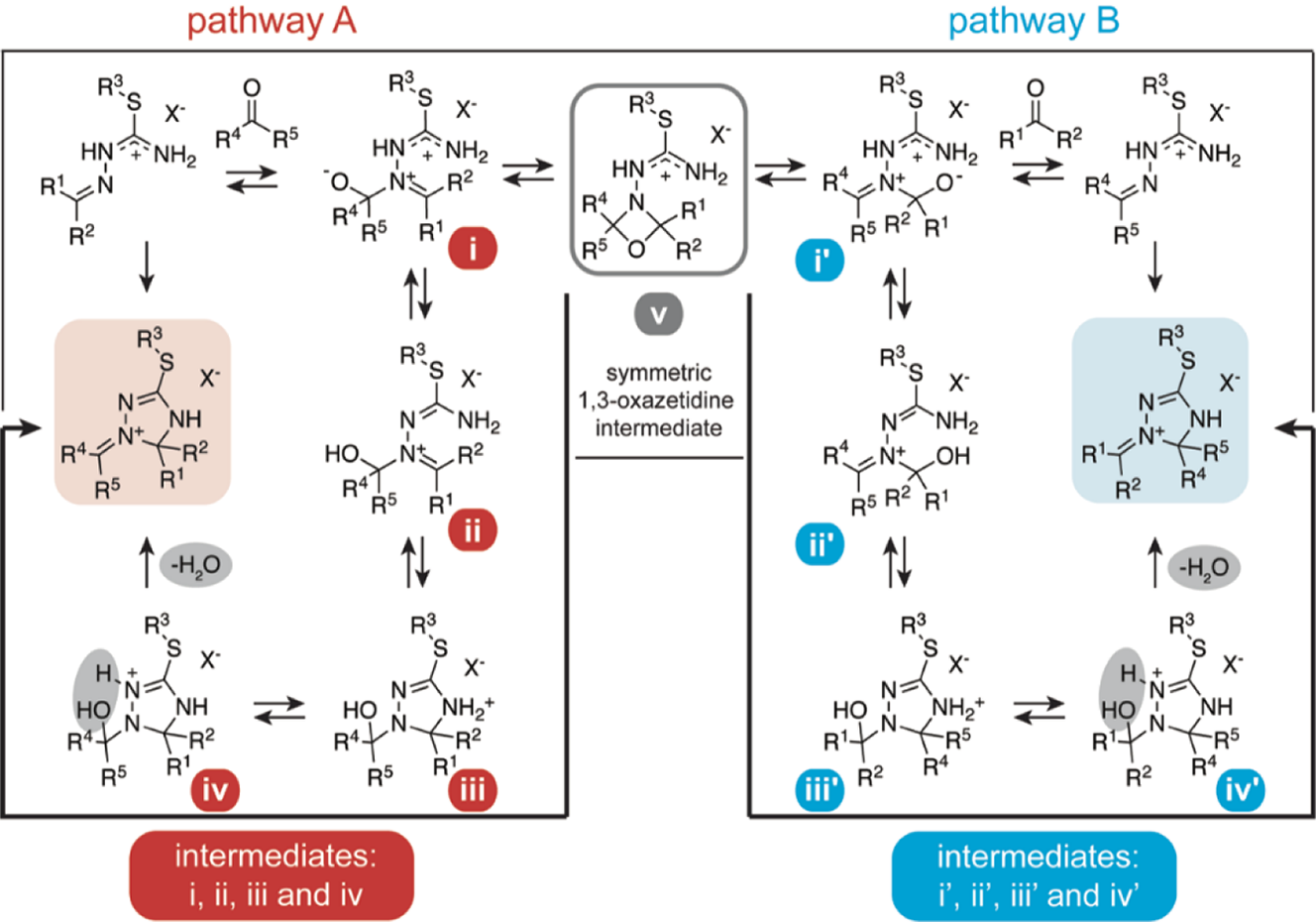
Revised Reaction Mechanism Reconciling All Experimentally Observed Phenomena and Including Results from DFT Calculations; the Intermediates and Protonation/Deprotonation Sequences Are Supposedly Stabilized and Catalyzed by Intermolecular Interactions with the Buffer System; In the Second Step of the Reaction Sequence, the Formed Intermediate **i** Can Diverge into Two Pathways; In the Productive Pathway **A** (Left, Highlighted in Red) after Proton Shift (**ii**), Intramolecular Cyclization (**iii**), and Loss of Water (**iv**), a 1,2,4-Triazolinium Structure with the Expected Substitution Pattern Is Obtained; Intermediate **i** Is However Also in Equilibrium with a Postulated 1,3-Oxazetidine Intermediate **v**; This Can in Turn Dissociate to Intermediate **i′**, Thereby Activating the Unproductive Pathway **B** (Right, Highlighted in Blue), Which Leads to the Formation of Both a 1,2,4-Triazolinium Structure with a Scrambled Substitution Pattern (over Intermediates **ii′**, **iii′**, and **iv′**) and the Generation of the Carbonyl-Exchanged Educt; the Postulated Symmetric 1,3-Oxazetidine Intermediate **v** Very Likely Plays the Key Role in the Isotopic Scrambling Process as Observed in the Acetone/Acetone and Benzaldehyde/Benzaldehyde Starting Material Composition and Can Cause Complex Product Mixtures; R^1^–R^5^ = Alkyl, Aryl, or H

In order to develop a deeper understanding of the observed isotopic scrambling, we conducted a comprehensive *in situ* NMR study focusing on HSQC and HMBC experiments to track down and identify possible intermediates. For this purpose, different labeling strategies with ^13^C labeling of the added carbonyl compounds and the isothiosemicarbazonium educt were applied. The experimental NMR data were complemented with *in silico* studies. The course of the reaction can be broken down into several distinct sequences, which will be discussed in this section in light of the experimental data and the results of the DFT calculations (Scheme 2). First, an initial nucleophilic attack of the isothiosemicarbazonium salt on the electrophilic C-atom of the carbonyl species needs to take place. The DFT calculations of the educts **1a** and **1b** gave energy-optimized geometries of the modeled molecules, which showed a trigonal planar geometry of the aminic N(1). Furthermore, the almost equal bond lengths between C(2) and its neighboring atoms and the associated Wiberg bond indices^34,35^ suggest a delocalization of the positive charge. Therefore, iminic N(3) was identified as being the most nucleophilic position of the isothiosemicarbazonium moiety, suggesting **i** as the first intermediate in the reaction sequence (Figure S2). For the subsequent steps, the originally proposed reaction mechanism was challenged by the observation of ^13^C isotopic scrambling (Figure 1B). We thus wanted to detect and characterize reaction intermediates by *in situ* NMR and using ^13^C-labeled starting reactants (Figure 1A). Thereby, the focus was laid on the acetone/benzaldehyde starting material composition, as it was the only one of the three investigated experiments to give a clean conversion in a reasonable conversion period under the aforementioned adjusted reaction conditions (Figure 2A). In the HSQC spectra, we were able to identify a peak with a proton resonance at 5.92 ppm and a carbon resonance at 79.3 ppm, showing an intensity build-up and a decay in accordance with a reaction intermediate (Figure 2B,**C**).

Using benzaldehyde-α-^13^C, the proton at 5.92 ppm was unambiguously confirmed to be attached to a ^13^C isotope (coupling ^1^*J*_CH_ = 159 Hz, Figure 2B). According to DFT calculations of the peak chemical shifts, the resonances were in good agreement with the cyclic carbinolamine structures (**iii** or **iv**, Table S3). This strong experimental evidence for the cyclic carbinolamine intermediate favors the initially proposed reaction mechanism for the formation of the 1-alkylidene/arylidene-1,2,4- triazolinium motifs. However, the observed ^13^C-scrambling for **2a** and **2c** and the selective synthesis of **2b** cannot be explained by this simple pathway. The ^13^C isotopic scrambling must occur on a very fast time scale as the formation of **2a′** was observed concomitantly with the formation of **2a** in the *in situ* NMR experiment. The simultaneous formation of **2a** and **2a′** and **2c** and **2c′** is in contradiction with a simple metathetical carbonyl exchange of starting materials **1a** and **1b**. The transazination was earlier only observed in different experiments at prolonged reaction times and refluxing temperatures, thus hinting at a presence of a higher lying activation barrier that slows down the kinetics of this reaction.^19^ As we could not find any NMR experimental evidence for a key intermediate to connect pathway **A** and **B** and to introduce ^13^C isotope scrambling, we resorted to DFT calculations. Carbonyl exchange reactions of different N-nucleophiles are long known.^36^ However, for thiosemicarbazones, the reaction was only investigated more closely in the presence of water.^28^ Owing to the similarities to the extensively studied imine metathesis in the absence of water where cyclic 4-membered intermediates were proposed, we focused our DFT study on a 1,3-oxazetidine species (Scheme 2, species **v**).^37,38^

**Figure 3:**
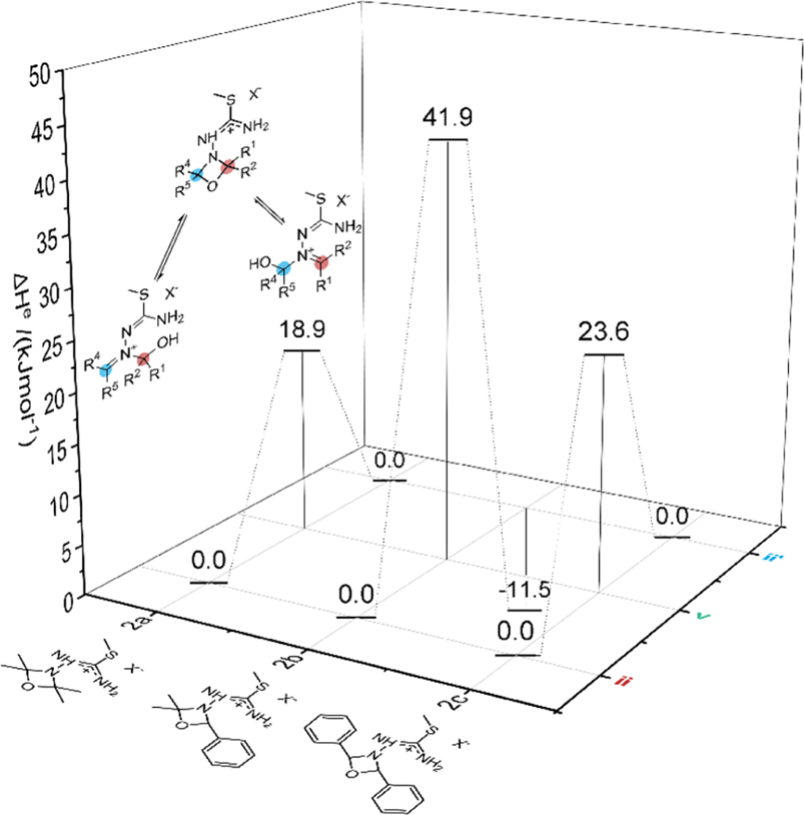
Energetic landscape of the exchange mechanism, depending on the residues R^1^, R^2^, R^4^, and R^5^. The electronic energies of the intermediates were arbitrarily referenced to the lower energetic carbinolamine intermediate. The reaction is not symmetric for **2b**. Investigating this landscape in terms of thermodynamic accessibility of the intermediates, these data indicate that the exchange pathway would be more accessible for the symmetric starting material compositions to give **2a** and **2c** than for the asymmetric starting material composition to give **2b**. B3LYP/x2c-SVPall-s; see the Supporting Information for computational details.

For all three starting material compositions, calculated electronic energies (Δ*H*^e^) of isomeric intermediates **ii**, **v**, and **ii′** were compared (Figure 3). Based on the data from the theoretical calculations, no conclusions about the associated transition states and the kinetic properties of the reaction can be obtained. Nevertheless, the comparison of the electronic energies of **ii**, **v**, and **ii′** enables an estimation of the relative equilibrium constants of the respective exchange reaction steps. These estimations confirm the ^13^C isotopic scrambling patterns as observed in the NMR reaction monitoring, hinting toward the existence of a pre-equilibrium that leads to **ii** and **ii′**. For the starting material compositions with active scrambling, the energy difference according to DFT calculations (for **2a**, Δ*H*_(ii–v)_^e^ = +18.9 kJ mol^–1^; for **2c**, Δ*H*_(ii–v)_^e^ = +23.6 kJ mol^–1^) was found to be significantly lower than that for the intermediates leading to **2b** (Δ*H*_(ii–v)_^e^ = +41.9 kJ mol^–1^). Investigations into the conformation of intermediates **v** revealed that the higher intermediate energy for **2b** arises from the steric repulsion of a phenyl and methyl group. In the case of **2c**, the repulsion is lower, since the phenyl groups can arrange in such a way that they are situated on the opposite sides of the ring plane. Under assumption of similar activation barriers in the three investigated starting material compositions, the thermodynamical equilibrium constants (*K*_(**ii**–**v**)_) and the relative state distribution (*K*_rel_ = *K*_(**ii–v**)_/*K*_(**ii–v**)**2a**_) at the applied reaction temperature (50 °C) can be estimated using the Boltzmann distribution (Table 1).

**Table 1:** Comparison of the Thermodynamic Data Obtained by DFT Calculations^a^

product	Δ*H*_(**ii–v**)_^e^/kJ mol^–1^	*K*_(**ii–v**)_	*K*_rel_
**2a**	+18.9	1.3 × 10^–3^	1
**2b**	+41.9	3.6 × 10^–7^	2.8 × 10^–4^
**2c**	+23.6	2.4 × 10^–4^	0.2

a B3LYP/x2c-SVPall-s; see the Supporting Information for computational details.

On a qualitative basis, the calculated values fairly reflect the observations of the NMR experiments. In both the acetone/acetone and benzaldehyde/benzaldehyde starting material compositions, the relative state distribution between **ii** and **v** is in an accessible range, thus leading to a superimposition of the cyclization reaction, whereas in the acetone/benzaldehyde starting material composition, the exchange pathway is thermodynamically disfavored by several orders of magnitude. In conclusion, the symmetric 1,3-oxazetidine intermediate (**v**) gives a very plausible explanation for the observed ^13^C scrambling in **2a** and **2c** and the regioselective formation of **2b**. Further attempts to validate the proposed mechanism by DFT calculations through comparison of intermediates **i**–**iv** in the acetone/benzaldehyde starting material composition were complicated by finding a proper computational model. Thus, it was not possible to account for the reaction intermediate and solvent/buffer interactions, which very likely have a significant effect on the thermodynamic accessibility and stability of the intermediates **iii** and **iv**. Therefore, the main arguments stem from the relative intermediate energies, which in turn dictate the distribution of the pre-equilibria.^39^ The gas-phase energies showed a high-energy intermediate between cyclic structures **iii** and **iv**. However, the transition from **ii** to **iv** was in a reasonable range. Most likely, the deprotonation/protonation sequences are facilitated by concerted intramolecular reactions or intermolecular reactions with the buffer systems. A possible carbonyl exchange in the acetone/benzaldehyde starting material composition seems to be thermodynamically favored, with an energy gain of −13.3 kJ mol^–1^ (Δ*H*_(ii–ii')_^e^). However, we could not find any NMR-based evidence for the scrambling product **2b′**, rather only minimal amounts of carbonyl exchange educt **1b**. In contrast to the transition of **ii** to **iv** (Δ*H*_(ii–iv)_^e^ = +1.8 kJ mol^–1^), the cyclization in pathway **B** is accompanied with a higher energy path (Δ*H*_(ii′–iv′)_^e^ = +15.7 kJ mol^–1^). This substantiates the observed preferred dissociation of intermediate **ii′** to **1b** over the experimentally not observed formation of **2b′** (Figure S9).

Alternative pathways, which do not include cyclic carbinolamine structures (**iii** and **iv**), were also considered. Especially, three-membered aziridinium structures—conceivable upon the loss of H_2_O from a protonated form of **ii**—were considered as possible intermediates since such species were postulated in earlier investigations (Scheme S1).^20^ However, the calculated NMR chemical shifts differed significantly from all observed intermediate NMR resonances. Furthermore, the absence of ^1^*J*_CC_ coupling in the detected intermediate (Figure 2) and the calculated energy differences between the respective 3-membered ring structures and products **2a–c** in the range of 240–280 kJ mol^–1^ strongly disfavor this pathway (Scheme S2).

To sum up, based on the findings of the NMR/DFT study, we propose adjustments to the initially suggested reaction mechanism (Scheme 1B *vs*Scheme 2). Following the nucleophilic attack of the iminic isothiosemicarbazonium nitrogen on the carbonyl reagent, the formed intermediate **i** can diverge into two pathways. The productive pathway **A** (Scheme 2, highlighted in red) involves—after a deprotonation/protonation step giving **ii**—an intramolecular cyclization to **iii**, which further rearranges to the NMR-detectable intermediate **iv**. Elimination of water results in the formation of the 1-alkylidene/arylidene-1,2,4-triazolinium structure motifs **2a–c**. Depending on the substitution patterns of the starting materials, a nucleophilic attack of the carbonyl oxygen onto the iminic carbon in intermediate **i** can alternatively result in the formation of a cyclic 1,3-oxazetidine intermediate (**v**). This can lead to the activation of pathway **B** (Scheme 2, highlighted in blue) upon its dissociation to intermediate **i′**. In pathway **B**, the formed **i′** either undergoes cyclization (Scheme 2, intermediates **ii′**, **iii′**, and **iv′**) to a 1,2,4-triazolinium structure with scrambled substituents or dissociates following a carbonyl exchange reaction. The thermodynamic accessibility of **v**, which seems to be strongly dependent on the chosen educts, is thus responsible for the complex product mixtures—as evidenced by the ^13^C scrambling—in the acetone/acetone and benzaldehyde/benzaldehyde starting material compositions.

### Considerations Regarding Stability and Factors Affecting Regio- and Stereoselectivity

**Figure 4:**
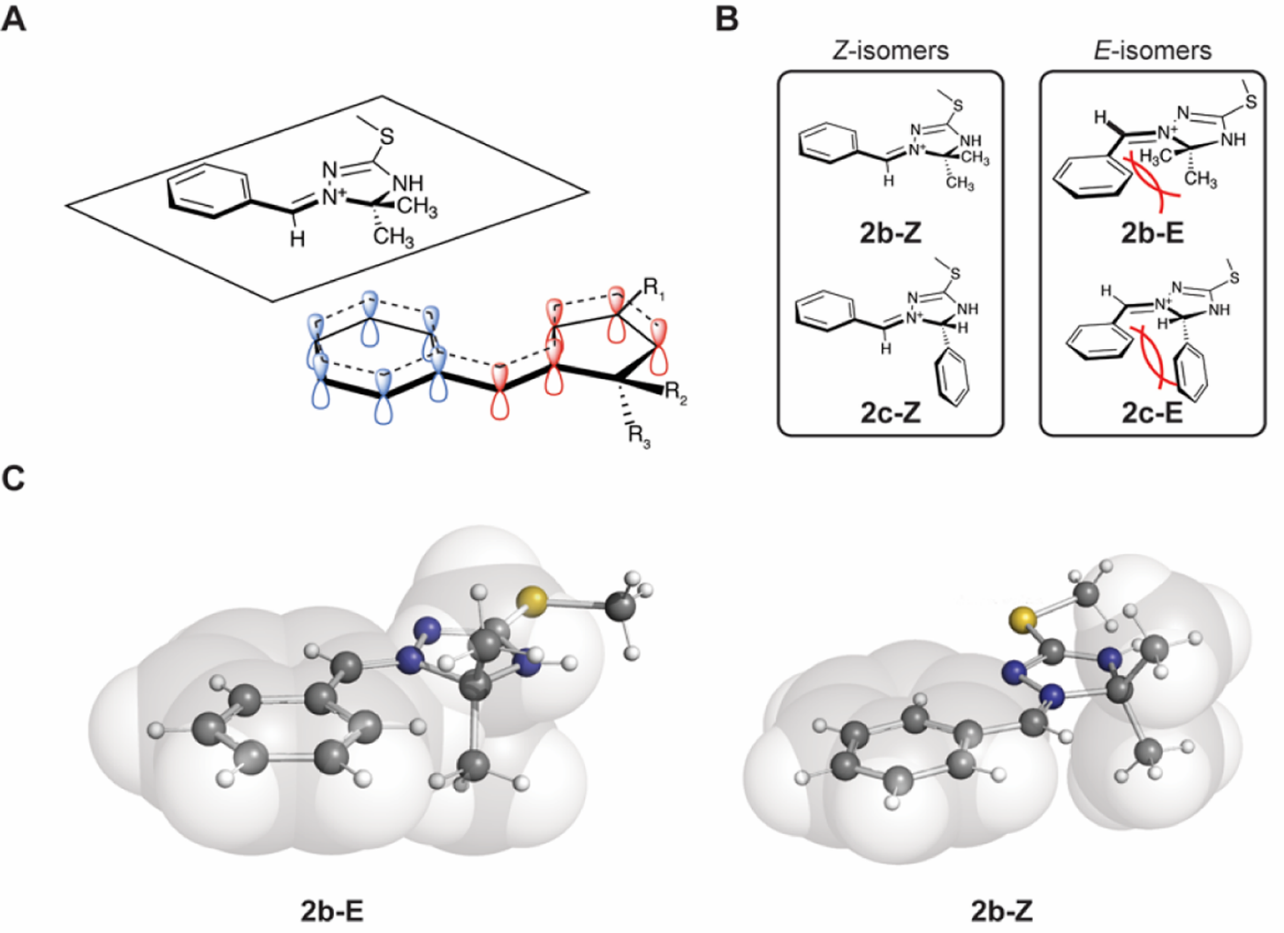
Factors affecting stability and stereoselectivity in the formation of the 1,2,4-triazolinium salts. (A) Planar structure and electrons participating in the conjugated π-system of compound **2b** (respective π-orbitals of the 1,2,4-triazolinium structure highlighted in red). The aromatic electrons of the phenyl substituent (highlighted in blue) seem to participate and further enhance the stability of **2b**. (B) Preference of the *Z*-isomer in **2b** by 39.2 kJ mol^–1^ and **2c** by 20.1 kJ mol^–1^ due to steric interference of the residues on the iminium carbon and C(5) substituents. (C) 3D ball and stick models with van der Waals radii of the possible isomers of **2b** supporting the favored formation of the *Z*-isomer due to steric reasons.

Despite the potentially reactive iminium functionality in an unstable 1,2,4-triazolinium motif,^21,22^ the structures described herein proved to be surprisingly robust and easy to handle if protected from water. Therefore, factors contributing to the stability of products **2a**–**c** were investigated. All three products showed a planar conformation in the obtained single-crystal X-ray structures^19^ and in the DFT calculations. This suggests the formation of a stabilizing expanded conjugated π-system in parts of the heterocycle and the iminium double bond. The phenyl substituents in **2b** and **2c** are located in the same plane as the heterocycle and also seem to participate in the conjugated system, further enhancing the thermodynamic stability through a mesomeric effect (Figure 4). On a qualitative basis, the empirically known rule in dihydro-triazole chemistry that disubstitutions at the sp^3^-hybridized carbon stabilize the structures also seems to apply here.^40^ Thus, the combination of the mesomeric effect and the C(5) disubstitution serves as a plausible explanation for the preferred formation of **2b** (Figure 4A). The enhanced stability hereby introduced is, for example, evidenced by the behavior toward water. Compared to **2c**, which readily dissociates under formation of the educts when exposed to trace amounts of water (as observable in the NMR spectra recorded in DMSO-*d*_6_), the iodide congener of compound **2b** could even be crystallized as a hydrate.^19^

In contrast to the acetone/acetone and benzaldehyde/benzaldehyde starting material compositions, the acetone/benzaldehyde reaction starting material composition led to the regioselective product formation of **2b**. This outcome in the formation of **2b** is presumed to be a consequence of the energetically disfavored exchange side reaction involving the 1,3-oxazetidine intermediate (**v**). We were further curious about the stereoselectivity as the NMR analysis and the X-ray crystal structure of **2b** and **2c** both revealed the *Z*-conformation at the iminium double bond. The results of the DFT calculations for the *E*/*Z* isomers of **2b** and **2c** point to a steric repulsion between the phenyl group and the substituents at C(5) in the heterocycle, resulting in a preference of the *Z*-isomer in **2b** by 39.2 kJ mol^–1^ and in **2c** by 20.1 kJ mol^–1^ (Figure 4B). Thus, in terms of molecular design for 1-alkylidene/arylidene-1,2,4-triazolinium salts, the formation of the *Z* isomer can be steered by the size of the substituent at the iminium double bond and at C(5) of the triazolinium heterocycle. In the case of **2c**, a chiral center is introduced at C(5) of the triazolinium heterocycle. As both stereoisomers possess the same free energy, there is no thermodynamic driving factor for stereoselectivity. The geometries of the reaction intermediates, however, very likely favor one C(5) stereoisomer over the other. Owing to the difficulties in the computational treatment of the intermediate—solvent/buffer interactions, we were not able to further elucidate this issue *via* DFT calculations.

### Consequences with Regard to the Synthetic Scope and Limitations

**Scheme 3:**
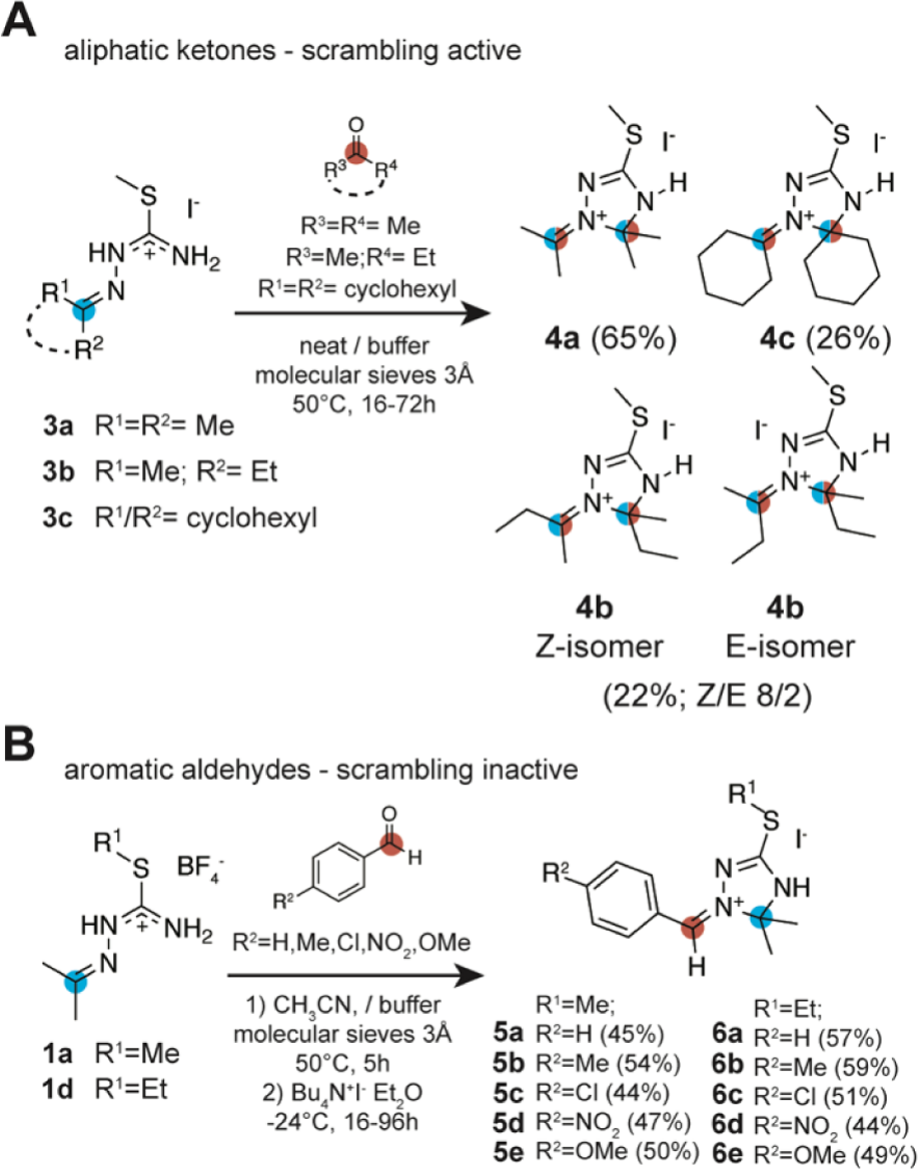
Scope of Reaction Taking into Account the Results of the NMR/DFT Mechanistic Study; (A) Practical Exploitability of the Reaction in the Case of Aliphatic Ketones is Limited to Substituents Identical to Those in the Starting Material; In This Case, the Scrambling Effect is Negligible; (B) In the Reaction with Aromatic Aldehydes, the Scrambling Effect is Energetically Disfavored and Homogeneous Products Are Obtained

To further verify the findings of the presented NMR/DFT study and to explore the scope of the reaction, 13 triazolinium derivatives with aliphatic and aromatic substituents were synthesized (Scheme 3A,B; see the Supporting Information for details). Based on the results of the previous sections, we focused on two experimental starting material compositions starting from isothiosemicarbazonium salts of aliphatic ketones. First, aliphatic ketones were reacted with isothiosemicarbazonium salts **3a–c**, since in this case, the scrambling reaction is active and depending on the used educts, no clean conversion can be expected (Scheme 3A). In the second starting material composition starting from **1a** and **1d**, para-substituted benzaldehydes were used as the carbonyl component (Scheme 3B). Here, the scrambling pathway is not active, and a regioselective product formation with the aromatic residues in the *Z* form at the iminium double bond was expected. The triazolinium salts **4a–c**, **5a–e**, and **6a–e** were isolated by recrystallization or precipitation procedures.

The isothiosemicarbazonium iodides (**3a–c**, Scheme 3) were prepared from the respective thiosemicarbazones, methyl iodide, and the respective carbonyl compound (acetone, butanone, and cyclohexanone). The starting materials **3a–c** were suspended in acetone, butanone, or cyclohexanone before the pivalic acid/*N*,*N*-diisopropyl-*N*-ethylamine buffer system and molecular sieves were added. Then, the mixtures were heated to 45–60 °C for several hours (16–72 h). Despite solubility issues in the reaction medium, the iodide salt form of the isothiosemicarbazonium starting material was used, as product crystals slowly form on the molecular sieves, allowing for a straightforward isolation of the desired product. If no crystallization was observed, the addition of diethyl ether led to product precipitation. As expected from the mechanistic study, the reaction using aliphatic ketones and starting materials with different substitution patterns led to complex product mixtures according to ^13^C NMR spectra of the collected raw products. This is a consequence of the low energetic barrier of the 1,3-oxazetidine intermediate activating both scrambling and the carbonyl exchange reaction. Typically, several peaks in the C(5) region at approximately 90 ppm were observed, as the reactants generated by the carbonyl exchange furthermore also participated in the reaction scheme (Figure S10). The experiments, however, with identical substitution patterns in the isothiosemicarbazonium starting material and the carbonyl educts gave rather satisfying results with uniform product formation and yields ranging from 20 to 70%. In the case of the symmetrical aliphatic ketones such as acetone or cyclohexanone (**4a** and **4c**), clean conversions were observed, as the scrambling pathway, although active, did not influence the product distribution. In **4b**, we observed the formation of both *E* and *Z* stereoisomers using the unsymmetrical butanone (**4b**, Scheme 3). According to a NOESY experiment, the energetically favored and expected *Z*—the isomer (approx. 80%) was predominantly formed, the *E*—isomer amounted to roughly 20%. Evidently, the steric demand of an ethyl group is insufficient to ensure a regioselective conversion.

We further investigated the reactivity of benzaldehydes with different electron-donating and -withdrawing para-substituents (−H, −Me, −OMe, −Cl, and −NO_2_). To avoid the aforementioned solubility issues in acetonitrile, isothiosemicarbazonium tetrafluoroborates **1a** and **1d** were used as educts. This allowed for lower temperatures (50 *vs* 90 °C) than in the preceding work.^19^ Thus, the thermodynamically controlled, unwanted side reaction can be further suppressed, and thermosensitive educts may be used in the preparation of the triazolinium salts. However, these adaptations led to problems in the isolation of the products since the tetrafluoroborate salts showed similar solubility properties as the buffer. Additionally, liquid/liquid extraction procedures with aqueous solutions and chromatographic purification steps were found to be unfit since, during these steps, water-induced product degradation was observed. Upon the addition of tetrabutylammonium iodide, however, the respective product iodide salts (**5a–e** and **6a–e**) could be obtained by applying a recently reported selective precipitation protocol from acetonitrile and diethyl ether.^41^ As predicted by the revised reaction mechanism, a regio- and stereoselective synthesis of all 1-benzylidene-1,2,4-triazolinium salts was achieved with isolated yields ranging from 40 to 60% (**5a–e** and **6a–e**, Scheme 3B). In contrast to the 1-alkylidene salts (**4a–c**), a complete conversion with the aromatic aldehydes was achieved in shorter reaction times (16–72 *vs* 5 h) and a lower molar excess (neat *vs* 2 equiv), which is likely ascribable to a preferred formation of products **5a–e** and **6a–e** through the mesomeric stabilization of the phenyl moieties. The influence of the different para-substituents was negligible on the reaction outcome, and the general reaction conditions could be applied for all compounds. There was, however, a difference in isolation behavior of methylthio- (**5a–e**) and ethylthio-(**6a–e**) derivatives, with the former readily precipitating from solution and the latter slowly crystallizing therefrom.

### Outlook on Possible Follow-Up Chemistry

**Scheme 4:**
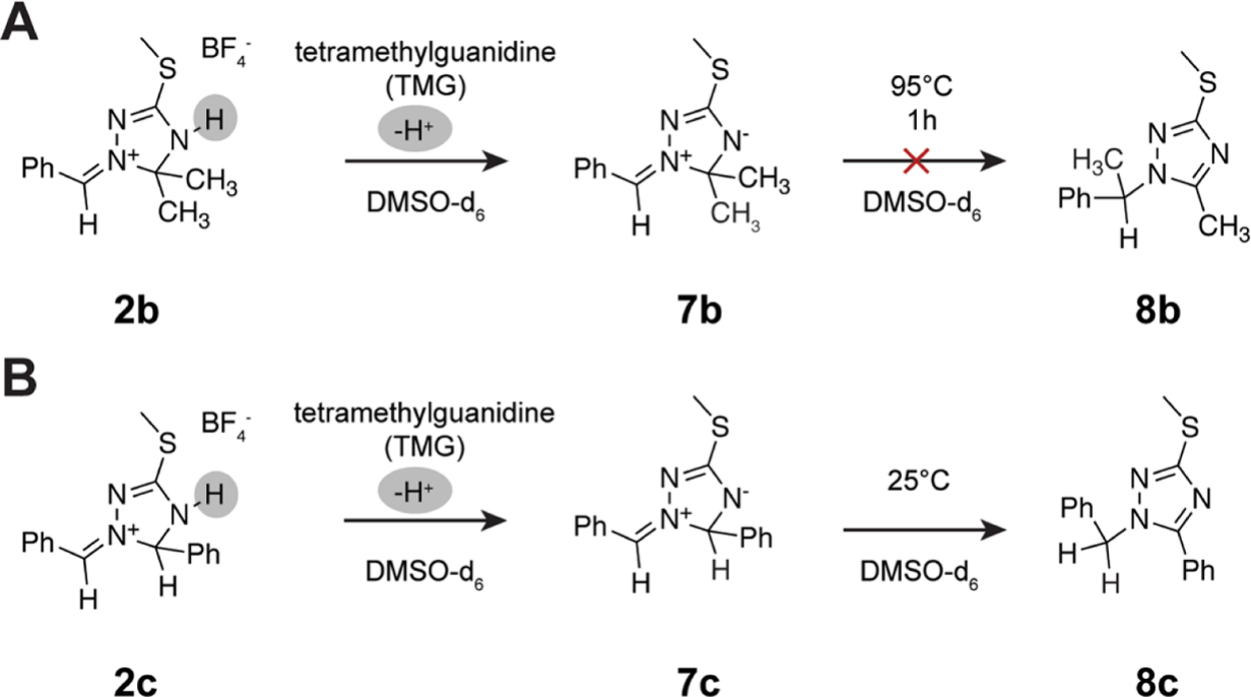
Deprotonation of Compounds **2b** and **2c** in DMSO-*d*_6_ Upon the Addition of TMG; (A) Abstraction of the NH Proton Results in the Formation of Mesomeric Betaine **7b**, Which Proved to Be Stable Even after Incubation at 95 °C for 1 h; (B) Mesomeric Betaine **7c** Spontaneously Rearranges to Form the More Stable Isomeric 1,2,4-Triazole **8c**; and Attempts to Deprotonate **2a** with TMG Led to Dissociation

To give a tentative outlook on possible follow-up chemistry of the presented compounds, the behavior of **2a–c** toward the strong base tetramethylguanidine (TMG) was investigated in solution NMR experiments using DMSO-*d*_6_ as a solvent. It was expected that the proton at N(4) would be subtracted by the base, resulting in the formation of a heterocyclic betaine structure. Indeed, the treatment of **2b** with TMG led to the disappearance of the NH peak in the ^1^H NMR spectrum and significant chemical shift changes in both ^1^H and ^13^C spectra were found, thus suggesting the formation of a zwitterionic species in solution (Scheme 4A, **7b** and Figures S12 and S13). The deprotonation leads to a bonding situation with a charge delocalization in the conjugated π-system, which is further supported by the electron density distribution according to DFT calculations (Figure S11). However, we were not able to classify the obtained structure in the extensively researched framework of mesomeric betaines.^42−44^ Upon the treatment of **2c** with TMG, the deprotonation of N(4) leading to **7c** was also observed, which spontaneously transformed to the known 1-benzyl-1,2,4-triazole **8c** (Scheme 4B and Figures S14 and S15).^45^ The underlying mechanism of this rearrangement would need further experiments using deuterium labeling at position C(5) in **2c**, and this aspect will be covered in follow-up work. The rearrangement is supported by DFT calculations with a gain in energy of 68.5 kJ mol^–1^. Attempts to induce a similar alkyl shift in **7b** through incubation of the NMR tube at 95 °C were not successful.

## Conclusions and Outlook

Based on the outcome of the mechanistic NMR/DFT study, we were able to revise the initially proposed reaction mechanism for the formation of recently discovered 1-alkylidene/arylidene-1,2,4-triazolinium salts.^19^ Depending on the carbonyl components, the originally proposed regio- and stereospecific productive cyclization pathway was found to compete with a faster, unproductive side reaction. This was evidenced by the ^13^C isotope scrambling patterns in the NMR reaction monitoring experiments. Based on DFT calculations, a 1,3-oxazetidine species is postulated as the key intermediate connecting pathway **A** and **B** and introducing ^13^C isotope scrambling in the products and metathetical carbonyl exchange to the respective isothiosemicarbazonium educts (Scheme 2). This more complex behavior leads to an updated view on the scope and limitations of the reaction to produce 1-alkylidene/arylidene-1,2,4-triazolinium salts. With the current state of knowledge, an unambiguous product formation in starting material compositions with an active scrambling pathway (e.g., acetone/acetone and benzaldehyde/benzaldehyde, products **4a–c**) is difficult. It can only be expected if the used carbonyl reactants have identical substitution patterns as the already incorporated ones in the isothiosemicarbazonium educts. In contrast, a regio- and stereoselective product formation with respect to the iminium double bond is obtained if educts are used where the scrambling pathway is suppressed by the higher energy of the 1,3-oxazetidine intermediate (acetone/benzaldehyde; products **5a–e** and **6a–e**).

By applying the reaction conditions currently in use, the scope of this novel reaction type is limited to certain educt combinations. However, it was possible to isolate a full set of homogenous 1,2,4-triazolinium salts. Considering that the investigations into the reaction are still in an early stage and as also aliphatic aldehydes and aromatic ketones have shown reactivity following this scheme,^19^ further research is needed to fully explore its scope. The updated view on the reaction mechanism with a more detailed knowledge of the unwarranted carbonyl exchange allows for the development of strategies to prevent this side reaction. Variations of the applied buffer systems or addition of other catalysts such as Lewis acids might suppress the formation of the 1,3-oxazetidine intermediate and allow for the isolation of products with unsymmetrical substitution patterns. Highlighting the mild applied reaction conditions and in many cases easily and cost effectively available starting materials, this opens new avenues in combinatoric 1,2,4-triazoline and—as shown by the deprotonation NMR experiments—1,2,4-triazole synthesis.

## Experimental Section

### General

All reagents and solvents were purchased from Sigma-Aldrich and used as received unless stated otherwise. The buffer solution used throughout the experiments was prepared by dissolving 0.2 mol (20.4 g) of pivalic acid and 0.1 mol (12.9 g) of *N*,*N*-diisopropyl-*N*-ethylamine in 100 mL of CH_3_CN, yielding a 1.0 M stock solution with approximately pH 5. Thiosemicarbazones were prepared following published procedures.^46^ Synthesis and characterization of compounds **3a**, **4a**, and **5a** were already discussed in the preceding work.^19^ However, we decided to include spectral characterization herein as well for clarity. NMR spectra of the isolated compounds were recorded with a Bruker Avance Neo 400 spectrometer or a Bruker NMR AV III 400 spectrometer. Owing to a reported ring–chain tautomerism in solution,^17^ the NMR-spectra of *S*-alkyl isothiosemicarbazonium salts (**1a–d** and **3a–c**) show more peaks with different shifts and coupling patterns than expected. Only the shifts of the main isomers are given. Structural assignments were made with additional information from ^1^H–^1^H COSY, ^1^H–^1^H NOESY ^1^H–^13^C HSQC, and ^1^H–^13^C HMBC experiments. When slightly varying the work-up procedures for **1a–d** and **3a–c**, the formation of different isomorphs can be observed, as evidenced by different appearances and melting points in hot-stage microscopy but identical NMR and MS spectra. IR spectra were obtained with a Bruker ALPHA Platinum FT-ATR instrument. HR-ESI-MS analysis was performed using a Thermo Scientific Q Exactive Orbitrap mass spectrometer (compounds **1a–c**; **2a–c**; **3a–c**; and **4a–c**; ESI positive ion mode; spray voltage 3.7 kV; solvent MeOH; and mass range from *m*/*z* 100 to 800) or with an Agilent 6350 QTOF mass spectrometer (compounds **1d**; **5a–e**; and **6a–e**; ESI positive ion mode; spray voltage 150 V; solvent MeCN; and mass range from *m*/*z* 100 to 1100).

#### *In Situ* NMR

Reaction monitoring experiments were obtained on a 600 MHz Bruker Avance II+ spectrometer equipped with a TCI Prodigy probe or a 700 MHz Avance 4 Neo spectrometer, also equipped with a TCI Prodigy probe. Standard pulse programs from the Bruker library were used: ^1^H zg30, ^13^C zgpg30, HSQC hsqcedetgpsp.3, and HMBC hmbcetgpl3nd. In order to avoid solubility issues in the CD_3_CN reaction medium associated with the iodide anion, NMR experiments were conducted with isothiosemicarbazonium tetrafluoroborates **1a–c**. To obtain clean spectra and a reasonable signal to noise ratio, no molecular sieves and only a slight molar excess of the carbonyl reactants were used.^19^ To obtain reasonable spectra of the products in CD_3_CN for comparison, 1-alkylidene/arylidene-1,2,4- triazolinium tetrafluoroborates (**2a–c**) were prepared from their iodide analogues^19^ by anion metathesis with AgBF_4_. It should be noted that the iodides were used after storage for over a year without further precautions (room temperature, closed container, and light protection), exemplifying the overall high stability of the structure motif.

#### *In Situ* Reaction Monitoring

Isothiosemicarbazonium tetrafluoroborate (1.0 mmol; **1a** or **1b**) was dissolved in deuterated acetonitrile (2000 μL). To this solution, pivalic acid/*N*,*N*-diisopropyl-*N*-ethylamine buffer was added (500 μL). An aliquot (600 μL, 0.3 mmol) of this solution was transferred into a standard 5 mm NMR tube. Then, the educt was characterized by ^1^H and ^13^C NMR spectroscopy at 50 °C. The reaction was then initiated by the addition of 2-^13^C-acetone or benzaldehyde-α-^13^C (1.3 equiv with respect to **1a** or **1b**), and the NMR tube was again inserted into the NMR spectrometer pre-heated at 50 °C. The reaction progress was monitored by the acquisition of ^1^H and ^13^C experiments for the next 5 h. It is noteworthy that in contrast to the reported reaction conditions, no molecular sieves, only a slight molar excess of the carbonyl reactants and milder temperatures were used to facilitate the shimming procedure and the spectra interpretation. Owing to these adjustments, a complete conversion was not achieved in all experimental setups.

#### Screening for Intermediates in the Acetone/Benzaldehyde Setup

Isothiosemicarbazonium tetrafluoroborate (1.0 mmol; **1a** or **1c**) was dissolved in deuterated acetonitrile (2000 μL). To this solution, pivalic acid/*N*,*N*-diisopropyl-*N*-ethylamine buffer was added (500 μL). An aliquot (600 μL, 0.3 mmol) of this solution was transferred into a standard 5 mm NMR tube. Then, the educt was characterized by ^1^H and ^13^C NMR spectroscopy at 50 or 35 °C. The reaction was then initiated by the addition of benzaldehyde-α-^13^C or freshly distilled unlabeled benzaldehyde (1.3 equiv with respect to **1a** or **1c**), and the NMR tube was again inserted into the NMR spectrometer pre-heated at 50 or 35 °C. Intermediates were followed by the acquisition of ^1^H and ^13^C 1D and 2D HSQC and HMBC experiments.

### Computational Methods

The density functional calculations were performed on the high-performance computing facility LEO3E and LEO4 (University of Innsbruck) employing the program package Gaussian 16 Rev A.03.^47−49^ The employed functional was B3LYP,^50^ and the used basis set was x2c-SVPall-s.^51^ Solution effects were implicitly taken into account *via* the polarizable continuum model.^52−54^ NMR calculations were based on the gauge-independent atomic orbital approach.^55^ As a reference substance for H and C NMR shifts, tetramethylsilane was treated identically to the investigated molecules and the isotropic shielding values (H: 31.470 ppm, C: 180.5258 ppm, 262.83 ppm B3LYP/x2c-SVPall-s) were used as references. The Wiberg bond indices were computed through natural bond orbital analysis.^34,35,56−60^ Cross-validation data for the structure optimization and the NMR shift computation to assess the accuracy of the performed calculations are listed in the Supporting Information.

### Synthetic Procedures and Product Characterization

#### General Procedures

Detailed descriptions of the synthetic procedures are listed in the Supporting Information.

##### Isothiosemicarbazonium Salts (**1a–d** and **3a–c**)

The respective thiosemicarbazone^46^ was reacted with trialkyloxonium tetrafluoroborates in CH_2_Cl_2_ (**1a–d**) or with iodomethane in CH_3_CN (**3a–c**) through heating by means of a heating mantle at varying reaction temperatures and times. For isolation, different precipitation protocols were applied.

##### 1,2,4-Triazolinium Tetrafluoroborates (**2a–c**)

The respective iodide salt^19^ was dissolved in MeOH/CH_3_CN mixtures and reacted with silver tetrafluoroborate. After removal of the resulting silver iodide residue by filtration, 1,2,4-triazolinium tetrafluoroborates (**2a–c**) were isolated from the filtrate by precipitation with Et_2_O.

##### 1-Alkylidene-1,2,4-triazolinium Iodides (**4a–c**)

The respective isothiosemicarbazonium iodide (**3a–c**) was suspended in the aliphatic ketone (neat), before molecular sieves (3 Å) and 1.0 M pivalic acid–*N*,*N*-diisopropyl-*N*-ethylamine buffer solution were added. The sealed vessel was kept at elevated temperatures (45–60 °C) by means of a heating mantle for several hours (18.5–64 h) without stirring. The formed product was isolated either by manual separation from the molecular sieves or using precipitation and recrystallization techniques.

##### 1-Benzylidene-1,2,4-triazolinium Iodides (**5a–e** and **6a–e**)

The respective isothiosemicarbazonium tetrafluoroborate (**1a** or **d**) and the para-substituted benzaldehyde reactant (2 equiv) were dissolved in CH_3_CN, before molecular sieves (3 Å) and 1.0 M pivalic acid–*N*,*N*-diisopropyl-*N*-ethylamine buffer solution were added. The sealed vessel was kept at elevated temperatures (50 °C) by means of an oil bath for 5 h without stirring. The formed colored solution was separated and mixed with a solution of tetrabutylammonium iodide dissolved in CH_3_CN. After addition of Et_2_O, the product iodides could be isolated.^41^ Further purification was achieved by recrystallization from MeOH/Et_2_O mixtures.

#### Product Characterization

##### *S*-Methyl-acetone Isothiosemicarbazonium Tetrafluoroborate (**1a**)

White, crystalline solid (3.4 g; 97%). **mp** 101–103 °C. ^**1**^**H NMR** (400 MHz, acetonitrile-*d*_3_): δ 9.90 (s, 1H), 8.33 (s, 1H), 7.69 (s, 1H), 2.67 (s, 3H), 2.11 (s, 3H), 2.03 (s, 3H) ppm. ^**13**^**C{**^**1**^**H} NMR** (101 MHz, acetonitrile-*d*_3_): δ 168.9 165.1, 25.3, 18.7, 13.7 ppm. **IR (neat)** ν: 3388 (w), 3295 (w), 3226 (w), 1633 (m), 1569 (m), 1498 (w), 1439 (m), 1377 (w), 1274 (w), 1013 (vs), 860 (w), 768 (w), 643 (m), 592 (m), 520 (w), 422 (w) cm^–1^. **HRMS** (ESI) *m*/*z*: [M]^+^ calcd for C_5_H_12_N_3_S_1_, 146.0746; found, 146.0738.

##### S-Methyl-benzaldehyde Isothiosemicarbazonium Tetrafluoroborate (**1b**)

White, crystalline solid (1.40 g; 99%). **mp** 111–113 °C. ^**1**^**H NMR** (400 MHz, acetonitrile-*d*_3_): δ 8.32 (s, 1H), 7.91–7.86 (m, 2H), 7.60–7.48 (m, 3H), 2.72 (s, 3H) ppm. ^**13**^**C{**^**1**^**H} NMR** (101 MHz, acetonitrile-*d*_3_): δ 168.7, 154.2, 133.0 (2C), 129.90 (2C), 129.3 (2C), 13.8 ppm. **IR (neat)** ν: 3614 (w), 3540 (w), 3410 (w), 3316 (w), 3246 (w), 2998 (w), 2931 (w), 2854 (w), 1639 (s), 1613 (s), 1593 (m), 1450 (w), 1383 (m), 1335 (w), 1305 (m), 1232 (w), 1019 (vs), 964 (s), 871 (w), 800 (w), 763 (m), 696 (m), 664 (m), 508 (m), 416 (w) cm^–1^. **HRMS** (ESI) *m*/*z*: [M]^+^ calcd for C_9_H_12_N_3_S_1_, 194.0746; found, 194.0735.

##### S-Methyl-(2-^13^C-acetone) Isothiosemicarbazonium Tetrafluoroborate (**1c**)

White, crystalline solid (0.47 g; 76%). **mp** 113–115 °C. ^**1**^**H NMR** (400 MHz, acetonitrile-*d*_3_): δ 9.92 (s, 1H), 7.98 (s, 2H), 2.67 (s, 3H), 2.11 (d, *J* = 6.9 Hz, 3H), 2.03 (d, *J* = 6.1 Hz, 3H) ppm. ^**13**^**C{**^**1**^**H} NMR** (101 MHz, acetonitrile-*d*_3_): δ 169.0 (d, *J* = 5.8 Hz), 165.2, 25.3 (d, *J* = 48.1 Hz), 18.8 (d, *J* = 38.4 Hz), 13.9 ppm. **IR (neat)** ν: 3387 (w), 3299 (m), 3251 (m), 3142 (m), 3090 (m), 2997 (w), 2944 (m), 2928 (m), 2853 (w), 1628 (s), 1562 (s), 1498 (m), 1441 (s), 1380 (m), 1318 (m), 1282 (m), 1245 (m), 1221 (w), 1107 (s), 1086 (s), 1018 (vs), 995 (s), 962 (s), 857 (m), 769 (w), 729 (m), 701 (m), 666 (s), 592 (m), 523 (m), 506 (m), 444 (m), 408 (w) cm^–1^. **HRMS** (ESI) *m*/*z*: [M]^+^ calcd for ^13^C_1_C_4_H_12_N_3_S_1_, 147.0780; found, 147.0771.

##### S-Ethyl-acetone Isothiosemicarbazonium Tetrafluoroborate (**1d**)

White, crystalline solid (9.9 g; 84%). **mp** 74–76 °C. ^**1**^**H NMR** (400 MHz, DMSO-*d*_6_): δ 11.76 (s, 1H), 9.50–8.58 (m, 2H), 3.25 (q, *J* = 7.3 Hz, 2H), 2.05 (d, *J* = 22.8 Hz, 5H), 1.29 (t, *J* = 7.3 Hz, 3H) ppm. ^**13**^**C{**^**1**^**H} NMR** (101 MHz, DMSO-*d*_6_): δ 165.9, 164.97, 25.6, 25.3, 19.3, 14.6 ppm. **IR (neat)** ν: 3388 (w), 3298 (w), 3235 (w), 1626 (m), 1574 (m), 1436 (w), 1272 (w), 1015 (vs), 638 (w) cm^–1^. **HRMS** (ESI) *m*/*z*: [M]^+^ calcd for C_6_H_14_N_3_S_1_, 160.0903; found, 160.0920.

##### 5,5-Dimethyl-3-(methylthio)-1-(propan-2-ylidene)-4,5-dihydro-1*H*-1,2,4-triazol-1-ium Tetrafluoroborate (**2a**)

White powder (0.22 g; 80%). **mp** 140–142 °C. ^**1**^**H NMR** (400 MHz, acetonitrile-*d*_3_): δ 7.78 (s, 1H), 2.58 (s, 3H), 2.55 (s, 3H), 2.46 (s, 3H), 1.90 (s, 6H) ppm. ^**1**^**H NMR** (400 MHz, DMSO-*d*_6_): δ 9.97 (s, 1H), 2.57 (s, 6H), 2.43 (s, 3H), 1.84 (s, 6H) ppm. ^**13**^**C{**^**1**^**H} NMR** (101 MHz, acetonitrile-*d*_3_): δ 169.2, 168.4, 90.3, 26.8 (2C), 25.6, 22.5, 13.8 ppm. ^**13**^**C{**^**1**^**H} NMR** (101 MHz, DMSO-*d*_6_): δ 167.6, 166.4, 88.9, 26.2 (2C), 25.0, 21.8, 12.9 ppm. **IR (neat)** ν: 3317 (w), 1646 (w), 1511 (s), 1486 (m), 1454 (m), 1432 (m), 1400 (m), 1383 (w), 1300 (w), 1208 (w), 1063 (vs), 1037 (s), 1013 (s), 985 (s), 967 (s), 892 (w), 872 (w), 769 (w), 715 (w), 678 (w), 561 (w), 523 (m), 477 (w) cm^–1^. **HRMS** (ESI) *m*/*z*: [M]^+^ calcd for C_8_H_16_N_3_S_1_, 186.1059; found, 186.1049.

##### (*Z*) 1-Benzylidene-5,5-dimethyl-3-(methylthio)-4,5-dihydro-1*H*-1,2,4-triazol-1-ium Tetrafluoroborate (**2b**)

Slightly yellow powder (0.21 g; 79%). **mp** 176–178 °C. ^**1**^**H NMR** (400 MHz, acetonitrile-*d*_3_): δ 8.48–8.42 (m, 2H), 8.11 (s, 1H), 7.81–7.75 (m, 1H), 7.73–7.66 (m, 2H), 2.75 (s, 3H), 1.90 (s, 6H) ppm. ^**1**^**H NMR** (400 MHz, DMSO-*d*_6_): δ 10.95 (s, 1H), 8.67 (s, 1H), 8.45–8.39 (m, 2H), 7.81–7.66 (m, 3H), 2.75 (s, 3H), 1.87 (s, 6H) ppm. ^**13**^**C{**^**1**^**H} NMR** (101 MHz, acetonitrile-*d*_3_): δ 172.6, 143.1, 136.1, 134.5 (2C), 130.3, 128.8 (2C), 92.1, 28.8 (2C), 14.3 ppm. ^**13**^**C{**^**1**^**H} NMR** (101 MHz, DMSO-*d*_6_): δ 170.7, 141.5, 134.8, 133.2 (2C), 129.4 (2C), 128.0, 91.0, 28.3 (2C), 13.4 ppm. **IR (neat)** ν: 3278 (w), 1630 (w), 1593 (w), 1484 (s), 1451 (s), 1408 (m), 1389 (m), 1328 (w), 1303 (w), 1283 (w), 1226 (w), 1200 (w), 1130 (w), 1053 (s), 994 (vs), 944 (s), 911 (m), 876 (w), 831 (m), 764 (s), 686 (s), 606 (w), 542 (m), 524 (m), 504 (m), 484 (m), 432 (w) cm^–1^. **HRMS** (ESI) *m*/*z*: [M]^+^ calcd for C_12_H_16_N_3_S_1_, 234.1059; found, 234.1045.

##### (*Z*)-1-Benzylidene-3-(methylthio)-5-phenyl-4,5-dihydro-1*H*-1,2,4-triazol-1-ium Tetrafluoroborate (**2c**)

Slightly yellow crystalline solid (0.30 g; 81%). According to NMR data, the MeCN monosolvate **2c**·**MeCN** was isolated. **mp** 169–171 °C. ^**1**^**H NMR** (400 MHz, acetonitrile-*d*_3_): δ 8.47 (s, 1H), 8.37–8.32 (m, 2H), 7.86 (d, *J* = 2.5 Hz, 1H), 7.79–7.74 (m, 1H), 7.68–7.59 (m, 7H), 7.04 (d, *J* = 2.5 Hz, 1H), 2.83 (s, 3H) ppm. ^**1**^**H NMR** (400 MHz, DMSO-*d*_6_–slight dissociation): δ 11.32 (s, 1H), 8.45–8.40 (m, 2H), 8.35 (d, *J* = 2.5 Hz, 1H), 7.76 (t, *J* = 7.5 Hz, 1H), 7.70–7.64 (m, 2H), 7.61 (s, 5H), 7.38 (d, *J* = 2.5 Hz, 1H), 2.83 (s, 3H) ppm. ^**13**^**C{**^**1**^**H} NMR** (101 MHz, acetonitrile-*d*_3_): δ 175.4, 145.9, 136.5, 135.1, 134.7 (2C), 132.7, 130.8, 130.4 (2C), 129.1 (2C), 128.2, 118.3, 87.2, 14.5 ppm. ^**13**^**C{**^**1**^**H} NMR** (101 MHz, DMSO-*d*_6_–slight dissociation): δ 173.6, 143.8, 135.2, 135.1, 133.5 (2C), 131.5, 129.8 (2C), 129.5 (2C), 127.8 (2C), 127.5, 86.1, 13.7 ppm. **IR (neat)** ν: 3278 (w), 1630 (w), 1593 (w), 1484 (s), 1451 (s), 1408 (m), 1389 (m), 1328 (w), 1303 (w), 1283 (w), 1226 (w), 1200 (w), 1130 (w), 1053 (s), 994 (vs), 944 (s), 911 (m), 876 (w), 831 (m), 764 (s), 686 (s), 606 (w), 542 (m), 524 (m), 504 (m), 484 (m), 432 (w) cm^–1^. **HRMS** (ESI) *m*/*z*: [M]^+^ calcd for C_16_H_16_N_3_S_1_, 282.1059; found, 282.1042.

##### S-Methyl-acetoneisothiosemicarbazonium Iodide (**3a**)

White solid (20.1 g; 98%). **mp** 174–176 °C. ^**1**^**H NMR** (400 MHz, DMSO-*d*_6_): δ 11.72 (s, 1H), 9.25 (s, 2H), 2.67 (s, 3H), 2.09 (s, 3H), 2.03 (s, 3H) ppm. ^**13**^**C{**^**1**^**H} NMR** (75 MHz, DMSO-*d*_6_): δ 166.1, 164.55, 25.0, 18.9, 13.5 ppm. **IR (neat)** ν: 3223 (m), 3168 (m), 3078 (s), 2970 (m), 1653 (w), 1613 (vs), 1559 (vs), 1491 (m), 1432 (s), 1372 (m), 1321 (m), 1272 (s), 1225 (m), 1108 (m), 1082 (m), 1021 (m), 984 (m), 861 (m), 770 (s), 651 (vs), 593 (m), 509 (m), 417 (m) cm^–1^. **HRMS** (ESI) *m*/*z*: [M]^+^ calcd for C_5_H_12_N_3_S_1_, 146.0746; found, 146.0738.

##### S-Methyl-butanone Isothiosemicarbazonium Iodide (**3b**)

White powder (19.0 g; 88%). **mp** 96–98 °C. ^**1**^**H NMR** (400 MHz, DMSO-*d*_6_): δ 11.66 (s, 1H), 9.37–8.69 (m, 2H), 2.73–2.53 (m, 3H), 2.45–2.28 (m, 2H), 2.10–1.94 (m, 2H), 1.15–0.99 (m, 3H) ppm. ^**13**^**C{**^**1**^**H} NMR** (101 MHz, DMSO-*d*_6_): δ 168.0, 166.2, 31.6, 17.5, 13.6, 10.4 ppm. **IR (neat)** ν: 3256 (m), 3179 (m), 3050 (m), 2972 (m), 2932 (m), 1625 (vs), 1567 (s), 1462 (m), 1438 (m), 1414 (s), 1366 (m), 1318 (m), 1223 (s), 1121 (m), 1074 (s), 989 (m), 963 (m), 728 (m), 702 (m), 662 (m), 598 (m), 573 (m), 525 (s), 471 (m), 428 (m) cm^–1^. **HRMS** (ESI) *m*/*z*: [M]^+^ calcd for C_6_H_14_N_3_S_1_, 160.0903; found, 160.0894.

##### S-Methyl-cyclohexanone Isothiosemicarbazonium Iodide (**3c**)

White, crystalline solid (15.8 g, 67%). According to NMR, besides the *ring*—*chain* tautomerism^17^ also *chair*—*boat* isomers of the cyclohexylidene moiety are formed in solution. **mp** 109–111 °C. ^**1**^**H NMR** (400 MHz, acetonitrile-*d*_3_): δ 11.21 (s, 1H), 9.45–7.47 (m, 2H), 2.75 (s, 1.5H), 2.68–2.63 (m, 1H), 2.63–2.59 (m, 1H), 2.58 (s, 1.5H), 2.43 (t, *J* = 6.3 Hz, 1H), 2.34–2.30 (m, 1H), 1.78–1.59 (m, 6H) ppm. ^**13**^**C{**^**1**^**H} NMR** (101 MHz, acetonitrile-*d*_3_): δ 174.2, 172.0, 168.6, 167.6, 35.9, 35.6, 30.9, 30.7, 28.0, 27.1, 26.8, 25.7, 25.6, 15.5, 14.7 ppm. **IR (neat)** ν: 3298 (m), 3251 (m), 3144 (m), 3095 (s), 2996 (w), 2944 (m), 2929 (m), 2855 (w), 1625 (vs), 1563 (s), 1498 (w), 1445 (s), 1423 (s), 1353 (m), 1318 (m), 1284 (m), 1243 (w), 1221 (w), 1109 (s), 1091 (s), 1020 (w), 994 (m), 961 (m), 845 (w), 730 (m), 701 (m), 668 (s), 604 (m), 578 (m), 530 (s), 477 (m), 445 (m) cm^–1^. **HRMS** (ESI) *m*/*z*: [M]^+^ calcd for C_8_H_16_N_3_S_1_, 186.1059; found, 186.1049.

##### 5,5-Dimethyl-3-(methylthio)-1-(propan-2-ylidene)-4,5-dihydro-1*H*-1,2,4-triazol-1-ium Iodide (**4a**)

Slightly yellow crystals (1.01 g; 65%). **mp** 200–202 °C. ^**1**^**H NMR** (400 MHz, DMSO-*d*_6_): δ 9.92 (s, 1H), 2.60 (s, 3H), 2.57 (s, 3H), 2.43 (s, 3H), 1.85 (s, 6H) ppm. ^**13**^**C{**^**1**^**H} NMR** (101 MHz, DMSO-*d*_6_): δ 167.4, 166.2, 88.7, 26.4 (2C), 25.2, 22.3, 13.1 ppm. **IR (neat)** ν: 3231 (w), 3143 (m), 2997 (w), 2910 (w), 1643 (m), 1500 (s), 1476 (vs), 1444 (vs), 1420 (vs), 1393 (s), 1377 (s), 1296 (m), 1259 (m), 1203 (s), 1165 (m), 1113 (m), 1064 (m), 1035 (m), 986 (m), 964 (m), 891 (m), 872 (m), 715 (w), 677 (w), 559 (m), 491 (m), 476 (m) cm^–1^. **HRMS** (ESI) *m*/*z*: [M]^+^ calcd for C_8_H_16_N_3_S_1_, 186.1059; found, 186.1048.

##### 1-(Butan-2-ylidene)-5-ethyl-5-methyl-3-(methylthio)-4,5-dihydro-1*H*-1,2,4-triazol-1-ium Iodide-Mixture of *Z*- and *E*-Isomers (**4b**)

Yellowish, crystalline solid (0.19 g; 22%). According to NMR, a mixture of the *Z*- and *E*-isomer was isolated, with a proportion of approximately 80% (*Z*) to 20% (*E*), based on the integrals of the alkylidene methyl peaks in the ^1^H NMR spectrum at 2.57 ppm (*Z*) and 2.45 ppm (*E*). **mp** 192–194 °C (decomposition). ^**1**^**H NMR** (400 MHz, DMSO-*d*_6_—only the peaks of the *Z*-isomer are given): δ 9.94 (s, 1H), 2.91–2.66 (m, 2H), 2.60 (s, 3H), 2.57 (s, 3H), 2.39–2.24 (m, 1H), 2.01–1.91 (m, 1H), 1.87 (s, 3H), 1.16 (t, *J* = 7.5 Hz, 3H), 0.80 (t, *J* = 7.3 Hz, 3H) ppm. ^**13**^**C{**^**1**^**H} NMR** (101 MHz, DMSO-*d*_6_—only the peaks of the *Z*-isomer are given): δ 171.3, 167.2, 92.3, 31.2, 30.9, 25.2, 19.6, 13.1, 8.9, 6.9 ppm. **IR (neat)** ν: 3339 (w), 3116 (m), 2975 (m), 2936 (m), 2910 (m), 2728 (w), 1623 (w), 1505 (vs), 1481 (s), 1448 (vs), 1423 (vs), 1384 (s), 1367 (s), 1351 (m), 1289 (m), 1260 (m), 1192 (m), 1133 (m), 1103 (w), 1042 (m), 1022 (m), 994 (s), 966 (s), 938 (m), 862 (w), 751 (w), 598 (w), 554 (w), 507 (m), 487 (m) cm^–1^. **HRMS** (ESI) *m*/*z*: [M]^+^ calcd for C_10_H_20_N_3_S_1_, 214.1372; found, 214.1359.

##### 1-Cyclohexylidene-3-(methylthio)-1,2,4-triazaspiro[4.5]dec-2-en-1-ium Iodide (**4c**)

Yellowish crystals (0.52 g; 26%). **mp** 177–179 °C (decomposition) ^**1**^**H NMR** (400 MHz, DMSO-*d*_6_): δ 10.23 (s, 1H), 3.03 (dt, *J* = 9.7 Hz, 6.2 Hz, 4H), 2.56 (s, 3H), 2.28 (td, *J* = 12.6 Hz, 4.4 Hz, 2H), 2.01 (d, *J* = 12.7 Hz, 2H), 1.89–1.76 (m, 6H), 1.70–1.42 (m, 6H) ppm. ^**13**^**C{**^**1**^**H} NMR** (101 MHz, DMSO-*d*_6_): δ 173.1, 166.3, 92.6, 34.7 (2C), 33.5, 31.4, 27.3, 26.2, 23.6, 22.8, 22.1 (2C), 13.1 ppm. **IR (neat)** ν: 3046 (m), 2924 (m), 2896 (m), 2862 (m), 2694 (w), 1617 (w), 1502 (vs), 1476 (s), 1456 (s), 1314 (m), 1303 (m), 1240 (m), 1165 (w), 1142 (w), 1111 (w), 1067 (w), 1020 (m), 982 (s), 906 (m), 857 (w), 713 (w), 527 (m), 514 (m), 469 (w) cm^–1^. **HRMS** (ESI) *m*/*z*: [M]^+^ calcd for C_14_H_24_N_3_S_1_, 266.1685; found, 266.1669.

##### (*Z*)-1-Benzylidene-5,5-dimethyl-3-(methylthio)-4,5-dihydro-1*H*-1,2,4-triazol-1-ium Iodide (**5a**)

Yellow crystals (0.41 g; 45%). **mp** 179–181 °C (decomposition). ^**1**^**H NMR** (400 MHz, DMSO-*d*_6_): δ 10.93 (s, 1H), 8.80 (s, 1H), 8.48–8.42 (m, 2H), 7.80–7.67 (m, 3H), 2.75 (s, 3H), 1.89 (s, 6H) ppm. ^**13**^**C{**^**1**^**H} NMR** (101 MHz, DMSO-*d*_6_): δ 170.7, 141.4, 134.7, 133.2 (2C), 129.4 (2C), 127.9, 91.0, 28.3 (2C), 13.6 ppm. **IR (neat)** ν: 3012 (m), 1628 (w), 1592 (w), 1467 (vs), 1198 (s), 995 (s), 782 (s), 684 (s), 504 (m) cm^–1^. **HRMS** (ESI) *m*/*z*: [M]^+^ calcd for C_12_H_16_N_3_S_1_, 234.1059; found, 234.1064.

##### (*Z*)-5,5-Dimethyl-1-(4-methylbenzylidene)-3-(methylthio)-4,5-dihydro-1*H*-1,2,4-triazol-1-ium Iodide (**5b**)

Yellow crystals (0.51 g; 54%). **mp** 198–200 °C (decomposition). ^**1**^**H NMR** (400 MHz, DMSO-*d*_6_): δ 10.83 (s, 1H), 8.72 (s, 1H), 8.34 (d, *J* = 7.9 Hz, 2H), 7.52 (d, *J* = 8.0 Hz, 2H), 2.74 (s, 3H), 2.45 (s, 3H), 1.86 (s, 6H) ppm. ^**13**^**C{**^**1**^**H} NMR** (101 MHz, DMSO-*d*_6_): δ 170.3, 146.0, 141.5, 133.3 (2C), 130.0 (2C), 125.4, 90.6, 28.3 (2C), 21.6, 13.5 ppm. **IR (neat)** ν: 3012 (m), 1598 (m), 1440 (vs), 1379 (vs) 1185 (s), 1064 (s), 980 (s), 875 (s), 762 (s), 508 (vs) cm^–1^. **HRMS** (ESI) *m*/*z*: [M]^+^ calcd for C_13_H_18_N_3_S_1_, 248.1216; found, 248.1227.

##### (*Z*)-1-(4-Chlorobenzylidene)-5,5-dimethyl-3-(methylthio)-4,5-dihydro-1*H*-1,2,4-triazol-1-ium Iodide (**5c**)

Orange crystalline solid (0.43 g; 44%). **mp** 182–184 °C (decomposition). ^**1**^**H NMR** (400 MHz, DMSO-*d*_6_): δ 11.01 (s, 1H), 8.77 (s, 1H), 8.44 (d, *J* = 7.1 Hz, 2H), 7.79 (d, *J* = 8.7 Hz, 2H), 2.74 (s, 3H), 1.88 (s, 6H) ppm. ^**13**^**C{**^**1**^**H} NMR** (101 MHz, DMSO-*d*_6_): δ 170.9, 140.0, 139.3, 134.7 (2C), 129.6 (2C), 126.8, 91.3, 28.3 (2C), 13.6 ppm. **IR (neat)** ν: 2982 (m), 2884 (m), 1580 (m), 1483 (m), 1372 (s), 1093 (m), 869 (s), 812 (s), 506 (vs) cm^–1^. **HRMS** (ESI) *m*/*z*: [M]^+^ calcd for C_12_H_15_N_3_S_1_Cl_1_, 268.0670; found, 268.0677.

##### (*Z*)-5,5-Dimethyl-3-(methylthio)-1-(4-nitrobenzylidene)-4,5-dihydro-1*H*-1,2,4-triazol-1-ium Iodide (**5d**)

Dark-orange, crystalline solid (0.48 g; 47%). **mp** 181–183 °C (decomposition). ^**1**^**H NMR** (400 MHz, DMSO-*d*_6_): δ 11.27 (s, 1H), 8.90 (s, 1H), 8.65 (d, *J* = 9.0 Hz, 2H), 8.48 (d, *J* = 9.0 Hz, 2H), 2.77 (s, 3H), 1.91 (s, 6H) ppm. ^**13**^**C{**^**1**^**H} NMR** (101 MHz, DMSO-*d*_6_): δ 171.8, 149.3, 138.1, 133.9 (2C), 133.3, 124.1 (2C), 92.6, 28.3 (2C), 13.7 ppm. **IR (neat)** ν: 3033 (m), 2945 (m), 1595 (s), 1517 (s), 1442 (vs), 1301 (s), 990 (s), 868 (s), 745 (s), 682 (s), 498 (m) cm^–1^. **HRMS** (ESI) *m*/*z*: [M]^+^ calcd for C_12_H_15_N_4_S_1_O_2_, 279.0910; found, 279.0911.

##### (*Z*)-1-(4-Methoxybenzylidene)-5,5-dimethyl-3-(methylthio)-4,5-dihydro-1*H*-1,2,4-triazol-1-ium Iodide (**5e**)

Yellow, crystalline solid (0.49 g; 50%). **mp** 185–186 °C (decomposition). ^**1**^**H NMR** (400 MHz, DMSO-*d*_6_): δ 10.66 (s, 1H), 8.68 (s, 1H), 8.44 (d, *J* = 9.1 Hz, 2H), 7.26 (d, *J* = 9.1 Hz, 2H), 3.92 (s, 3H), 2.73 (s, 3H), 1.85 (s, 6H) ppm. ^**13**^**C{**^**1**^**H} NMR** (101 MHz, DMSO-*d*_6_): δ 169.5, 164.4, 141.1, 136.0 (2C), 120.6, 115.1 (2C), 89.8, 56.0, 28.4 (2C), 13.5 ppm. **IR (neat)** ν: 3090 (m), 2940 (m), 1592 (s), 1450 (vs), 1260 (s), 1173 (s), 1016 (s), 836 (vs), 575 (m), 478 (m) cm^–1^. **HRMS** (ESI) *m*/*z*: [M]^+^ calcd for C_13_H_18_N_3_S_1_O_1_, 264.1165; found, 264.1170.

##### (*Z*)-1-Benzylidene-3-(ethylthio)-5,5-dimethyl-4,5-dihydro-1*H*-1,2,4-triazol-1-ium Iodide (**6a**)

Yellow solid (0.53 g; 57%). **mp** 167–169 °C (decomposition). ^**1**^**H NMR** (400 MHz, DMSO-*d*_6_): δ 10.94 (s, 1H), 8.82 (s, 1H), 8.50–8.41 (m, 2H), 7.80–7.66 (m, 3H), 3.33 (q, *J* = 7.3 Hz, 2H), 1.89 (s, 6H), 1.45 (t, *J* = 7.3 Hz, 3H) ppm. ^**13**^**C{**^**1**^**H} NMR** (101 MHz, DMSO-*d*_6_): δ 169.7, 141.5, 134.7, 133.1 (2C), 129.4 (2C), 128.0, 90.6, 28.3 (2C), 25.8, 14.7 ppm. **IR (neat)** ν: 3114 (m), 2957 (m), 1592 (s), 1442 (vs), 1200 (s), 988 (s), 756 (s), 688 (s), 490 (m) cm^–1^. **HRMS** (ESI) *m*/*z*: [M]^+^ calcd for C_13_H_18_N_3_S_1_, 248.1216; found, 248.1219.

##### (*Z*)-3-(Ethylthio)-5,5-dimethyl-1-(4-methylbenzylidene)-4,5-dihydro-1*H*-1,2,4-triazol-1-ium Iodide (**6b**)

Yellow solid (0.57 g; 59%). **mp** 192–195 °C (decomposition). ^**1**^**H NMR** (400 MHz, DMSO-*d*_6_): δ 10.84 (s, 1H), 8.76 (s, 1H), 8.35 (d, *J* = 8.4 Hz, 2H), 7.52 (d, *J* = 8.2 Hz, 2H), 3.32 (q, *J* = 7.3 Hz, 2H), 2.44 (s, 3H), 1.87 (s, 6H), 1.44 (t, *J* = 7.3 Hz, 3H) ppm. ^**13**^**C{**^**1**^**H} NMR** (101 MHz, DMSO-*d*_6_): δ 169.3, 146.0, 141.5, 133.2 (2C), 130.0 (2C), 125.4, 90.1, 28.3 (2C), 25.7, 21.6, 14.7 ppm. **IR (neat)** ν: 3051 (w), 2942 (w), 1597 (m), 1485 (s), 1450 (s), 1185 (m), 979 (m), 812 (s), 506 (vs) cm^–1^. **HRMS** (ESI) *m*/*z*: [M]^+^ calcd for C_14_H_20_N_3_S_1_, 262.1372; found, 262.1376.

##### (*Z*)-1-(4-Chlorobenzylidene)-3-(ethylthio)-5,5-dimethyl-4,5-dihydro-1*H*-1,2,4-triazol-1-ium Iodide (**6c**)

Orange crystals (0.52 g; 51%). **mp** 174–175 °C (decomposition). ^**1**^**H NMR** (400 MHz, DMSO-*d*_6_): δ 11.02 (s, 1H), 8.83 (s, 1H), 8.45 (d, *J* = 8.8 Hz, 2H), 7.84–7.75 (m, 2H), 3.33 (q, *J* = 7.3 Hz, 2H), 1.89 (s, 6H), 1.44 (t, *J* = 7.3 Hz, 3H) ppm. ^**13**^**C{**^**1**^**H} NMR** (101 MHz, DMSO-*d*_6_): δ 169.9, 140.1, 139.3, 134.6 (2C), 129.6 (2C), 126.8, 90.8, 28.3 (2C), 25.8, 14.7 ppm. **IR (neat)** ν: 3040 (m), 2951 (m), 1586 (m), 1474 (vs), 1380 (s), 1198 (m), 1061 (m), 825 (vs), 509 (s) cm^–1^. **HRMS** (ESI) *m*/*z*: [M]^+^ calcd for C_13_H_17_N_3_S_1_Cl_1_, 282.0826; found, 282.0829.

##### (*Z*)-3-(Ethylthio)-5,5-dimethyl-1-(4-nitrobenzylidene)-4,5-dihydro-1*H*-1,2,4-triazol-1-ium Iodide (**6d**)

Dark-orange solid (0.46 g; 44%). **mp** 178–180 °C (decomposition). ^**1**^**H NMR** (400 MHz, DMSO-*d*_6_): δ 11.32 (s, 1H), 8.86 (s, 1H), 8.63 (d, *J* = 9.0 Hz, 2H), 8.50 (d, *J* = 9.0 Hz, 2H), 3.36 (q, *J* = 7.3 Hz, 2H), 1.90 (s, 6H), 1.46 (t, *J* = 7.3 Hz, 3H) ppm. ^**13**^**C{**^**1**^**H} NMR** (101 MHz, DMSO-*d*_6_): δ 170.9, 149.3, 138.1, 133.7 (2C), 133.4, 124.2 (2C), 92.3, 28.3 (2C), 25.9, 14.7 ppm. **IR (neat)** ν: 3027 (m), 2940 (m), 1598 (s), 1518 (s), 1375 (s), 1304 (s), 1198 (s), 1061 (s), 985 (s), 841 (s), 746 (vs), 682 (s), 502 (s) cm^–1^. **HRMS** (ESI) *m*/*z*: [M]^+^ calcd for C_13_H_17_N_4_S_1_O_2_, 293.1067; found, 293.1068.

##### (*Z*)-3-(Ethylthio)-1-(4-methoxybenzylidene)-5,5-dimethyl-4,5-dihydro-1*H*-1,2,4-triazol-1-ium Iodide (**6e**)

Yellow crystals (0.52 g; 49%). According to NMR the MeOH hemisolvate **6e·0.5 MeOH** was isolated. **mp** 169–171 °C (decomposition). ^**1**^**H NMR** (400 MHz, DMSO-*d*_6_): δ 10.67 (s, 1H), 8.67 (s, 1H), 8.43 (d, *J* = 9.0 Hz, 2H), 7.27 (d, *J* = 9.1 Hz, 2H), 3.92 (s, 3H), 3.31 (q, *J* = 7.3 Hz, 2H), 1.84 (s, 6H), 1.45 (t, *J* = 7.3 Hz, 3H) ppm. ^**13**^**C{**^**1**^**H} NMR** (101 MHz, DMSO-*d*_6_): δ 168.6, 164.4, 141.3, 135.9 (2C), 120.6, 115.2 (2C), 89.4, 56.1, 28.3 (2C), 25.7, 14.7 ppm. **IR (neat)** ν: 3044 (w), 2941(w), 1595 (s),1446 (s),1261 (s),1177 (s), 1014 (m), 865 (vs), 527 (m) cm^–1^. **HRMS** (ESI) *m*/*z*: [M]^+^ calcd for C_14_H_20_N_3_S_1_O_1_, 278.1322; found, 278.1328.

## Supporting Information Available

Additional calculations and spectra concerning the reaction mechanism and deprotonation experiments; complete details of DFT calculations; and full details of synthetic procedures, including complete spectral characterization (^1^H NMR, ^13^C NMR, IR, and HRMS) (PDF)
