# Supplementary material for: Mechanistic Insights into the Formation of 1-Alkylidene/Arylidene-1,2,4-triazolinium Salts: A Combined NMR/Density Functional Theory Approach
Source: J Org Chem. 2022 Jan 3;87(2):1019–31. doi: 10.1021/acs.joc.1c02327 (PMC8790756; doi:10.1021/acs.joc.1c02327)
Supplement: Supplementary file 1 — jo1c02327_si_001.pdf [file jo1c02327_si_001.pdf]

Supporting Information

Mechanistic insights into the formation of 1-alkylidene/arylidene-1,2,4-triazolinium salts: A combined NMR / DFT approach

Johann Pann[1], Kevin Erharter[2], Daniel Langerreiter[3], Gabriel Partl[1], Thomas Müller[2], Herwig Schottenberger[1], Michael Hummel[3], Thomas S. Hofer[1], Christoph Kreutz[2,*] and Lukas Fliri[1,3,*]

[1] Institute of General, Inorganic Chemistry and Theoretical Chemistry, Faculty of Chemistry and Pharmacy, University of Innsbruck, Innrain 80-82, 6020 Innsbruck, Austria.
[2] Institute of Organic Chemistry and Center for Molecular Bioscience Innsbruck (CMBI), Faculty of Chemistry and Pharmacy, University of Innsbruck, Innrain 80-82, 6020 Innsbruck, Austria.
[3] Department of Bioproducts and Biosystems, Aalto University, P.O. Box 16300, 0076 Aalto, Finland

* To whom correspondence should be addressed:

Christoph Kreutz: Email: christoph.kreutz@uibk.ac.at

Lukas Fliri: Email: lukas.fliri@aalto.fi

Table of Contents

1) Computational Details S4

2) Additional information concerning the reaction mechanism S6

2.1) DFT-Analysis of educts **1a** and **1b** to locate the most nucleophilic center S6

2.2) Additional spectra of the *in-situ* reaction monitoring experiments S7

2.3) GIAO calculated ^{13}C and ^{1}H-NMR shifts of the benzaldehyde synthon for selected **2b** intermediates S10

2.4) Calculated energy landscape of the exchange pathway in the acetone/benzaldehyde setup S10

2.5) Excluded reaction pathway focusing on symmetric aziridinium intermediates S11

2.6) Scrambling in the introduction of mixed aliphatic ketones into the reaction scheme S13

3) Deprotonation NMR-experiments using tetramethylguanidine (TMG) S14

3.1) *In-situ* formation of (*Z*)-1-benzylidene-5,5-dimethyl-3-(methylthio)-1,5-dihydro-1,2,4-triazol-1-ium-4-ide (**7b**): S14

3.2) *In-situ* formation of 1-benzyl-3-(methylthio)-5-phenyl-1*H*-1,2,4-triazole (**8c**): S16

4) Detailed experimental Procedures S17

4.1) *S*-Methyl- acetone isothiosemicarbazonium tetrafluoroborate (1a) S17

4.2) *S*-Methyl- benzaldehyde isothiosemicarbazonium tetrafluoroborate (1b) S18

4.3) *S*-Methyl- (2-^{13}*C*-acetone) isothiosemicarbazonium tetrafluoroborate (1c) S18

4.4) *S*-Ethyl- acetone isothiosemicarbazonium tetrafluoroborate (1d) S19

4.5) 5,5-Dimethyl-3-(methylthio)-1-(propan-2-ylidene)-4,5-dihydro-1*H*-1,2,4-triazol-1-ium tetrafluoroborate (2a) S19

4.6) (*Z*) 1-Benzylidene-5,5-dimethyl-3-(methylthio)-4,5-dihydro-1*H*-1,2,4-triazol-1-ium tetrafluoroborate (2b) S20

4.7) (*Z*)-1-Benzylidene-3-(methylthio)-5-phenyl-4,5-dihydro-1*H*-1,2,4-triazol-1-ium tetrafluoroborate (2c) S21

4.8) *S*-Methyl- acetoneisothiosemicarbazonium iodide (3a) S21

4.9) *S*-Methyl- butanone isothiosemicarbazonium iodide (3b) S22

4.10) *S*-Methyl- cyclohexanone isothiosemicarbazonium iodide (3c) S22

4.11) 5,5-Dimethyl-3-(methylthio)-1-(propan-2-ylidene)-4,5-dihydro-1*H*-1,2,4-triazol-1-ium iodide (4a) S23

4.12) 1-(Butan-2-ylidene)-5-ethyl-5-methyl-3-(methylthio)-4,5-dihydro-1*H*-1,2,4-triazol-1-ium iodide – mixture of *Z* – and *E* isomers (4b) S23

4.13) 1-Cyclohexylidene-3-(methylthio)-1,2,4-triazaspiro[4.5]dec-2-en-1-ium iodide (4c) S24

4.14) (*Z*)-1-Benzylidene-5,5-dimethyl-3-(methylthio)-4,5-dihydro-1*H*-1,2,4-triazol-1-ium iodide (5a) S25

4.15) (*Z*)-5,5-Dimethyl-1-(4-methylbenzylidene)-3-(methylthio)-4,5-dihydro-1*H*-1,2,4-triazol-1-ium iodide (5b) S25

4.16) (*Z*)-1-(4-Chlorobenzylidene)-5,5-dimethyl-3-(methylthio)-4,5-dihydro-1*H*-1,2,4-triazol-1-ium iodide (5c) S26

4.17) (*Z*)-5,5-Dimethyl-3-(methylthio)-1-(4-nitrobenzylidene)-4,5-dihydro-1*H*-1,2,4-triazol-1-ium iodide (5d) S26

4.18) (*Z*)-1-(4-Methoxybenzylidene)-5,5-dimethyl-3-(methylthio)-4,5-dihydro-1*H*-1,2,4-triazol-1-ium iodide (5e) S27

4.19) (*Z*)-1-Benzylidene-3-(ethylthio)-5,5-dimethyl-4,5-dihydro-1*H*-1,2,4-triazol-1-ium iodide (6a) S28

4.20) (*Z*)-3-(Ethylthio)-5,5-dimethyl-1-(4-methylbenzylidene)-4,5-dihydro-1*H*-1,2,4-triazol-1-ium iodide (6b) S28

4.21) (*Z*)-1-(4-Chlorobenzylidene)-3-(ethylthio)-5,5-dimethyl-4,5-dihydro-1*H*-1,2,4-triazol-1-ium iodide (6c) S29

4.22) (*Z*)-3-(Ethylthio)-5,5-dimethyl-1-(4-nitrobenzylidene)-4,5-dihydro-1*H*-1,2,4-triazol-1-ium iodide (6d) S30

4.23) (*Z*)-3-(ethylthio)-1-(4-methoxybenzylidene)-5,5-dimethyl-4,5-dihydro-1*H*-1,2,4-triazol-1-ium iodide (6e) S30

5) Spectral characterization S32

6) DFT Computational Data S89

7) References S105

1) Computational Details

Energy minimization: The molecule geometries were energy minimized via the DFT method implemented in the Gaussian package. The input geometry was taken from crystal structure or modelled with GaussView 6.0.16.[1] Different conformers were tested such as rotation around the C-S(CH_3) bond and the lowest energy conformer was used for further investigations. **Table S1** compares selected bond lengths of the energy minimized molecule and the structure determined by single crystal diffractometry.

Table S1: Comparison of the DFT geometry optimized (B3LYP/x2c-SVPall-s) and single crystal XRD structures **2b** and **2c**. Deviation = (d_{DFT} - d_{XRD})/ d_{XRD} *100

	**2a**			**2c**		
**Bond**	d_{XRD} /Å	d_{DFT} /Å	Deviation /%	d_{XRD} /Å	d_{DFT} /Å	Deviation /%
**N^2-C^3**	1,312	1,308	-0,30	1,321	1,312	-0,65
**C^3-C^4**	1,328	1,349	1,58	1,336	1,354	1,33
**N^1-N^2**	1,416	1,383	-2,33	1,395	1,374	-1,49
**N^1-C^8**	1,284	1,306	1,68	1,298	1,306	0,60
**N^1-C^5**	1,528	1,532	0,26	1,508	1,521	0,88
**C^3-S^6**	1,736	1,740	0,24	1,725	1,734	0,53
**N^4-C^5**	1,458	1,465	0,50	1,444	1,451	0,47

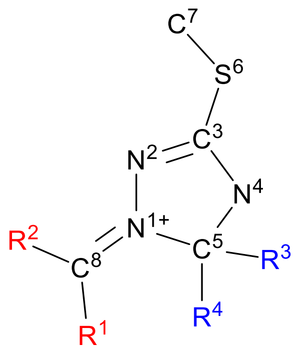

Figure S1: Structure motif with numbering as used in main manuscript.

The deviation of the calculated and refined structure is in a reasonable range. (-2.3 % to +1.5 %) Typically differences are attributed to non-symmetrical potential surfaces where the thermal ground state is nested in. Additionally, packing and explicit solvent effects are neglected in the computational model, which gives rise to an error. Hence, the model represents the chemical reality reasonably well, thus allowing helpful deductions for the understanding of the reaction mechanism.

Calculation of NMR spectra: Experimental NMR studies show the presence of an intermediate. Different intermediates were thought to be possible. Computationally these were

modelled and comparisons with the computed NMR shifts and the peaks of the observed intermediates were carried out. For charged species, like these intermediate cations, molecule solvent interactions are presumably present, therefore the proton and nitrogen NMR shifts are poorly described without the use of explicit solvents. On the other hand, computed carbon shielding tensors are more reliable in this manner[2] and readily available ^{13}C-labelled ketones and aldehydes were employed in the investigation of the reaction mechanism. Therefore, we focused mainly on the carbon NMR shift correlation.

For validation, ^{13}C NMR shifts were computed for products **2a** and **2c**. **Table S2** summarizes the gathered data.

Table S2: Comparison of the GIAO calculated (B3LYP/x2c-SVPall-s) and experimental ^{13}C NMR shifts for **2b** and **2c**. Difference = δ_{DFT} - δ_{Exp}

	**2a** in $(CD_3)_2SO$			**2c** in CD_3CN		
**Atom**	δ_{Exp} /ppm	δ_{DFT} /ppm	Difference /ppm	δ_{Exp} /ppm	δ_{DFT} /ppm	Difference /ppm
**C^3**	166.2	175.0	8.8	175.0	179.0	4.0
**C^5**	88.7	94.4	5.7	86.8	91.9	5.1
**C^8**	167.4	178.5	11.1	145.6	152.5	6.9
**C^7**	13.1	17.8	4.7	14.2	21.1	6.9
**R^{Aryl}**	–	–	–	127.9 - 136.2	133.9 - 149.6	6.0 - 13.4
**R^{Me}**	22.2 - 26.3	24.4 - 28.1	2.2 - 1.8	–	–	–

Table S2 shows good agreement of the computed and experimental values. The deviations are in the reported range for this method.[3] Better agreement was achieved with a scaling function, but for these types of molecules there are not enough data points available. Therefore, a scaling function was omitted.[4]

2) Additional information concerning the reaction mechanism

2.1) DFT-Analysis of educts **1a** and **1b** to locate the most nucleophilic center

Formally representing a condensation reaction, an attack from the isothiosemicarbazonium onto the carbonyl must happen as a first step of the scheme. Considering the product constitution, both *N*(1) and *N*(3) (**Figure S2**) represent possible active sites. Although the iminic nitrogen (*N*(3)) was already suspected as the most nucleophilic position based on the outcome of the previous experiments,[5] and because of a reported delocalization of the positive charge in similar isothiuronium cations,[6,7] we wanted to further verify our claims through DFT calculations of the educts **1a-b**. As depicted in **Figure S2**, the resulting energy minimized geometries of the modelled molecules show a trigonal planar geometry of the expected aminic *N*(1). Also, the almost equal bond lengths between *C*(2) and its neighboring atoms as well as the associated Wiberg Bond Indices, suggest a delocalization of the positive charge, thus leaving iminic *N*(3) as most nucleophilic position of the isothiosemicarbazonium structure motif.

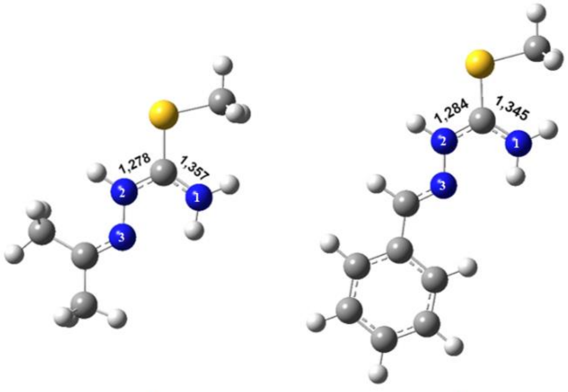

1a) 1b)

Figure S2. Energy minimized geometries of educts **1a** and **1b**. The two highlighted Wiberg Bond Indices (**1a)** 1.276 to 1.357; **1b)** 1.284 to 1.345) show that the positive charge is distributed between *N*(1) and *N*(2) of the isothiosemicarbazonium moiety (H = white, C = grey; S = yellow; N = blue).

2.2) Additional spectra of the *in-situ* reaction monitoring experiments

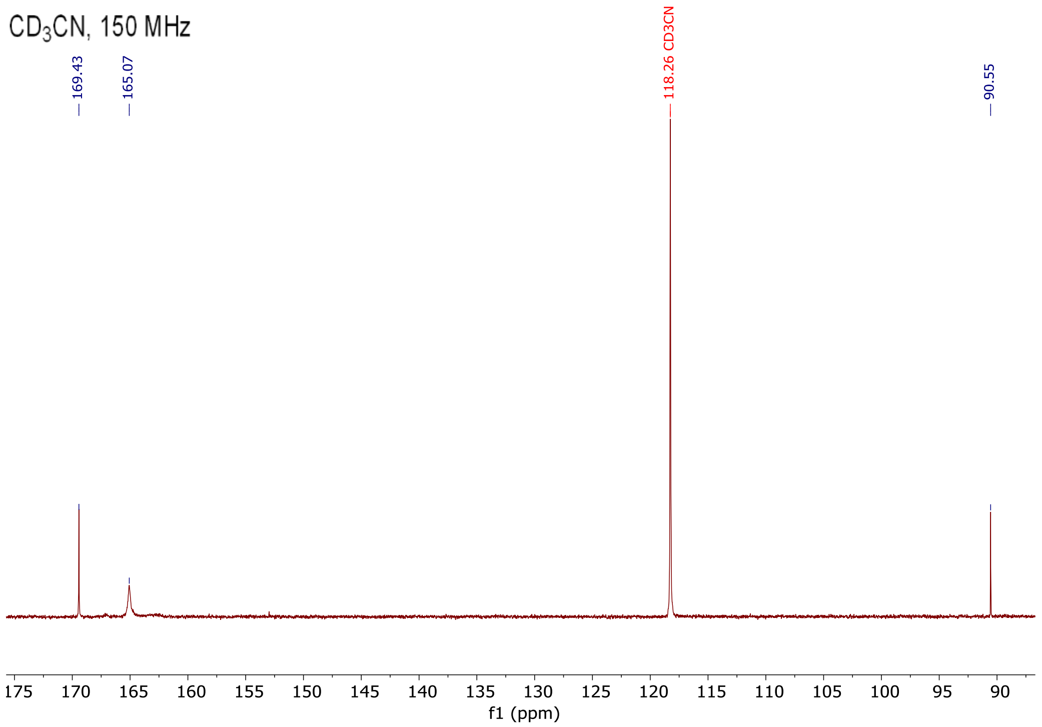

Figure S3. $^{13}C\{^1H\}$ NMR spectrum (CD_3CN, 150 MHz) of the *in-situ* reaction monitoring experiment for the acetone/acetone setup obtained after **120 min** at 50 °C. Scrambling is evidenced by incorporation of the ^{13}C labeling into both the iminium position (169.4 ppm) and the *C*(5) of the heterocycle (90.6 ppm). Metathetical carbonyl exchange is evidenced by ^{13}C incorporation into educt **1a** (165.1 ppm). Owing to the adjusted reaction conditions no full conversion was obtained.

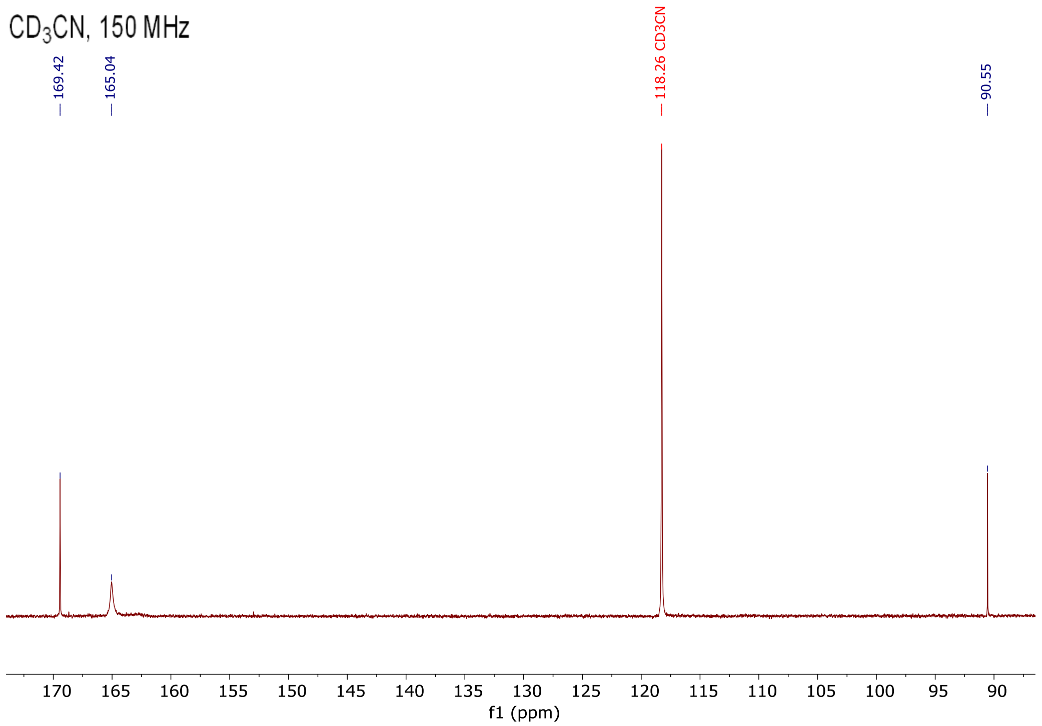

Figure S4. $^{13}C\{^1H\}$ NMR spectrum (CD_3CN, 150 MHz) of the *in-situ* reaction monitoring experiment for the acetone/acetone setup obtained after **240 min** at 50 °C. Scrambling is evidenced by incorporation of the ^{13}C labeling into both the iminium position (169.4 ppm) and the *C*(5) of the heterocycle (90.6 ppm). Metathetical carbonyl exchange is evidenced by ^{13}C incorporation into educt **1a** (165.1 ppm). Owing to the adjusted reaction conditions no full conversion was obtained.

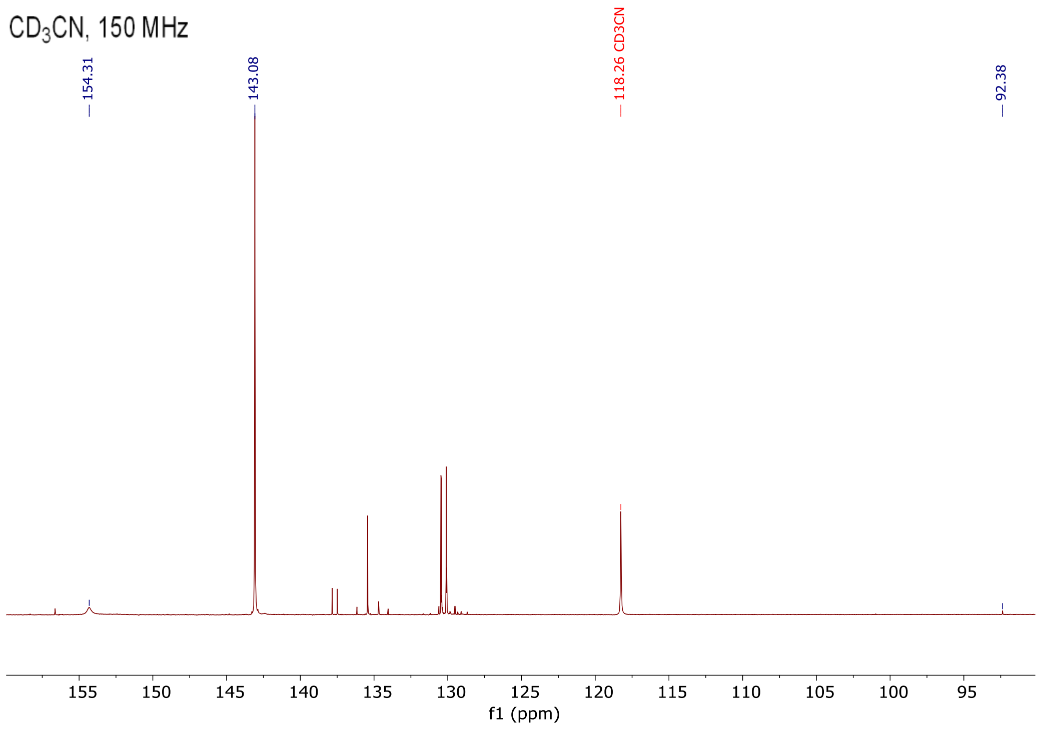

Figure S5. $^{13}C\{^1H\}$ NMR spectrum (CD_3CN, 150 MHz) of the *in-situ* reaction monitoring experiment for the acetone/benzaldehyde setup obtained after **30 min** at 50 °C. No scrambling is observed and only ^{13}C incorporation into the iminium position (143.1 ppm) occurs. Minimal metathetical carbonyl exchange is evidenced by ^{13}C incorporation into educt **1b** (154.3 ppm), however it is strongly suppressed in relation to product formation. Owing to the stabilizing factors in **2b** full conversion is obtained even under the adjusted reaction conditions

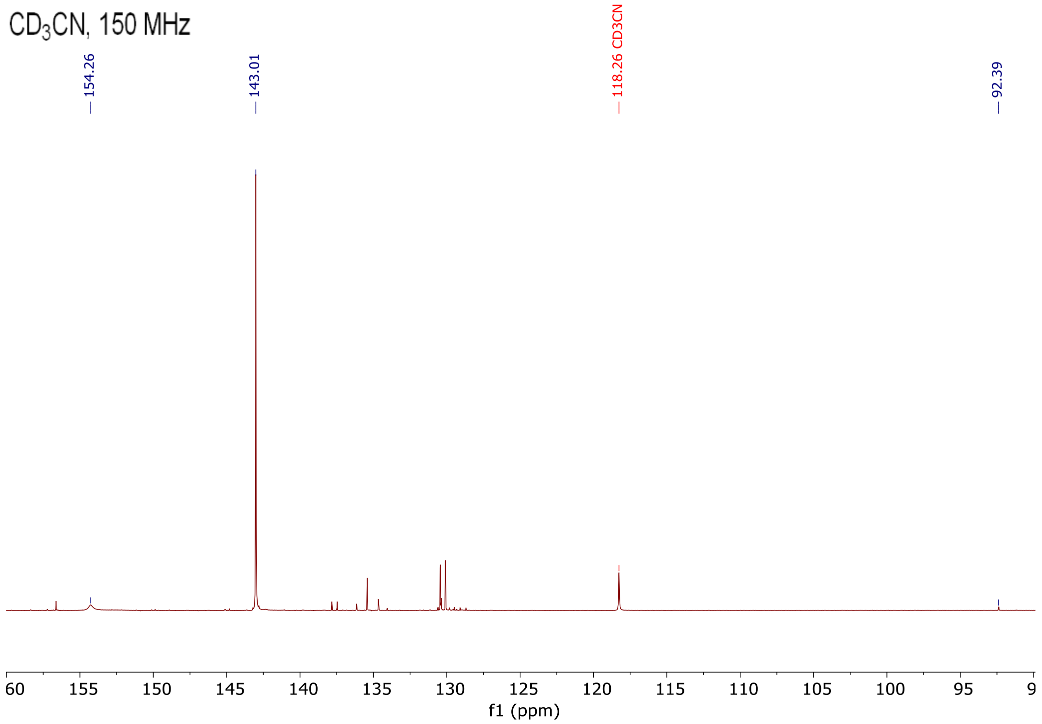

Figure S6. $^{13}C\{^1H\}$ NMR spectrum (CD_3CN, 150 MHz) of the *in-situ* reaction monitoring experiment for the acetone/benzaldehyde setup obtained after **180 min** at 50 °C. No scrambling is observed and only ^{13}C incorporation into the iminium position (143.1 ppm) occurs. Minimal metathetical carbonyl exchange is evidenced by ^{13}C incorporation into educt **1b** (154.3 ppm), however it is strongly suppressed in relation to product formation. Owing to the stabilizing factors in **2b** full conversion is obtained even under the adjusted reaction conditions

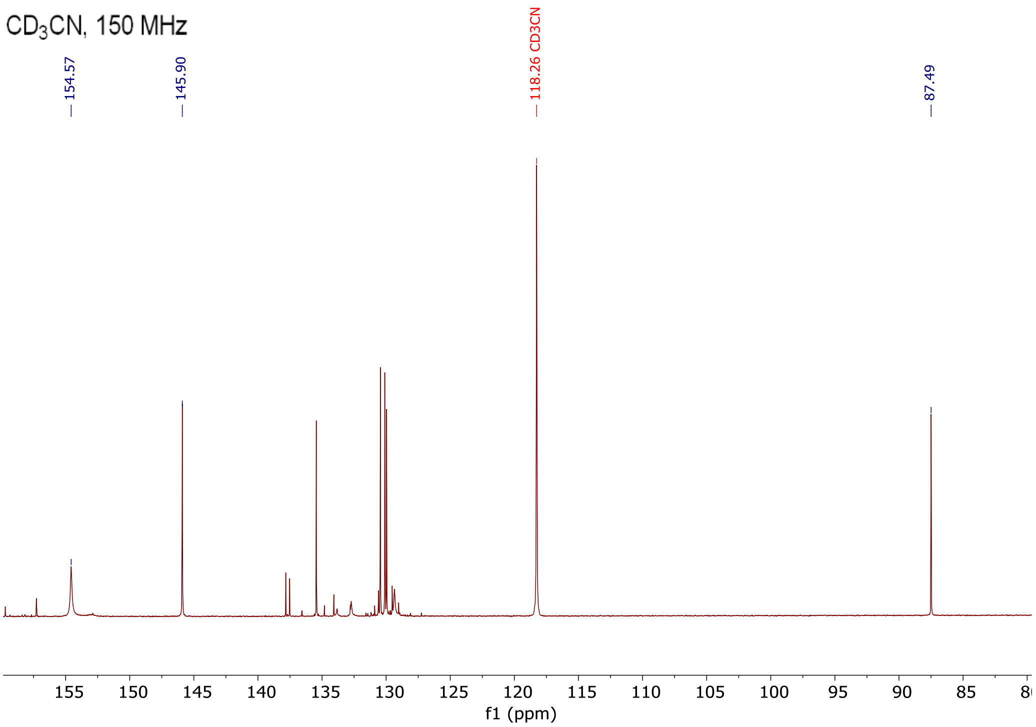

Figure S7. $^{13}C\{^{1}H\}$ NMR spectrum (CD_3CN, 150 MHz) of the *in-situ* reaction monitoring experiment for the benzaldehyde/benzaldehyde setup obtained after **120 min** at 50 °C. Scrambling is evidenced by incorporation of the ^{13}C labeling into both the iminium position (145.9 ppm) and the *C*(5) of the heterocycle (87.5 ppm). Metathetical carbonyl exchange is evidenced by ^{13}C incorporation into educt **1b** (154.6 ppm). Owing to the adjusted reaction conditions no full conversion was obtained.

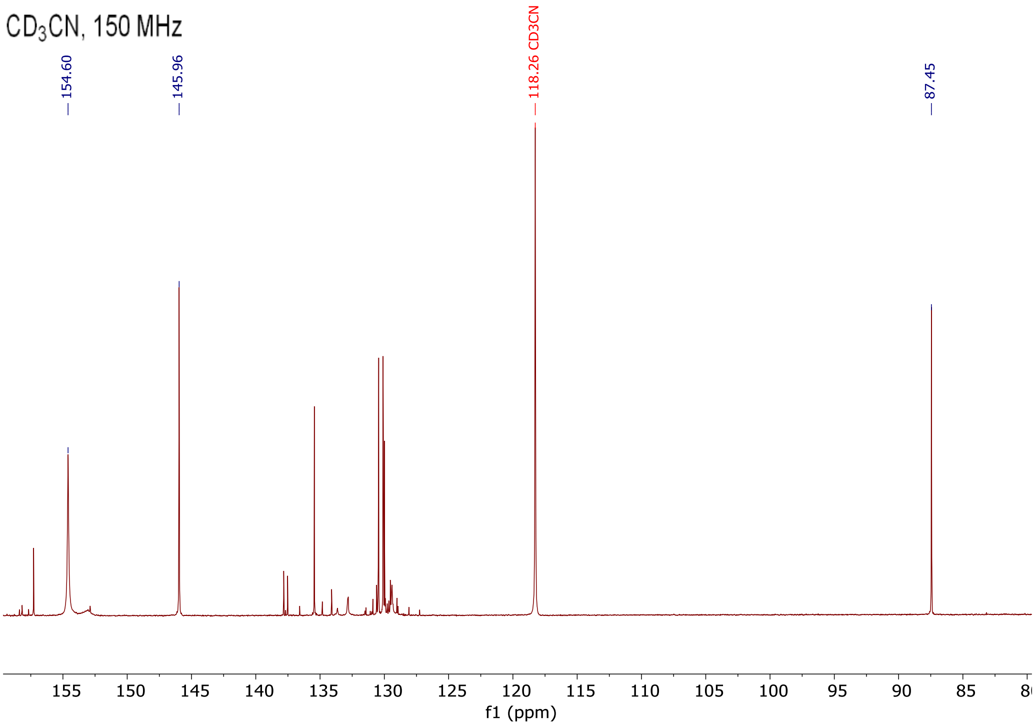

Figure S8. $^{13}C\{^{1}H\}$ NMR spectrum (CD_3CN, 150 MHz) of the *in-situ* reaction monitoring experiment for the benzaldehyde/benzaldehyde setup obtained after **240 min** at 50 °C. Scrambling is evidenced by incorporation of the ^{13}C labeling into both the iminium position (145.9 ppm) and the *C*(5) of the heterocycle (87.5 ppm). Metathetical carbonyl exchange is evidenced by ^{13}C incorporation into educt **1b** (154.6 ppm). Owing to the adjusted reaction conditions no full conversion was obtained.

2.3) GIAO calculated ^{13}C and ^{1}H-NMR shifts of the benzaldehyde synthon for selected **2b** intermediates

Table S3: GIAO calculated (B3LYP/x2c-SVPall-s) ^{13}C and ^{1}H-NMR shifts of the benzaldehyde synthon for selected 2b intermediates. Values in ppm *versus* TMS.

	**2b-ii**	**2b-iii**	**2b-iv**	**2b-v**	**2b-Az**
^{13}**C**	96.7	83.5	87.7	105.3	61.8
^{1}**H**	6.44	5.81	5.56	5.73	5.29

2.4) Calculated energy landscape of the exchange pathway in the acetone/benzaldehyde setup

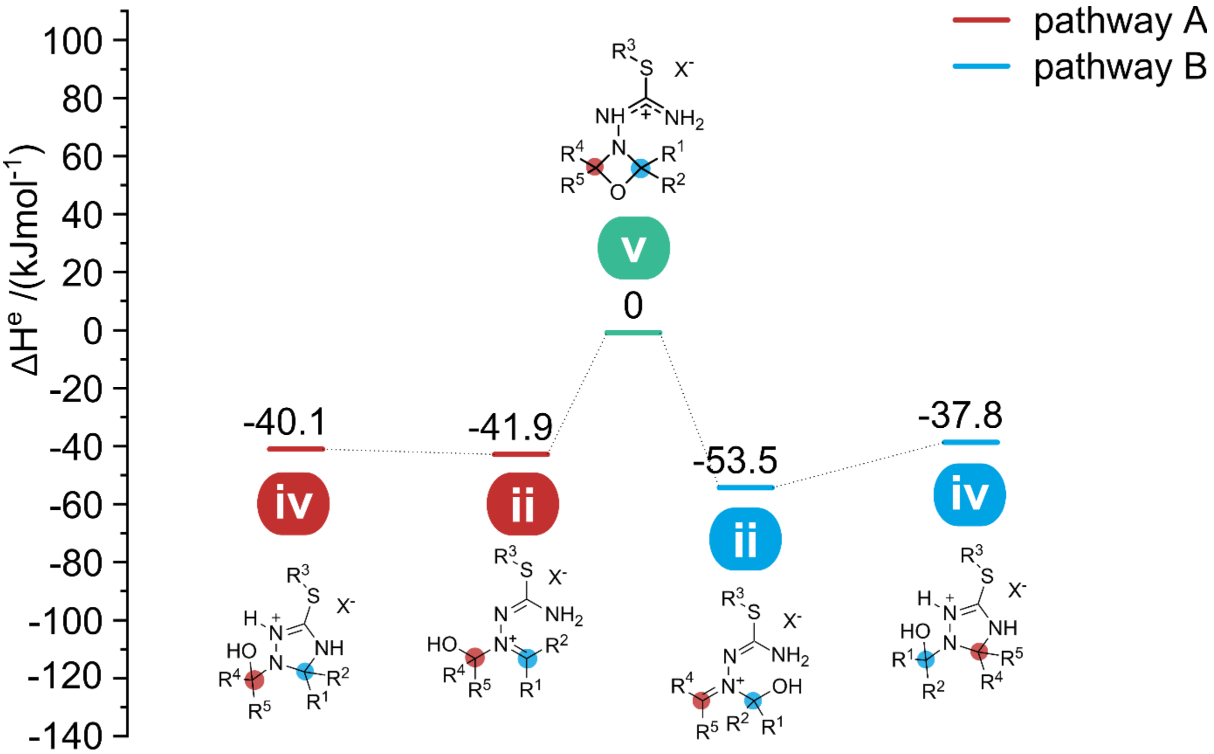

Figure S9. The energetic landscape of the exchange pathway between **2b-ii** and **2b-ii'**. Although the exchange pathway is thermodynamically favored, we could not find any NMR evidence for the scrambling product **2b'**, but only trace amounts of metathetical exchange educt **1b**. According to DFT calculations, this is a consequence of an energy barrier of +15.7 kJ mol^{-1} between **ii'** and **iv'** disfavoring a cyclization of **2b-ii'**. As a consequence, the dissociation to **1b** seems to occur predominantly.

The electronic energies of the intermediates were arbitrarily referenced to the 1,3-oxazetidine species **2b-v**. $R^1 = R^2 = R^3 = CH_3$; R^4 = phenyl; R^5 = H. B3LYP/x2c-SVPall-s.

2.5) Excluded reaction pathway focusing on symmetric aziridinium intermediates

A structure motif similar to the presented 1-alkylidene-1,2,4-triazolinium salts was so far only obtained once in a reaction of an 1-aza-2-azoniaallene salt with an aziridine congener. There, the formation of the 5-membered heterocycle was explained through a rearrangement of 3-membered intermediate structures, which were supported by AM1 calculations.[8] In the investigated reaction mechanism, the generation of 3-membered structures (**Az**) is also conceivable upon loss of H_2O of a protonated form of intermediate **ii** (**Scheme S1**). Exact knowledge of the active intermediates is fundamental to determine the scope of the reaction and to find entry points for future improvements through manipulation of the reaction conditions.

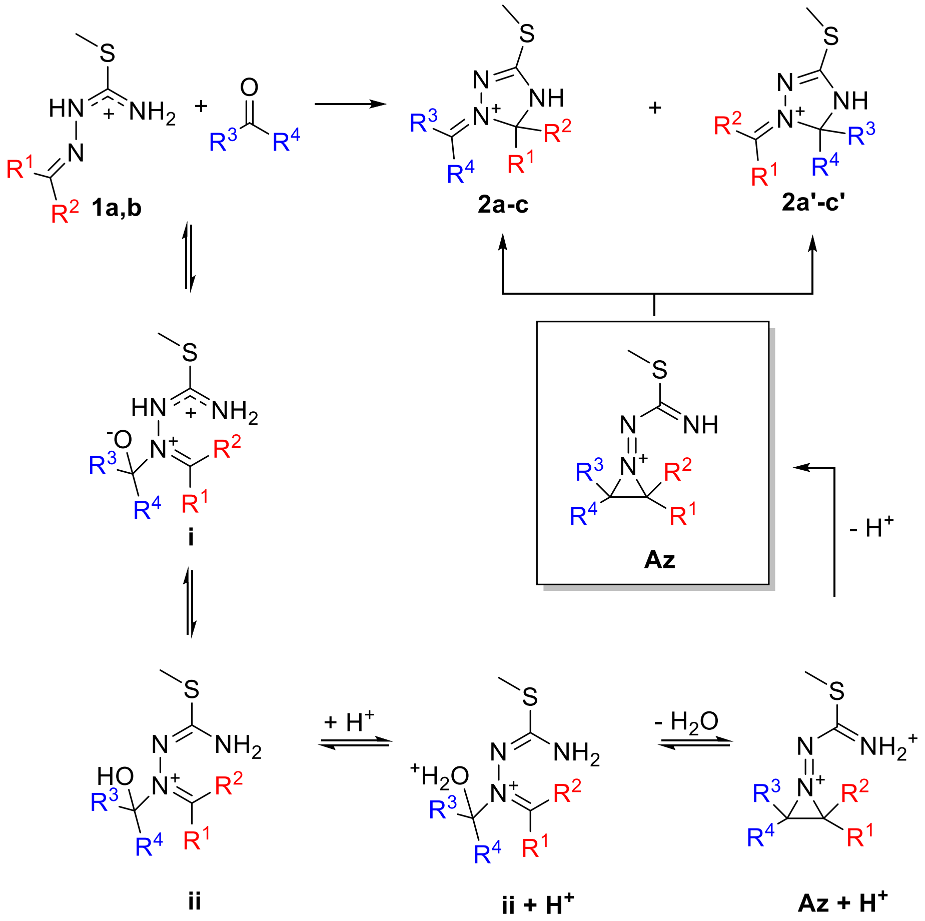

Scheme S1. Ultimately discarded mechanism for the formation of the 1-alkylidene-1,2,4-triazolinium moieties focusing on a symmetric aziridinium key intermediate. For **2a**: $R^1 = R^2 = R^3 = R^4 = CH_3$; for **2b**: $R^1 = R^2 = CH_3$, R^3 = phenyl, R^4 = H; for **2c**: $R^1 = R^4$ = phenyl, $R^2 = R^3$ = H.

The observed regio- as well as stereospecific outcome of the reaction scheme can thereby be explained by the different thermodynamic gains obtained through rearrangement of the symmetric intermediate (**Scheme S2**). However, we could ultimately discard this pathway based on three criteria: *i)* the calculated NMR shifts for **2b-Az** differed strongly from the observed intermediate peak in the acetone/benzaldehyde setup; *ii)* in an adjusted experimental setup starting from ^{13}C labeled educt **1c** and benzaldehyde *α*-^{13}C, no $^{1}J_{CC}$ coupling was observed in the NMR intermediate, which should be visible in **2b-Az**; *iii)* the calculated energy differences of -240 to -280 kJ mol^{-1} suggest a high energy intermediate, making the 3-membered ring intermediate energetically unfavored. Furthermore, a pathway including the aziridine intermediate can only explain the observed ^{13}C scrambling but would not explain the also observed metathetical carbonyl exchange.

Thus, the postulated reaction mechanism focusing on the cyclic carbinolamine and the 1,3-oxazetidine intermediates is better suited to describe the observed experimental outcome.

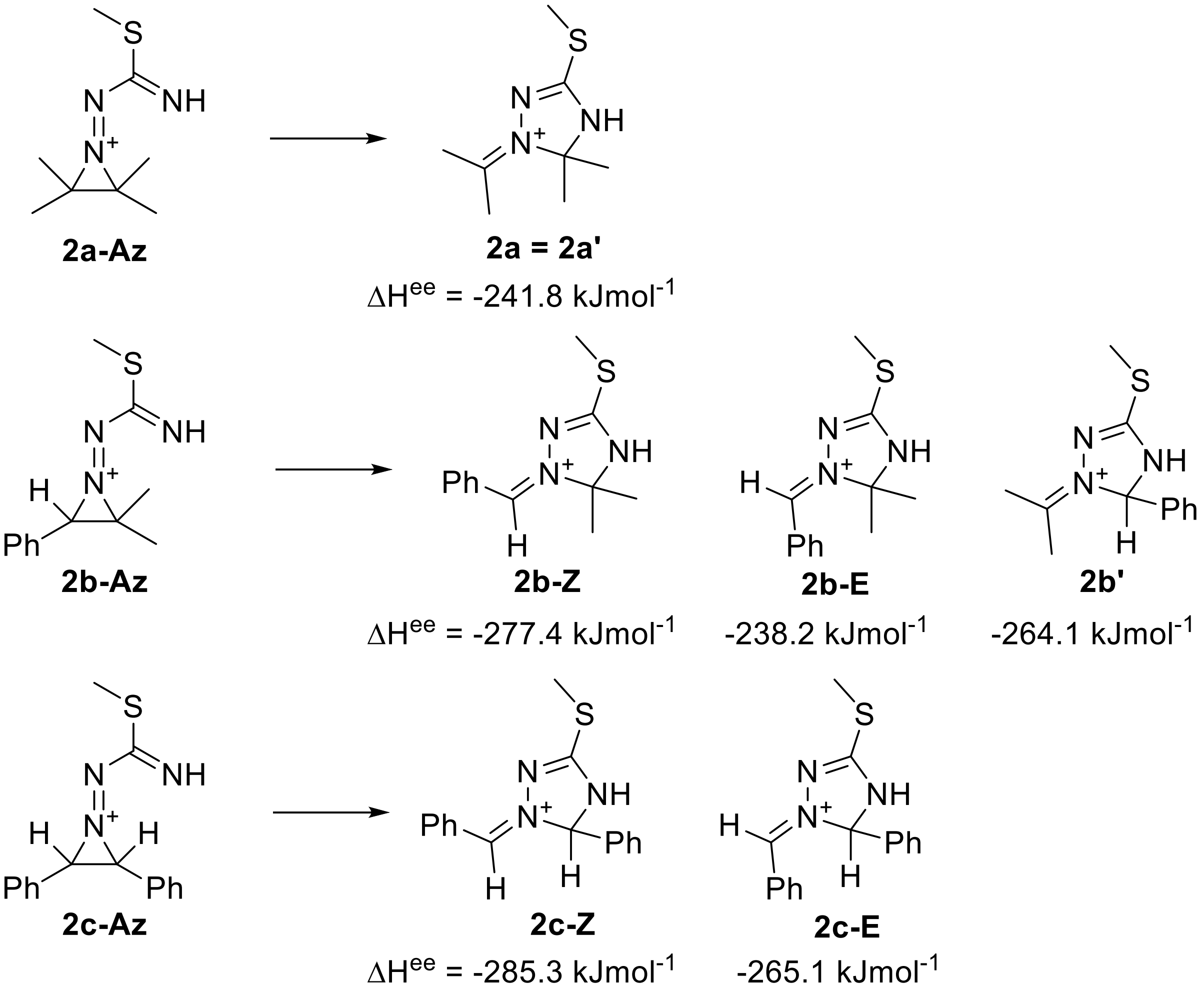

Scheme S2. Energy differences through rearrangement of conceivable symmetric 3-membered aziridine intermediates (**Az**) to 1,2,4-triazolinium products according to DFT calculations.

2.6) Scrambling in the introduction of mixed aliphatic ketones into the reaction scheme

To showcase the problem of scrambling and metathetical carbonyl exchange in the synthetic access to the presented 1,2,4-triazolinium structures, the outcome of the reaction of isothiosemicarbazonium **3a** with butanone at elevated temperatures (90 °C) is presented. Owing to the low energy barrier of the 1,3-oxazetidine intermediate the metathetical carbonyl exchange readily takes place, leading to the formation of both **3b** and acetone. As these liberated side products can also participate in the reaction scheme and undergo cyclization, a complex mixture of six different 1,2,4-triazolinium structures is formed. This is evidenced by six peaks in the *C*(5) region of the heterocycle (approximately 85-95 ppm; **Figure S10**).

Experimental setup: In a 50 mL round bottom flask *S*-methyl- acetoneisothiosemicarbazonium iodide (**3a**, 1.36 g, 5.00 mmol) and butanone (1.8 g, 25.0 mmol) were suspended in MeCN (10 mL). Thereafter 1.0 M pivalic acid – *N,N*-diisopropyl-*N*-ethylamine buffer solution (1.0 mL) was added and the mixture was refluxed at 90 °C for 16 h under stirring and protection of a drying funnel ($CaCl_2$). The vessel was cooled to -20 °C and Et_2O (5 mL) was added. After keeping the mixture at -24 °C for additional 5h a pale-yellow precipitate had formed, which was filtered off, washed with Et_2O (2 × 10 mL) and dried under reduced pressure. The obtained yellowish solid (0.56 g) was characterized by ^{13}C NMR spectroscopy.

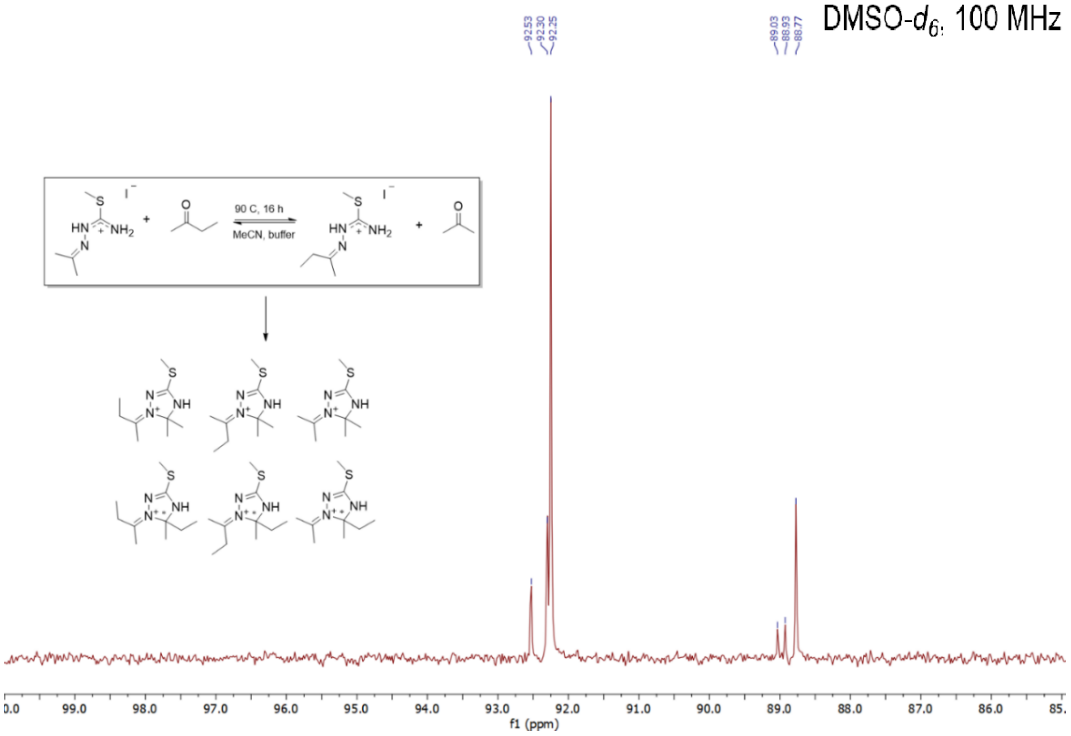

Figure S10. $^{13}C\{^1H\}$ NMR spectrum (DMSO-*d*$_6$, 100 MHz) of the crude product isolated from the reaction of **3a** with butanone. Owing to the active scrambling and metathetical carbonyl exchange pathways a complex mixture of six different products is formed, as evidenced by six peaks in the *C*(5) region of the heterocycle. Note: Owing to the chiral center on the *C*(5) also different stereoisomers for three products are likely formed, which cannot be discriminated by NMR. Thus, in total nine different products are potentially formed.

3) Deprotonation NMR-experiments using tetramethylguanidine (TMG)

3.1) *In-situ* formation of (*Z*)-1-benzylidene-5,5-dimethyl-3-(methylthio)-1,5-dihydro-1,2,4-triazol-1-ium-4-ide (**7b**):

(*Z*)-1-Benzylidene-5,5-dimethyl-3-(methylthio)-4,5-dihydro-1*H*-1,2,4-triazol-1-ium tetrafluoroborate (**2b;** 30 mg; 0.09 mmol) was dissolved in DMSO-d_6 (600 µL) in a standard 5 mm NMR tube and characterized by means of ^{1}H and ^{13}C NMR. Thereafter tetramethylguanidine (TMG; 15 µl; 0.12 mmol; 1.3 eq) was added and the solution was homogenized by shaking. Upon addition, the color of the solution visibly changed from pale yellow to orange. The generated zwitterionic structure was characterized by means of 1D ^{1}H and ^{13}C as well as 2D NMR spectroscopy. ^{1}H NMR (400 MHz, DMSO-d_6) δ = 8.23 (d, J = 7.8 Hz, 2H), 7.62 (s, 1H), 7.51 (t, J = 7.6 Hz, 2H), 7.40 (t, J = 7.4 Hz, 1H), 2.46 (s, 3H), 1.55 (s, 6H) ppm. ^{13}C{^{1}H} NMR (101 MHz, DMSO-d_6) δ = 168.5, 130.9, 129.3, 129.2 (2C), 128.6 (2C), 122.4, 101.5, 29.1 (2C), 12.7 ppm.

After incubating the NMR tube at 95 °C for 1h no differences in the NMR spectra were discernable.

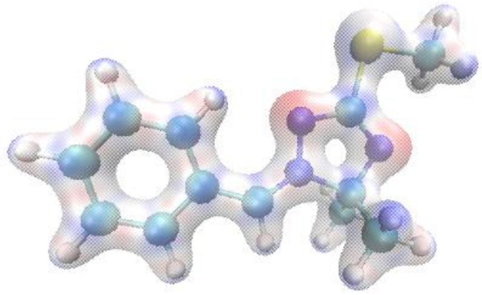

Figure S11. Charge contour plot of the mesoionic betaine obtained upon the deprotonation of **2b** with TMG. A localization of the negative charge on N2 and N4 is depicted, as well as a more positive partial charge located at N1.

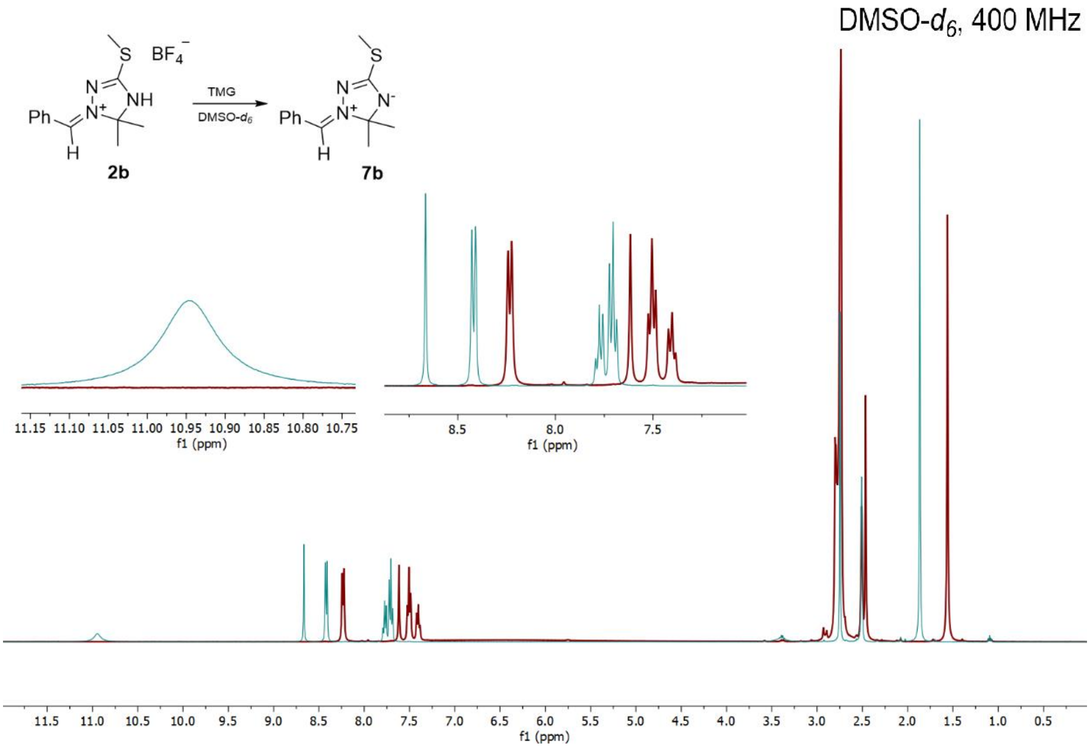

Figure S12. Comparison of the ^{1}H-NMR spectra (DMSO-d_6, 400 MHz) of (*Z*) 1-benzylidene-5,5-dimethyl-3-(methylthio)-4,5-dihydro-1*H*-1,2,4-triazol-1-ium tetrafluoroborate (**2b**) before (background) and after (red – in front) addition of tetramethylguanidine (TMG) leading to the formation of (*Z*)-1-benzylidene-5,5-dimethyl-3-(methylthio)-1,5-dihydro-1,2,4-triazol-1-ium-4-ide (**7b**).

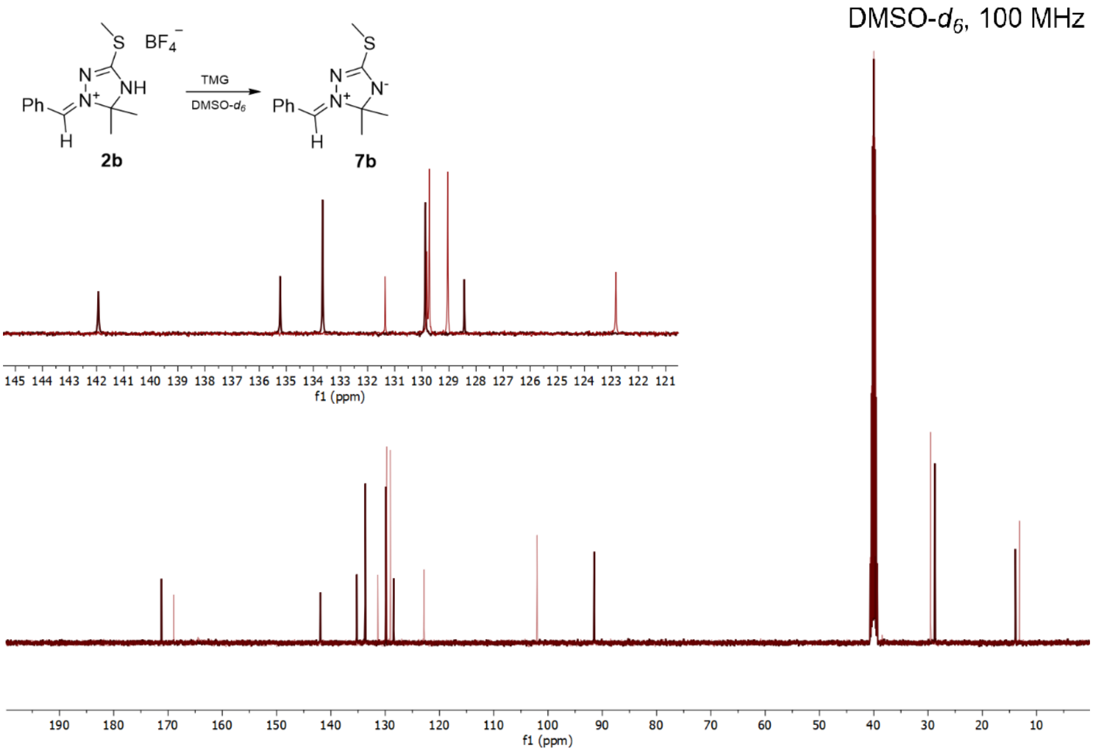

Figure S13. Comparison of the $^{13}C\{^1H\}$ NMR spectra (DMSO-d_6, 100 MHz) of (*Z*) 1-benzylidene-5,5-dimethyl-3-(methylthio)-4,5-dihydro-1*H*-1,2,4-triazol-1-ium tetrafluoroborate (**2b**) before (background) and after (red – in front) addition of tetramethylguanidine (TMG) leading to the formation of (*Z*)-1-benzylidene-5,5-dimethyl-3-(methylthio)-1,5-dihydro-1,2,4-triazol-1-ium-4-ide (**7b**).

3.2) *In-situ* formation of 1-benzyl-3-(methylthio)-5-phenyl-1*H*-1,2,4-triazole (**8c**):

(*Z*)-1-Benzylidene-3-(methylthio)-5-phenyl-4,5-dihydro-1*H*-1,2,4-triazol-1-ium tetrafluoroborate (**2c;** 35 mg; 0.095 mmol) was dissolved in DMSO-d_6 (600 µL) in a standard 5 mm NMR tube and characterized by means of ^{1}H and ^{13}C NMR. Thereafter tetramethylguanidine (TMG; 25 µl; 0.20 mmol; 2.1 eq) was added and the solution was homogenized by shaking. Upon addition, the color of the solution visibly changed from pale yellow to dark orange. The generated product of the hydride shift reaction was characterized by means of 1D ^{1}H and ^{13}C as well as 2D NMR spectroscopy. ^{1}H NMR (400 MHz, DMSO-d_6) δ = 7.66 – 7.62 (m, 2H), 7.55 – 7.49 (m, 3H), 7.36 – 7.27 (m, 3H), 7.13 – 7.09 (m, 2H), 5.45 (s, 2H), 2.56 (s, 3H) ppm. ^{13}C{^{1}H] NMR (101 MHz, DMSO-d_6) δ = 159.8, 155.6, 136.2, 130.4, 129.0 (2C), 128.8 (2C), 128.4(2C), 127.8, 127.2, 126.8 (2C), 52.3, 13.8 ppm.

Compound **8c** was isolated and characterized by NMR spectroscopy following a previously reported protocol: Literature:[9] ^{1}H NMR (CDCl$_3$) δ = 7.57 – 7.17 (m, 10H; aromatic), 5.36 (s, 2H, CH$_2$), 2.64 (s, 3H, Me) ppm. ^{13}C{^{1}H] NMR (CDCl$_3$; only given for *C*(3) and *C*(5) of 1,2,4-triazole heterocycle) δ = 161.1 (*C*(3)) and 156.3 (*C*(5)) ppm. **Found:** ^{13}C NMR (101 MHz, DMSO-d_6) δ = 159.8 (*C*(3)) and 155.6 (*C*(5)) ppm.

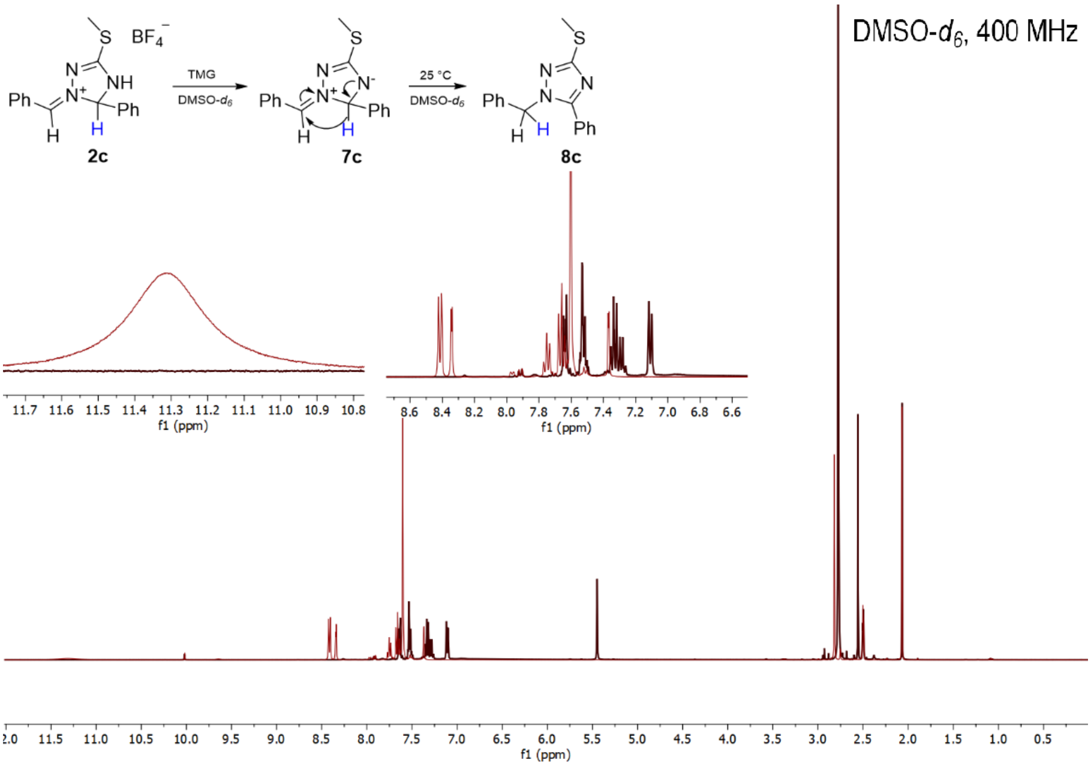

Figure S14. Comparison of the ^{1}H-NMR spectra (DMSO-d_6, 400 MHz) of (*Z*) -1-benzylidene-3-(methylthio)-5-phenyl-4,5-dihydro-1*H*-1,2,4-triazol-1-ium tetrafluoroborate (**2c**) before (background) and after (red – in front) addition of tetramethylguanidine (TMG) leading to the formation of 1-benzyl-3-(methylthio)-5-phenyl-1*H*-1,2,4-triazole (**8c**). Small amounts of benzaldehyde are present before the addition of TMG owing to H_2O contamination of the used DMSO-d_6.

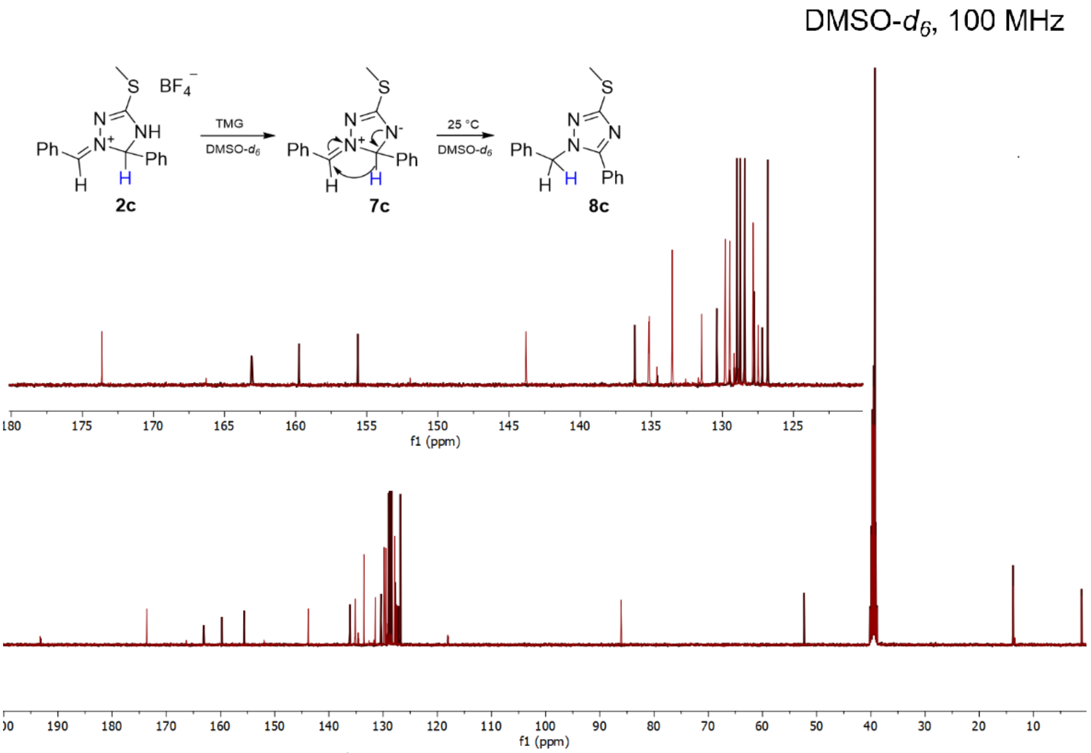

Figure S15. Comparison of the $^{13}C\{^{1}H\}$ NMR spectra (DMSO-d_6, 100 MHz) of (*Z*) -1-benzylidene-3-(methylthio)-5-phenyl-4,5-dihydro-1*H*-1,2,4-triazol-1-ium tetrafluoroborate (**2c**) before (background) and after (red – in front) addition of tetramethylguanidine (TMG) leading to the formation of 1-benzyl-3-(methylthio)-5-phenyl-1*H*-1,2,4-triazole (**8c**). Small amounts of benzaldehyde are present before the addition of TMG owing to H_2O contamination of the used DMSO-d_6.

4) Detailed experimental Procedures

4.1) *S*-Methyl- acetone isothiosemicarbazonium tetrafluoroborate (1a)

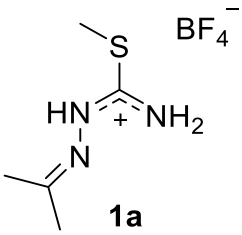

1a

Acetonethiosemicarbazone (15.0 mmol, 1.97 g) and trimethyloxonium tetrafluoroborate (16.5 mmol, 2.44 g, 1.05 eq.) were dissolved in CH_2Cl_2 (12 mL) and stirred for 72 h at room temperature. A colorless suspension formed, which was treated with MeOH (15 mL) and stirred for an additional 1 h. The now homogenous solution was thereafter stripped off of CH_2Cl_2 by means of a rotary evaporator and the resulting methanolic solution treated with Et_2O (70 mL), leading to the separation of a slightly yellow oil, which solidified after keeping the mixture at -32 °C for 16 h. The precipitate was filtered off, washed with Et_2O (20 mL) and dried under reduced pressure, yielding 3.4 g (97 %) of a white, crystalline solid. **Mp** = 101 – 103 °C. **^{1}H NMR** (400 MHz, acetonitrile-d_3) δ = 9.90 (s, 1H), 8.33 (s, 1H), 7.69 (s, 1H), 2.67 (s, 3H), 2.11 (s, 3H), 2.03 (s, 3H) ppm. **$^{13}C\{^{1}H\}$ NMR** (101 MHz, acetonitrile-d_3) δ = 168.94, 165.14, 25.26, 18.65, 13.69 ppm. **IR (neat):** v = 3388 (w), 3295 (w), 3226 (w), 1633 (m), 1569 (m), 1498 (w),

1439 (m), 1377 (w), 1274 (w), 1013 (vs), 860 (w), 768 (w), 643 (m), 592 (m), 520 (w), 422 (w) cm^{-1}. **HRMS** (ESI) *m/z*: [M]$^+$ Calcd for $C_5H_{12}N_3S_1$ 146.0746; Found 146.0738.

4.2) *S*-Methyl- benzaldehyde isothiosemicarbazonium tetrafluoroborate (1b)

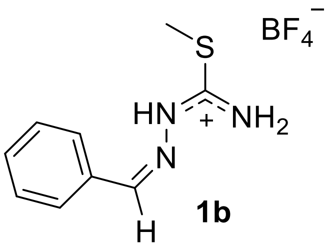

1b

Benzaldehydethiosemicarbazone (5.0 mmol, 0.9 g) and trimethyloxonium tetrafluoroborate (5.5 mmol, 0.81 g, 1.1 eq.) were dissolved in CH_2Cl_2 (20 mL) and stirred for 24 h at room temperature. A colorless solution formed, which was treated with MeOH (10 mL) and stirred for additional 3 h. The solution was thereafter stripped off of CH_2Cl_2 by means of a rotary evaporator and the resulting methanolic phase treated with Et_2O (30 mL) and kept at -32 °C for 16 h. The generated precipitate was filtered off, washed with Et_2O (20 mL) and dried under reduced pressure, yielding 1.40 g (99 %) of a white, crystalline solid. **Mp** = 111 – 113 °C. **^{1}H NMR** (400 MHz, acetonitrile-*d*$_3$) δ = 8.32 (s, 1H), 7.91 – 7.86 (m, 2H), 7.60 – 7.48 (m, 3H), 2.72 (s, 3H) ppm. **^{13}C{^{1}H} NMR** (101 MHz, acetonitrile-*d*$_3$) δ = 168.7, 154.2, 133.0 (2C), 129.90 (2C), 129.3 (2C), 13.8 ppm. **IR (neat):** v = 3614 (w), 3540 (w), 3410 (w), 3316 (w), 3246 (w), 2998 (w), 2931 (w), 2854 (w), 1639 (s), 1613 (s), 1593 (m), 1450 (w), 1383 (m), 1335 (w), 1305 (m), 1232 (w), 1019 (vs), 964 (s), 871 (w), 800 (w), 763 (m), 696 (m), 664 (m), 508 (m), 416 (w) cm^{-1}. **HRMS** (ESI) *m/z*: [M]$^+$ Calcd for $C_9H_{12}N_3S_1$ 194.0746; Found 194.0735.

4.3) *S*-Methyl- (2-^{13}*C*-acetone) isothiosemicarbazonium tetrafluoroborate (1c)

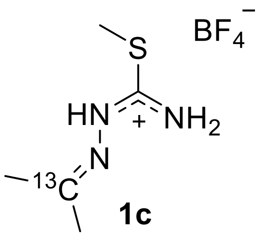

1c

2-^{13}C-Acetonethiosemicarbazone (2.65 mmol, 0.35 g) and trimethyloxonium tetrafluoroborate (2.91 mmol, 0.43 g, 1.1 eq.) were dissolved in CH_2Cl_2 (10 mL) and stirred for 24 h at room temperature. A colorless solution formed, which was treated with MeOH (10 mL) and stirred for additional 2 h. The solution was thereafter stripped off of CH_2Cl_2 by means of a rotary evaporator and the resulting methanolic phase treated with Et_2O (80 mL) and kept at -32 °C for 16 h. The generated precipitate was filtered off, washed with Et_2O (40 mL) and dried under reduced pressure, yielding 0.47 g (76 %) of a white, crystalline solid. **Mp** = 113 – 115 °C. **^{1}H NMR** (400 MHz, acetonitrile-*d*$_3$) δ = 9.92 (s, 1H), 7.98 (s, 2H), 2.67 (s, 3H), 2.11 (d, *J*=6.9 Hz,3H), 2.03 (d, *J*=6.1 Hz, 3H) ppm. **^{13}C{^{1}H} NMR** (101 MHz, acetonitrile-*d*$_3$) δ = 169.0 (d, *J* =5.8 Hz), 165.2, 25.3 (d, *J* =48.1 Hz), 18.8 (d, *J* =38.4 Hz), 13.9 ppm. **IR (neat):** v = 3387 (w), 3299 (m), 3251 (m), 3142 (m), 3090 (m), 2997 (w), 2944 (m), 2928 (m), 2853 (w),

1628 (s), 1562 (s), 1498 (m), 1441 (s), 1380 (m), 1318 (m), 1282 (m), 1245 (m), 1221 (w), 1107 (s), 1086 (s), 1018 (vs), 995 (s), 962 (s), 857 (m), 769 (w), 729 (m), 701 (m), 666 (s), 592 (m), 523 (m), 506 (m), 444 (m), 408 (w) cm^{-1}. **HRMS** (ESI) *m/z*: $[M]^+$ Calcd for $^{13}C_1C_4H_{12}N_3S_1$ 147.0780; Found 147.0771.

4.4) *S*-Ethyl- acetone isothiosemicarbazonium tetrafluoroborate (1d)

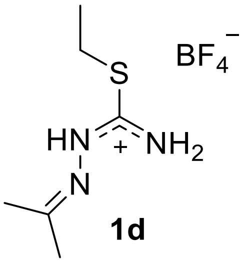

1d

Acetonethiosemicarbazone (47.6 mmol, 6.2 g) and triethyloxonium tetrafluoroborate (50.0 mmol, 9.5 g, 1.05 eq.) were suspended in CH_2Cl_2 (70 mL) and stirred for 24 h at room temperature. A colorless suspension formed, which was treated with EtOH (25 mL) and stirred for an additional 4 h. The now homogenous, colorless solution was thereafter stripped from CH_2Cl_2 by means of a rotary evaporator and the resulting ethanolic solution treated with Et_2O (220 mL). After keeping the mixture at -24 °C for 96 h a white precipitate formed, which was filtered off, washed with Et_2O (30 mL) and dried under reduced pressure, yielding 9.9 g (84 %) of a white, crystalline solid. **Mp** = 74 – 76 °C. **^{1}H NMR** (400 MHz, DMSO-d_6) δ = 11.76 (s, 1H), 9.50 – 8.58 (m, 2H), 3.25 (q, J = 7.3 Hz, 2H), 2.05 (d, J = 22.8 Hz, 5H), 1.29 (t, J = 7.3 Hz, 3H) ppm. **^{13}C{^{1}H} NMR** (101 MHz, DMSO-d_6) δ = 165.9, 164.97, 25.6, 25.3, 19.3, 14.6 ppm. **IR (neat):** v = 3388 (w), 3298 (w), 3235 (w), 1626 (m), 1574 (m), 1436 (w), 1272 (w), 1015 (vs), 638 (w) cm^{-1}. **HRMS** (ESI) *m/z*: $[M]^+$ Calcd for $C_6H_{14}N_3S_1$ 160.0903; Found 160.0920.

4.5) 5,5-Dimethyl-3-(methylthio)-1-(propan-2-ylidene)-4,5-dihydro-1*H*-1,2,4-triazol-1-ium tetrafluoroborate (2a)

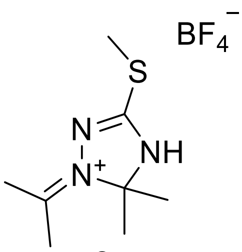

2a

5,5-Dimethyl-3-(methylthio)-1-(propan-2-ylidene)-4,5-dihydro-1*H*-1,2,4-triazol-1-ium iodide (**4a**, 1.0 mmol, 0.35 g) was dissolved in MeOH (5 mL) and combined with a solution of $AgBF_4$ (1.05 mmol, 0.20 g 1.05 eq.) in MeOH (2 mL). After the addition of MeCN (5 mL), the resulting suspension was ultrasonicated by means of an ultrasonication bath (10 min), then the yellow precipitate (AgI) filtered off and washed with MeCN (10 mL). The filtrate was concentrated by means of a rotary evaporator to about 5 mL and thereafter treated with Et_2O (50 mL). The mixture was kept at -32 °C for 24 h, during which time the product precipitated. The solid was filtered off, washed with Et_2O (10 mL) and dried under reduced pressure, yielding 0.22 g (80 %) of a white powder. **Mp** = 140 – 142 °C. **^{1}H NMR** (400 MHz,

acetonitrile-d_3) δ = 7.78 (s, 1H), 2.58 (s, 3H), 2.55 (s, 3H), 2.46 (s, 3H), 1.90 (s, 6H) ppm. **^{1}H NMR** (400 MHz, DMSO-d_6) δ = 9.97 (s, 1H), 2.57 (s, 6H), 2.43 (s, 3H), 1.84 (s, 6H) ppm. **^{13}C{^{1}H} NMR** (101 MHz, acetonitrile-d_3) δ = 169.2, 168.4, 90.3, 26.8 (2C), 25.6, 22.5, 13.8 ppm. **^{13}C{^{1}H} NMR** (101 MHz, DMSO-d_6) δ = 167.6, 166.4, 88.9, 26.2 (2C), 25.0, 21.8, 12.9 ppm. **IR (neat):** v = 3317 (w), 1646 (w), 1511 (s), 1486 (m), 1454 (m), 1432 (m), 1400 (m), 1383 (w), 1300 (w), 1208 (w), 1063 (vs), 1037 (s), 1013 (s), 985 (s), 967 (s), 892 (w), 872 (w), 769 (w), 715 (w), 678 (w), 561 (w), 523 (m), 477 (w) cm^{-1}. **HRMS** (ESI) *m/z*: [M]$^+$ Calcd for $C_8H_{16}N_3S_1$ 186.1059; Found 186.1049.

4.6) (*Z*) 1-Benzylidene-5,5-dimethyl-3-(methylthio)-4,5-dihydro-1*H*-1,2,4-triazol-1-ium tetrafluoroborate (2b)

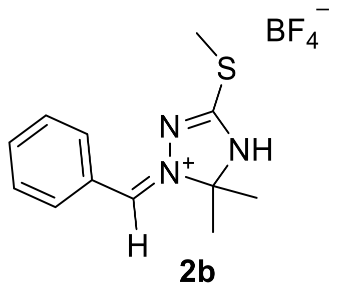

2b

(*Z*) 1-Benzylidene-5,5-dimethyl-3-(methylthio)-4,5-dihydro-1*H*-1,2,4-triazol-1-ium iodide[21] 0.83 mmol, 0.30 g) was dissolved in MeOH (5 mL) and combined with a solution of $AgBF_4$ (0.87 mmol, 0.17 g, 1.05 eq.) in MeOH (2 mL). After the addition of MeCN (3 mL), the resulting suspension was ultrasonicated by means of an ultrasonication bath (10 min), then the yellow precipitate (AgI) filtered off and washed with MeCN (10 mL). The filtrate was concentrated by means of a rotary evaporator to about 5 mL and thereafter treated with Et_2O (50 mL). The mixture was kept at -32 °C for 24 h, during which time the product precipitated. The solid was filtered off, washed with Et_2O (10 mL) and dried under reduced pressure, yielding 0.21 g (79 %) of a slightly yellow powder. **Mp** = 176 – 178 °C. **^{1}H NMR** (400 MHz, acetonitrile-d_3) δ = 8.48 – 8.42 (m, 2H), 8.11 (s, 1H), 7.81 – 7.75 (m, 1H), 7.73 – 7.66 (m, 2H), 2.75 (s, 3H), 1.90 (s, 6H) ppm. **^{1}H NMR** (400 MHz, DMSO-d_6) δ = 10.95 (s, 1H), 8.67 (s, 1H), 8.45 – 8.39 (m, 2H), 7.81 – 7.66 (m, 3H), 2.75 (s, 3H), 1.87 (s, 6H) ppm. **^{13}C{^{1}H} NMR** (101 MHz, acetonitrile-d_3) δ = 172.6, 143.1, 136.1, 134.5 (2C), 130.3, 128.8 (2C), 92.1, 28.8 (2C), 14.3 ppm. **^{13}C{^{1}H} NMR** (101 MHz, DMSO-d_6) δ = 170.7, 141.5, 134.8, 133.2 (2C), 129.4 (2C), 128.0, 91.0, 28.3 (2C), 13.4 ppm. **IR (neat):** v = 3278 (w), 1630 (w), 1593 (w), 1484 (s), 1451 (s), 1408 (m), 1389 (m), 1328 (w), 1303 (w), 1283 (w), 1226 (w), 1200 (w), 1130 (w), 1053 (s), 994 (vs), 944 (s), 911 (m), 876 (w), 831 (m), 764 (s), 686 (s), 606 (w), 542 (m), 524 (m), 504 (m), 484 (m), 432 (w) cm^{-1}. **HRMS** (ESI) *m/z*: [M]$^+$ Calcd for $C_{12}H_{16}N_3S_1$ 234.1059; Found 234.1045.

4.7) (*Z*)-1-Benzylidene-3-(methylthio)-5-phenyl-4,5-dihydro-1*H*-1,2,4-triazol-1-ium tetrafluoroborate (2c)

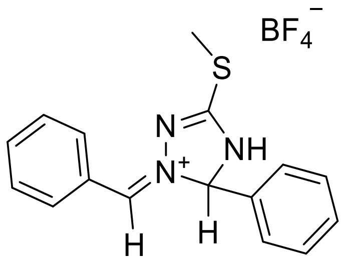

2c

(*Z*)-1-Benzylidene-3-(methylthio)-5-phenyl-4,5-dihydro-1*H*-1,2,4-triazol-1-ium iodide[21] (1.0 mmol, 0.41 g) was dissolved in MeOH (5 mL) and combined with a solution of $AgBF_4$ (1.05 mmol, 0.20 g 1.05 eq.) in MeOH (2 mL). After the addition of MeCN (5 mL), the resulting suspension was ultrasonicated by means of an ultrasonication bath (10 min), then the yellow precipitate (AgI) filtered off and washed with MeCN (10 mL). The filtrate was concentrated by means of a rotary evaporator to about 5 mL and thereafter treated with Et_2O (50 mL). The mixture was kept at -32 °C for 24 h, whereas the product precipitated. The solid was filtered off, washed with Et_2O (10 mL) and dried under reduced pressure, yielding 0.30 g (81 %) of a slightly yellow crystalline solid. According to NMR data, the MeCN monosolvate **2c · MeCN** was isolated. **Mp** = 169 – 171 °C. **^{1}H NMR** (400 MHz, acetonitrile-*d*$_3$) *δ* = 8.47 (s, 1H), 8.37 – 8.32 (m, 2H), 7.86 (d, *J*=2.5 Hz, 1H), 7.79 – 7.74 (m, 1H), 7.68 – 7.59 (m, 7H), 7.04 (d, *J*=2.5 Hz, 1H), 2.83 (s, 3H) ppm. **^{1}H NMR** (400 MHz, DMSO-*d*$_6$ – slight dissociation) *δ* = 11.32 (s, 1H), 8.45 – 8.40 (m, 2H), 8.35 (d, *J*=2.5 Hz, 1H), 7.76 (t, *J*=7.5 Hz, 1H), 7.70 – 7.64 (m, 2H), 7.61 (s, 5H), 7.38 (d, *J*=2.5 Hz, 1H), 2.83 (s, 3H) ppm. **^{13}C{^{1}H} NMR** (101 MHz, acetonitrile-*d*$_3$) *δ* = 175.4, 145.9, 136.5, 135.1, 134.7 (2C), 132.7, 130.8, 130.4 (2C), 129.1 (2C), 128.2, 118.3, 87.2, 14.5 ppm. **^{13}C{^{1}H} NMR** (101 MHz, DMSO-*d*$_6$ – slight dissociation) *δ* = 173.6, 143.8, 135.2, 135.1, 133.5 (2C), 131.5, 129.8 (2C), 129.5 (2C), 127.8 (2C), 127.5, 86.1, 13.7 ppm. **IR (neat):** *v* = 3278 (w), 1630 (w), 1593 (w), 1484 (s), 1451 (s), 1408 (m), 1389 (m), 1328 (w), 1303 (w), 1283 (w), 1226 (w), 1200 (w), 1130 (w), 1053 (s), 994 (vs), 944 (s), 911 (m), 876 (w), 831 (m), 764 (s), 686 (s), 606 (w), 542 (m), 524 (m), 504 (m), 484 (m), 432 (w) cm^{-1}. **HRMS** (ESI) *m/z*: $[M]^+$ Calcd for $C_{16}H_{16}N_3S_1$ 282.1059; Found 282.1042.

4.8) *S*-Methyl- acetoneisothiosemicarbazonium iodide (3a)

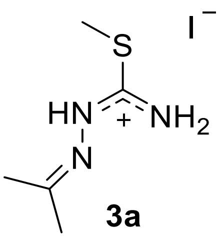

3a

Acetonethiosemicarbazone (9.84 g, 75 mmol) and iodomethane (11.2 g, 78.8 mmol) were dissolved in MeCN (35.0 mL) and MeOH (2.00 mL). The mixture was refluxed for 18 h under stirring. After cooling to room temperature, Et_2O (100 mL) was added and the suspension was kept at -32 °C for 16 h. The resulting white precipitate was filtered off, washed with Et_2O (2 × 20 mL) and dried under reduced

pressure to yield 20.1 g (98%) of a white solid. **Mp** = 174 – 176 °C. **^{1}H NMR** (400 MHz, DMSO-*d*$_6$) *δ* = 11.72 (s, 1H), 9.25 (s, 2H), 2.67 (s, 3H), 2.09 (s, 3H), 2.03 (s, 3H) ppm. **^{13}C{^{1}H} NMR** (75 MHz, DMSO-*d*$_6$) *δ* 166.1, 164.55, 25.0, 18.9, 13.5 ppm. **IR (neat):** *v* = 3223 (m), 3168 (m), 3078 (s), 2970 (m), 1653 (w), 1613 (vs), 1559 (vs), 1491 (m), 1432 (s), 1372 (m), 1321 (m), 1272 (s), 1225 (m), 1108 (m), 1082 (m), 1021 (m), 984 (m), 861 (m), 770 (s), 651 (vs), 593 (m), 509 (m), 417 (m) cm^{-1}. **HRMS** (ESI) *m/z*: [M]$^+$ Calcd for $C_5H_{12}N_3S_1$ 146.0746; Found 146.0738.

4.9) *S*-Methyl- butanone isothiosemicarbazonium iodide (3b)

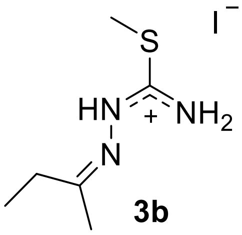

3b

Butanonethiosemicarbazone (75.0 mmol, 10.9 g) and iodomethane (78.8 mmol, 11.2 g, 1.05 eq.) were dispersed in a mixture of MeCN (35 mL) and MeOH (5 mL). The suspension was refluxed at 90 °C for 1 h. The resulting yellowish solution was treated with Et_2O (100 mL) and kept at -32 °C for 5 h. The formed precipitate was filtered off, washed with Et_2O (50 ml) and dried under reduced pressure, yielding 19.0 g (88 %) of a white powder. **Mp** = 96 – 98 °C. **^{1}H NMR** (400 MHz, DMSO-*d*$_6$) δ = 11.66 (s, 1H), 9.37 – 8.69 (m, 2H), 2.73 – 2.53 (m, 3H), 2.45 – 2.28 (m, 2H), 2.10 – 1.94 (m, 2H), 1.15 – 0.99 (m, 3H) ppm. **^{13}C{^{1}H} NMR** (101 MHz, DMSO-*d*$_6$) δ = 168.0, 166.2, 31.6, 17.5, 13.6, 10.4 ppm. **IR (neat):** *v* = 3256 (m), 3179 (m), 3050 (m), 2972 (m), 2932 (m), 1625 (vs), 1567 (s), 1462 (m), 1438 (m), 1414 (s), 1366 (m), 1318 (m), 1223 (s), 1121 (m), 1074 (s), 989 (m), 963 (m), 728 (m), 702 (m), 662 (m), 598 (m), 573 (m), 525 (s), 471 (m), 428 (m) cm^{-1}. **HRMS** (ESI) *m/z*: [M]$^+$ Calcd for $C_6H_{14}N_3S_1$ 160.0903; Found 160.0894.

4.10) *S*-Methyl- cyclohexanone isothiosemicarbazonium iodide (3c)

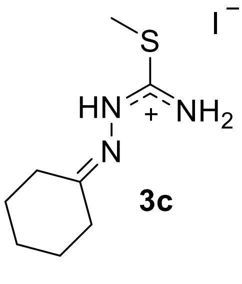

3c

Cyclohexanonethiosemicarbazone (75.0 mmol, 12.8 g) and iodomethane (78.8 mmol, 11.2 g, 1.05 eq.) were dispersed in a mixture of MeCN (35 mL) and MeOH (5 mL). The suspension was refluxed at 90 °C for 2 h and thereafter kept at -32 °C for 72 h. The yellowish solution was treated with Et_2O (150 mL), resulting in the separation of a yellow oil at the bottom. After keeping the mixture at -32 °C for 96 h, the oil had solidified and was filtered off, washed with Et_2O (100 ml) and dried under reduced pressure, yielding 15.8 g (67 %) of a white crystalline solid, suitable for further experiments. According to NMR, besides the *ring – chain* tautomerism[10] also *chair – boat* isomers of the cyclohexylidene moiety are

formed in solution. **Mp** = 109 – 111 °C. **^{1}H NMR** (400 MHz, acetonitrile-*d*$_3$) *δ* = 11.21 (s, 1H), 9.45 – 7.47 (m, 2H), 2.75 (s, 1.5H), 2.68 – 2.63 (m, 1H), 2.63 – 2.59 (m, 1H), 2.58 (s, 1.5H), 2.43 (t, *J*=6.3 Hz, 1H), 2.34 – 2.30 (m, 1H), 1.78 – 1.59 (m, 6H) ppm. **^{13}C{^{1}H} NMR** (101 MHz, acetonitrile-*d*$_3$) *δ* = 174.2, 172.0, 168.6, 167.6, 35.9, 35.6, 30.9, 30.7, 28.0, 27.1, 26.8, 25.7, 25.6, 15.5, 14.7 ppm. **IR (neat):** *v* = 3298 (m), 3251 (m), 3144 (m), 3095 (s), 2996 (w), 2944 (m), 2929 (m), 2855 (w), 1625 (vs), 1563 (s), 1498 (w), 1445 (s), 1423 (s), 1353 (m), 1318 (m), 1284 (m), 1243 (w), 1221 (w), 1109 (s), 1091 (s), 1020 (w), 994 (m), 961 (m), 845 (w), 730 (m), 701 (m), 668 (s), 604 (m), 578 (m), 530 (s), 477 (m), 445 (m) cm^{-1}. **HRMS** (ESI) *m/z*: [M]$^+$ Calcd for $C_8H_{16}N_3S_1$ 186.1059; Found 186.1049.

4.11) 5,5-Dimethyl-3-(methylthio)-1-(propan-2-ylidene)-4,5-dihydro-1*H*-1,2,4-triazol-1-ium iodide (4a)

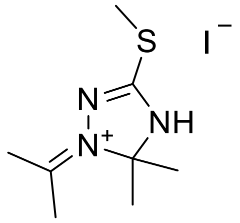

4a

In a 50 mL round bottom flask *S*-methyl- acetoneisothiosemicarbazonium iodide (**3a**, 1.36 g, 5.00 mmol) and activated molecular sieves (2.00 g; 3 Å) were suspended in acetone (20 mL) and 1.0 M pivalic acid – *N*,*N*-diisopropyl-*N*-ethylamine buffer solution (1.0 mL) was added. Subsequently, the vessel was sealed, and the mixture heated to 60 °C for 48 h without stirring. Over the course of the reaction the starting material dissolved gradually, and colorless product crystals formed on top of the molecular sieves. After cooling to room temperature, the solid matter was filtered off and washed with acetone (2 × 10 mL). After manual separation from the molecular sieves, the product was dried under reduced pressure, yielding 1.01 g (65 %) of slightly yellow crystals. **Mp** = 200 – 202 °C. **^{1}H NMR** (400 MHz, DMSO-*d*$_6$) δ = 9.92 (s, 1H), 2.60 (s, 3H), 2.57 (s, 3H), 2.43 (s, 3H), 1.85 (s, 6H) ppm. **^{13}C{^{1}H} NMR** (101 MHz, DMSO-*d*$_6$) δ = 167.4, 166.2, 88.7, 26.4 (2C), 25.2, 22.3, 13.1 ppm. **IR (neat)**: *v* = 3231 (w), 3143 (m), 2997 (w), 2910 (w), 1643 (m), 1500 (s), 1476 (vs), 1444 (vs), 1420 (vs), 1393 (s), 1377 (s), 1296 (m), 1259 (m), 1203 (s), 1165 (m), 1113 (m), 1064 (m), 1035 (m), 986 (m), 964 (m), 891 (m), 872 (m), 715 (w), 677 (w), 559 (m), 491 (m), 476 (m) cm^{-1}. **HRMS** (ESI) *m/z*: [M]$^+$ Calcd for $C_8H_{16}N_3S_1$ 186.1059; Found 186.1048.

4.12) 1-(Butan-2-ylidene)-5-ethyl-5-methyl-3-(methylthio)-4,5-dihydro-1*H*-1,2,4-triazol-1-ium iodide – mixture of *Z* – and *E* isomers (4b)

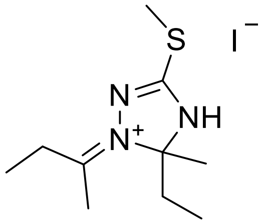

+

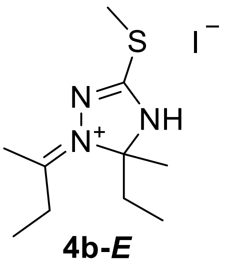

4b-*Z* **4b-*E***

In a 50 mL round bottom flask *S*-methyl- butanone isothiosemicarbazonium iodide (**3b**, 2.5 mmol, 0.72

g) and activated molecular sieves (1.0 g; 3 Å) were suspended in butanone (5 mL) and 1.0 M pivalic acid – *N*,*N*-diisopropyl-*N*-ethylamine buffer solution (0.5 mL) was added. Subsequently the reaction vessel was sealed and heated to 45 °C for 18.5 h without stirring. The resulting orange solution with crystalline sediment was treated with Et_2O (5 mL) and kept at -32 °C for 2 h. Thereafter the precipitate was filtered off, washed with Et_2O (10 mL) and manually separated from the molecular sieves, yielding 0.4 g of a yellowish crystalline powder as raw product. For further purification the solid was dissolved in MeOH (4 mL) in a small test tube and placed in a closed container filled with Et_2O (15 ml), which slowly diffused into the MeOH-phase. After keeping the vessel at 4 °C for 96 h slightly yellow crystals had formed, which were filtered off, washed with Et_2O (10 mL) and dried under reduced pressure, yielding 0.19 g (22 %) of a yellowish, crystalline solid. According to NMR, a mixture of the *Z* – and *E* isomer was isolated, with a proportion of approximately 80 % (*Z*) to 20 % (*E*), based on the integrals of the alkylidene methyl peaks in the ^{1}H-NMR spectrum at 2.57 ppm (*Z*) and 2.45 ppm (*E*). **Mp** = 192 – 194 °C (decomposition). **^{1}H NMR** (400 MHz, DMSO-d_6 – only the peaks of the *Z* – isomer are given) δ = 9.94 (s, 1H), 2.91 – 2.66 (m, 2H), 2.60 (s, 3H), 2.57 (s, 3H), 2.39 – 2.24 (m, 1H), 2.01 – 1.91 (m, 1H), 1.87 (s, 3H), 1.16 (t, *J*=7.5 Hz, 3H), 0.80 (t, *J*=7.3 Hz, 3H) ppm. **^{13}C{^{1}H} NMR** (101 MHz, DMSO-d_6 – only the peaks of the *Z* – isomer are given) δ = 171.3, 167.2, 92.3, 31.2, 30.9, 25.2, 19.6, 13.1, 8.9, 6.9 ppm. **IR (neat):** *v* = 3339 (w), 3116 (m), 2975 (m), 2936 (m), 2910 (m), 2728 (w), 1623 (w), 1505 (vs), 1481 (s), 1448 (vs), 1423 (vs), 1384 (s), 1367 (s), 1351 (m), 1289 (m), 1260 (m), 1192 (m), 1133 (m), 1103 (w), 1042 (m), 1022 (m), 994 (s), 966 (s), 938 (m), 862 (w), 751 (w), 598 (w), 554 (w), 507 (m), 487 (m) cm^{-1}. **HRMS** (ESI) *m/z*: [M]$^+$ Calcd for $C_{10}H_{20}N_3S_1$ 214.1372; Found 214.1359.

4.13) 1-Cyclohexylidene-3-(methylthio)-1,2,4-triazaspiro[4.5]dec-2-en-1-ium iodide (4c)

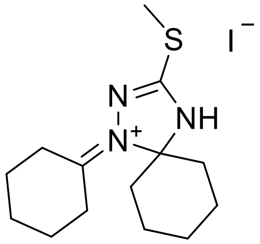

4c

In a 50 mL round bottom flask *S*-methyl- cyclohexanoneisothiosemicarbazonium iodide (**3c**, 5.0 mmol, 1.56 g) and activated molecular sieves (2.0 g; 3 Å) were suspended in cyclohexanone (10 mL) and 1.0 M pivalic acid – *N*,*N*-diisopropyl-*N*-ethylamine buffer solution (1.0 mL) was added. Subsequently the reaction vessel was sealed and heated to 60 °C for 64 h without stirring. The resulting orange solution with crystalline sediment was kept at -32 °C for 24 h. Thereafter the precipitate was filtered off, washed with cyclohexanone (20 mL) and Et_2O (20 mL). After manual separation from the molecular sieves, the product was dried under reduced pressure, yielding 0.52 g (26 %) of yellowish crystals. **Mp** = 177 – 179 °C (decomposition). **^{1}H NMR** (400 MHz, DMSO-d_6) *δ* = 10.23 (s, 1H), 3.03 (dt, *J*=9.7 Hz, 6.2 Hz, 4H), 2.56 (s, 3H), 2.28 (td, *J*=12.6 Hz, 4.4 Hz, 2H), 2.01 (d, *J*=12.7 Hz, 2H), 1.89 – 1.76 (m, 6H), 1.70 – 1.42 (m, 6H) ppm. **^{13}C{^{1}H} NMR** (101 MHz, DMSO-d_6) *δ* = 173.1, 166.3, 92.6, 34.7 (2C), 33.5, 31.4, 27.3, 26.2, 23.6, 22.8, 22.1 (2C), 13.1 ppm. **IR (neat):** *v* = 3046 (m), 2924 (m), 2896 (m), 2862 (m), 2694 (w), 1617 (w), 1502 (vs), 1476 (s), 1456 (s), 1314 (m), 1303 (m), 1240 (m), 1165 (w), 1142 (w), 1111 (w),

1067 (w), 1020 (m), 982 (s), 906 (m), 857 (w), 713 (w), 527 (m), 514 (m), 469 (w) cm^{-1}. **HRMS** (ESI) *m/z*: $[M]^+$ Calcd for $C_{14}H_{24}N_3S_1$ 266.1685; Found 266.1669.

4.14) (*Z*)-1-Benzylidene-5,5-dimethyl-3-(methylthio)-4,5-dihydro-1*H*-1,2,4-triazol-1-ium iodide (5a)

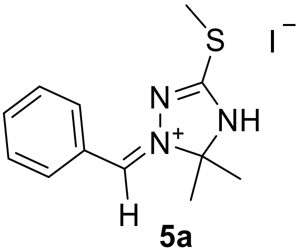

5a

S-Methyl- acetone isothiosemicarbazonium tetrafluoroborate (**1a**, 2.5 mmol, 0.58 g) and benzaldehyde (5.0 mmol, 0.53 g; 2 eq.) were dissolved in MeCN (2.5 mL) before activated molecular sieves (0.5 g; 3 Å) and 1.0 M pivalic acid – *N*,*N*-diisopropyl-*N*-ethylamine buffer solution (0.5 mL) were added. Subsequently the reaction vessel was sealed and heated to 50 °C for 5 h without stirring. The resulting orange solution was separated from the molecular sieves with a syringe and the residue was washed with MeCN (1.0 mL). Thereafter tetrabutylammonium iodide (2.4 mmol, 0.88 g, 0.96 eq) dissolved in MeCN (3.5 mL) was added – followed by Et_2O (10 mL), before the reaction vessel was kept at – 24 °C for 16 h. The resulting crystalline precipitate was filtered off, washed with cold MeCN (– 24 °C, 10 mL) and Et_2O (10 mL) and dried under reduced pressure, yielding 0.41 g (45 %) of yellow crystals. **Mp** = 179 – 181 °C (decomposition). **^{1}H NMR** (400 MHz, DMSO-d_6) δ = 10.93 (s, 1H), 8.80 (s, 1H), 8.48 – 8.42 (m, 2H), 7.80 – 7.67 (m, 3H), 2.75 (s, 3H), 1.89 (s, 6H) ppm. **^{13}C{^{1}H} NMR** (101 MHz, DMSO-d_6) δ = 170.7, 141.4, 134.7, 133.2 (2C), 129.4 (2C), 127.9, 91.0, 28.3 (2C), 13.6 ppm. **IR (neat):** v = 3012 (m), 1628 (w), 1592 (w), 1467 (vs), 1198 (s), 995 (s), 782 (s), 684 (s), 504 (m) cm^{-1}. **HRMS** (ESI) *m/z*: $[M]^+$ Calcd for $C_{12}H_{16}N_3S_1$ 234.1059; Found 234.1064.

4.15) (*Z*)-5,5-Dimethyl-1-(4-methylbenzylidene)-3-(methylthio)-4,5-dihydro-1*H*-1,2,4-triazol-1-ium iodide (5b)

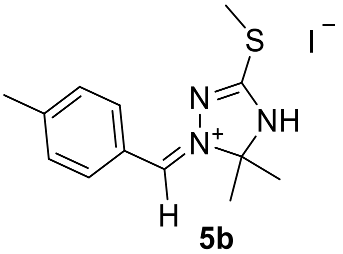

5b

S-Methyl- acetone isothiosemicarbazonium tetrafluoroborate (**1a**, 2.5 mmol, 0.58 g) and 4-methylbenzaldehyde (5.0 mmol, 0.60 g; 2 eq.) were dissolved in MeCN (2.5 mL) before activated molecular sieves (0.5 g; 3 Å) and 1.0 M pivalic acid – *N*,*N*-diisopropyl-*N*-ethylamine buffer solution (0.5 mL) were added. Subsequently the reaction vessel was sealed and heated to 50 °C for 5 h without stirring. The resulting orange solution was separated from the molecular sieves with a syringe and the residue was washed with MeCN (1.0 mL). Thereafter tetrabutylammonium iodide (2.4 mmol, 0.88 g, 0.96 eq) dissolved in MeCN (3.5 mL) was added – followed by Et_2O (10 mL), before the reaction vessel

was kept at – 24 °C for 16 h. The resulting crystalline precipitate was filtered off, washed with cold MeCN (– 24 °C, 10 mL) and Et_2O (10 mL) and dried under reduced pressure, yielding 0.51 g (54 %) of yellow crystals. **Mp** = 198 – 200 °C (decomposition). **^{1}H NMR** (400 MHz, DMSO-*d*$_6$) δ = 10.83 (s, 1H), 8.72 (s, 1H), 8.34 (d, *J* = 7.9 Hz, 2H), 7.52 (d, *J* = 8.0 Hz, 2H), 2.74 (s, 3H), 2.45 (s, 3H), 1.86 (s, 6H) ppm. **^{13}C{^{1}H} NMR** (101 MHz, DMSO-*d*$_6$) δ = 170.3, 146.0, 141.5, 133.3 (2C), 130.0 (2C), 125.4, 90.6, 28.3 (2C), 21.6, 13.5 ppm. **IR (neat):** v = 3012 (m), 1598 (m), 1440 (vs), 1379 (vs) 1185 (s), 1064 (s), 980 (s), 875 (s), 762 (s), 508 (vs) cm^{-1}. **HRMS** (ESI) *m/z*: [M]$^+$ Calcd for $C_{13}H_{18}N_3S_1$ 248.1216; Found 248.1227.

4.16) (*Z*)-1-(4-Chlorobenzylidene)-5,5-dimethyl-3-(methylthio)-4,5-dihydro-1*H*-1,2,4-triazol-1-ium iodide (5c)

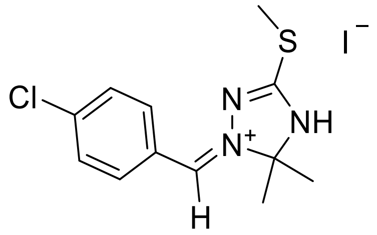

5c

S-Methyl- acetone isothiosemicarbazonium tetrafluoroborate (**1a**, 2.5 mmol, 0.58 g) and 4-chlorobenzaldehyde (5.0 mmol, 0.70 g; 2 eq.) were dissolved in MeCN (2.5 mL) before activated molecular sieves (0.5 g; 3 Å) and 1.0 M pivalic acid – *N*,*N*-diisopropyl-*N*-ethylamine buffer solution (0.5 mL) were added. Subsequently the reaction vessel was sealed and heated to 50 °C for 5 h without stirring. The resulting orange solution was separated from the molecular sieves with a syringe and the residue was washed with MeCN (1.0 mL). Thereafter tetrabutylammonium iodide (2.4 mmol, 0.88 g, 0.96 eq) dissolved in MeCN (3.5 mL) was added – followed by Et_2O (10 mL), before the reaction vessel was kept at – 24 °C for 16 h. The resulting crystalline precipitate was filtered off, washed with cold MeCN (– 24 °C, 10 mL) and Et_2O (10 mL) and dried under reduced pressure, yielding 0.43 g (44 %) of an orange crystalline solid. **Mp** = 182 – 184 °C (decomposition). **^{1}H NMR** (400 MHz, DMSO-*d*$_6$) δ = 11.01 (s, 1H), 8.77 (s, 1H), 8.44 (d, *J* = 7.1 Hz, 2H), 7.79 (d, *J* = 8.7 Hz, 2H), 2.74 (s, 3H), 1.88 (s, 6H) ppm. **^{13}C{^{1}H} NMR** (101 MHz, DMSO-*d*$_6$) δ = 170.9, 140.0, 139.3, 134.7 (2C), 129.6 (2C), 126.8, 91.3, 28.3 (2C), 13.6 ppm. **IR (neat):** v = 2982 (m), 2884 (m), 1580 (m), 1483 (m), 1372 (s), 1093 (m), 869 (s), 812 (s), 506 (vs) cm^{-1}. **HRMS** (ESI) *m/z*: [M]$^+$ Calcd for $C_{12}H_{15}N_3S_1Cl_1$ 268.0670; Found 268.0677.

4.17) (*Z*)-5,5-Dimethyl-3-(methylthio)-1-(4-nitrobenzylidene)-4,5-dihydro-1*H*-1,2,4-triazol-1-ium iodide (5d)

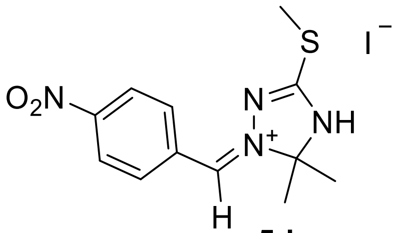

5d

S-Methyl- acetone isothiosemicarbazonium tetrafluoroborate (**1a**, 2.5 mmol, 0.58 g) and 4-nitrobenzaldehyde (5.0 mmol, 0.76 g; 2 eq.) were dissolved in MeCN (2.5 mL) before activated molecular sieves (0.5 g; 3 Å) and 1.0 M pivalic acid – *N*,*N*-diisopropyl-*N*-ethylamine buffer solution (0.5 mL) were added. Subsequently the reaction vessel was sealed and heated to 50 °C for 5 h without stirring. The resulting dark orange solution was separated from the molecular sieves with a syringe and the residue was washed with MeCN (1.0 mL). Thereafter tetrabutylammonium iodide (2.4 mmol, 0.88 g, 0.96 eq) dissolved in MeCN (3.5 mL) was added – followed by Et_2O (10 mL), before the reaction vessel was kept at – 24 °C for 16 h. The resulting crystalline precipitate was filtered off, washed with cold MeCN (– 24 °C, 10 mL) and Et_2O (10 mL) and dried under reduced pressure, yielding 0.48 g (47 %) of a dark orange crystalline solid. **Mp** = 181 – 183 °C (decomposition). **^{1}H NMR** (400 MHz, DMSO-d_6) δ = 11.27 (s, 1H), 8.90 (s, 1H), 8.65 (d, J = 9.0 Hz, 2H), 8.48 (d, J = 9.0 Hz, 2H), 2.77 (s, 3H), 1.91 (s, 6H) ppm. **^{13}C{^{1}H} NMR** (101 MHz, DMSO-d_6) δ = 171.8, 149.3, 138.1, 133.9 (2C), 133.3, 124.1 (2C), 92.6, 28.3 (2C), 13.7 ppm. **IR (neat):** v = 3033 (m), 2945 (m), 1595 (s), 1517 (s), 1442 (vs), 1301 (s), 990 (s), 868 (s), 745 (s), 682 (s), 498 (m) cm^{-1}. **HRMS** (ESI) *m/z*: $[M]^+$ Calcd for $C_{12}H_{15}N_4S_1O_2$ 279.0910; Found 279.0911.

4.18) (*Z*)-1-(4-Methoxybenzylidene)-5,5-dimethyl-3-(methylthio)-4,5-dihydro-1*H*-1,2,4-triazol-1-ium iodide (5e)

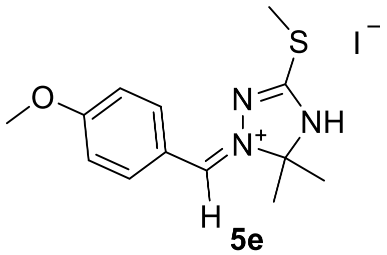

5e

S-Methyl- acetone isothiosemicarbazonium tetrafluoroborate (**1a**, 2.5 mmol, 0.58 g) and 4-methoxybenzaldehyde (5.0 mmol, 0.68 g; 2 eq.) were dissolved in MeCN (2.5 mL) before activated molecular sieves (0.5 g; 3 Å) and 1.0 M pivalic acid – *N*,*N*-diisopropyl-*N*-ethylamine buffer solution (0.5 mL) were added. Subsequently the reaction vessel was sealed and heated to 50 °C for 5 h without stirring. The resulting orange solution was separated from the molecular sieves with a syringe and the residue was washed with MeCN (1.0 mL). Thereafter tetrabutylammonium iodide (2.4 mmol, 0.88 g, 0.96 eq) dissolved in MeCN (3.5 mL) was added – followed by Et_2O (10 mL), before the reaction vessel was kept at – 24 °C for 16 h. The resulting crystalline precipitate was filtered off, washed with cold MeCN (– 24 °C, 10 mL) and Et_2O (10 mL) and dried under reduced pressure, yielding 0.49 g (50 %) of a yellow crystalline solid. **Mp** = 185 – 186 °C (decomposition). **^{1}H NMR** (400 MHz, DMSO-d_6) δ = 10.66 (s, 1H), 8.68 (s, 1H), 8.44 (d, J = 9.1 Hz, 2H), 7.26 (d, J = 9.1 Hz, 2H), 3.92 (s, 3H), 2.73 (s, 3H), 1.85 (s, 6H) ppm. **^{13}C{^{1}H} NMR** (101 MHz, DMSO-d_6) δ = 169.5, 164.4, 141.1, 136.0 (2C), 120.6, 115.1 (2C), 89.8, 56.0, 28.4 (2C), 13.5 ppm. **IR (neat):** v = 3090 (m), 2940 (m), 1592 (s), 1450 (vs), 1260 (s), 1173 (s), 1016 (s), 836 (vs), 575 (m), 478 (m) cm^{-1}. **HRMS** (ESI) *m/z*: $[M]^+$ Calcd for $C_{13}H_{18}N_3S_1O_1$ 264.1165; Found 264.1170.

4.19) (*Z*)-1-Benzylidene-3-(ethylthio)-5,5-dimethyl-4,5-dihydro-1*H*-1,2,4-triazol-1-ium iodide (6a)

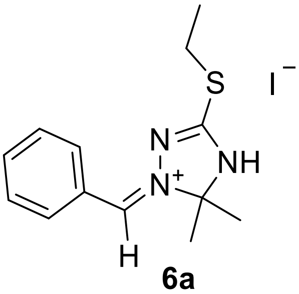

6a

S-Ethyl- acetone isothiosemicarbazonium tetrafluoroborate (**1d**, 2.5 mmol, 0.62 g) and benzaldehyde (5.0 mmol, 0.53 g; 2 eq.) were dissolved in MeCN (2.5 mL) before activated molecular sieves (0.5 g; 3 Å) and 1.0 M pivalic acid – *N*,*N*-diisopropyl-*N*-ethylamine buffer solution (0.5 mL) were added. Subsequently the reaction vessel was sealed and heated to 50 °C for 5 h without stirring. The resulting orange solution was separated from the molecular sieves with a syringe and the residue was washed with MeCN (1.0 mL). Thereafter tetrabutylammonium iodide (2.4 mmol, 0.88 g, 0.96 eq) dissolved in MeCN (3.5 mL) was added – followed by Et_2O (15 mL), before the reaction vessel was kept at – 24 °C for 96 h. The resulting precipitate was filtered off, washed with cold MeCN (– 24 °C, 10 mL) and Et_2O (10 mL) and dried under reduced pressure, yielding 0.53 g (57 %) of a yellow solid. **Mp** = 167 – 169 °C (decomposition). **^{1}H NMR** (400 MHz, DMSO-d_6) δ = 10.94 (s, 1H), 8.82 (s, 1H), 8.50 – 8.41 (m, 2H), 7.80 – 7.66 (m, 3H), 3.33 (q, J = 7.3 Hz, 2H), 1.89 (s, 6H), 1.45 (t, J = 7.3 Hz, 3H) ppm. **^{13}C{^{1}H} NMR** (101 MHz, DMSO-d_6) δ = 169.7, 141.5, 134.7, 133.1 (2C), 129.4 (2C), 128.0, 90.6, 28.3 (2C), 25.8, 14.7 ppm. **IR (neat):** v = 3114 (m), 2957 (m), 1592 (s), 1442 (vs), 1200 (s), 988 (s), 756 (s), 688 (s), 490 (m) cm^{-1}. **HRMS** (ESI) *m/z*: $[M]^+$ Calcd for $C_{13}H_{18}N_3S_1$ 248.1216; Found 248.1219.

4.20) (*Z*)-3-(Ethylthio)-5,5-dimethyl-1-(4-methylbenzylidene)-4,5-dihydro-1*H*-1,2,4-triazol-1-ium iodide (6b)

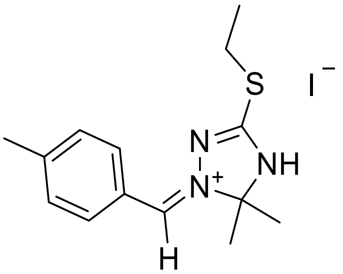

6b

S-Ethyl- acetone isothiosemicarbazonium tetrafluoroborate (**1d**, 2.5 mmol, 0.62 g) and 4-methylbenzaldehyde (5.0 mmol, 0.60 g; 2 eq.) were dissolved in MeCN (2.5 mL) before activated molecular sieves (0.5 g; 3 Å) and 1.0 M pivalic acid – *N*,*N*-diisopropyl-*N*-ethylamine buffer solution (0.5 mL) were added. Subsequently the reaction vessel was sealed and heated to 50 °C for 5 h without stirring. The resulting orange solution was separated from the molecular sieves with a syringe and the residue was washed with MeCN (1.0 mL). Thereafter tetrabutylammonium iodide (2.4 mmol, 0.88 g, 0.96 eq) dissolved in MeCN (3.5 mL) was added – followed by Et_2O (15 mL), before the reaction vessel

was kept at – 24 °C for 96 h. The resulting precipitate was filtered off, washed with cold MeCN (– 24 °C, 10 mL) and Et_2O (10 mL) and dried under reduced pressure, yielding 0.57 g (59 %) of a yellow solid. **Mp** = 192 – 195 °C (decomposition). **^{1}H NMR** (400 MHz, DMSO-*d*$_6$) δ = 10.84 (s, 1H), 8.76 (s, 1H), 8.35 (d, *J* = 8.4 Hz, 2H), 7.52 (d, *J* = 8.2 Hz, 2H), 3.32 (q, *J* = 7.3 Hz, 2H), 2.44 (s, 3H), 1.87 (s, 6H), 1.44 (t, *J* = 7.3 Hz, 3H) ppm. **^{13}C{^{1}H} NMR** (101 MHz, DMSO-*d*$_6$) δ = 169.3, 146.0, 141.5, 133.2 (2C), 130.0 (2C), 125.4, 90.1, 28.3 (2C), 25.7, 21.6, 14.7 ppm. **IR (neat):** ν = 3051 (w), 2942 (w), 1597 (m), 1485 (s), 1450 (s), 1185 (m), 979 (m), 812 (s), 506 (vs) cm^{-1}. **HRMS** (ESI) *m/z*: [M]$^+$ Calcd for $C_{14}H_{20}N_3S_1$ 262.1372; Found 262.1376.

4.21) (*Z*)-1-(4-Chlorobenzylidene)-3-(ethylthio)-5,5-dimethyl-4,5-dihydro-1*H*-1,2,4-triazol-1-ium iodide (6c)

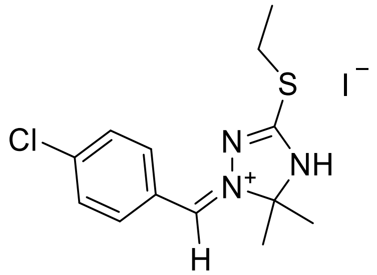

6c

S-Ethyl- acetone isothiosemicarbazonium tetrafluoroborate (**1d**, 2.5 mmol, 0.62 g) and 4-chlorobenzaldehyde (5.0 mmol, 0.70 g; 2 eq.) were dissolved in MeCN (2.5 mL) before activated molecular sieves (0.5 g; 3 Å) and 1.0 M pivalic acid – *N*,*N*-diisopropyl-*N*-ethylamine buffer solution (0.5 mL) were added. Subsequently the reaction vessel was sealed and heated to 50 °C for 5 h without stirring. The resulting orange solution was separated from the molecular sieves with a syringe and the residue was washed with MeCN (1.0 mL). Thereafter tetrabutylammonium iodide (2.4 mmol, 0.88 g, 0.96 eq) dissolved in MeCN (3.5 mL) was added – followed by Et_2O (25 mL), before the reaction vessel was kept at – 24 °C for 48 h. The resulting crystalline precipitate was filtered off, washed with cold MeCN (– 24 °C, 10 mL), yielding 0.98 g of an orange crystalline solid as a raw product impurified with buffer substances. For further purification the solid was dissolved in MeOH (2 mL) in a small test tube and placed in a closed container filled with Et_2O (15 ml), which slowly diffused into the MeOH-phase. After keeping the vessel at 4 °C for 96 h crystals had formed, which were filtered off, washed with Et_2O (10 mL) and dried under reduced pressure, yielding 0.52 g (51 %) of orange crystals. **Mp** = 174 – 175 °C (decomposition). **^{1}H NMR** (400 MHz, DMSO-*d*$_6$) δ = 11.02 (s, 1H), 8.83 (s, 1H), 8.45 (d, *J* = 8.8 Hz, 2H), 7.84 – 7.75 (m, 2H), 3.33 (q, *J* = 7.3 Hz, 2H), 1.89 (s, 6H), 1.44 (t, *J* = 7.3 Hz, 3H) ppm. **^{13}C{^{1}H} NMR** (101 MHz, DMSO-*d*$_6$) δ = 169.9, 140.1, 139.3, 134.6 (2C), 129.6 (2C), 126.8, 90.8, 28.3 (2C), 25.8, 14.7 ppm. **IR (neat)**: ν = 3040 (m), 2951 (m), 1586 (m), 1474 (vs), 1380 (s), 1198 (m), 1061 (m), 825 (vs), 509 (s) cm^{-1}. **HRMS** (ESI) *m/z*: [M]$^+$ Calcd for $C_{13}H_{17}N_3S_1Cl_1$ 282.0826; Found 282.0829.

4.22) (*Z*)-3-(Ethylthio)-5,5-dimethyl-1-(4-nitrobenzylidene)-4,5-dihydro-1*H*-1,2,4-triazol-1-ium iodide (6d)

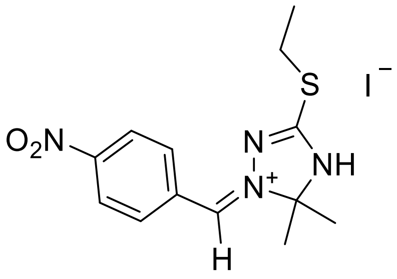

6d

S-Ethyl- acetone isothiosemicarbazonium tetrafluoroborate (**1d**, 2.5 mmol, 0.62 g) and 4-nitrobenzaldehyde (5.0 mmol, 0.76 g; 2 eq.) were dissolved in MeCN (2.5 mL) before activated molecular sieves (0.5 g; 3 Å) and 1.0 M pivalic acid – *N*,*N*-diisopropyl-*N*-ethylamine buffer solution (0.5 mL) were added. Subsequently the reaction vessel was sealed and heated to 50 °C for 5 h without stirring. The resulting dark orange solution was separated from the molecular sieves with a syringe and the residue was washed with MeCN (1.0 mL). Thereafter tetrabutylammonium iodide (2.4 mmol, 0.88 g, 0.96 eq) dissolved in MeCN (3.5 mL) was added – followed by Et_2O (15 mL), before the reaction vessel was kept at – 24 °C for 96 h. The resulting precipitate was filtered off, washed with cold MeCN (– 24 °C, 10 mL) and Et_2O (10 mL) and dried under reduced pressure, yielding 0.46 g (44 %) of a dark orange solid. **Mp** = 178 – 180 °C (decomposition). **^{1}H NMR** (400 MHz, DMSO-*d*$_6$) δ = 11.32 (s, 1H), 8.86 (s, 1H), 8.63 (d, *J* = 9.0 Hz, 2H), 8.50 (d, *J* = 9.0 Hz, 2H), 3.36 (q, *J* = 7.3 Hz, 2H), 1.90 (s, 6H), 1.46 (t, *J* = 7.3 Hz, 3H) ppm. **^{13}C{^{1}H} NMR** (101 MHz, DMSO-*d*$_6$) δ = 170.9, 149.3, 138.1, 133.7 (2C), 133.4, 124.2 (2C), 92.3, 28.3 (2C), 25.9, 14.7 ppm. **IR (neat):** v = 3027 (m), 2940 (m), 1598 (s), 1518 (s), 1375 (s), 1304 (s), 1198 (s), 1061 (s), 985 (s), 841 (s), 746 (vs), 682 (s), 502 (s) cm^{-1}. **HRMS** (ESI) *m/z*: $[M]^+$ Calcd for $C_{13}H_{17}N_4S_1O_2$ 293.1067; Found 293.1068.

4.23) (*Z*)-3-(ethylthio)-1-(4-methoxybenzylidene)-5,5-dimethyl-4,5-dihydro-1*H*-1,2,4-triazol-1-ium iodide (6e)

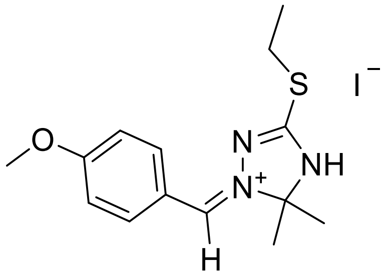

6e

S-Ethyl- acetone isothiosemicarbazonium tetrafluoroborate (**1d**, 2.5 mmol, 0.62 g) and 4-methoxybenzaldehyde (5.0 mmol, 0.68 g; 2 eq.) were dissolved in MeCN (2.5 mL) before activated molecular sieves (0.5 g; 3 Å) and 1.0 M pivalic acid – *N*,*N*-diisopropyl-*N*-ethylamine buffer solution (0.5 mL) were added. Subsequently the reaction vessel was sealed and heated to 50 °C for 5 h without stirring. The resulting orange solution was separated from the molecular sieves with a syringe and the residue was washed with MeCN (1.0 mL). Thereafter tetrabutylammonium iodide (2.4 mmol, 0.88 g,

0.96 eq) dissolved in MeCN (3.5 mL) was added – followed by Et_2O (25 mL), before the reaction vessel was kept at – 24 °C for 16 h. The resulting crystalline precipitate was filtered off, washed with cold MeCN (– 24 °C, 10 mL) and Et_2O (10 mL), yielding 0.70 g of a yellow crystalline solid as a raw product impurified with buffer substances. For further purification the solid was dissolved in MeOH (2 mL) in a small test tube and placed in a closed container filled with Et_2O (15 ml), which slowly diffused into the MeOH-phase. After keeping the vessel at 4 °C for 96 h crystals had formed, which were filtered off, washed with Et_2O (10 mL) and dried under reduced pressure, yielding 0.52 g (49 %) of yellow crystals. According to NMR the MeOH hemisolvate **6e · 0.5 MeOH** was formed. **Mp** = 169 – 171 °C (decomposition). **^{1}H NMR** (400 MHz, DMSO-d_6) δ = 10.67 (s, 1H), 8.67 (s, 1H), 8.43 (d, J = 9.0 Hz, 2H), 7.27 (d, J = 9.1 Hz, 2H), 3.92 (s, 3H), 3.31 (q, J = 7.3 Hz, 2H), 1.84 (s, 6H), 1.45 (t, J = 7.3 Hz, 3H) ppm. **^{13}C{^{1}H} NMR** (101 MHz, DMSO-d_6) δ = 168.6, 164.4, 141.3, 135.9 (2C), 120.6, 115.2 (2C), 89.4, 56.1, 28.3 (2C), 25.7, 14.7 ppm. **IR (neat):** v = 3044 (w), 2941(w), 1595 (s),1446 (s),1261 (s),1177 (s), 1014 (m), 865 (vs), 527 (m) cm^{-1}. **HRMS** (ESI) *m/z*: $[M]^+$ Calcd for $C_{14}H_{20}N_3S_1O_1$ 278.1322; Found 278.1328.

5) Spectral characterization

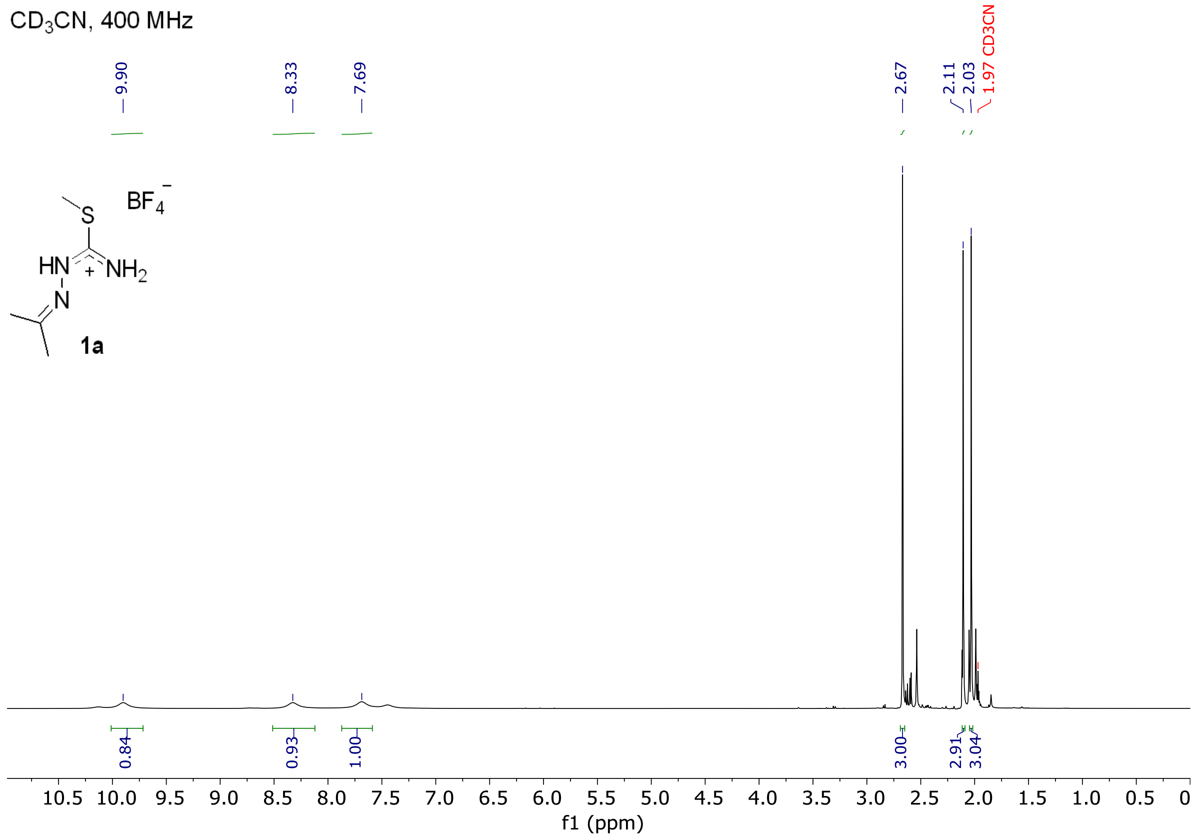

Figure S16. ^{1}H NMR spectrum (acetonitrile-d_3, 400 MHz) of **1a**

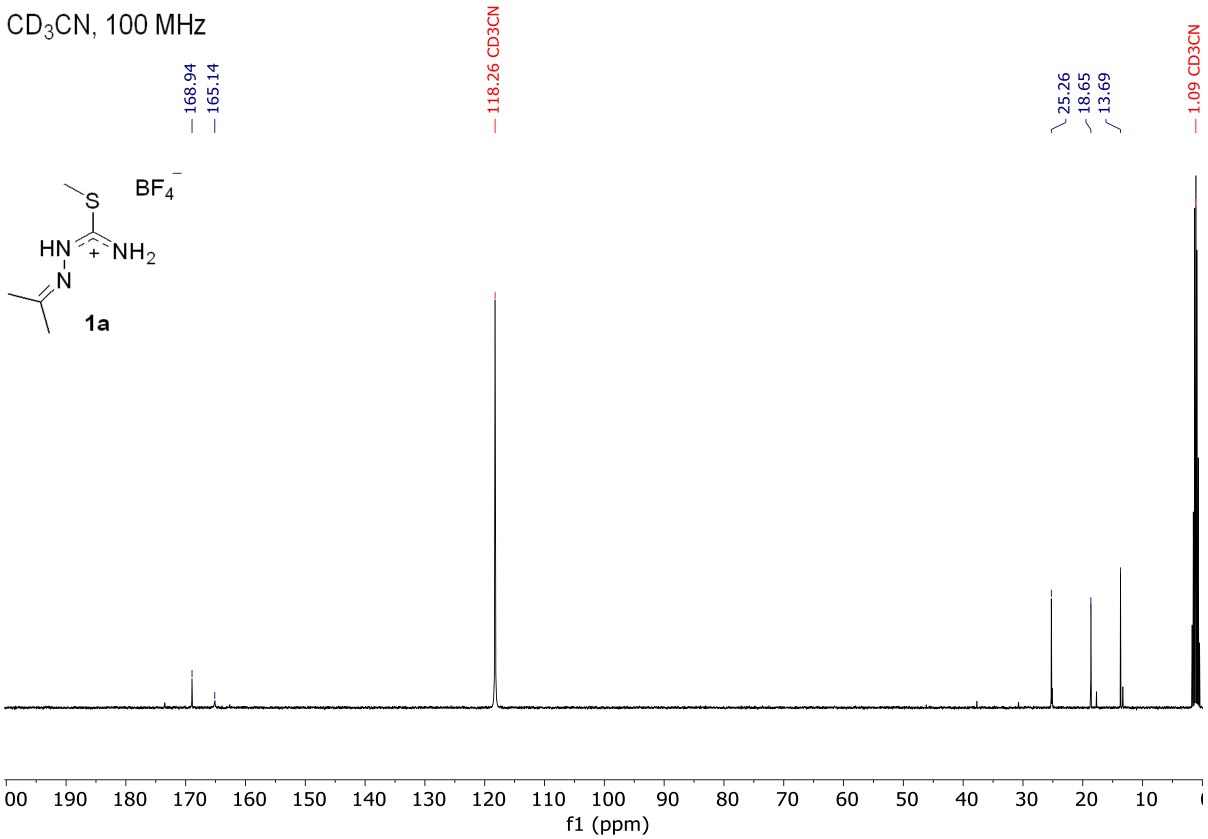

Figure S17. $^{13}C\{^{1}H\}$ NMR spectrum (acetonitrile-d_3, 100 MHz) of **1**

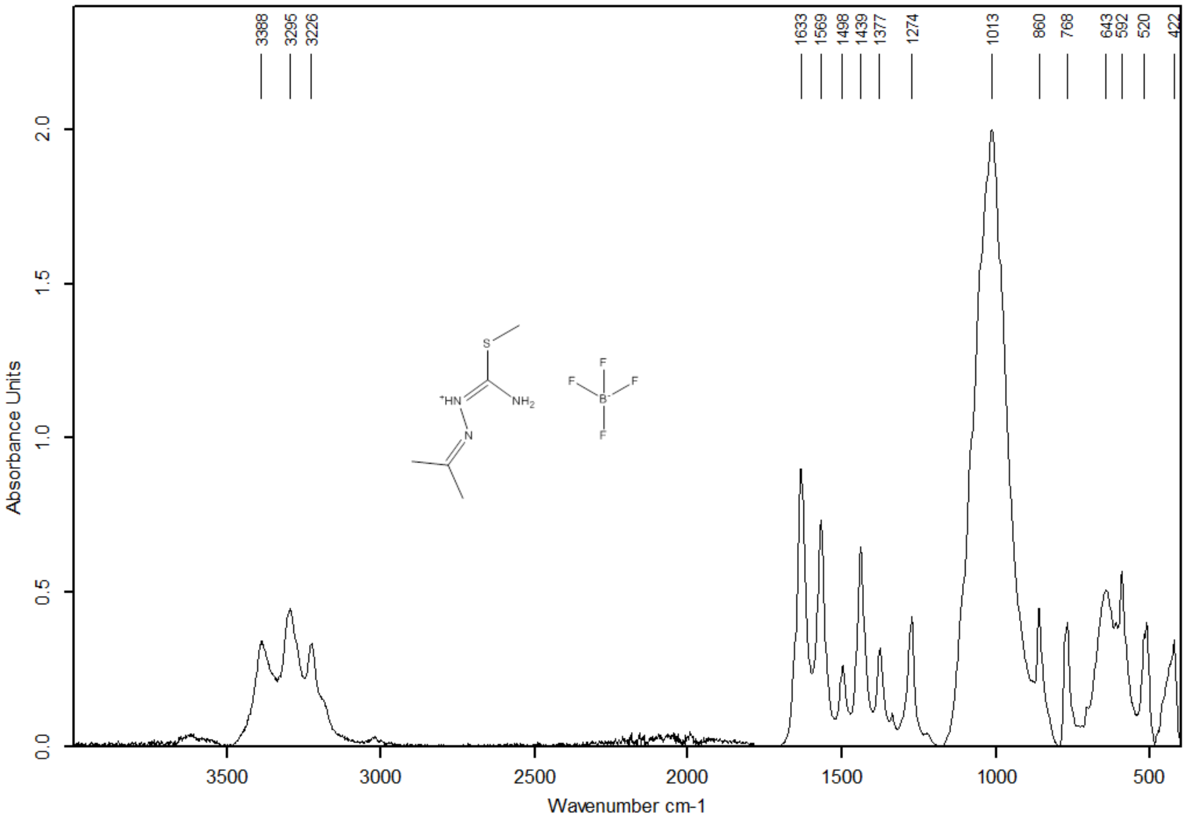

Figure S18. IR- absorption spectrum (neat) **1a**

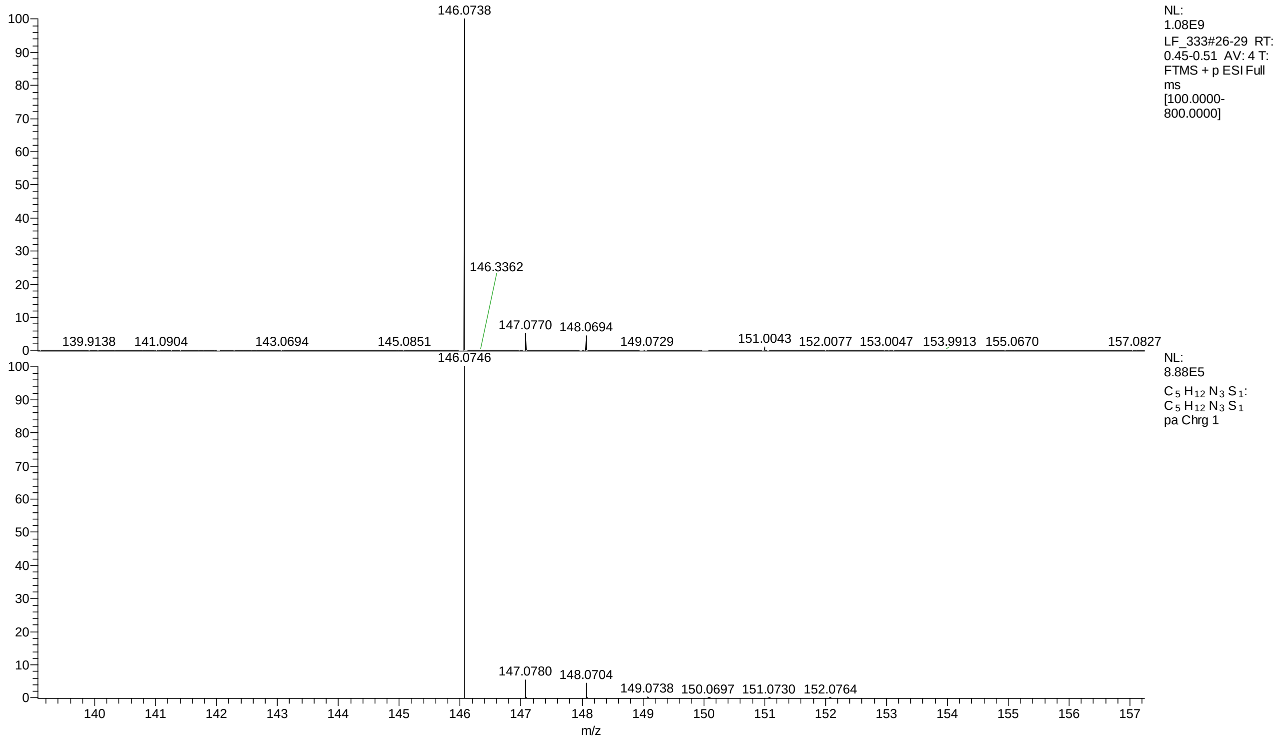

Figure S19. HR-ESI-MS (*m/z*) of **1a**. Top: measured peaks for $[C_5H_{12}N_3S_1]^+$; Bottom: calculated peaks for $[C_5H_{12}N_3S_1]^+$

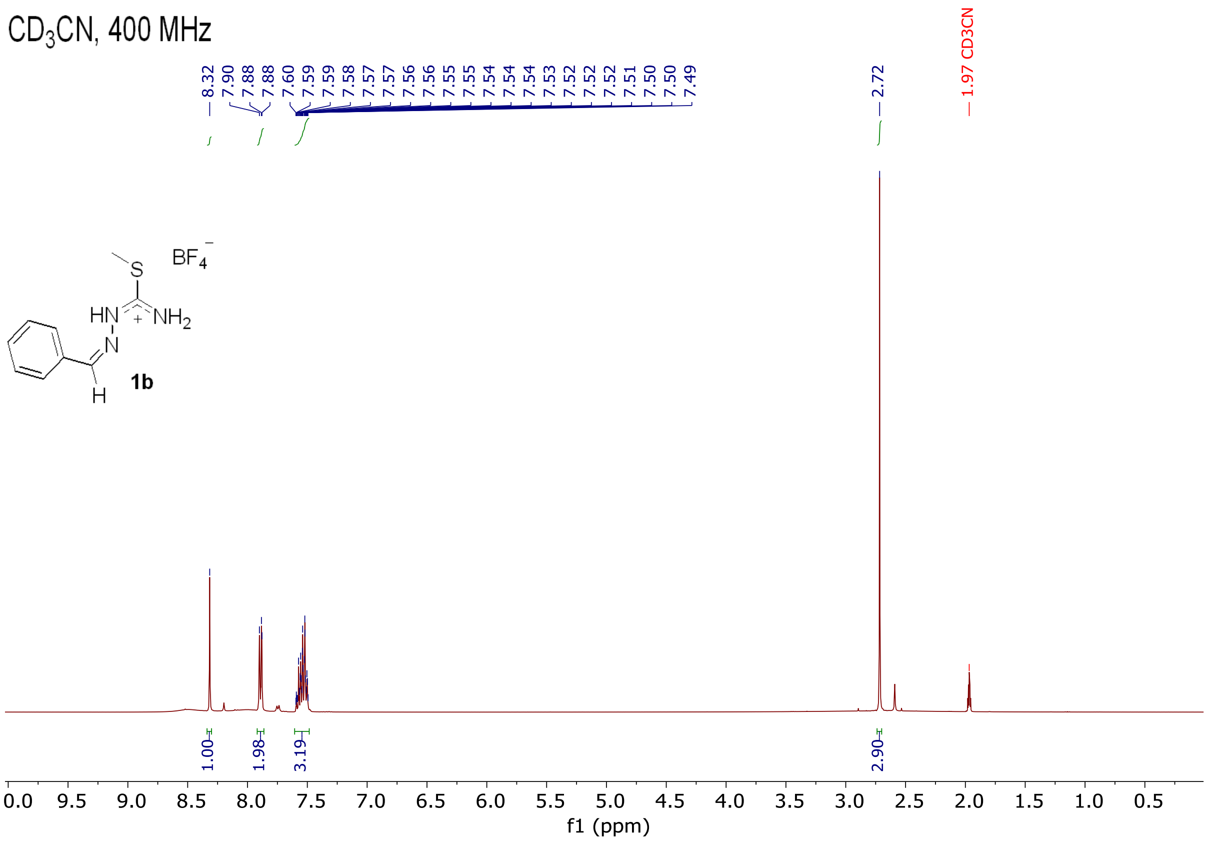

Figure S20. ^{1}H NMR spectrum (acetonitrile-d_3, 400 MHz) of **1b**

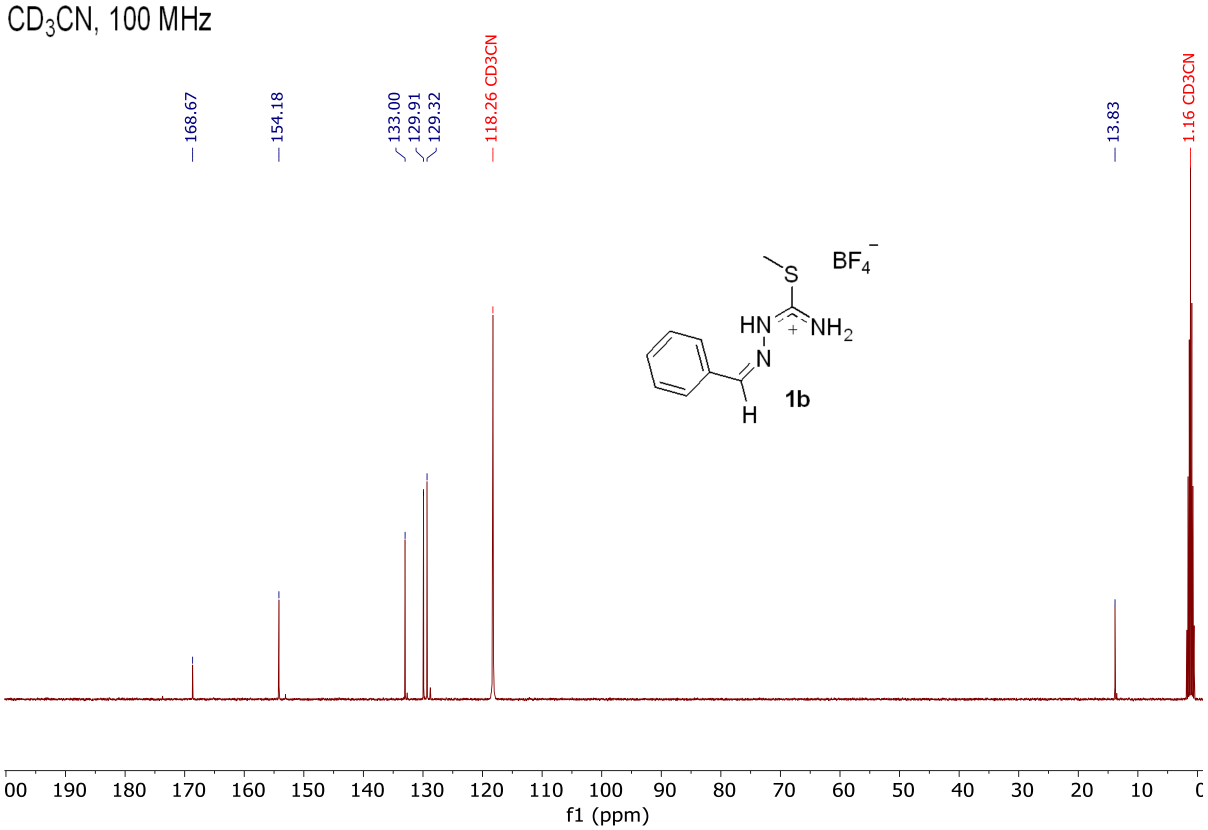

Figure S21. ^{13}C{^{1}H} NMR spectrum (acetonitrile-d_3, 100 MHz) of **1b**

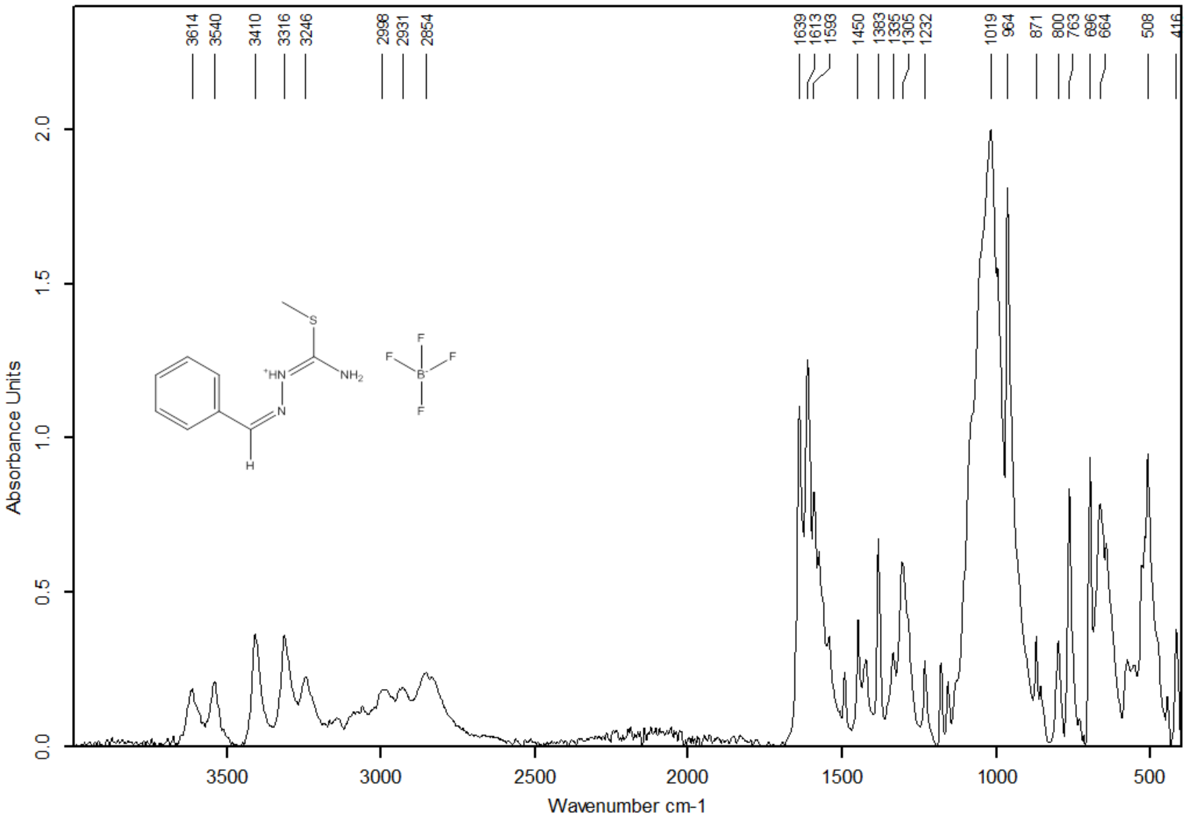

Figure S22. IR- absorption spectrum (neat) of **1b**

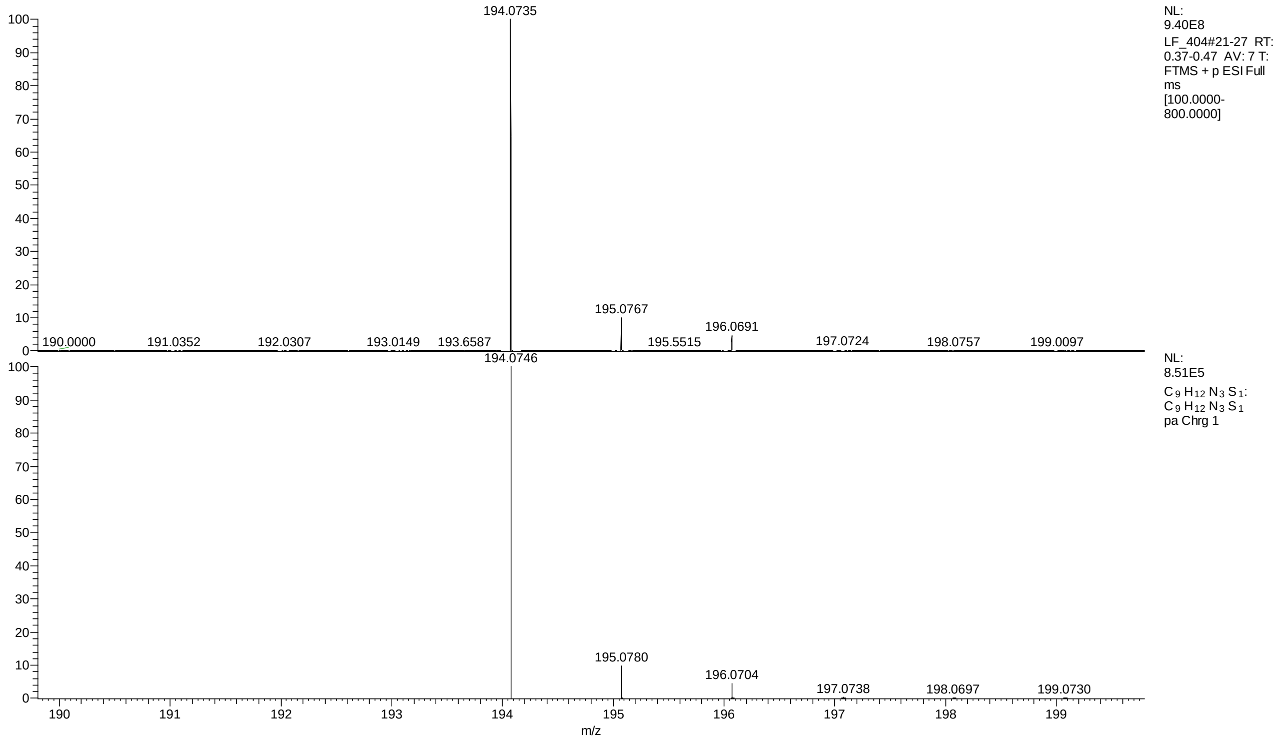

Figure S23. HR-ESI-MS (*m/z*) of **1b**. Top: measured peaks for $[C_9H_{12}N_3S_1]^+$; Bottom: calculated peaks for $[C_9H_{12}N_3S_1]^+$

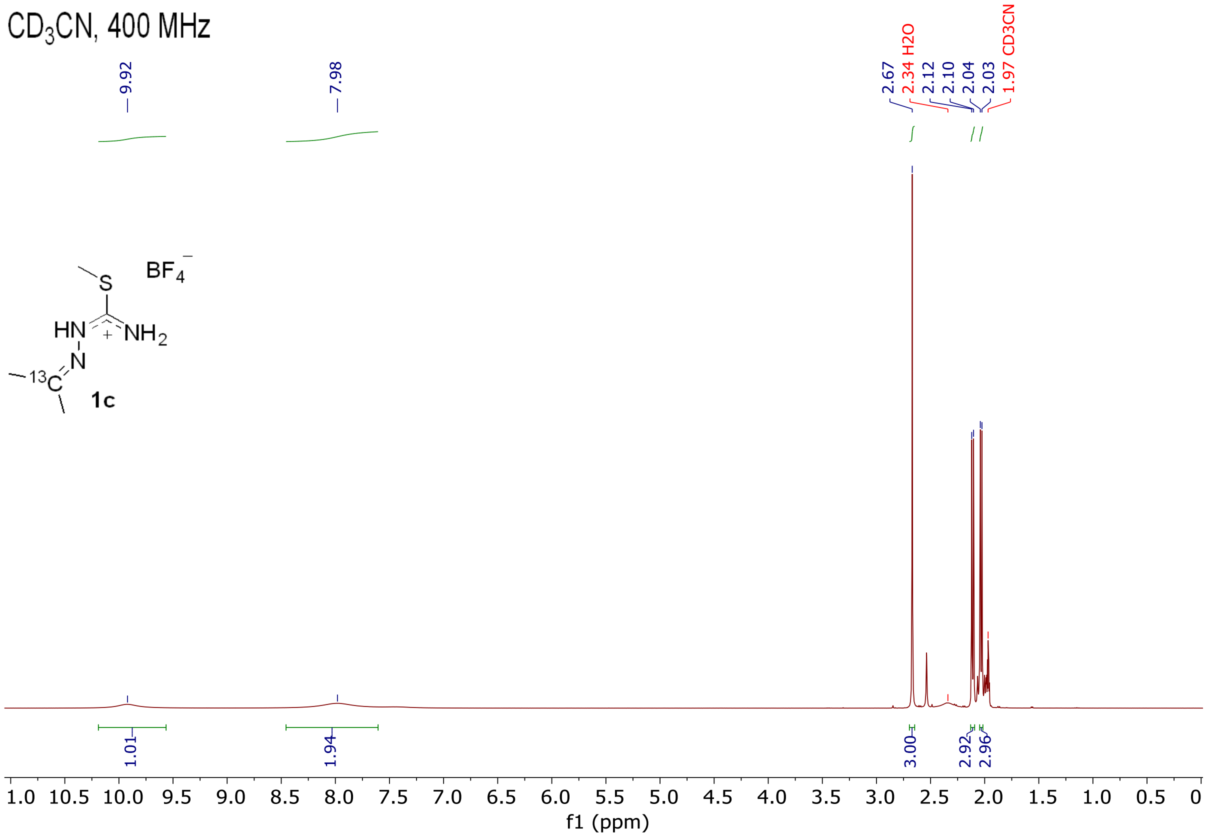

Figure S24. ^{1}H NMR spectrum (acetonitrile-d_3, 400 MHz) of **1c**

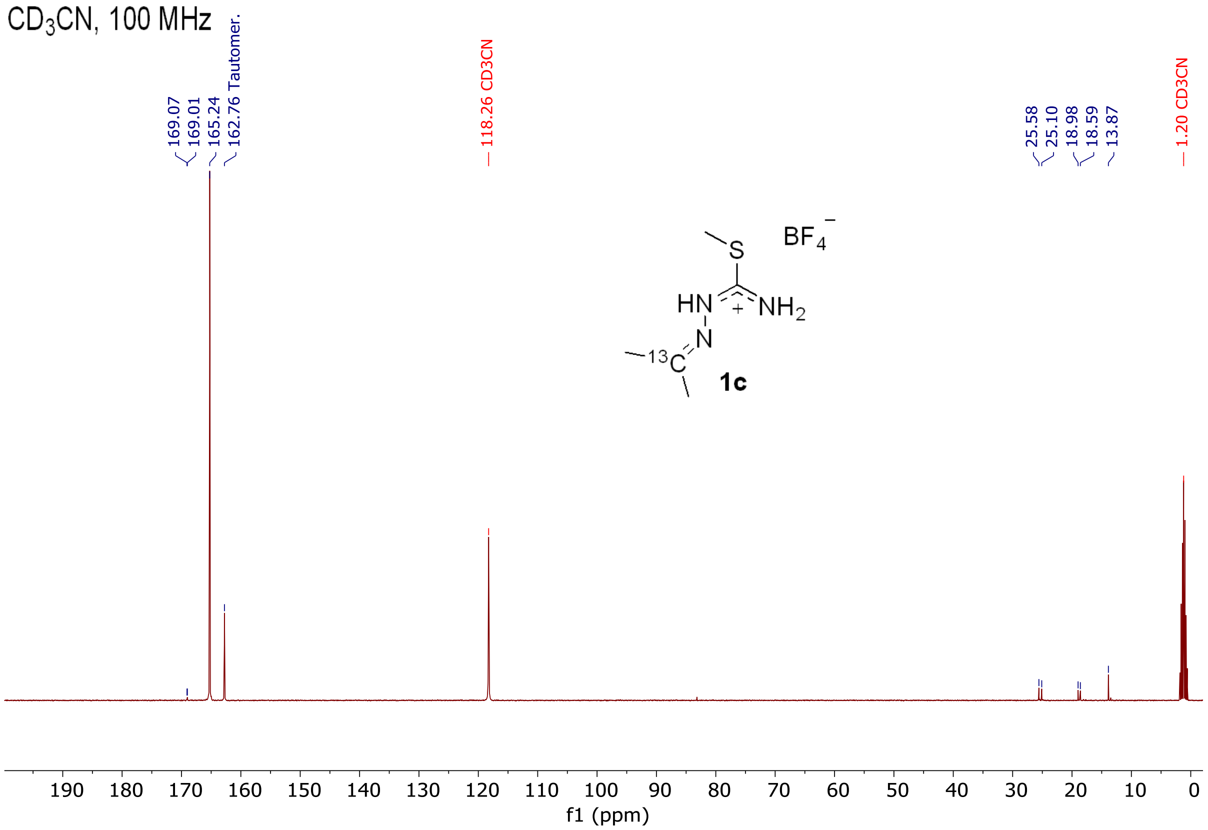

Figure S25. $^{13}C\{^{1}H\}$ NMR spectrum (acetonitrile-d_3, 100 MHz) of **1c**

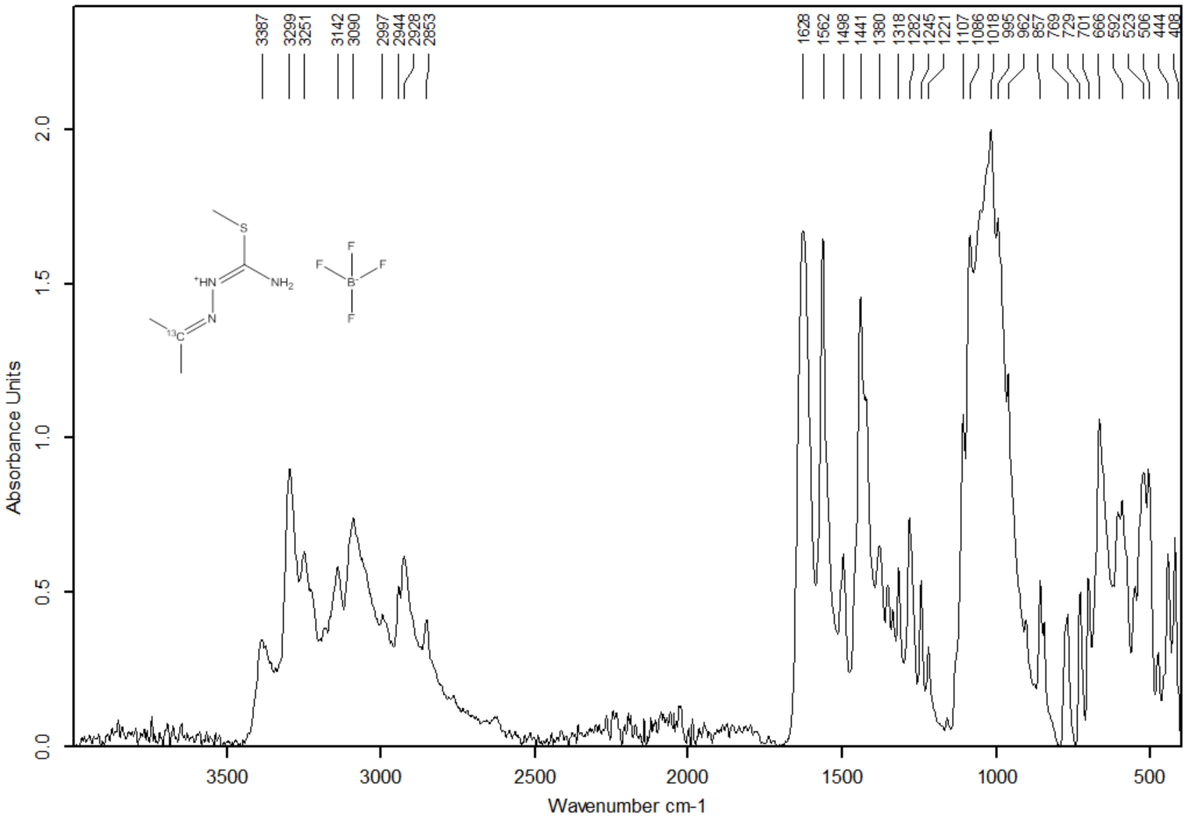

Figure S26. IR- absorption spectrum (neat) of **1c**

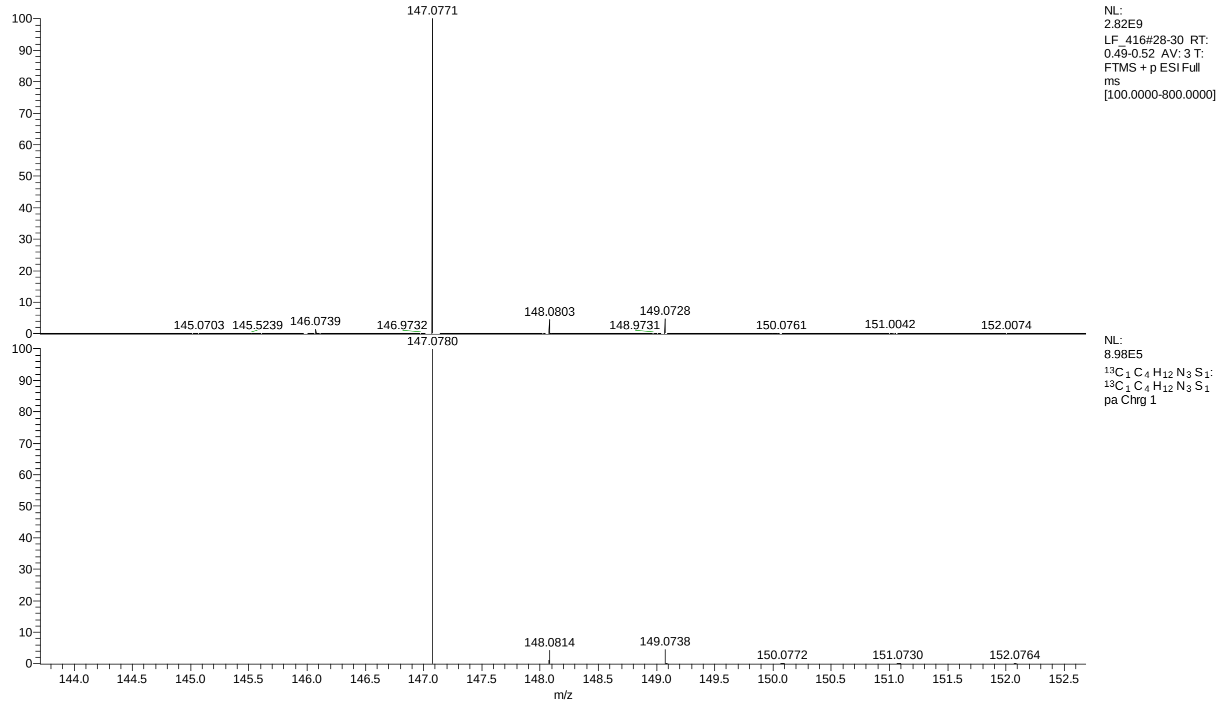

Figure S27. HR-ESI-MS (*m/z*) of **1c**. Top: measured peaks for $[^{13}C_1C_4H_{12}N_3S_1]^+$; Bottom: calculated peaks for $[^{13}C_1C_4H_{12}N_3S_1]^+$

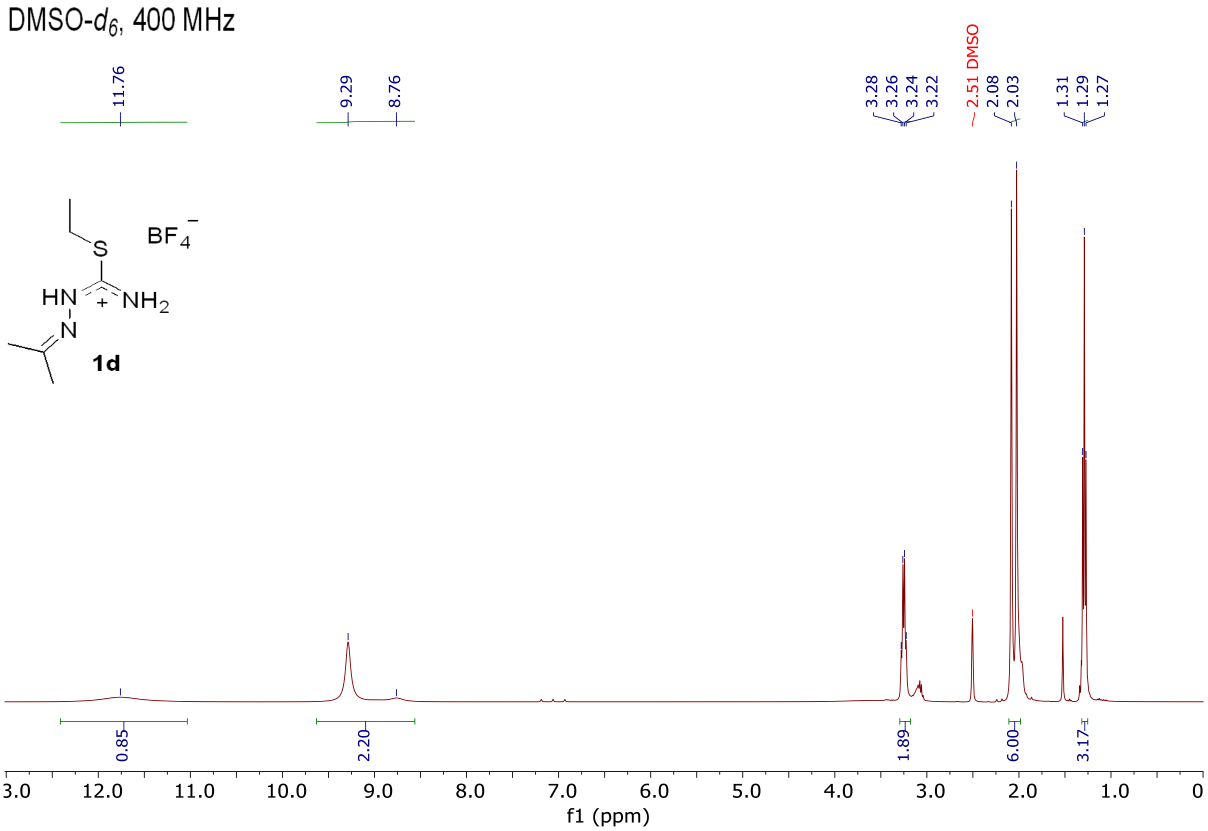

Figure S28. ^{1}H NMR spectrum (DMSO-d_6, 400 MHz) of **1d.**

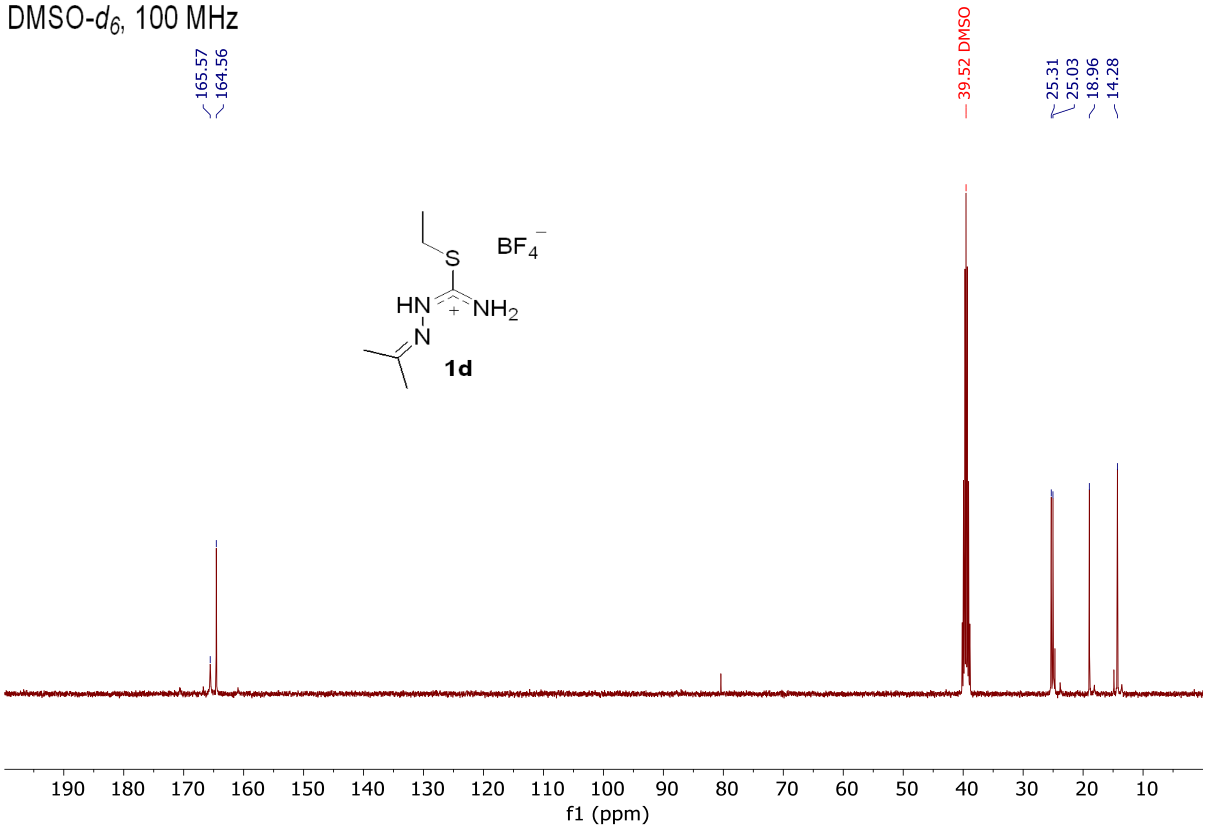

Figure S29. ^{13}C{^{1}H} NMR spectrum (DMSO-d_6, 100 MHz) of **1d.**

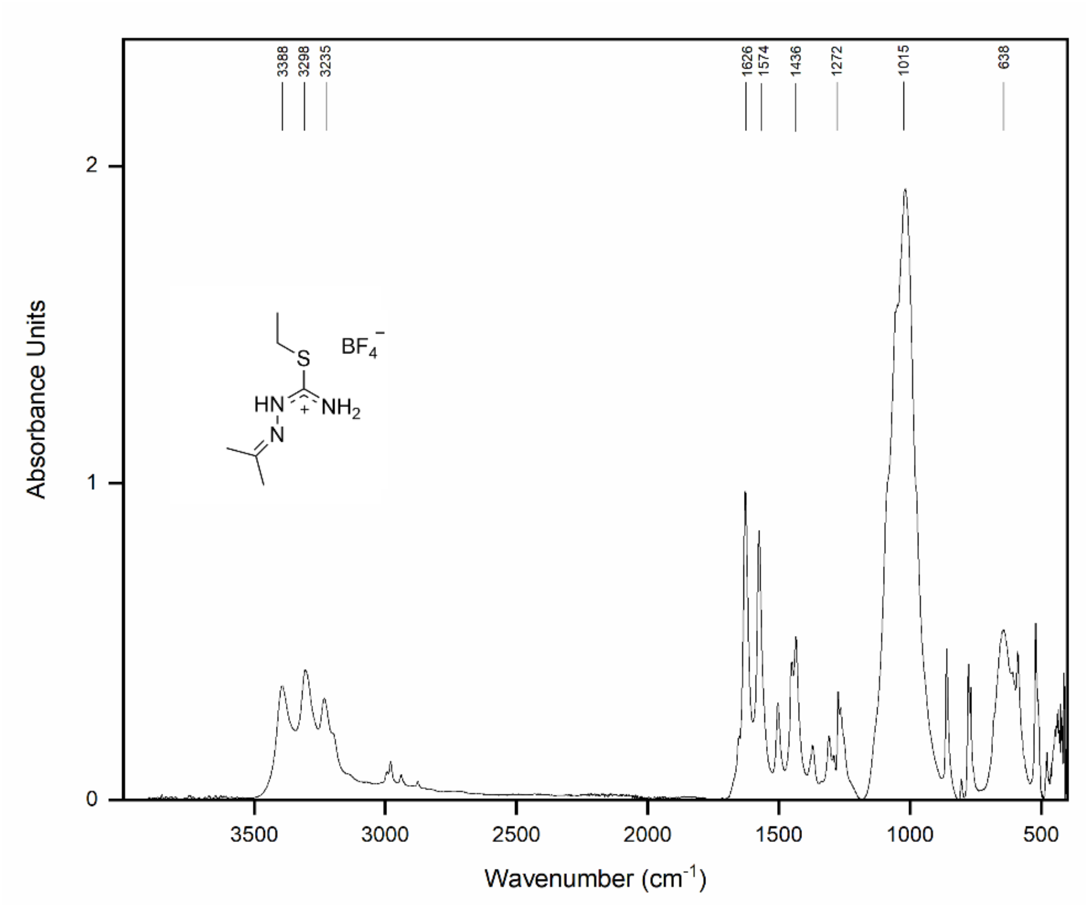

Figure S30. IR- absorption spectrum (neat) of **1d.**

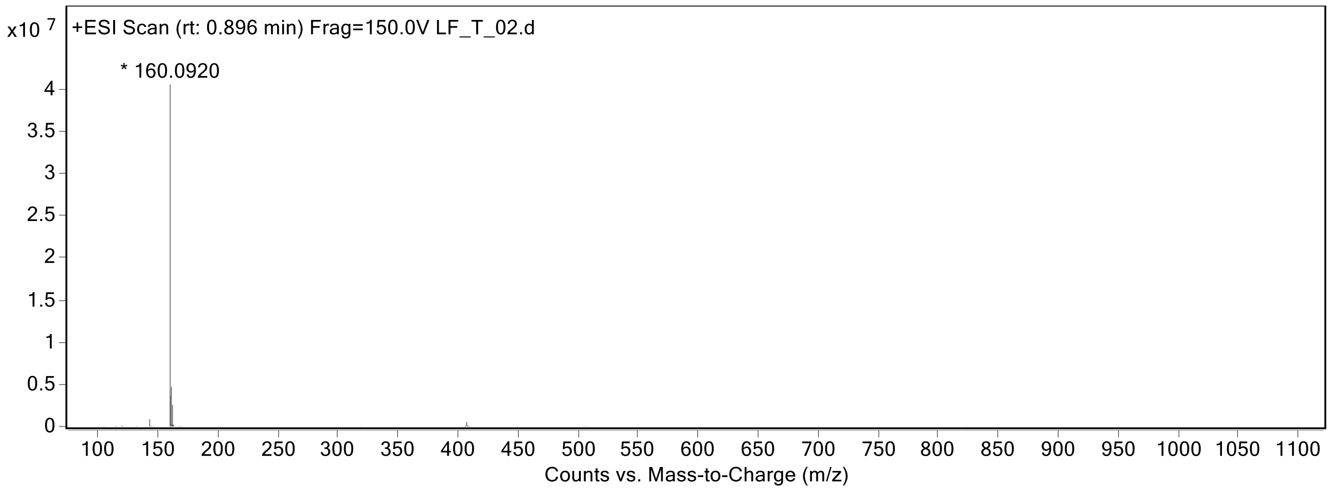

Figure S31. HR-ESI-MS (*m/z*) of **1d**. Measured peak for $[C_6H_{14}N_3S_1]^+$

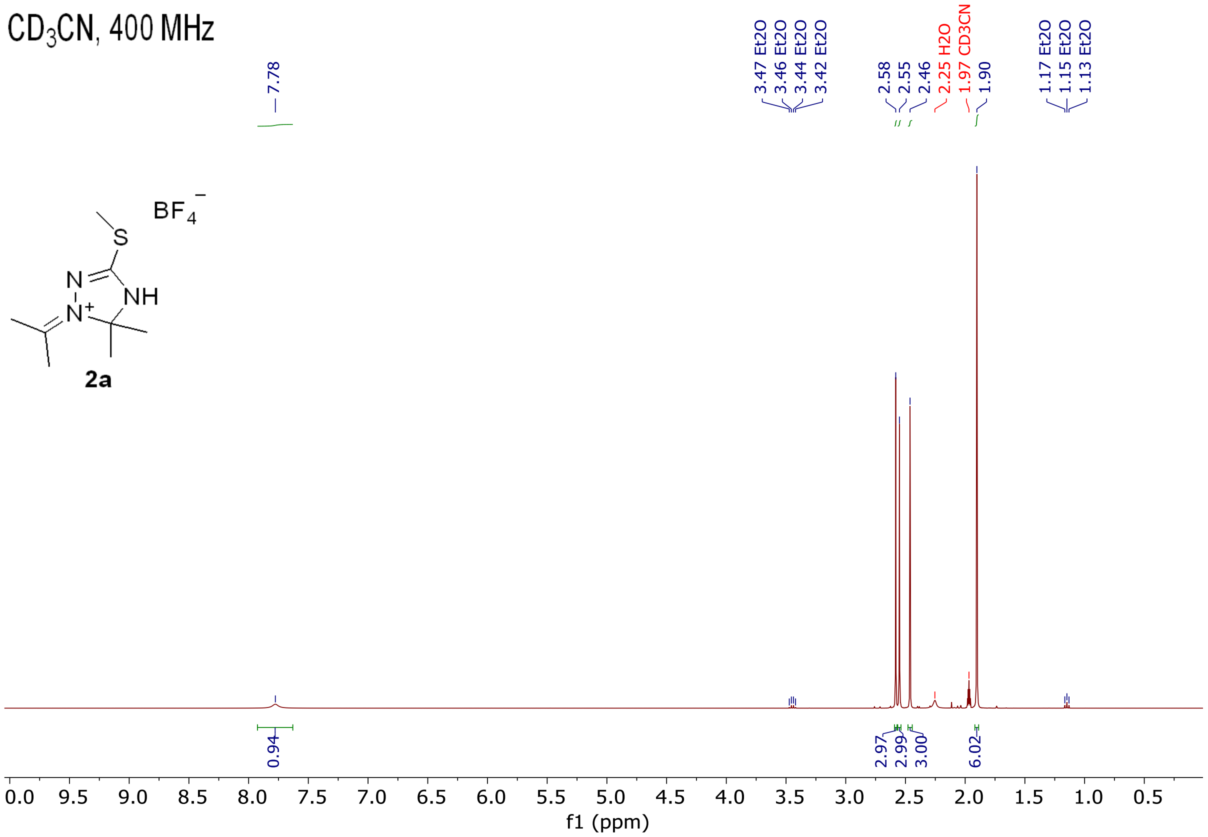

Figure S32. ^{1}H NMR spectrum (acetonitrile-d_3, 400 MHz) of **2a**

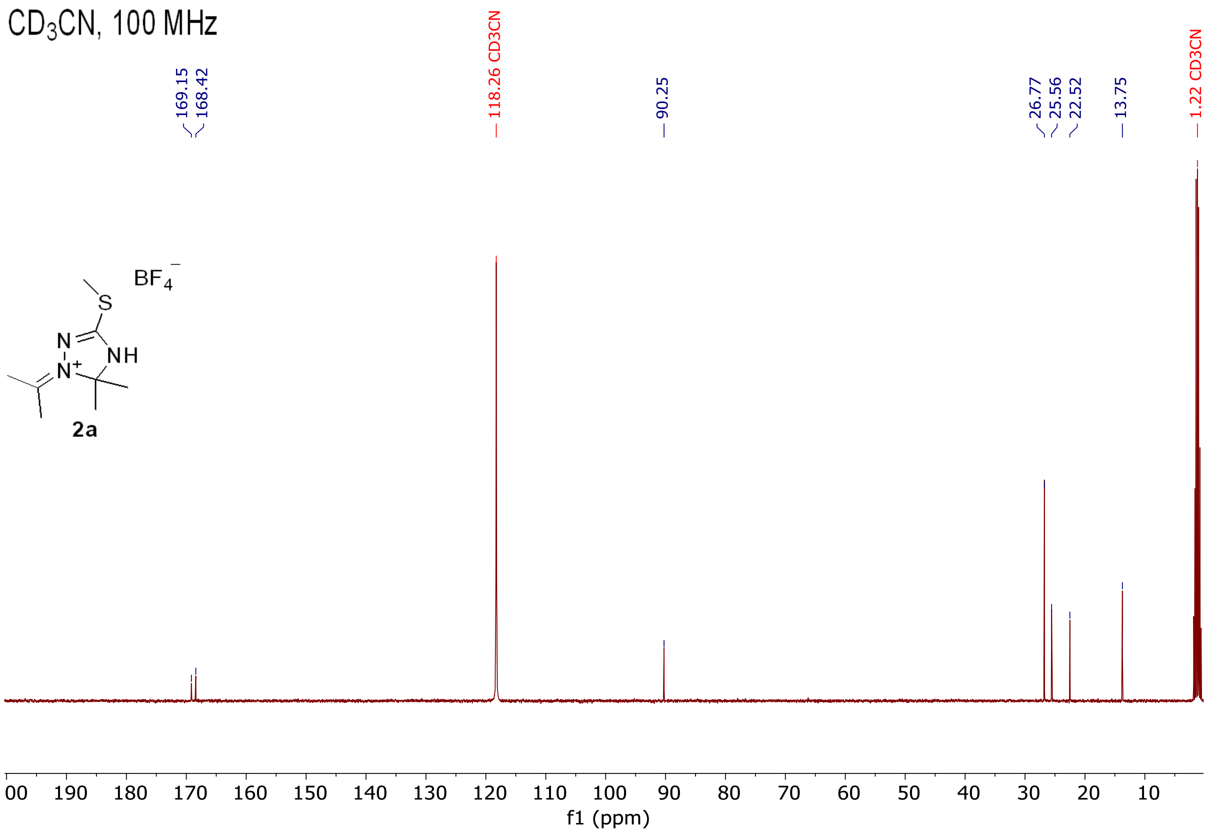

Figure S33. ^{13}C{^{1}H} NMR spectrum (acetonitrile-d_3, 100 MHz) of **2a**.

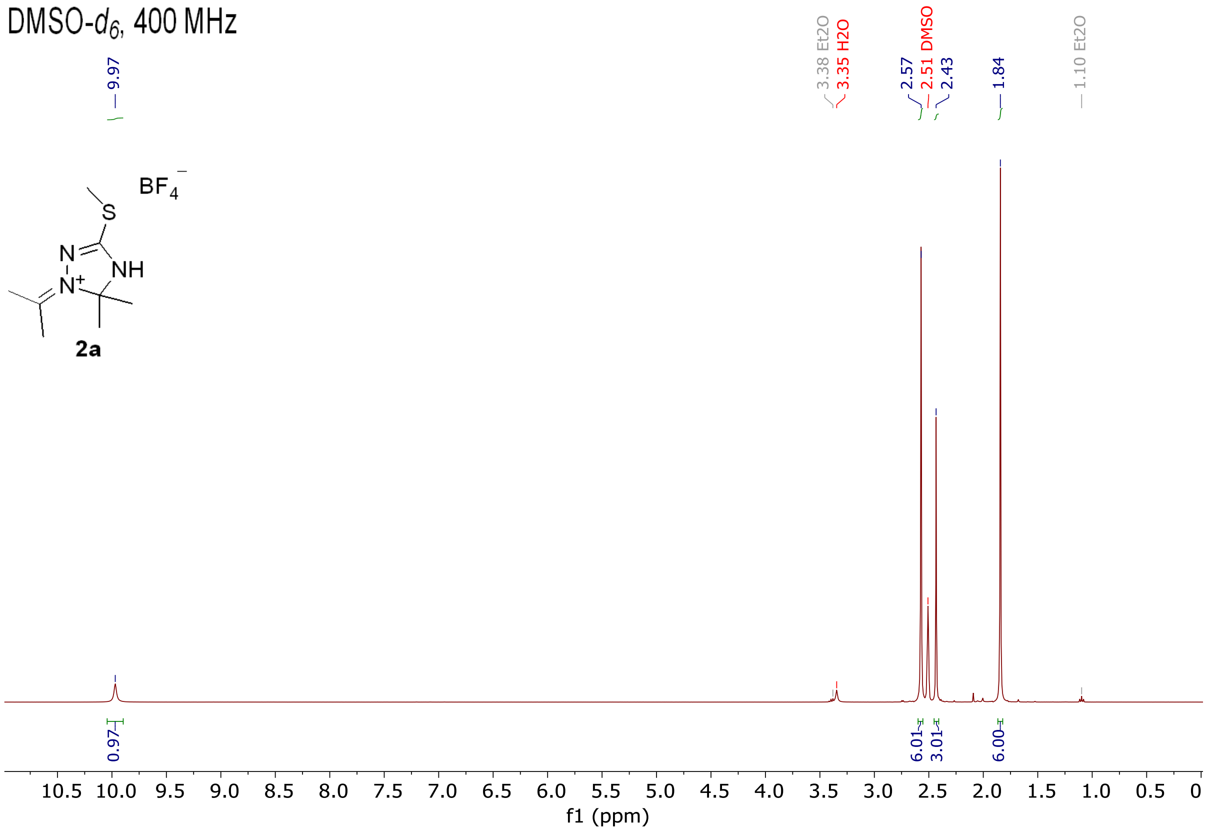

Figure S34. ^{1}H NMR spectrum (DMSO-d_6, 400 MHz) of **2a**.

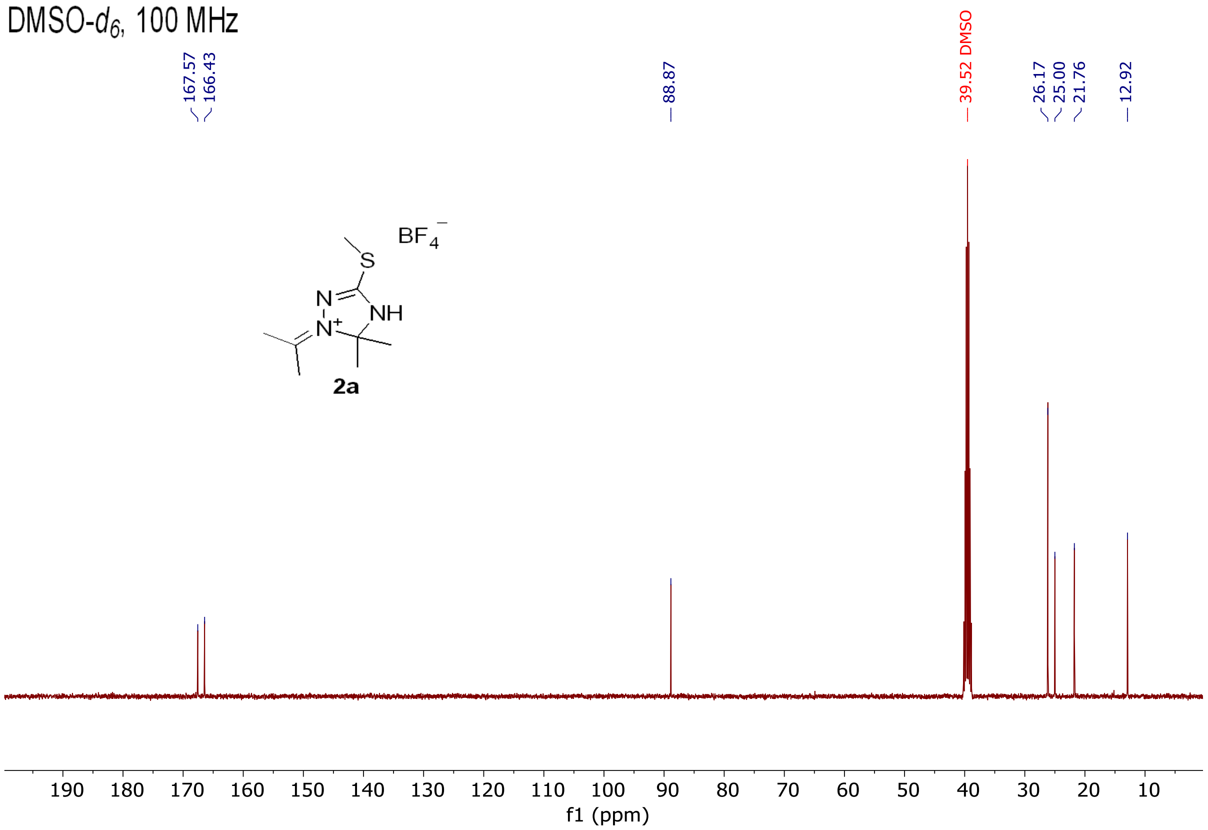

Figure S35. $^{13}C\{^{1}H\}$ NMR spectrum (DMSO-d_6, 100 MHz) of **2a.**

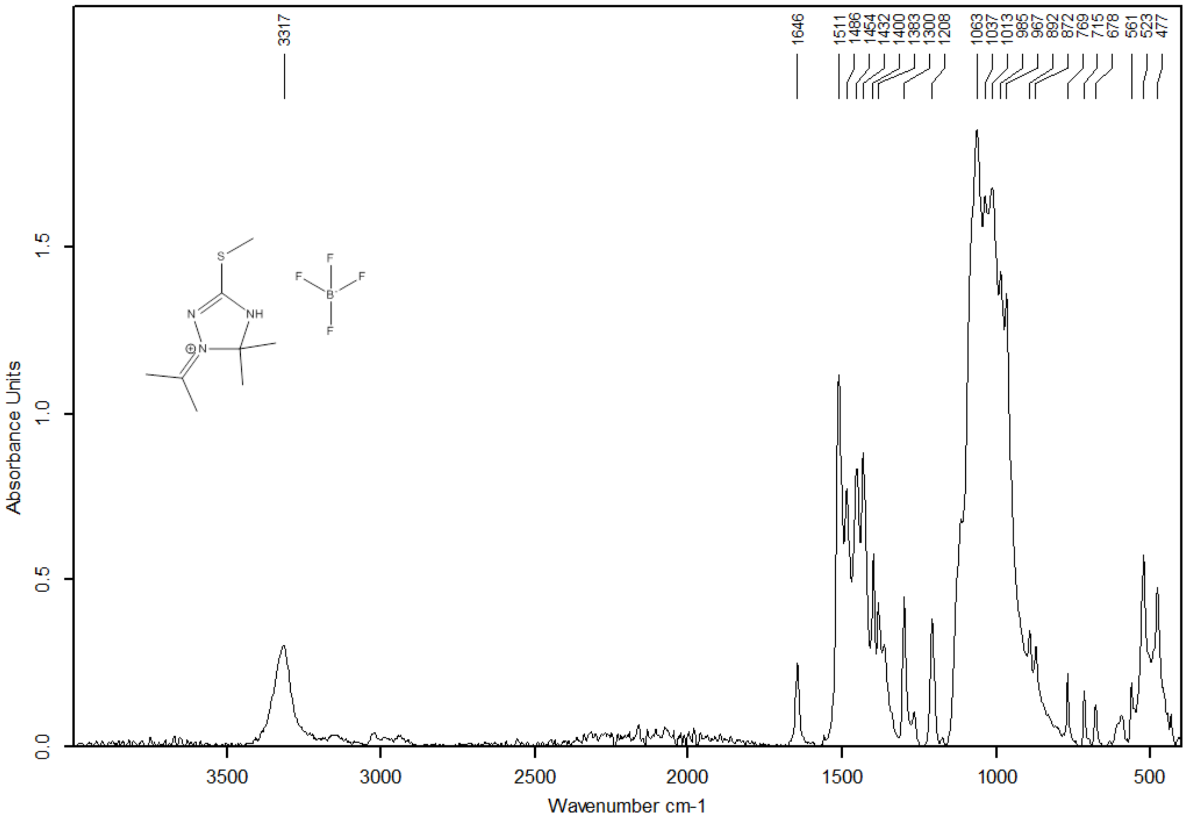

Figure S36. IR- absorption spectrum (neat) **2a**.

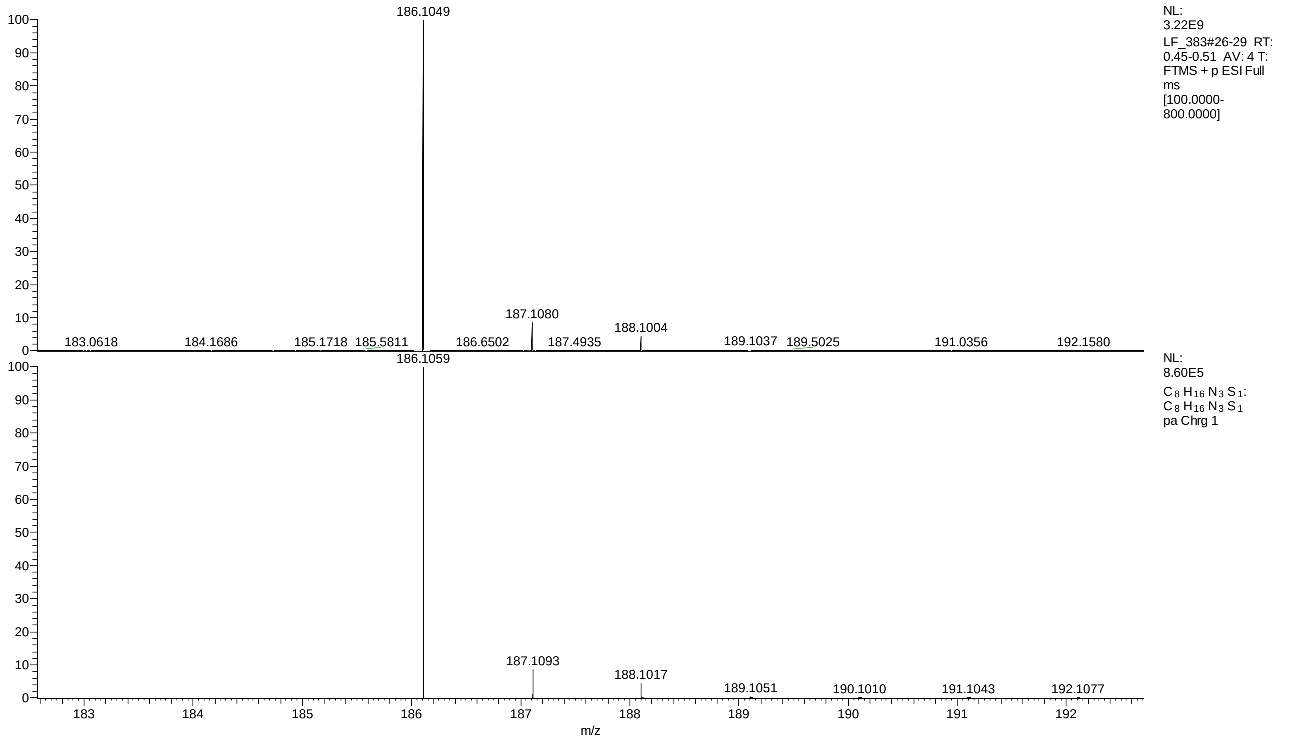

Figure S37. HR-ESI-MS (*m/z*) **2a**. Top: measured peaks for $[C_8H_{16}N_3S_1]^+$; Bottom: calculated peaks for $[C_8H_{16}N_3S_1]^+$

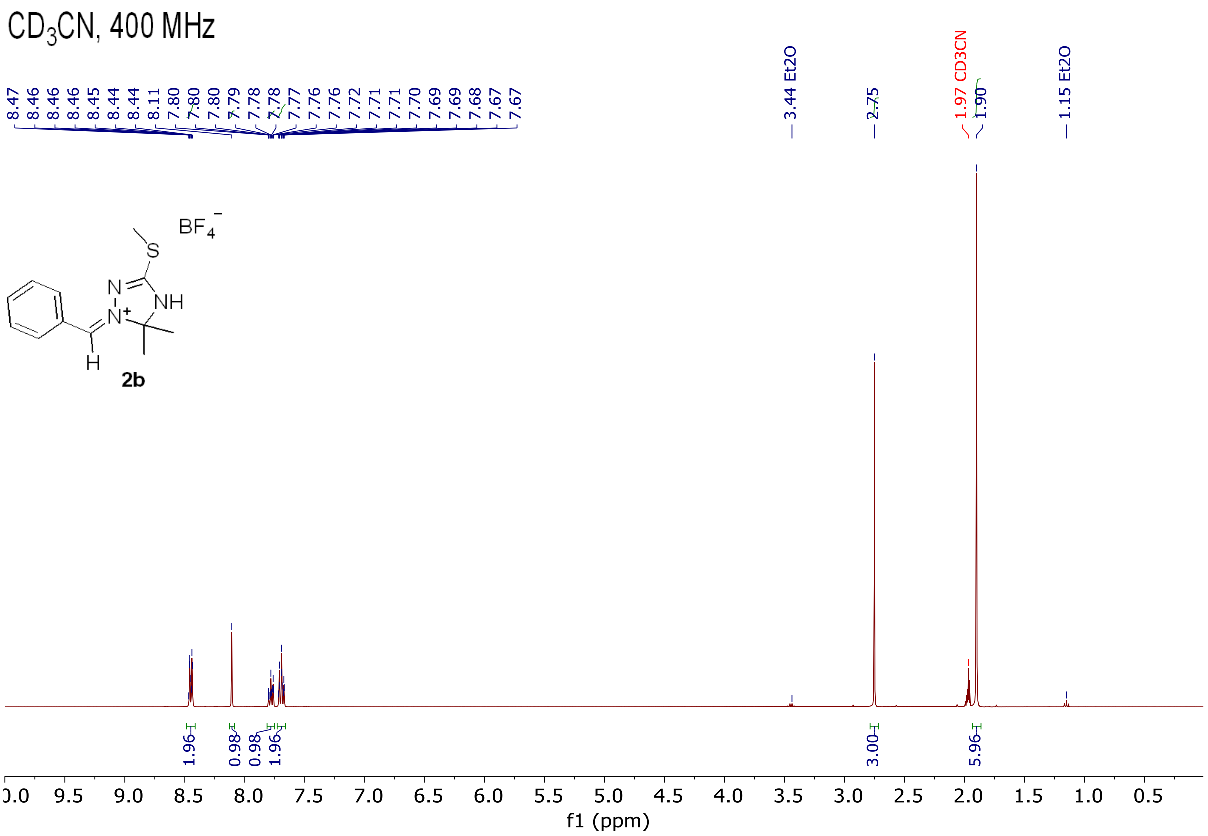

Figure S38. ^{1}H NMR spectrum (acetonitrile-d_3, 400 MHz) of **2b.**

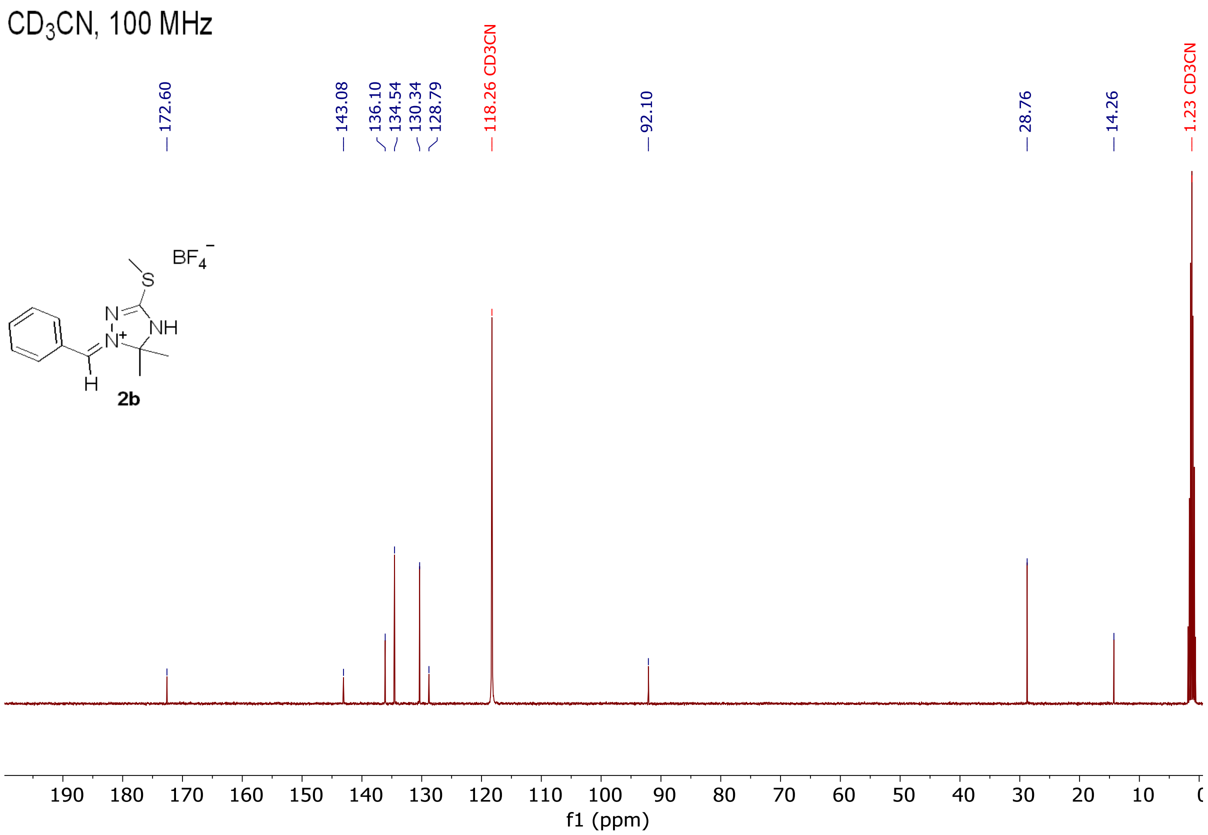

Figure S39. $^{13}C\{^{1}H\}$ NMR spectrum (acetonitrile-d_3, 100 MHz) of **2b**.

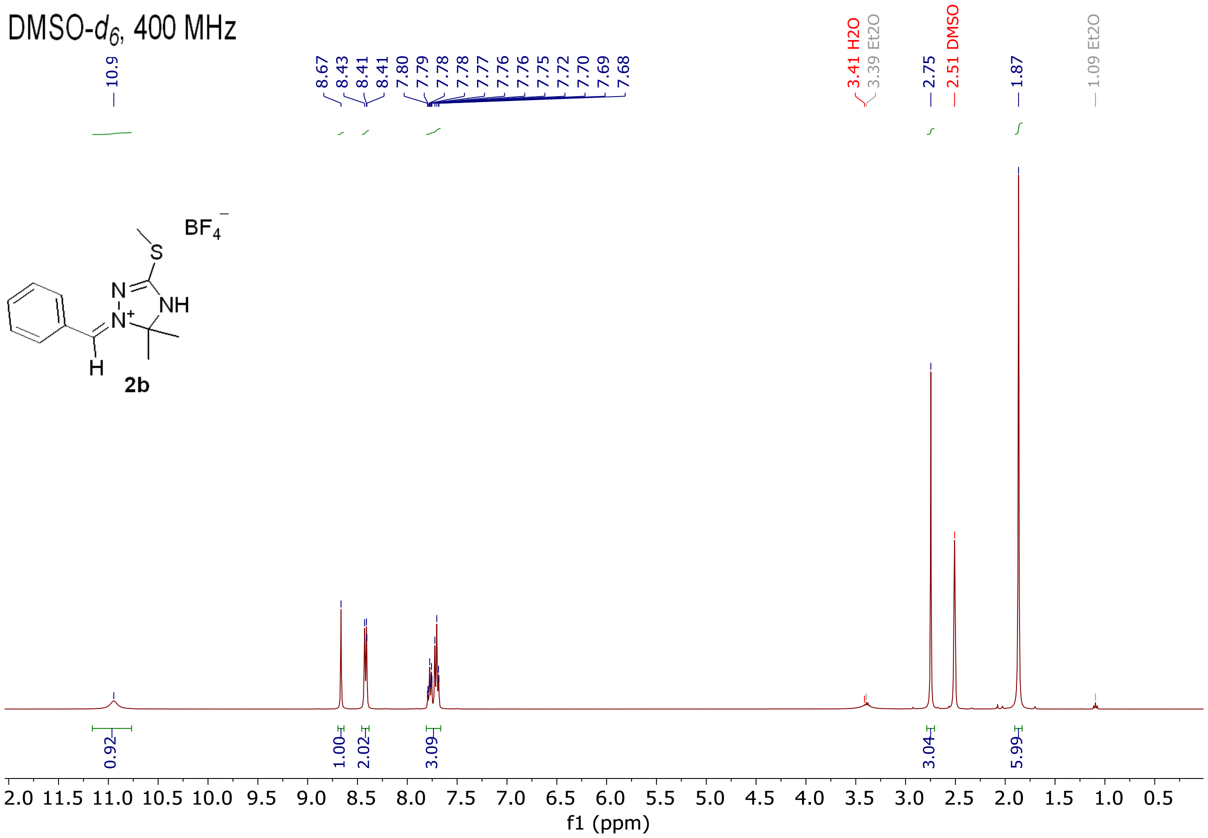

Figure S40. ^{1}H NMR spectrum (DMSO-d_6, 400 MHz) of **2b**.

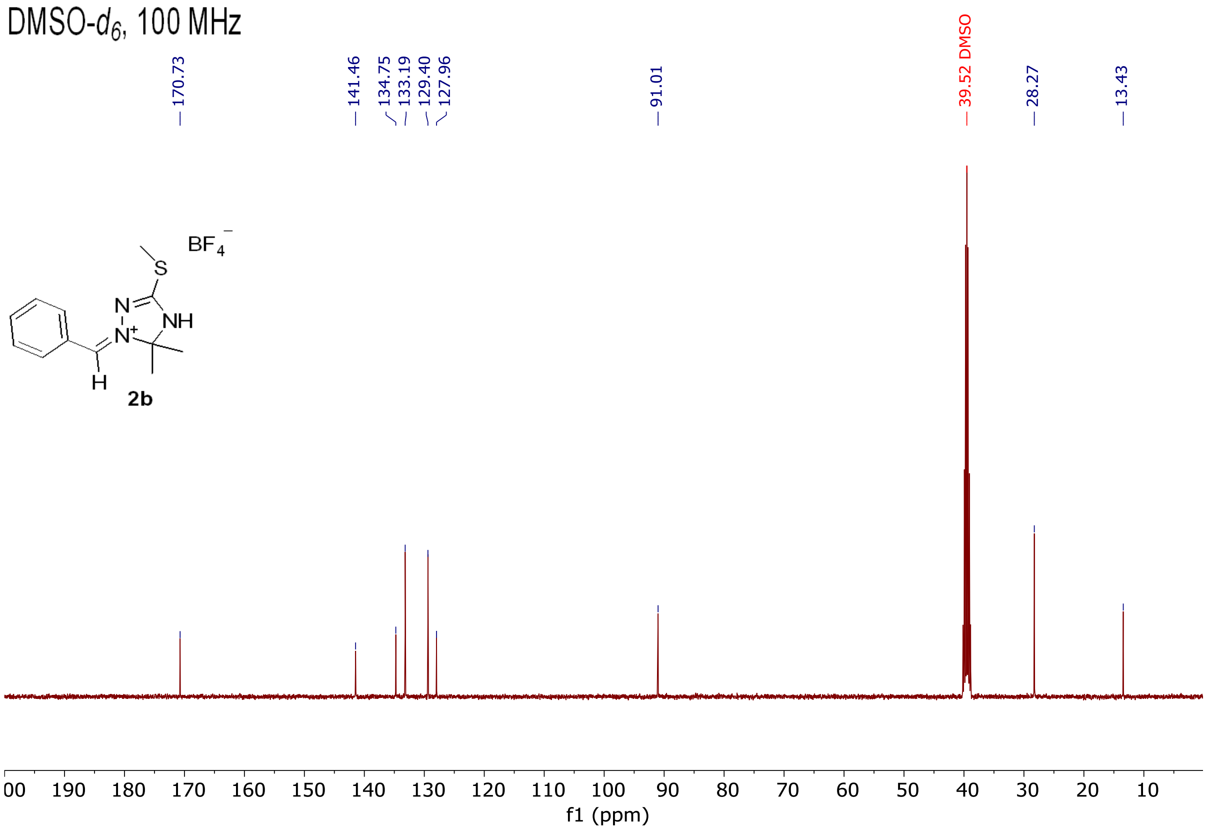

Figure S41. ^{13}C{^{1}H} NMR spectrum (DMSO-d_6, 100 MHz) of **2b.**

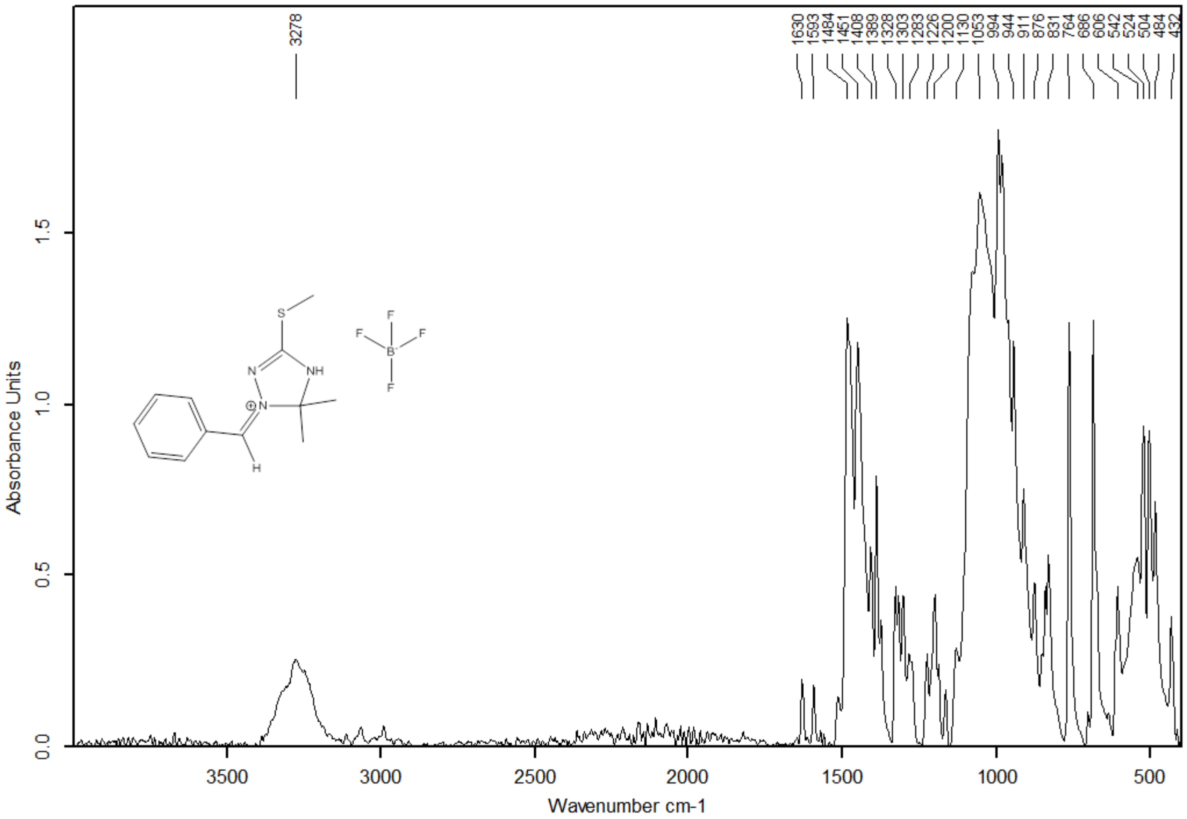

Figure S42. IR- absorption spectrum (neat) of **2b**

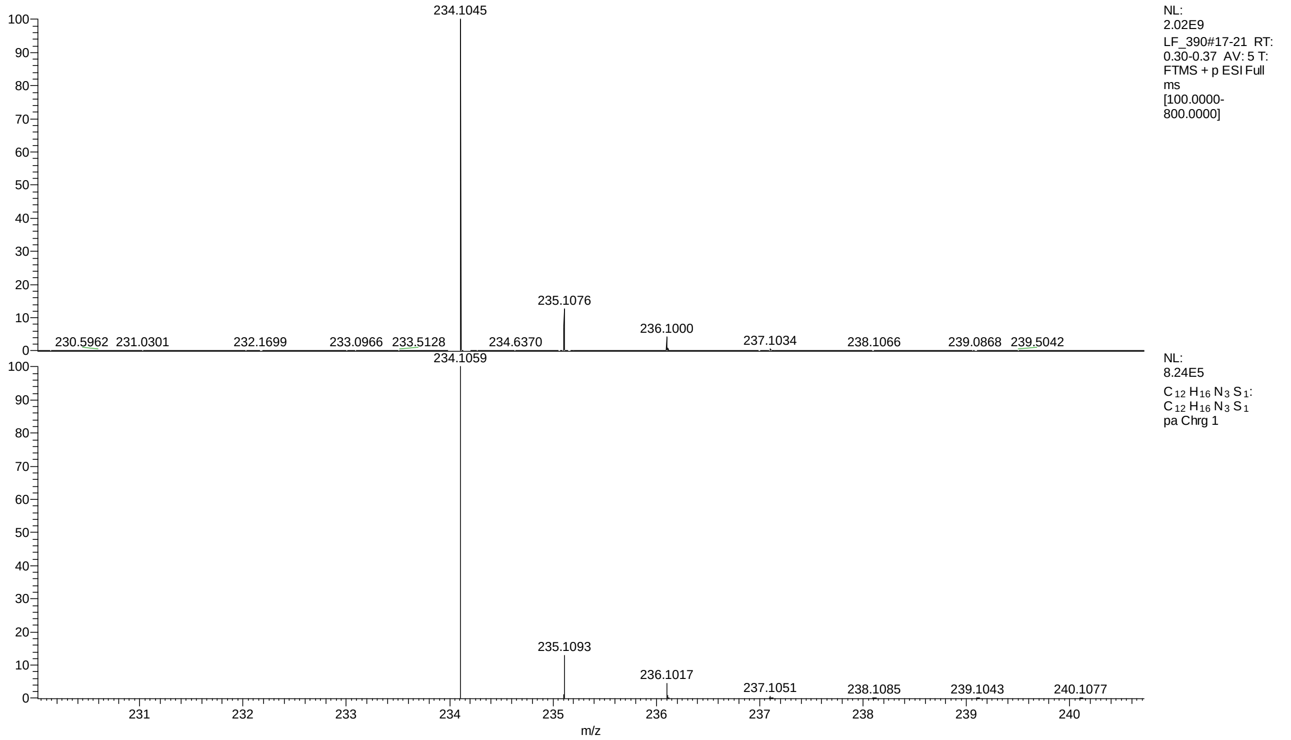

Figure S43. HR-ESI-MS (*m/z*) of **2b**. Top: measured peaks for $[C_{12}H_{16}N_3S_1]^+$; Bottom: calculated peaks for $[C_{12}H_{16}N_3S_1]^+$

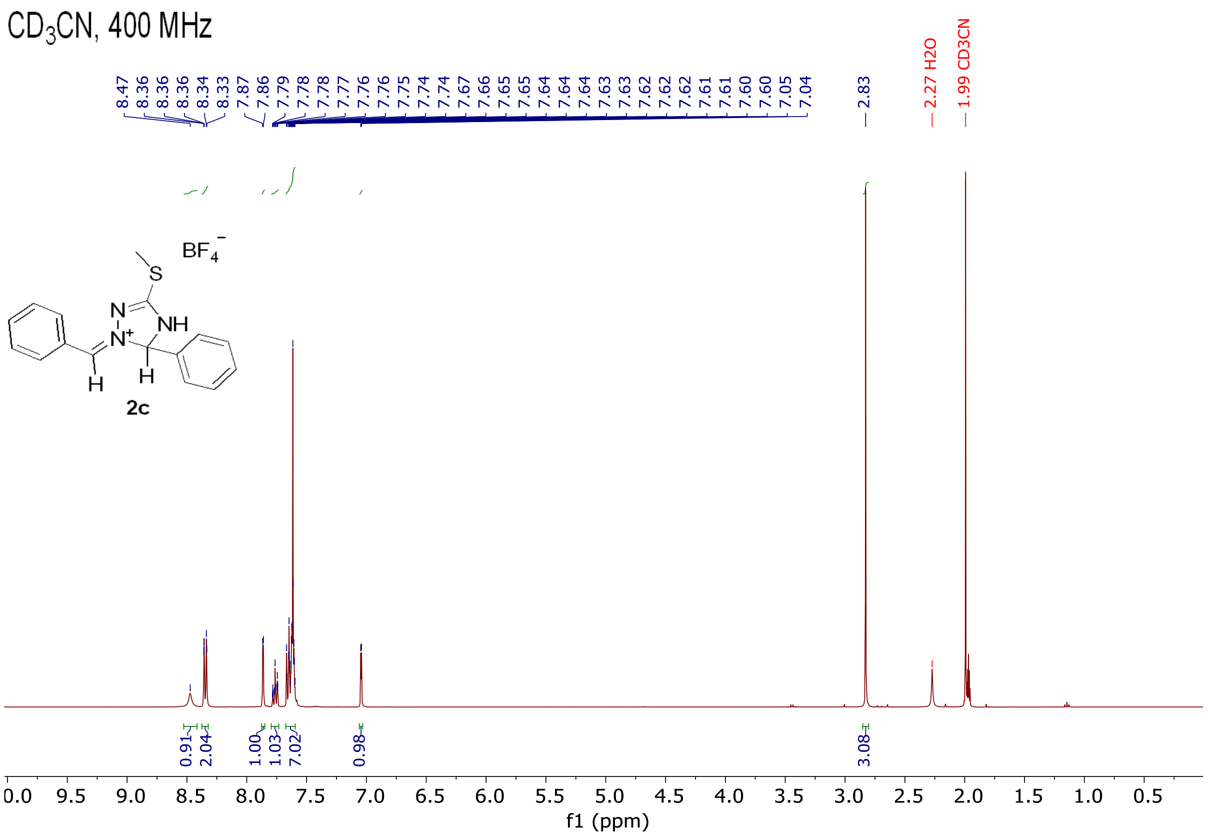

Figure S44. ^{1}H NMR spectrum (acetonitrile-d_3, 400 MHz) of **2c**.

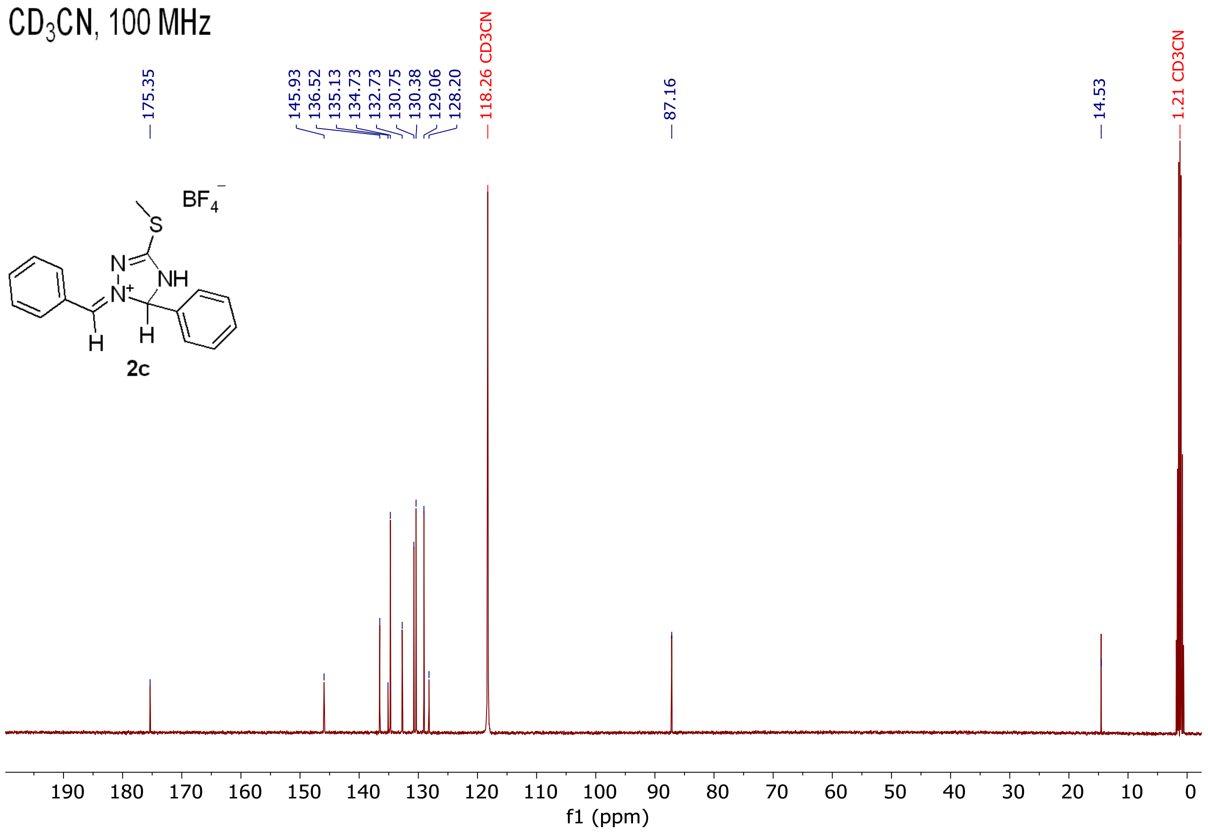

Figure S45. $^{13}C\{^{1}H\}$ NMR spectrum (acetonitrile-d_3, 100 MHz) of **2c**.

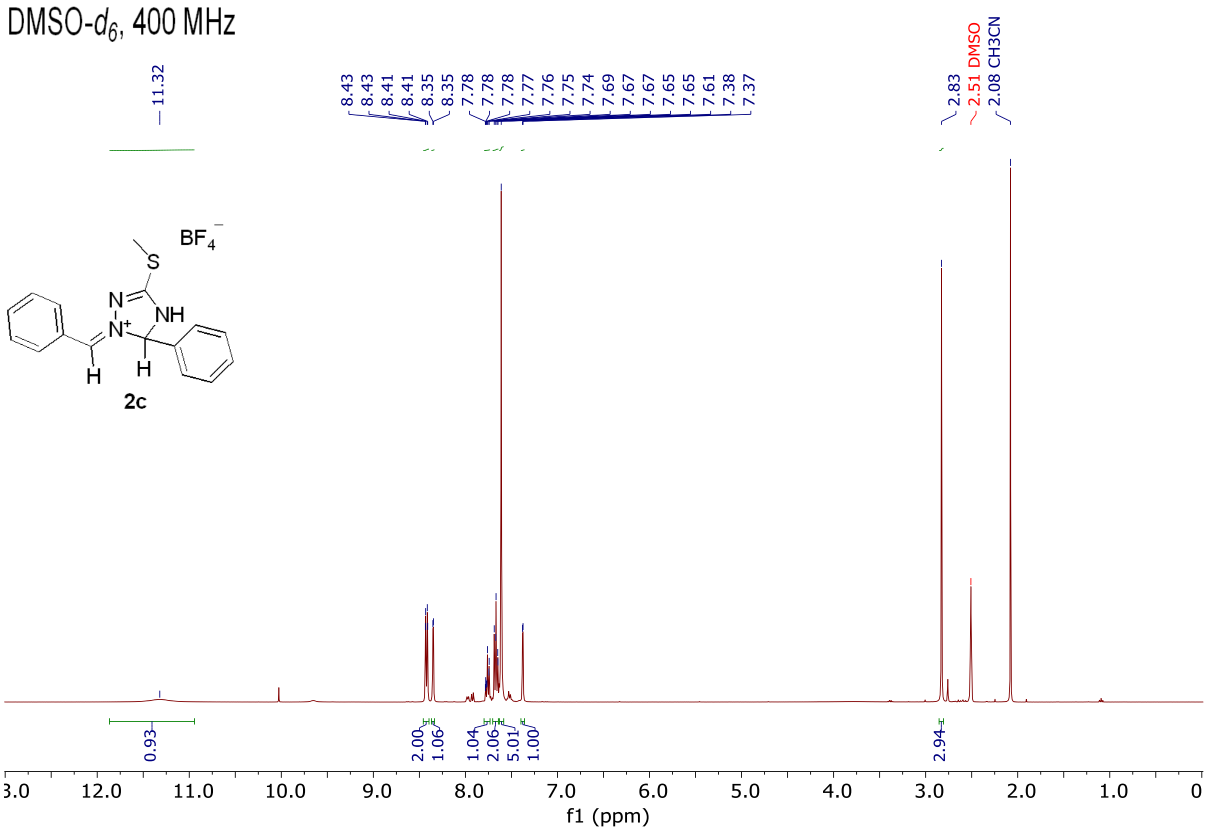

Figure S46. ^{1}H NMR spectrum (DMSO-d_6, 400 MHz) of **2c**. Signs of slight dissociation likely due to H_2O contaminations of the used DMSO-d_6 are visible.

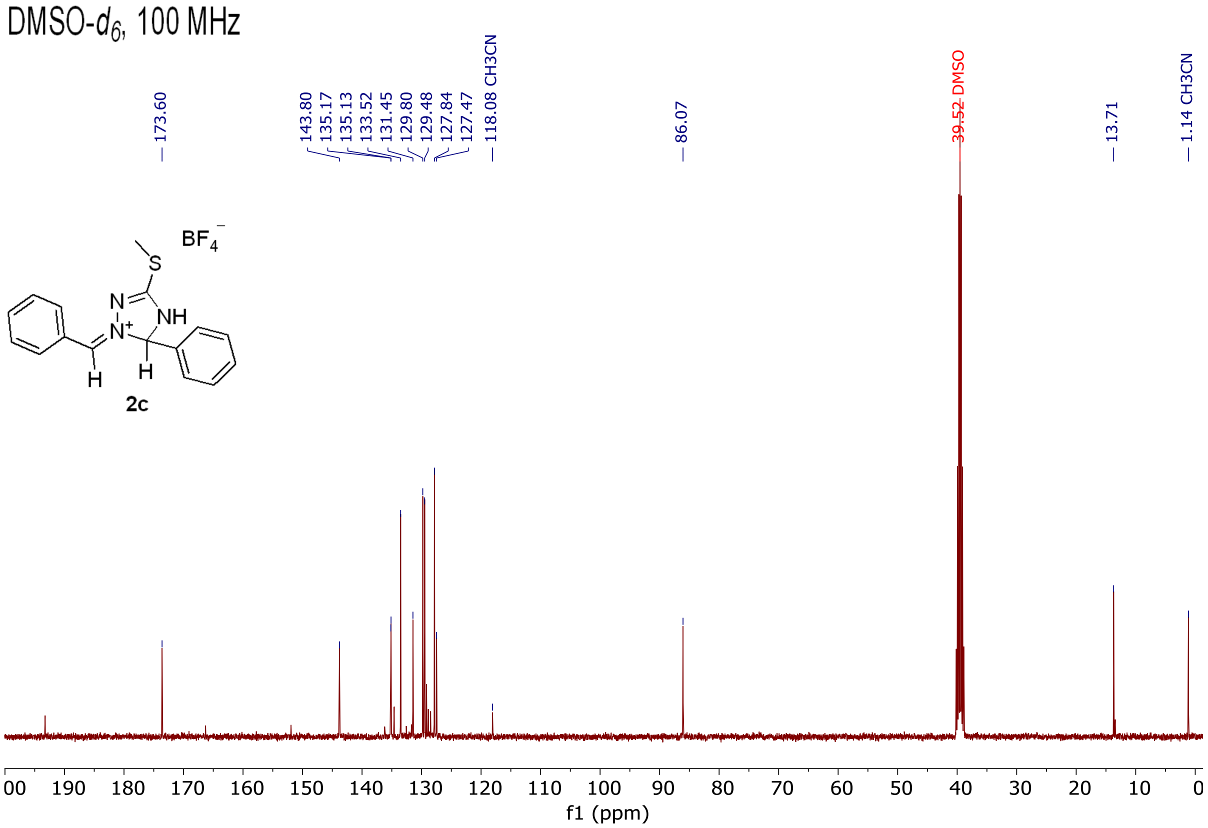

Figure S47. $^{13}C\{^{1}H\}$ NMR spectrum (DMSO-d_6, 100 MHz) **2c**. Signs of slight dissociation likely due to H_2O contaminations of the used DMSO-d_6 are visible.

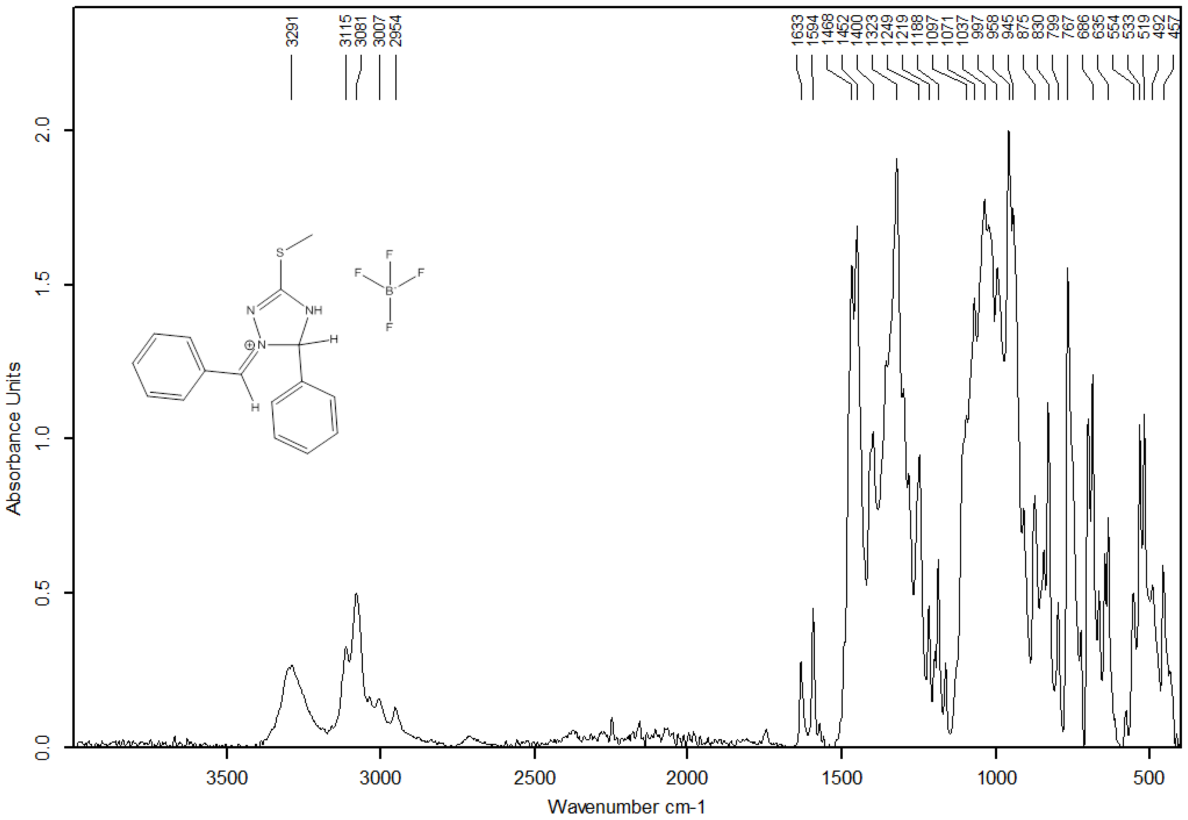

Figure S48. IR- absorption spectrum (neat) of **2c**.

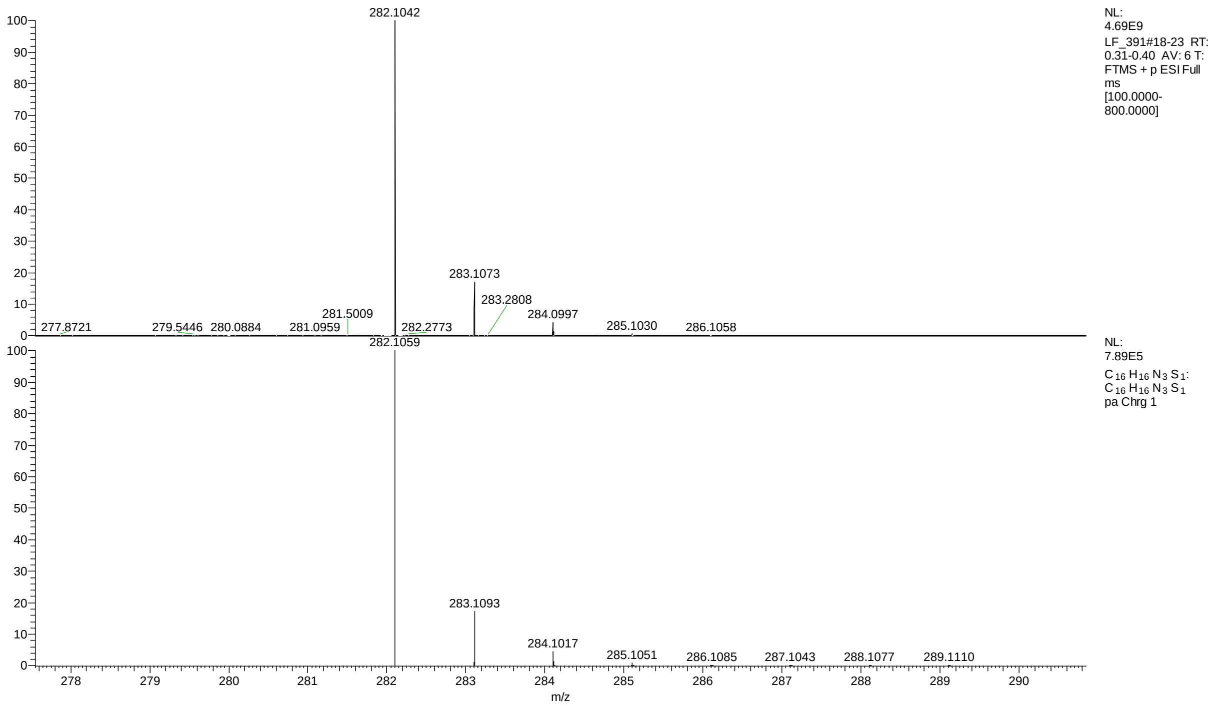

Figure S49. HR-ESI-MS (*m/z*) **2c**. Top: measured peaks for $[C_{16}H_{16}N_3S_1]^+$; Bottom: calculated peaks for $[C_{16}H_{16}N_3S_1]^+$

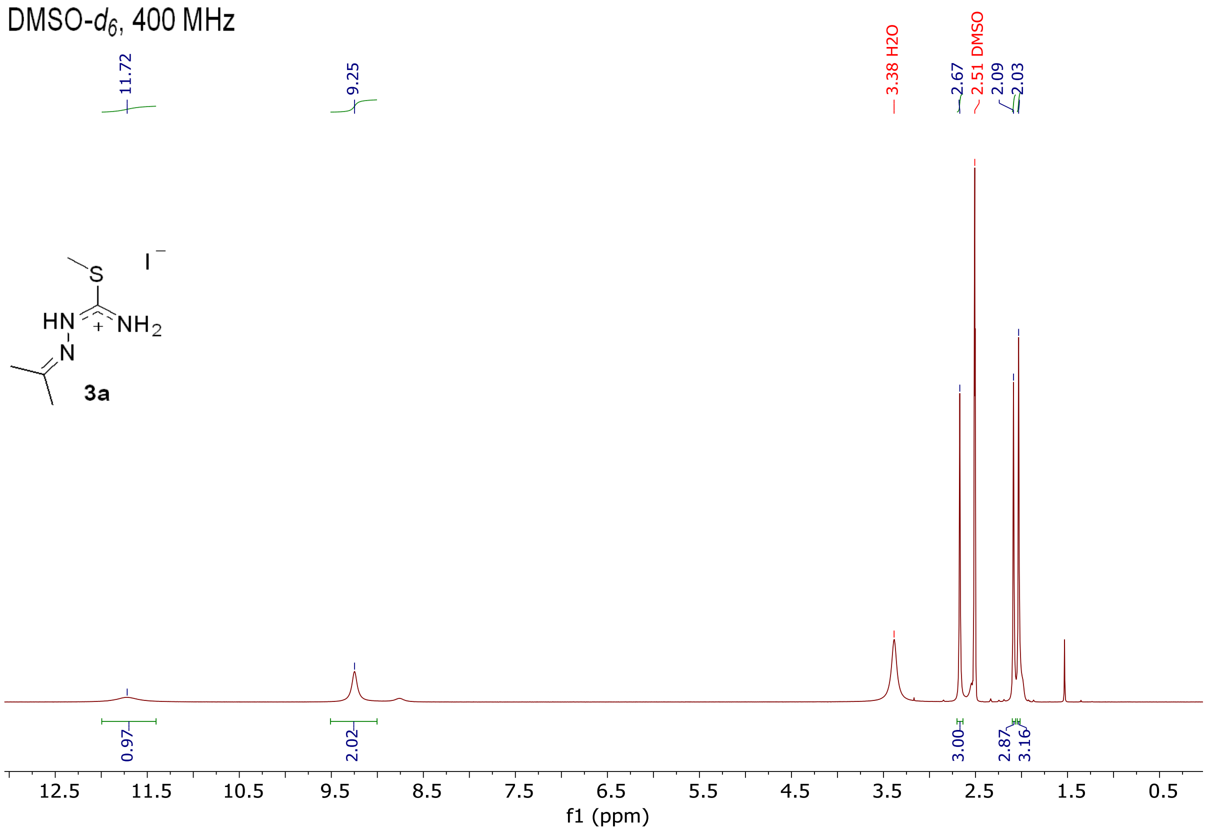

Figure S50. ^{1}H NMR spectrum (DMSO-d_6, 400 MHz) of **3a**.

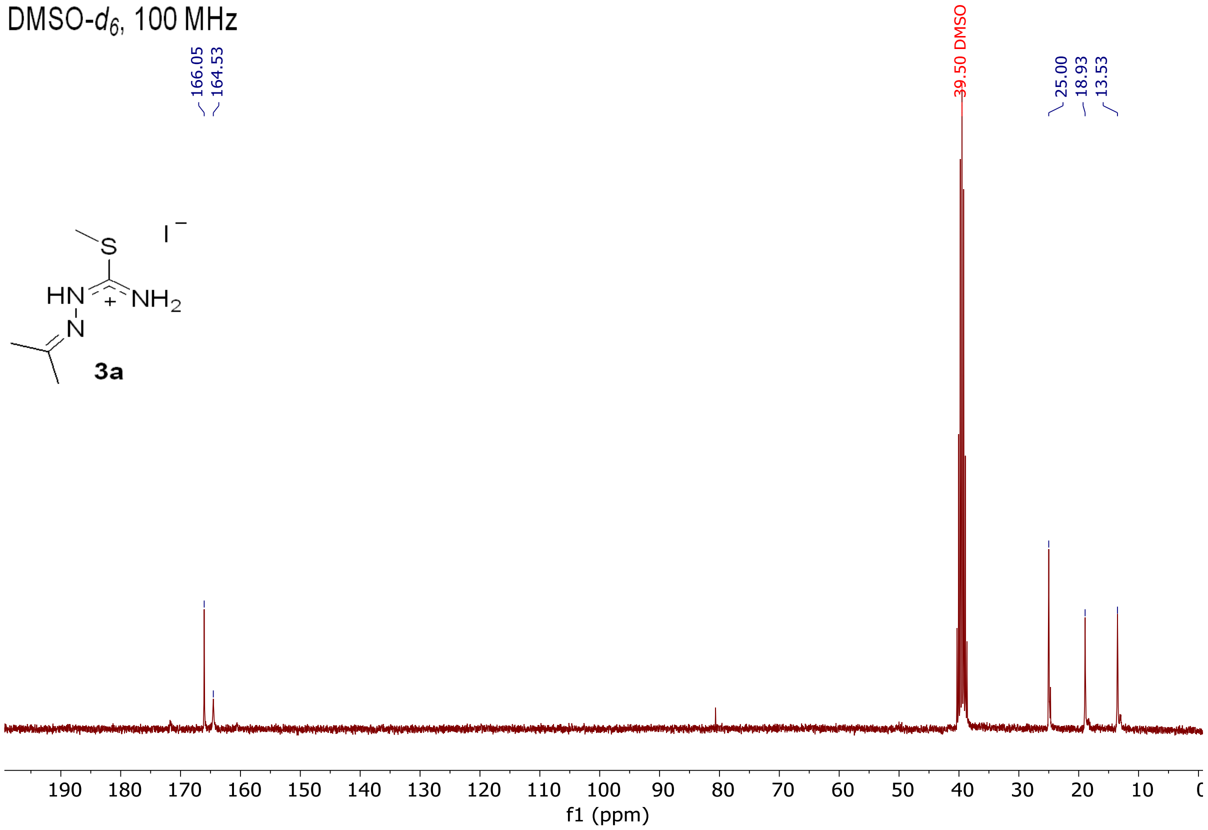

Figure S51. $^{13}C\{^{1}H\}$ NMR spectrum (DMSO-d_6, 100 MHz) of **3a**.

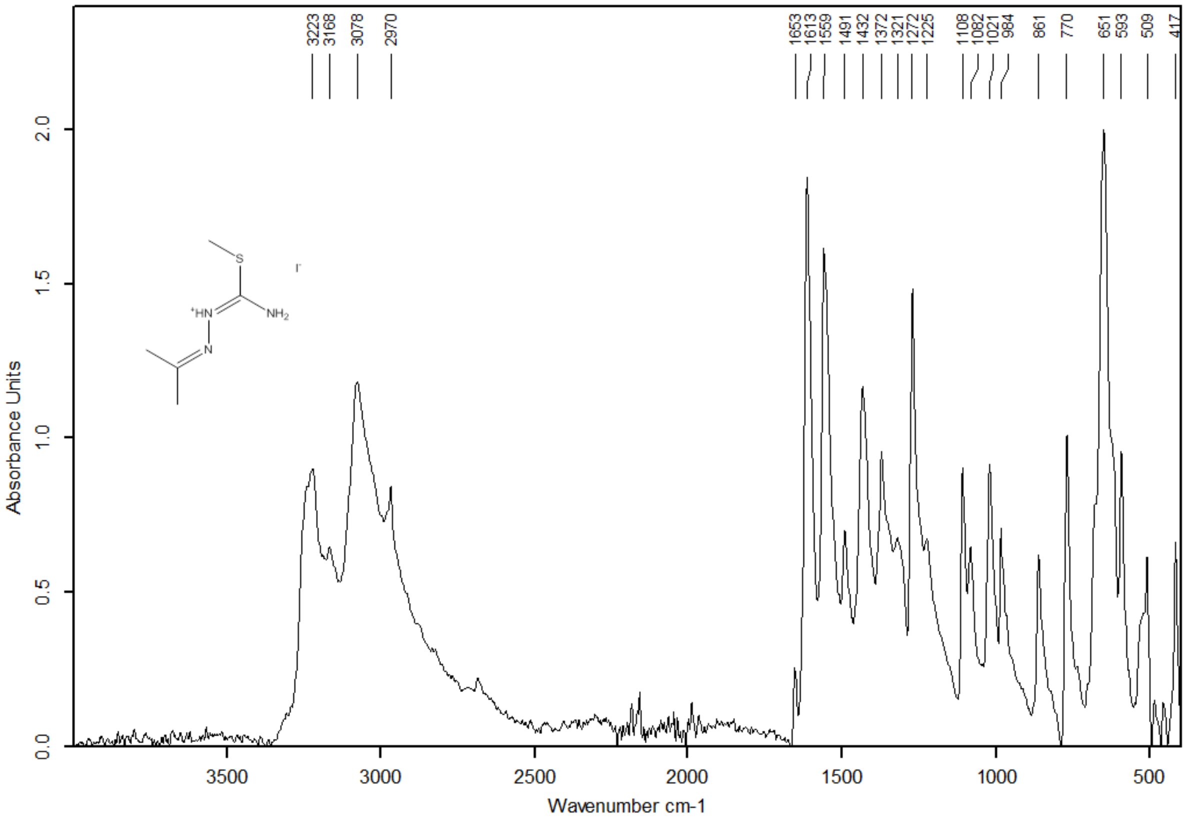

Figure S52. IR- absorption spectrum (neat) of **3a**.

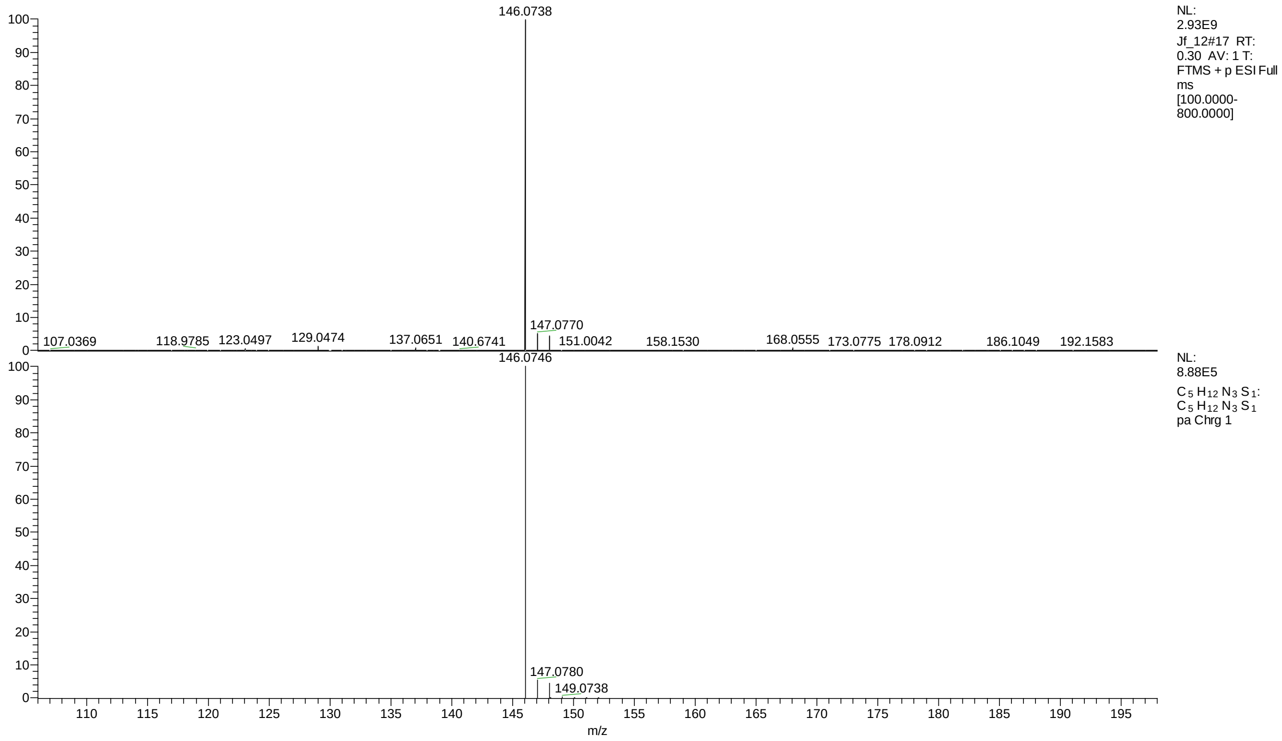

Figure S53. HR-ESI-MS (*m/z*) of **3a**. Top: measured peaks for $[C_5H_{12}N_3S_1]^+$; Bottom: calculated peaks for $[C_5H_{12}N_3S_1]^+$

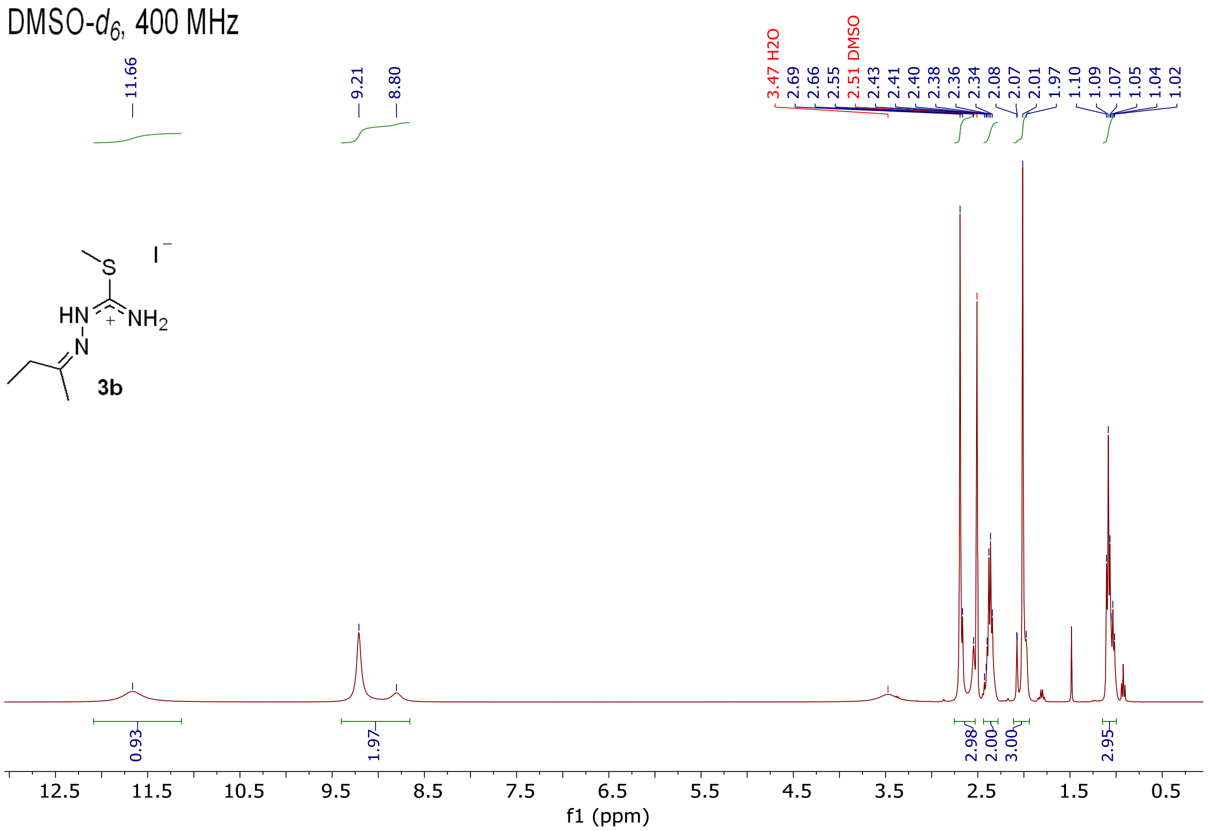

Figure S54. ^{1}H NMR spectrum (DMSO-d_6, 400 MHz) of **3b**.

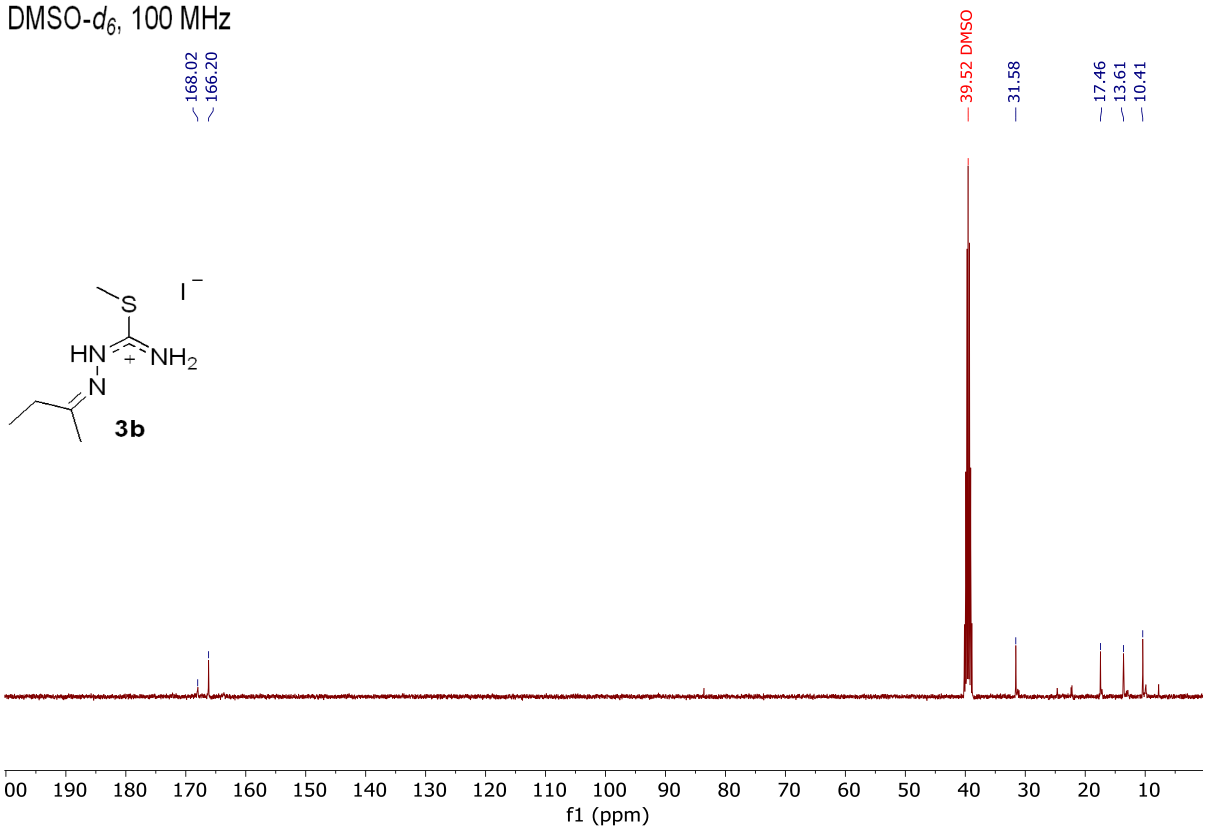

Figure S55. $^{13}C\{^{1}H\}$ NMR spectrum (DMSO-d_6, 100 MHz) of **3b**.

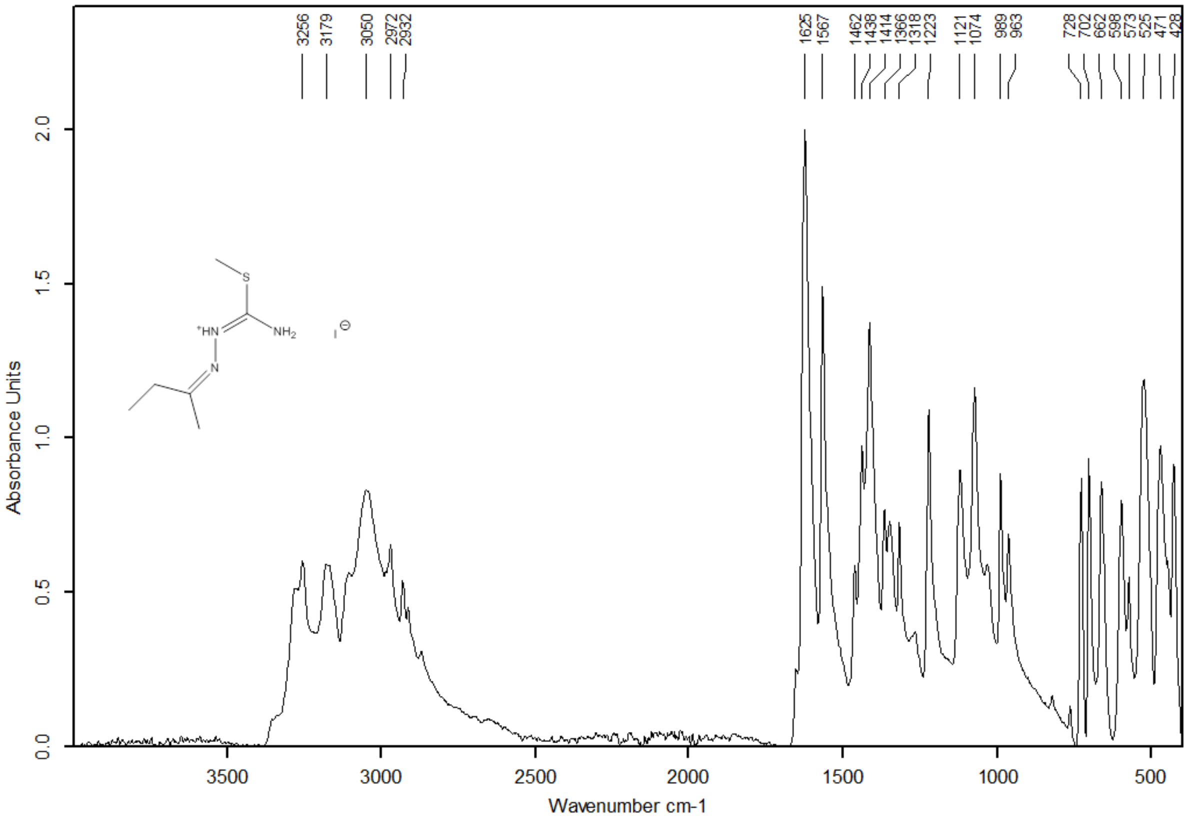

Figure S56. IR- absorption spectrum (neat) of **3b**.

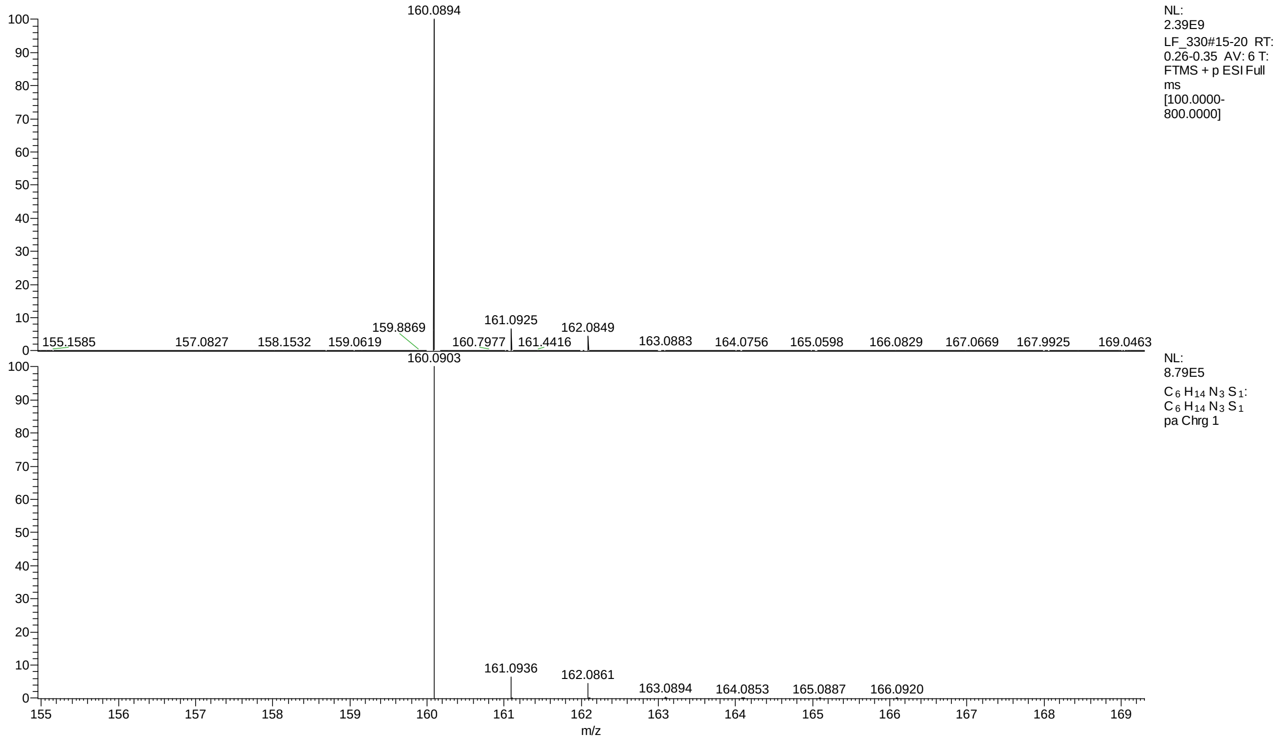

Figure S57. HR-ESI-MS (*m/z*) of **3b**. Top: measured peaks for $[C_6H_{14}N_3S_1]^+$; Bottom: calculated peaks for $[C_6H_{14}N_3S_1]^+$

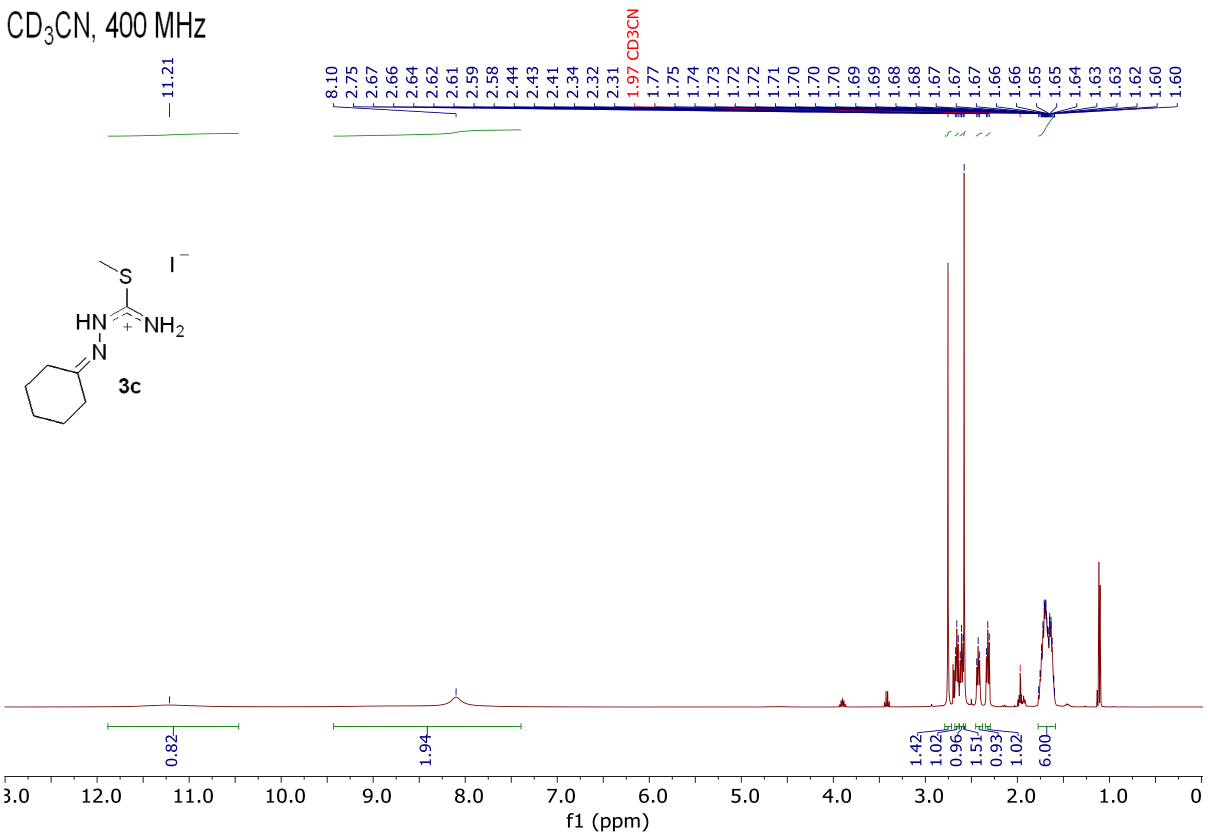

Figure S58. ^{1}H NMR spectrum (acetonitrile-d_3, 400 MHz) of **3c**.

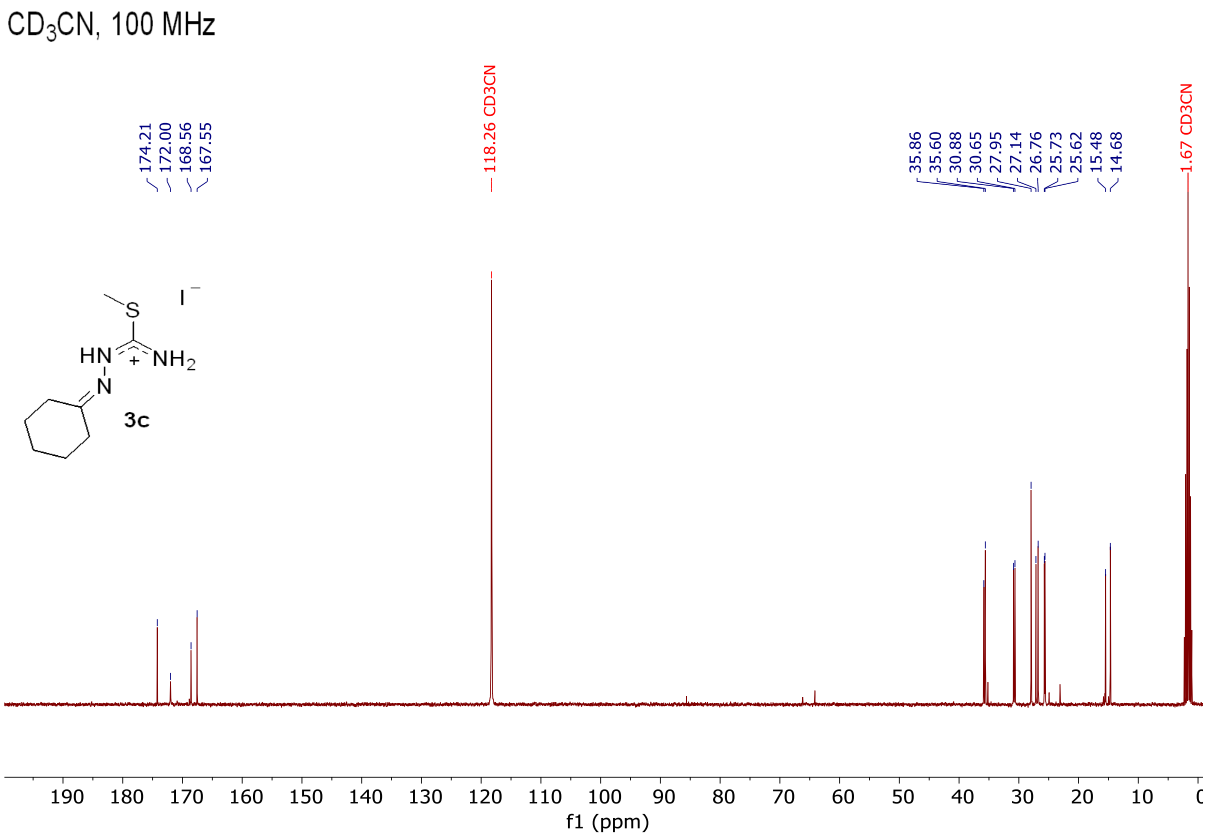

Figure S59. $^{13}C\{^{1}H\}$ NMR spectrum (acetonitrile-d_3, 100 MHz) of **3c**.

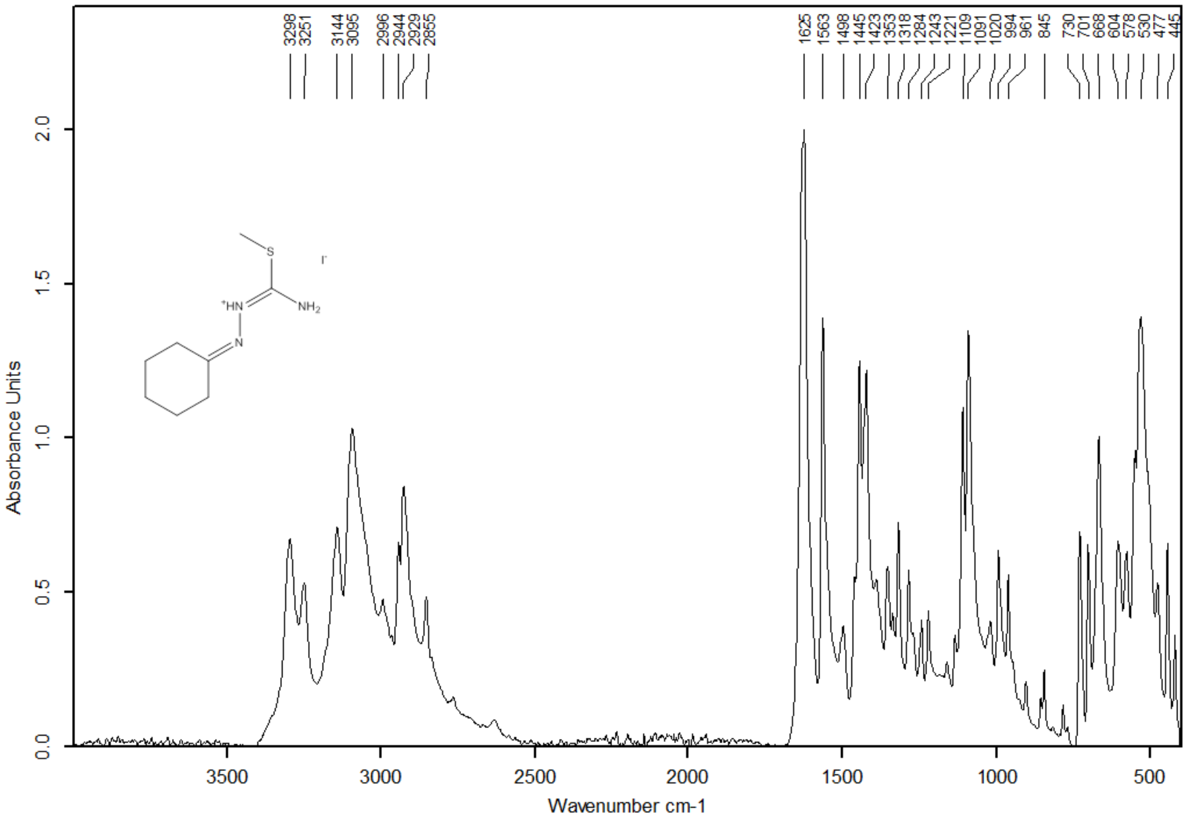

Figure S60. IR- absorption spectrum (neat) of **3c**.

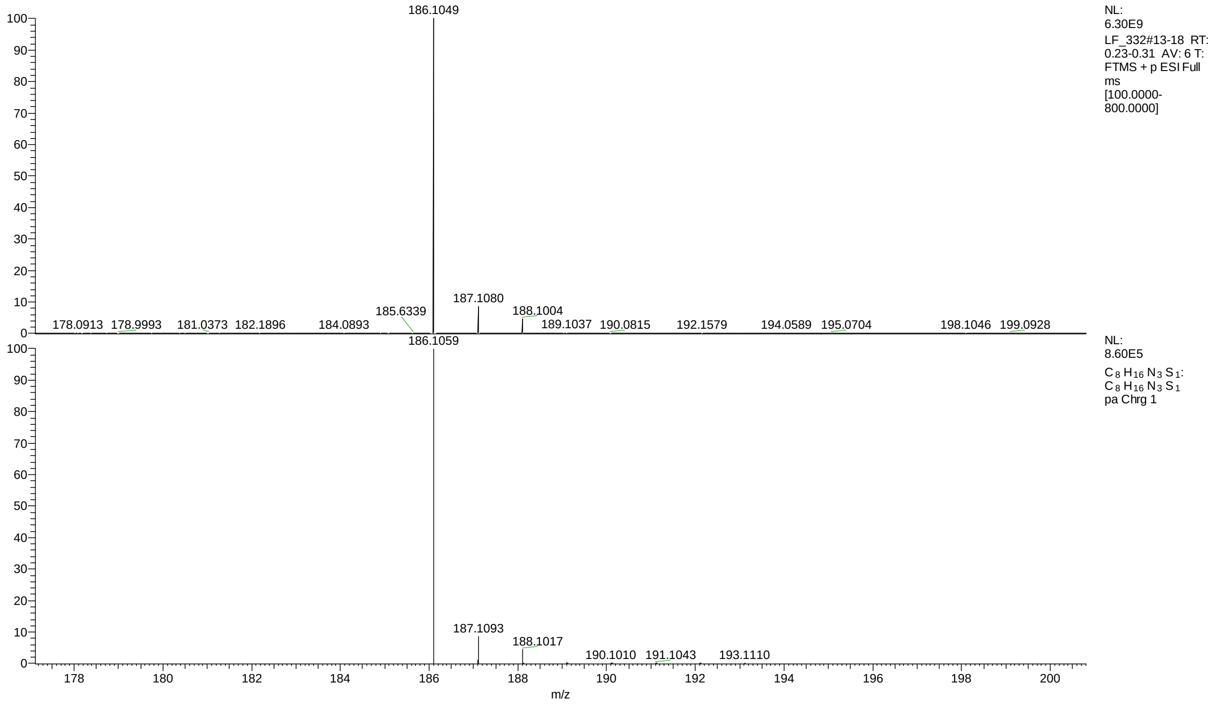

Figure S61. HR-ESI-MS (*m/z*) of **3c**. Top: measured peaks for $[C_8H_{16}N_3S_1]^+$; Bottom: calculated peaks for $[C_8H_{16}N_3S_1]^+$

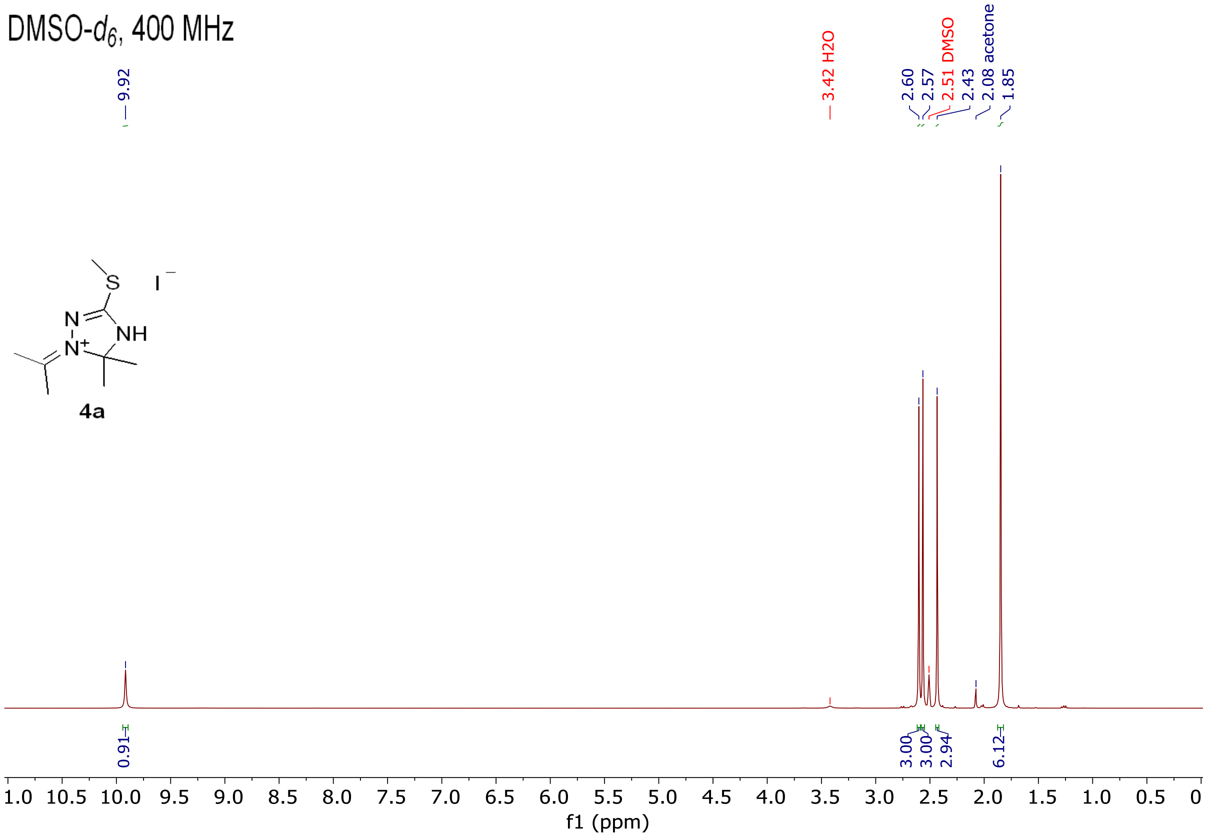

Figure S62. ^{1}H NMR spectrum (DMSO-d_6, 400 MHz) of **4a**.

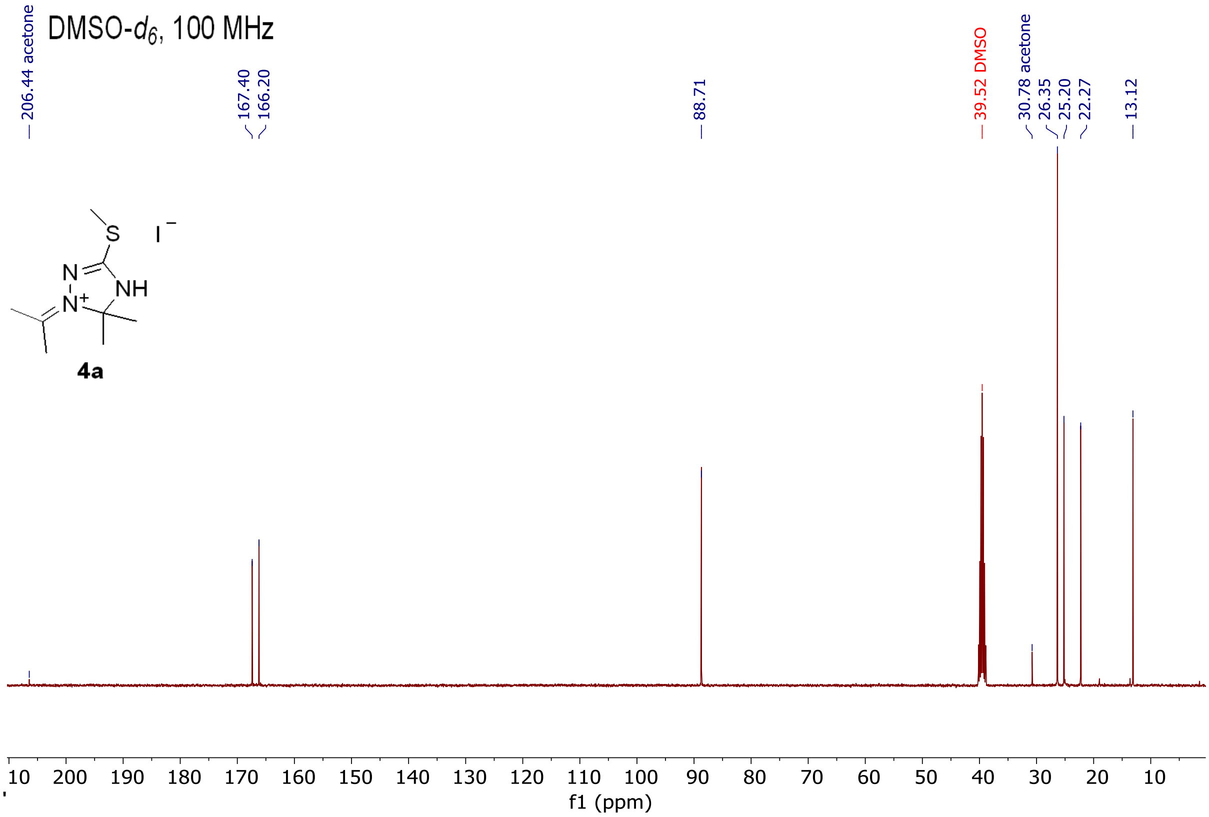

Figure S63. ^{13}C{^{1}H} NMR spectrum (DMSO-d_6, 100 MHz) of **4a**.

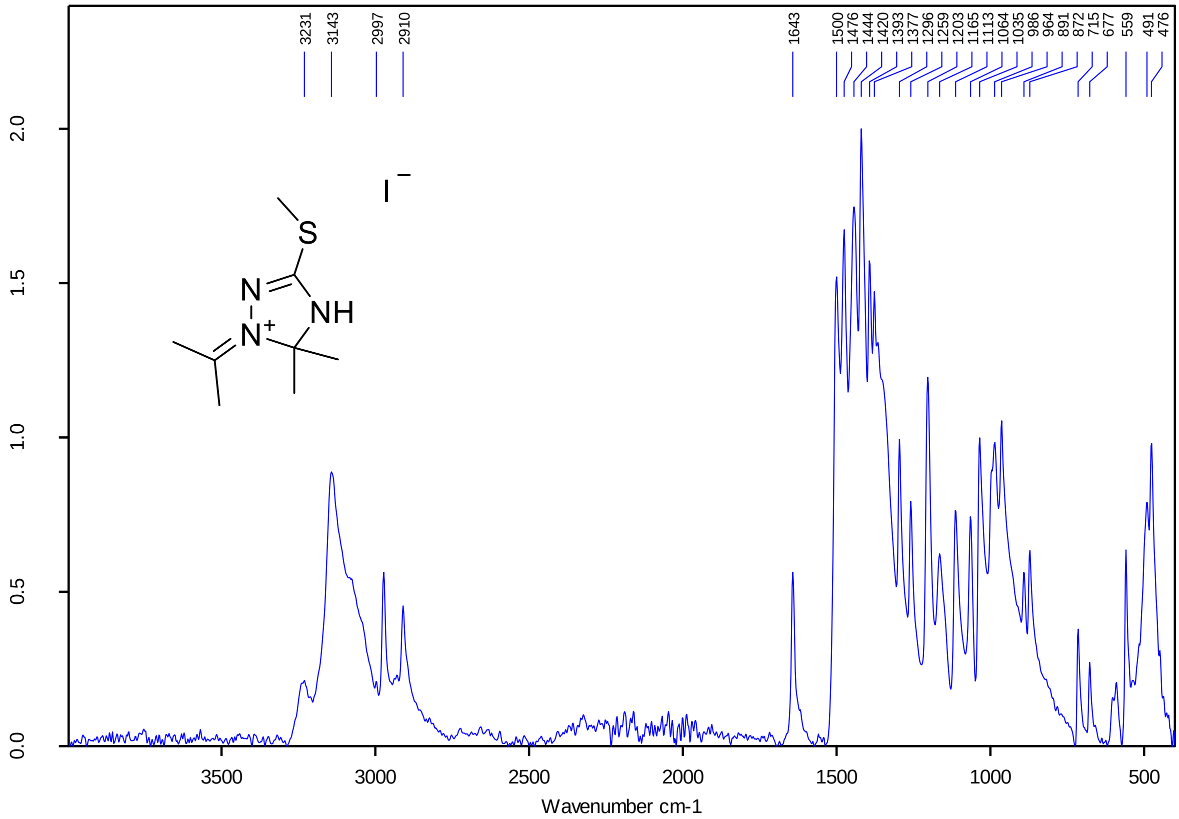

Figure S64. IR- absorption spectrum (neat) of **4a**.

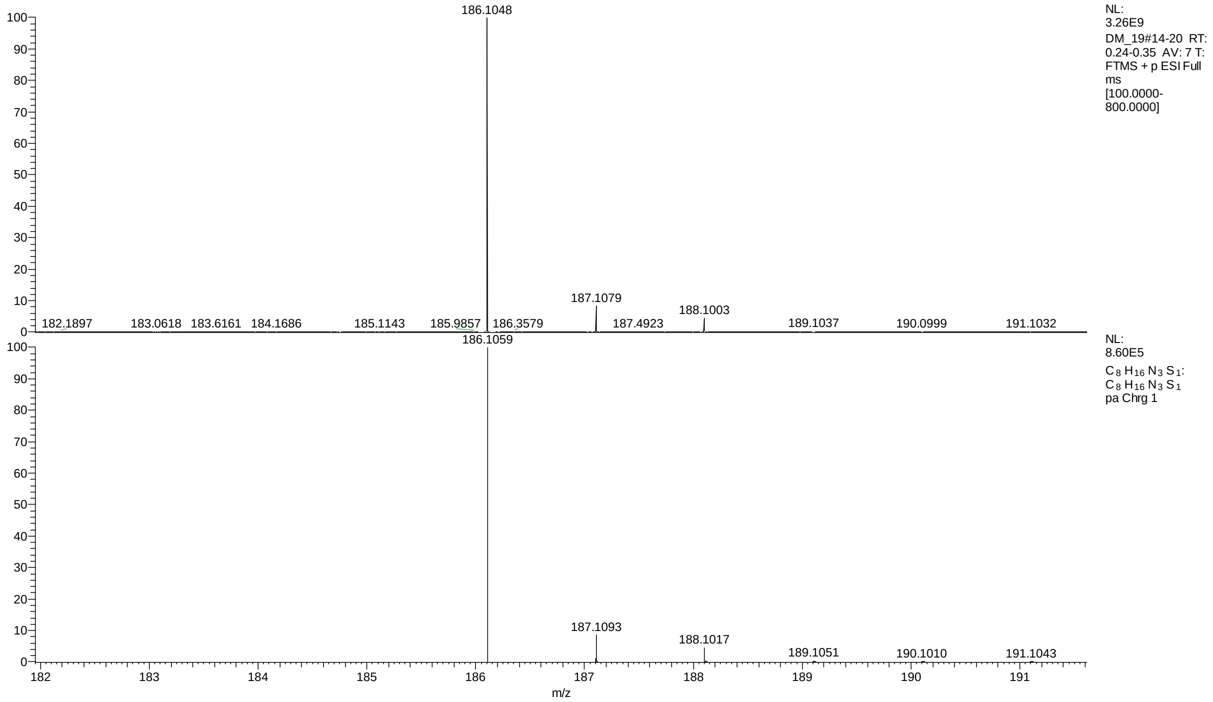

Figure S65. HR-ESI-MS (*m/z*) of **4a**. Top: measured peaks for $[C_8H_{16}N_3S_1]^+$; Bottom: calculated peaks for $[C_8H_{16}N_3S_1]^+$

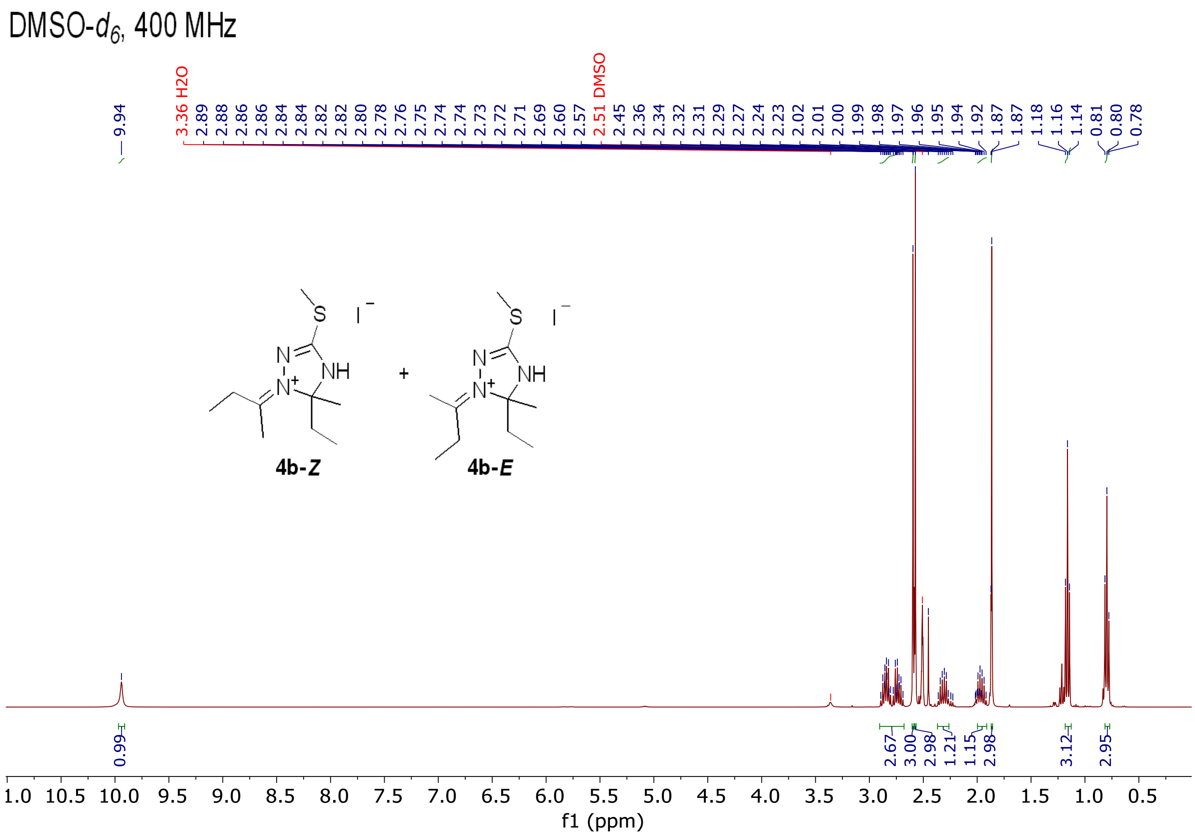

Figure S66. ^{1}H NMR spectrum (DMSO-d_6, 400 MHz) of **4b**– mixture of *Z* – and *E* isomers.

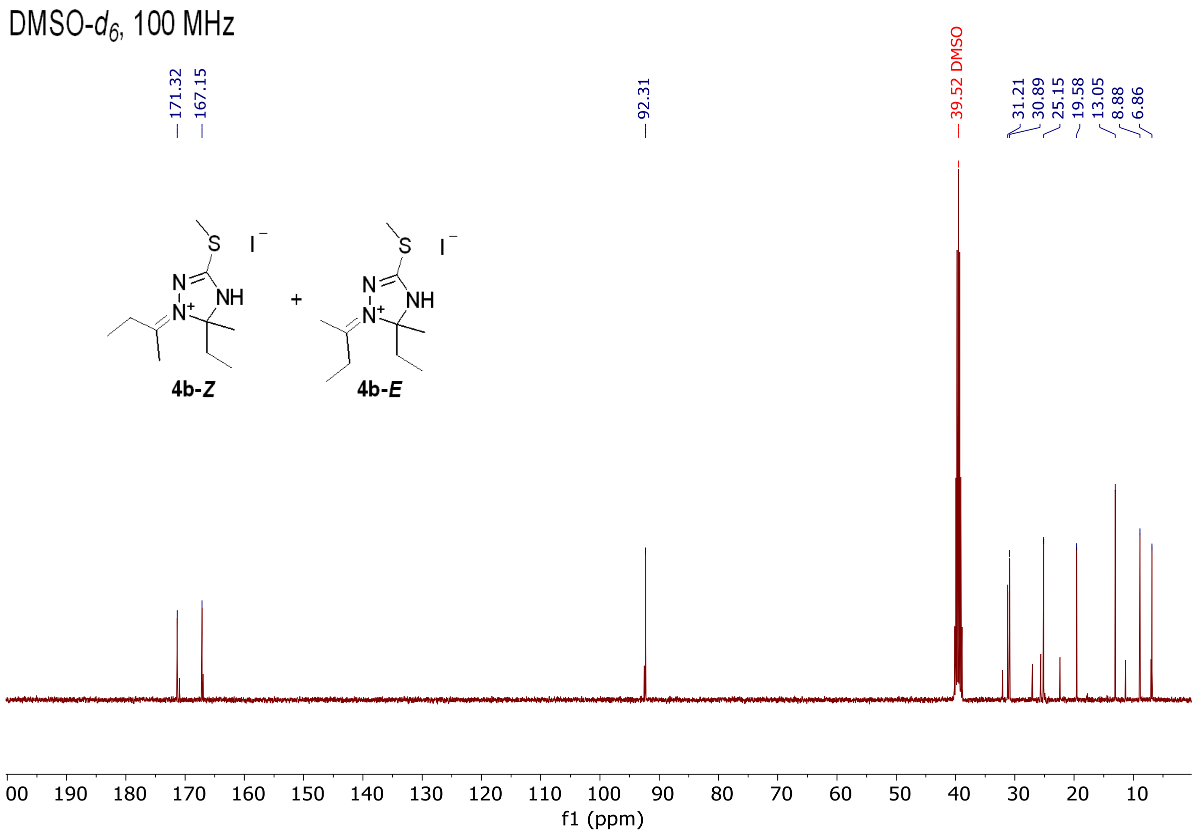

Figure S67. $^{13}C\{^{1}H\}$ NMR spectrum (DMSO-d_6, 100 MHz) of **4b** – mixture of *Z* – and *E* isomers.

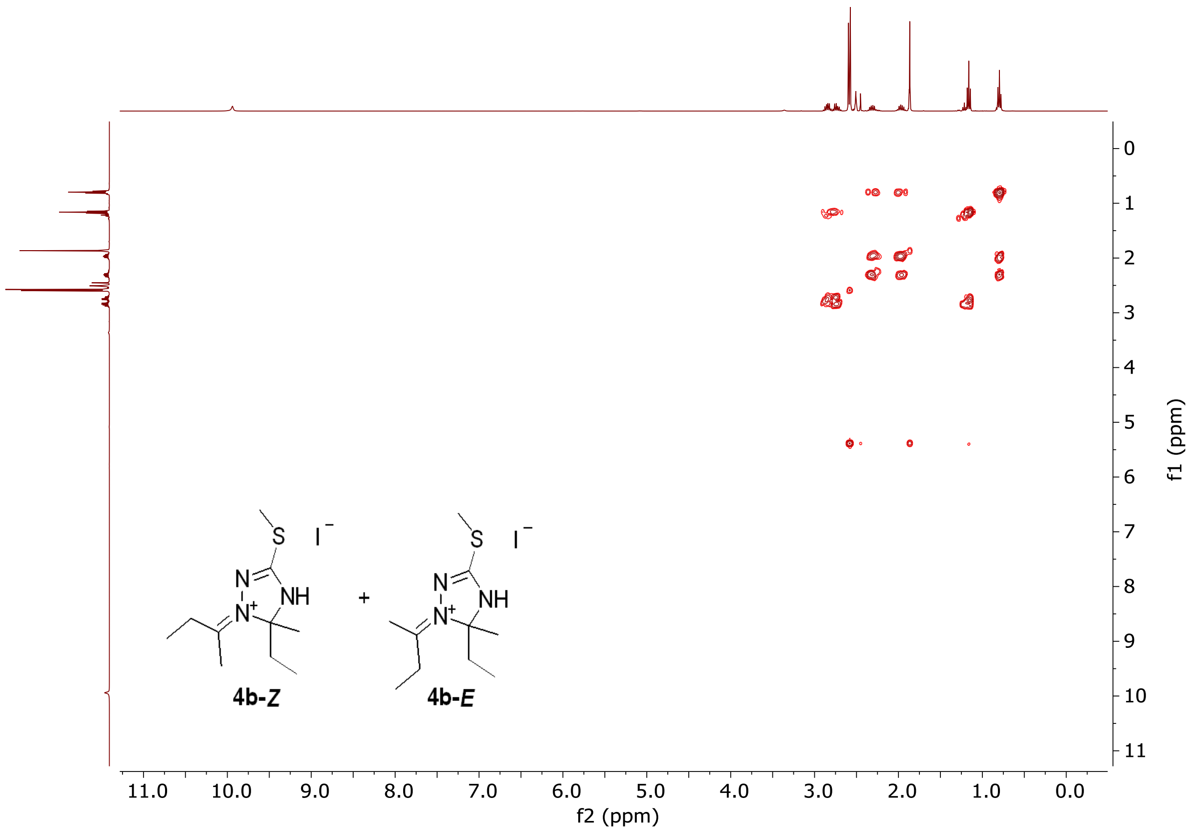

Figure S68. COSY spectrum (DMSO-d_6) of **4b** – mixture of *Z* – and *E* isomers.

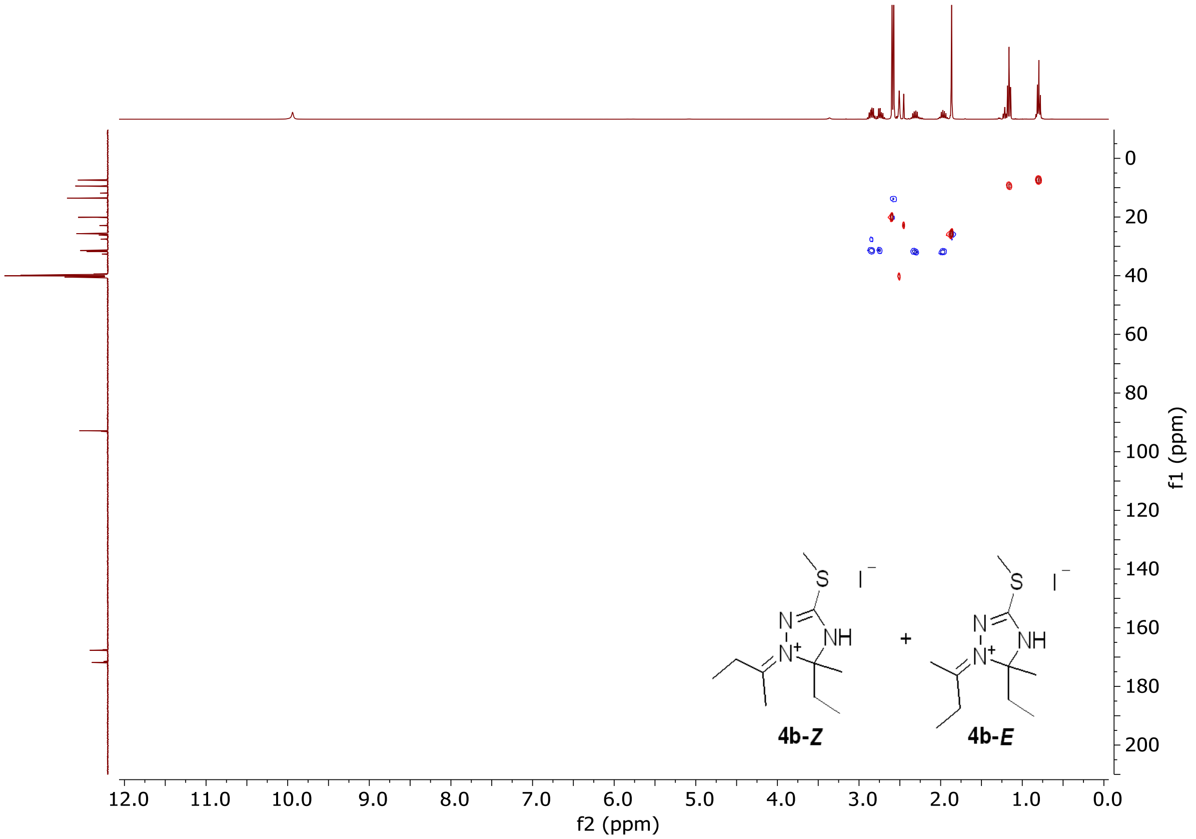

Figure S69. HSQC spectrum (DMSO-d_6) of **4b** – mixture of *Z* – and *E* isomers.

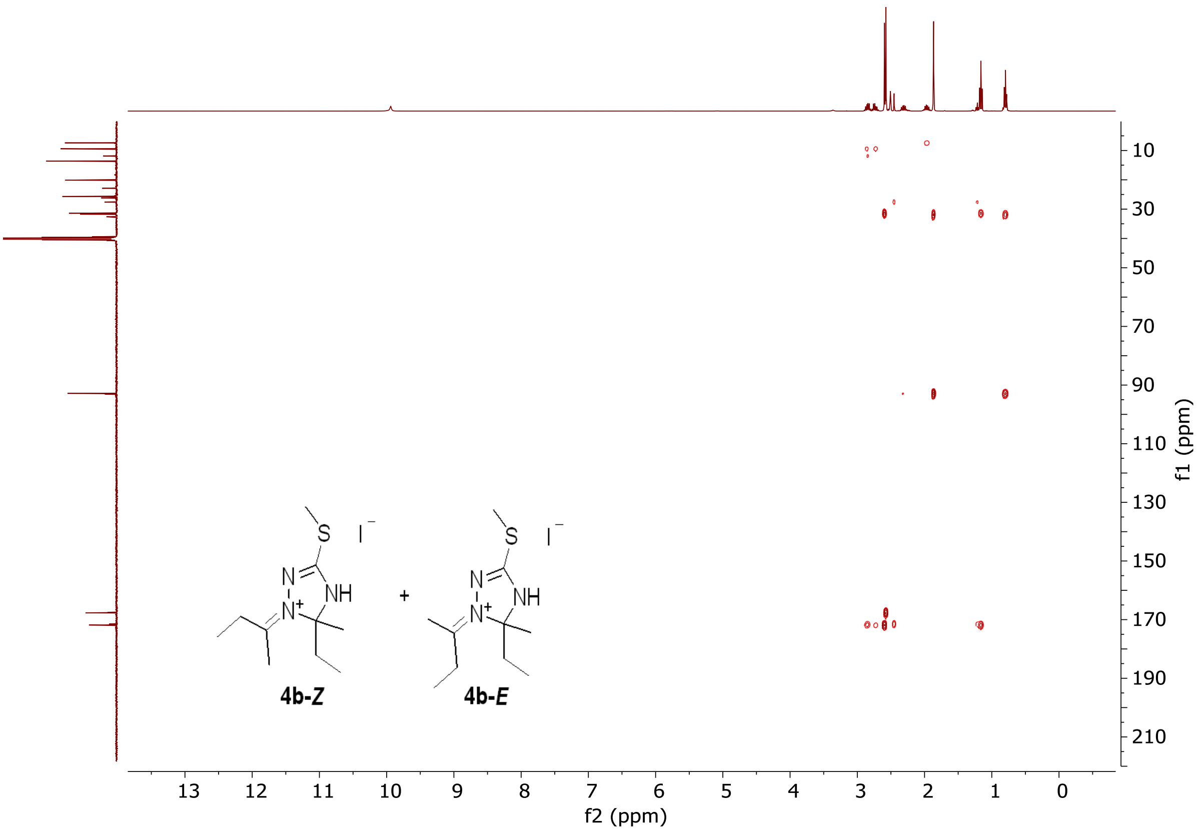

Figure S70. HMBC spectrum (DMSO-d_6) of **4b** – mixture of *Z* – and *E* isomers.

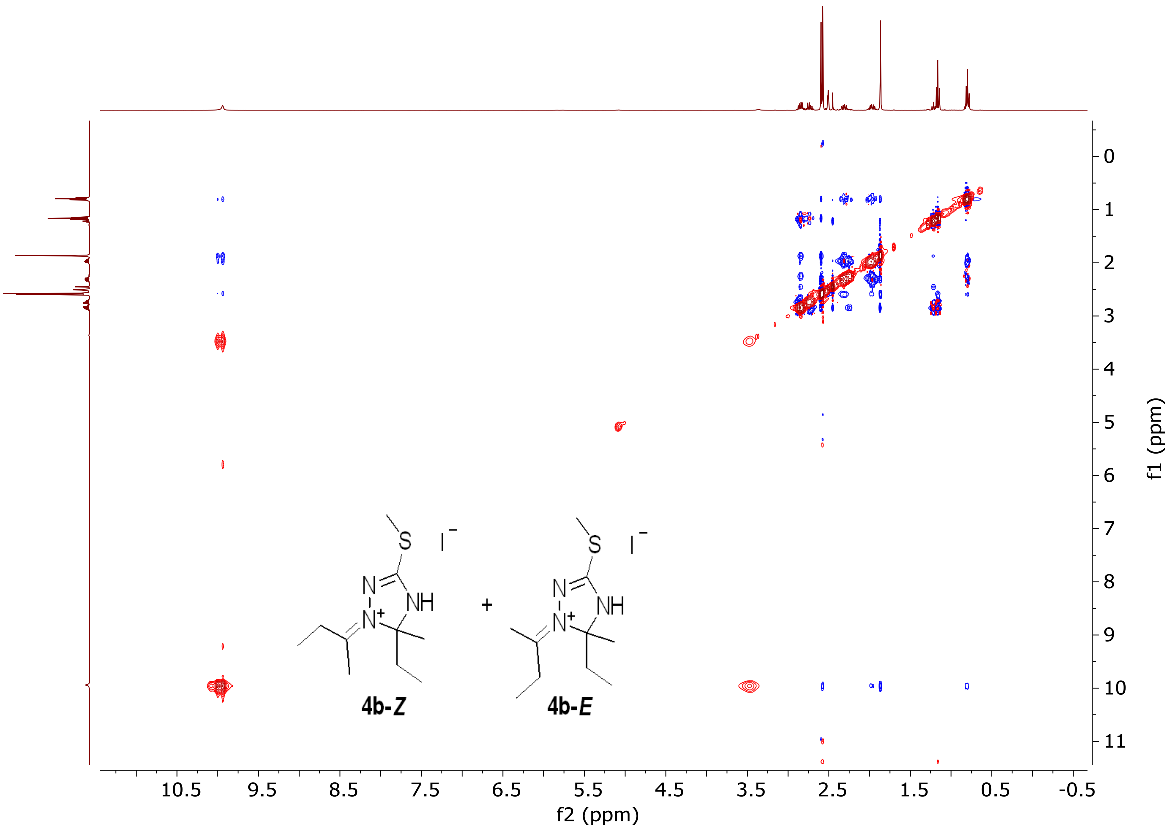

Figure S71. NOESY spectrum (DMSO-d_6) of **4b** – mixture of *Z* – and *E* isomers.

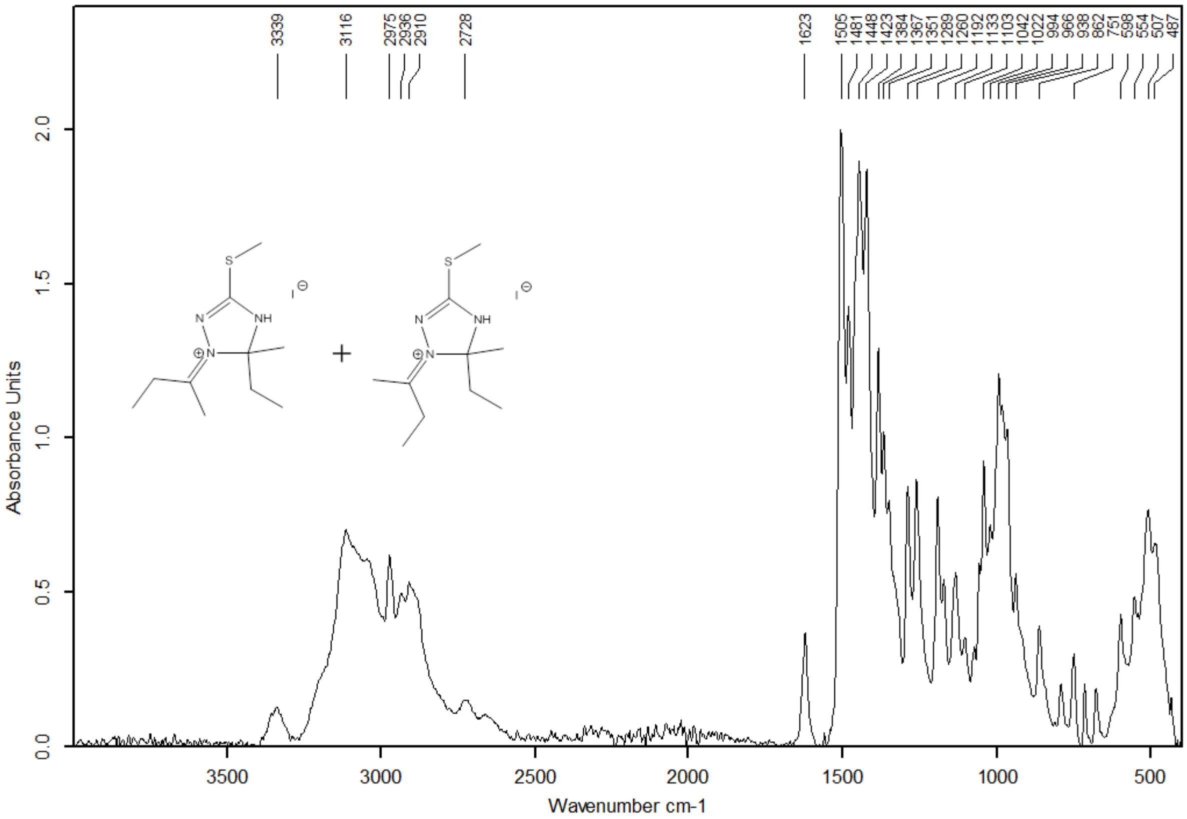

Figure S72. IR- absorption spectrum (neat) of **4b** – mixture of *Z* – and *E* isomers.

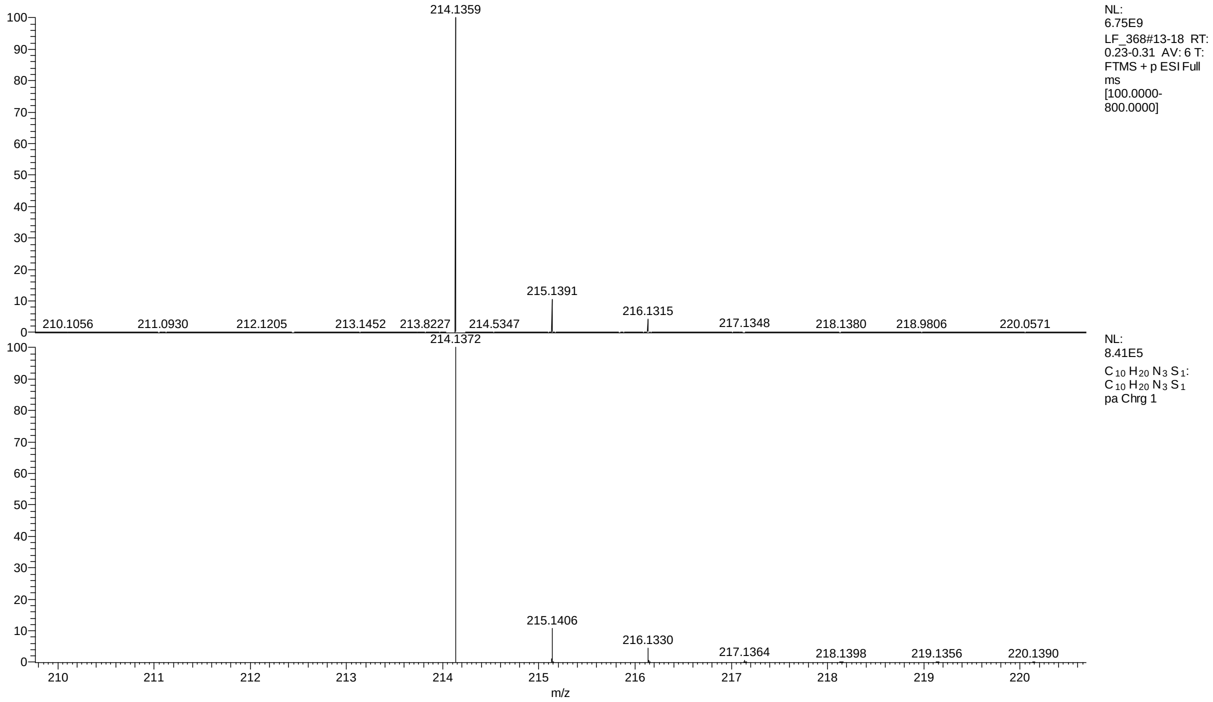

Figure S73. HR-ESI-MS (*m/z*) of **4b** – mixture of *Z* – and *E* isomers. Top: measured peaks for $[C_{10}H_{20}N_3S_1]^+$; Bottom: calculated peaks for $[C_{10}H_{20}N_3S_1]^+$

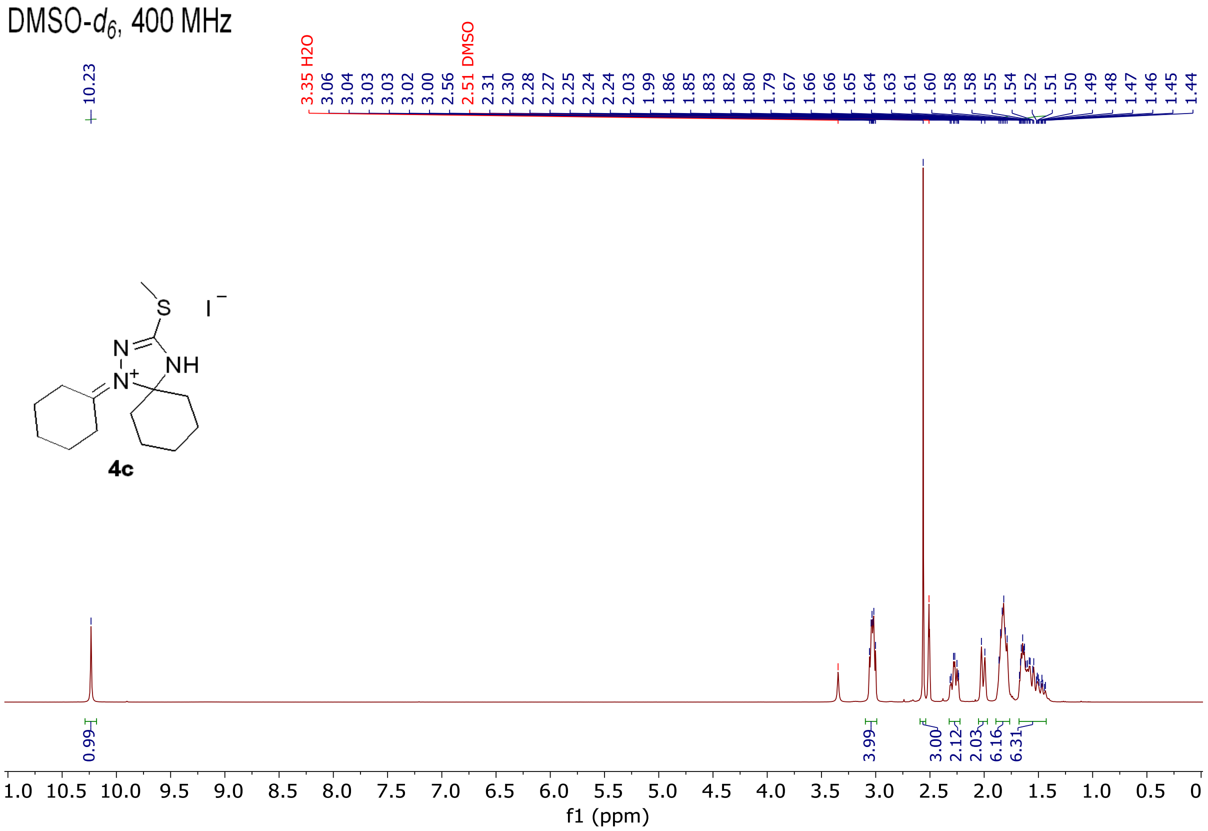

Figure S74. ^{1}H NMR spectrum (DMSO-d_6, 400 MHz) of **4c**

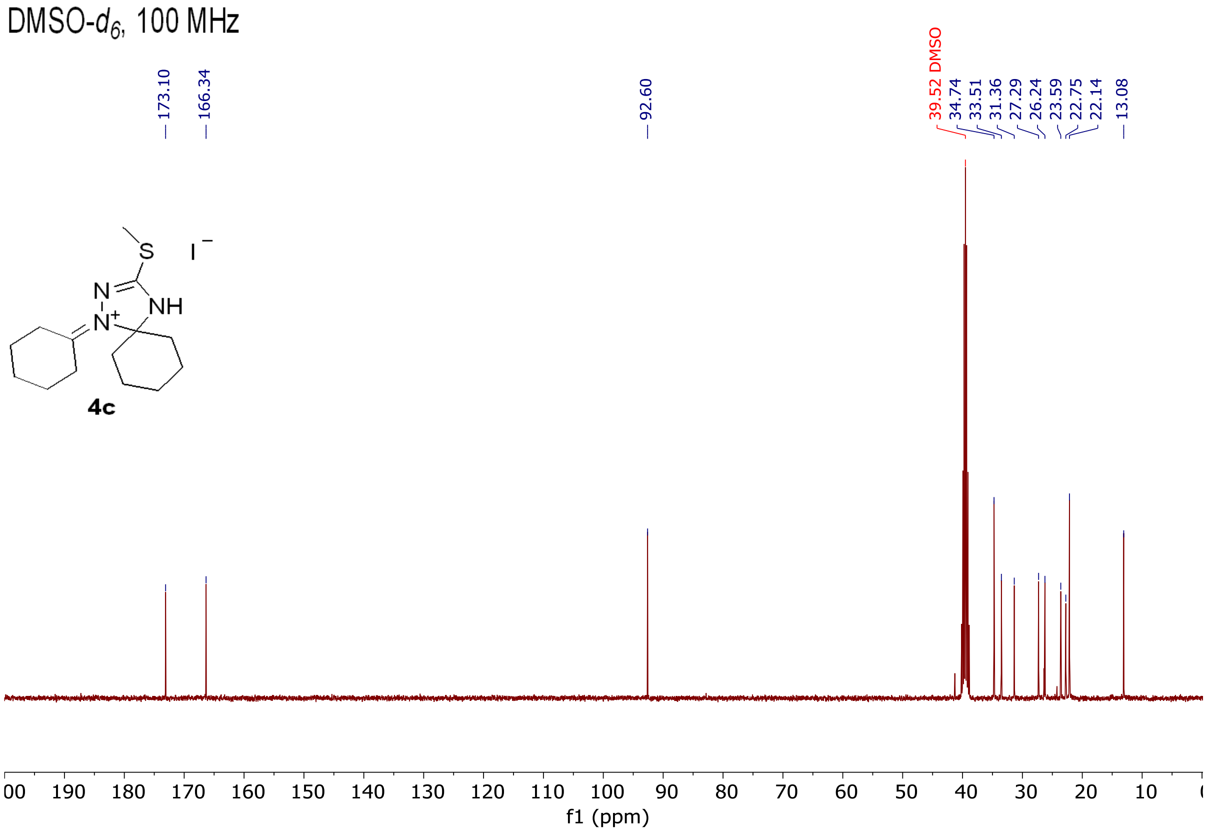

Figure S75. ^{13}C{^{1}H} NMR spectrum (DMSO-d_6, 100 MHz) **4c**

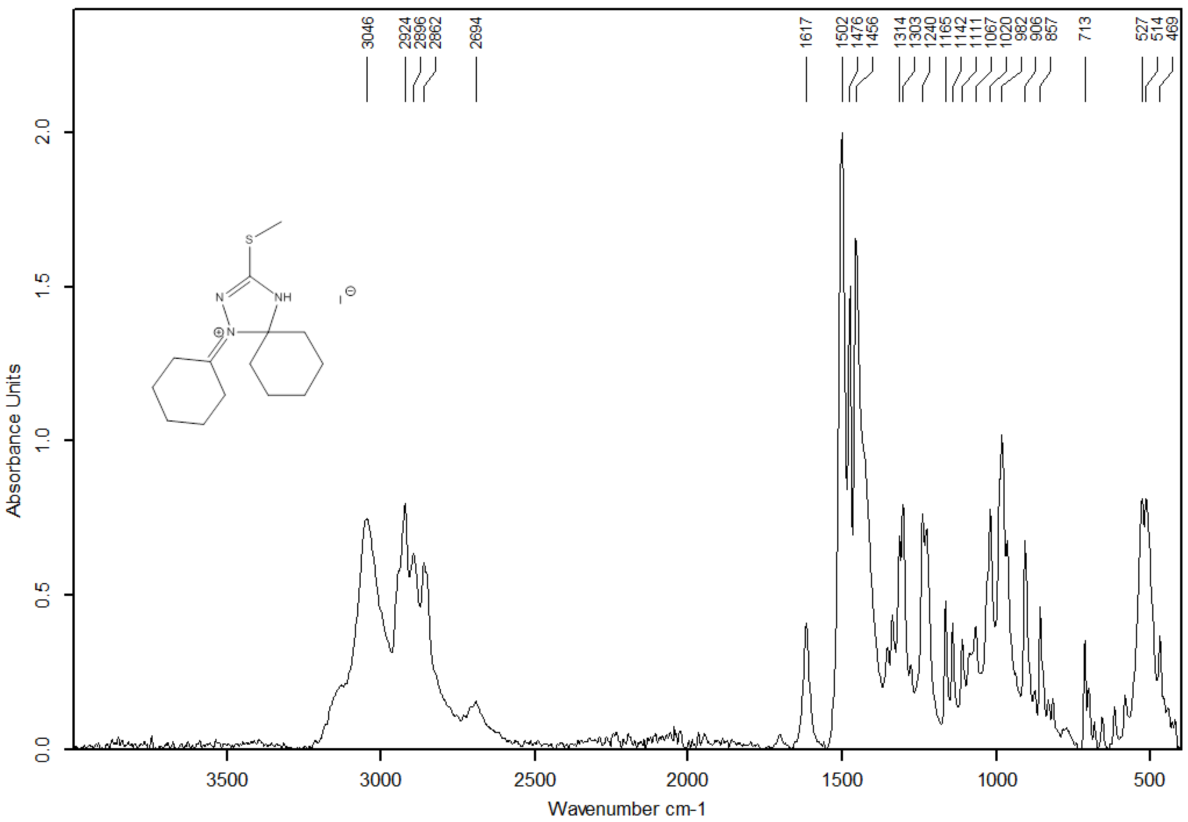

Figure S76. IR- absorption spectrum (neat) of **4c**.

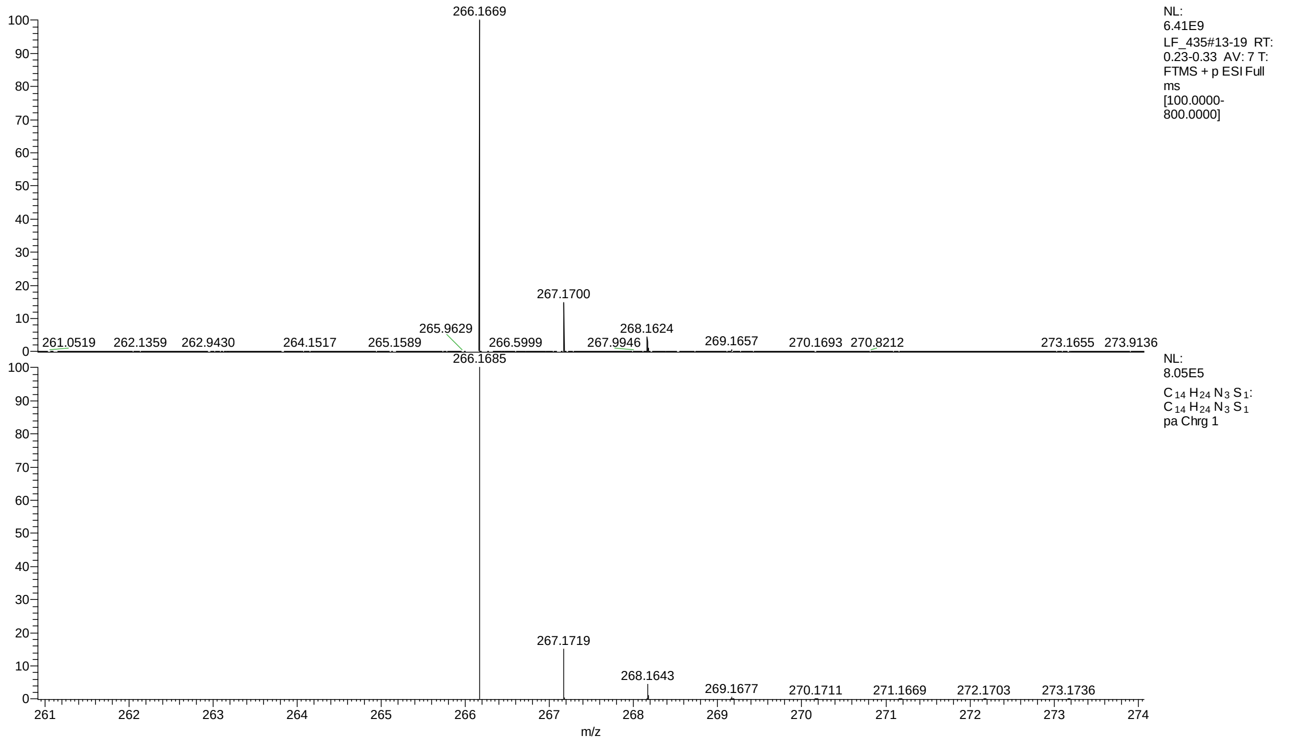

Figure S77. HR-ESI-MS (*m/z*) of **4c**. Top: measured peaks for $[C_{14}H_{24}N_3S_1]^+$; Bottom: calculated peaks for $[C_{14}H_{24}N_3S_1]^+$

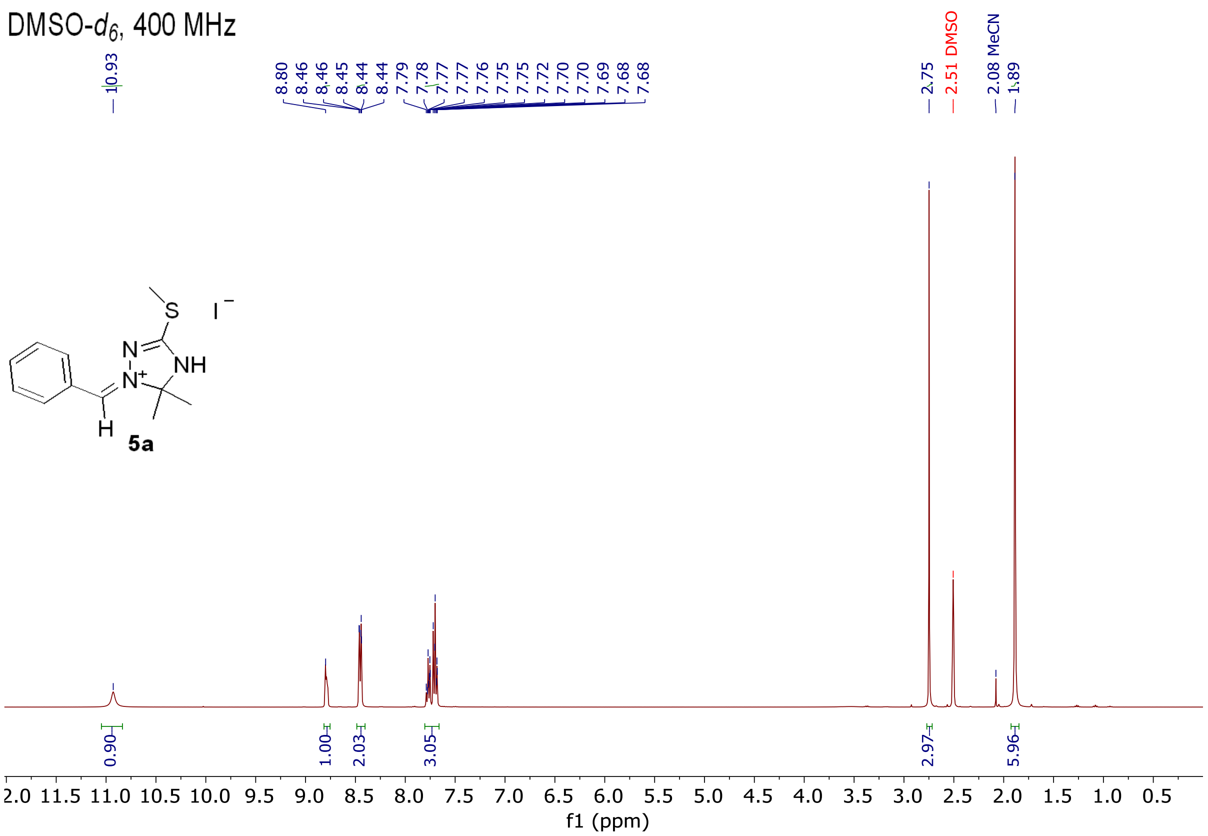

Figure S78. ^{1}H NMR spectrum (DMSO-d_6, 400 MHz) of **5a**.

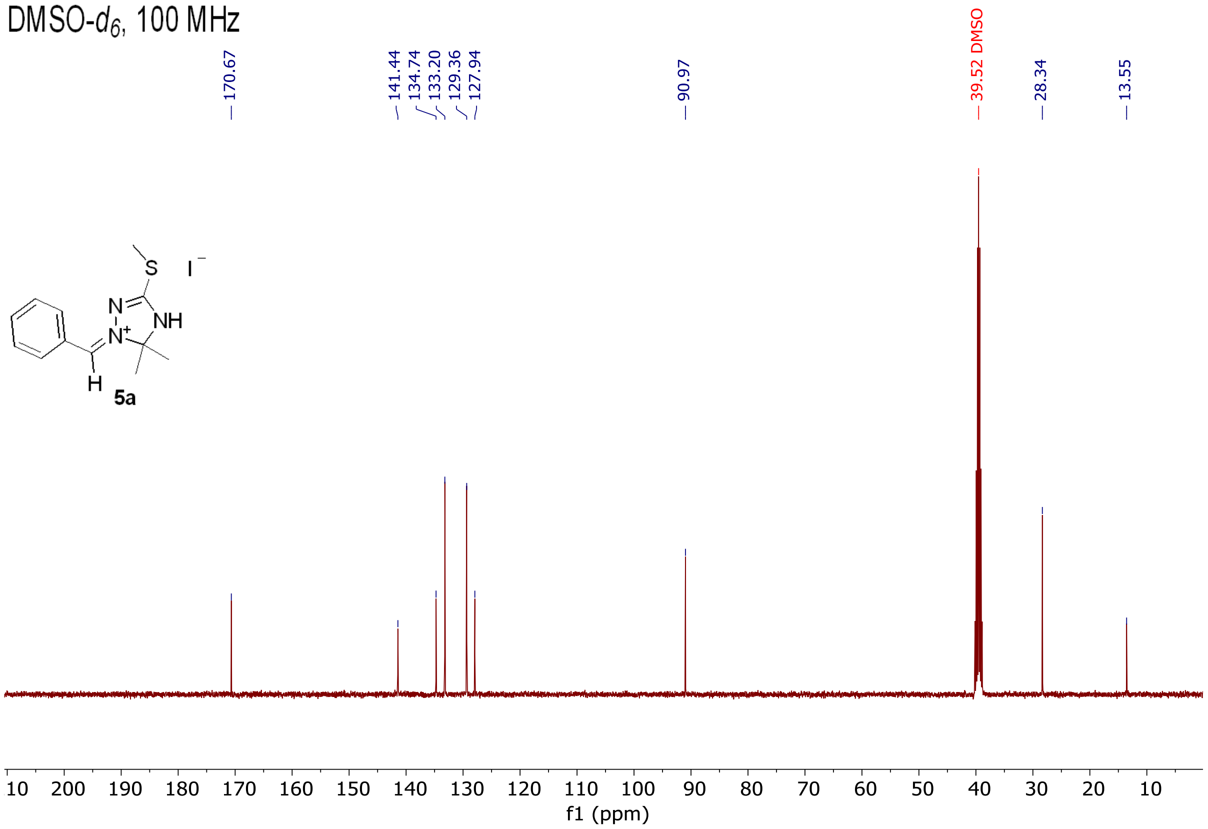

Figure S79. ^{13}C{^{1}H} NMR spectrum (DMSO-d_6, 100 MHz) **5a**.

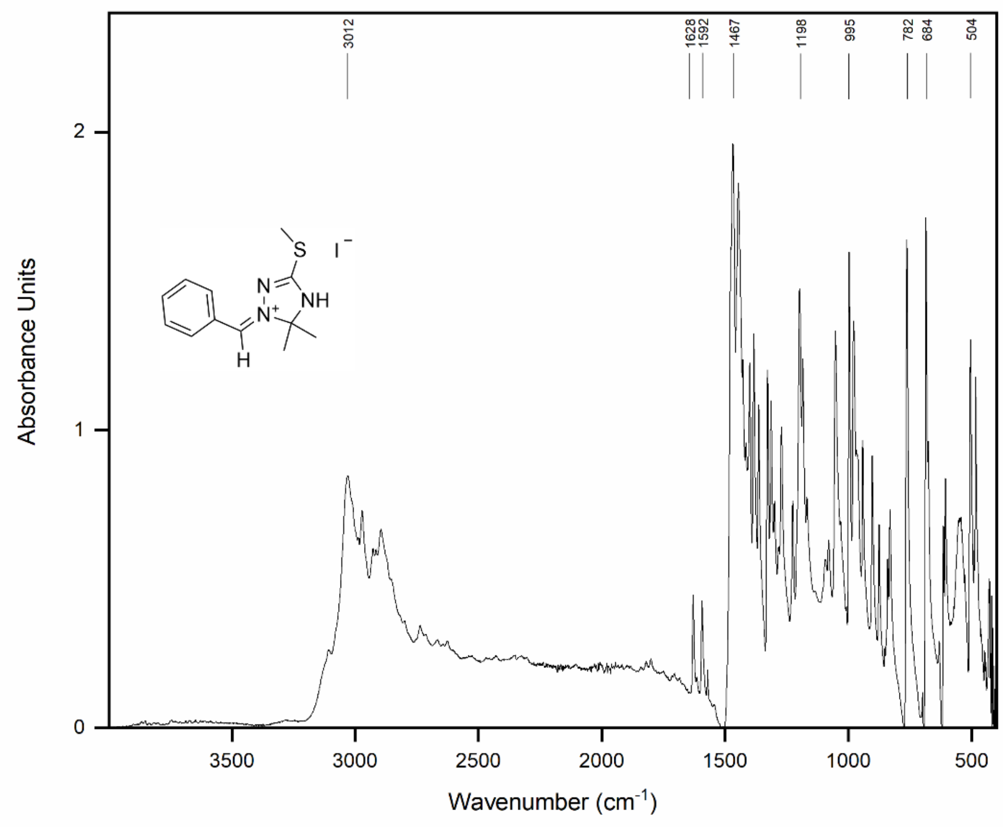

Figure S80. IR- absorption spectrum (neat) of **5a**.

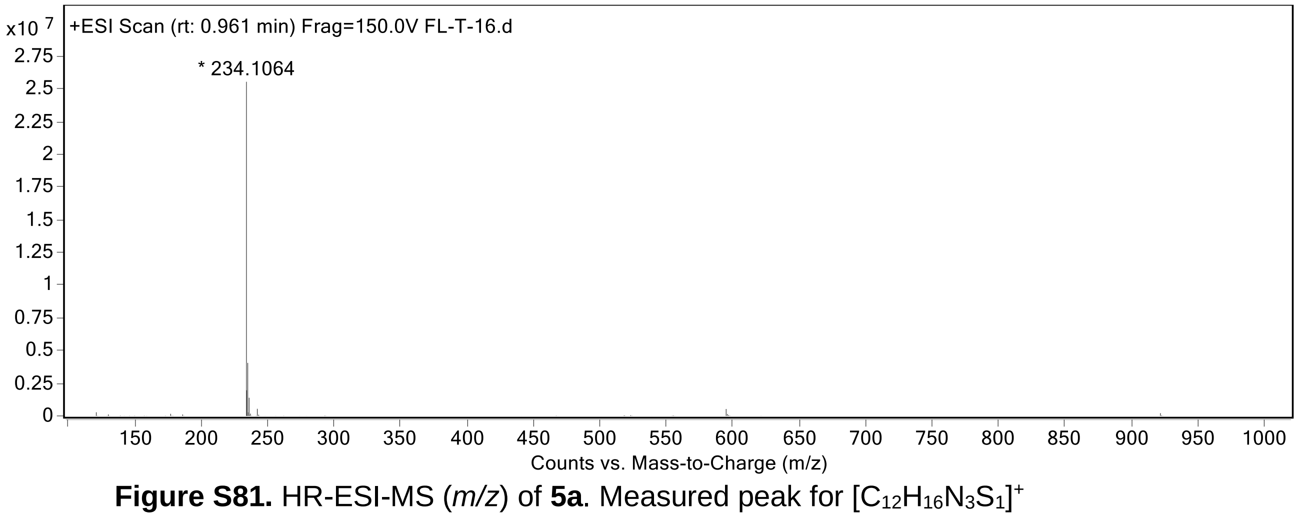

Figure S81. HR-ESI-MS (*m/z*) of **5a**. Measured peak for $[C_{12}H_{16}N_3S_1]^+$

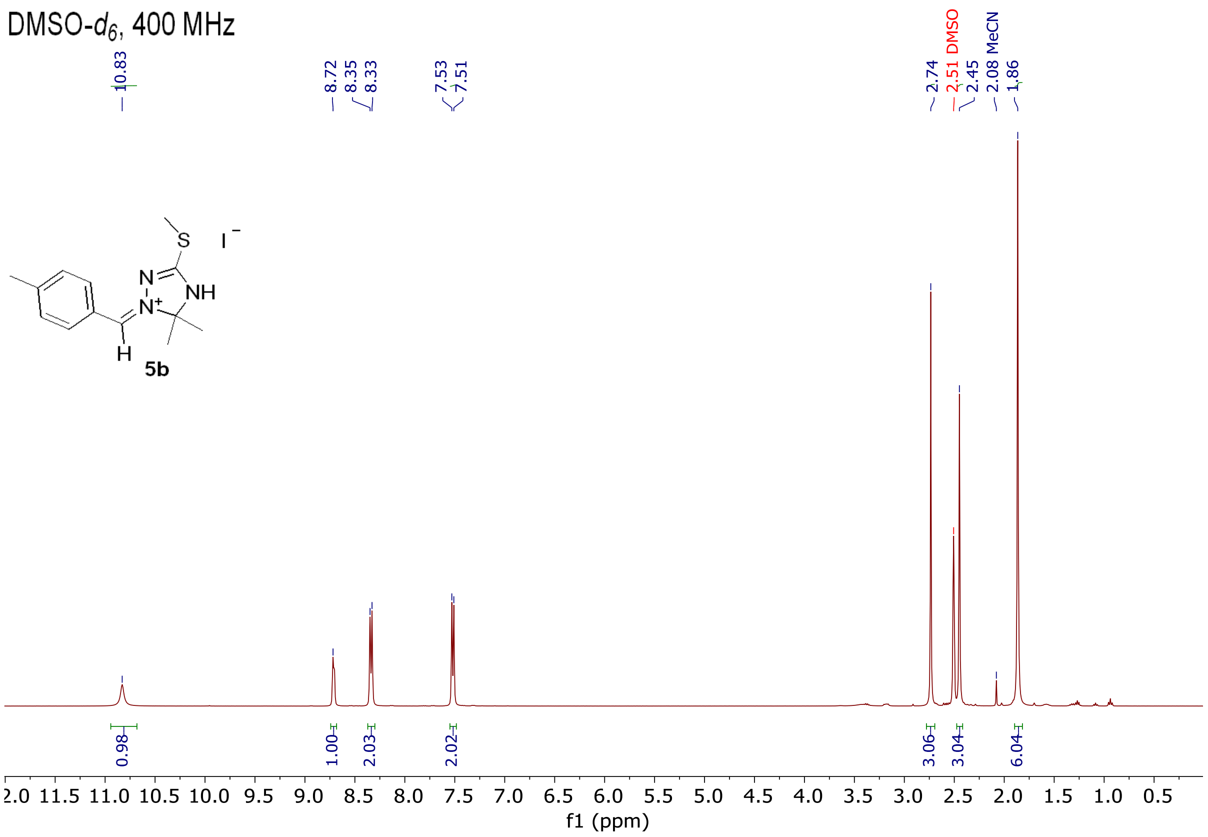

Figure S82. ^{1}H NMR spectrum (DMSO-d_6, 400 MHz) of **5b**.

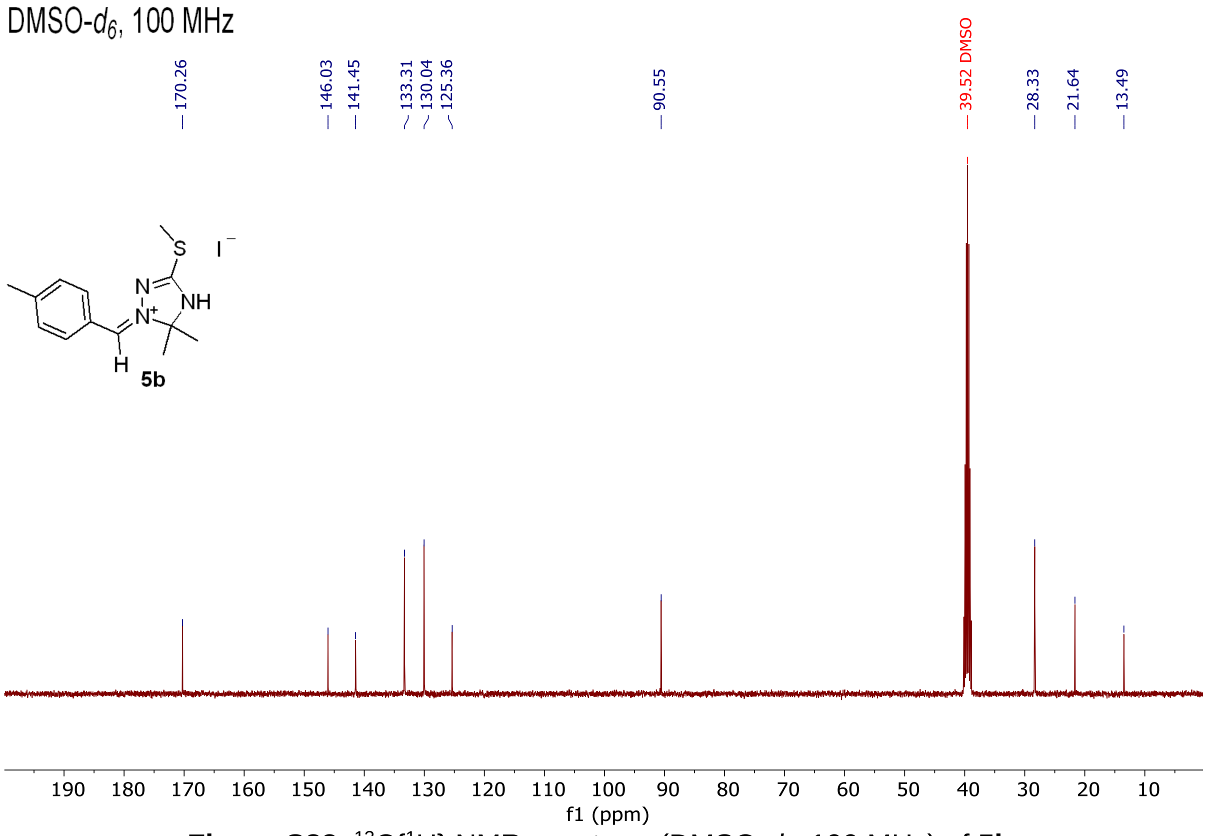

Figure S83. ^{13}C{^{1}H} NMR spectrum (DMSO-d_6, 100 MHz) of **5b**.

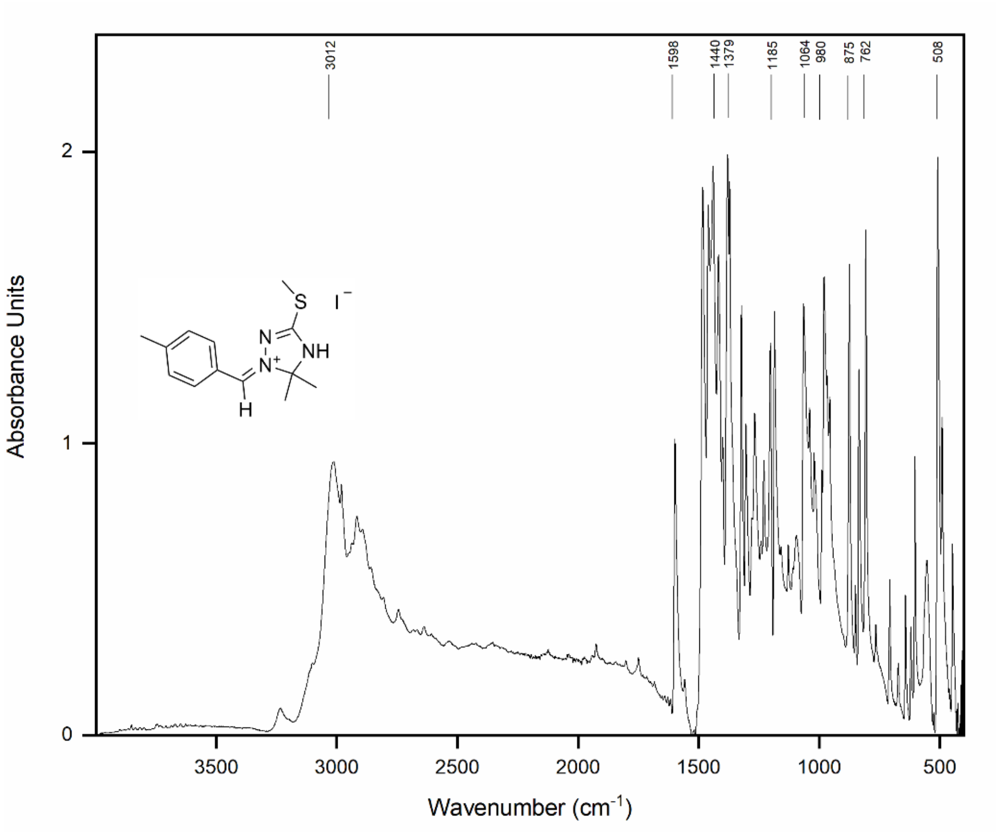

Figure S84. IR- absorption spectrum (neat) of **5b**.

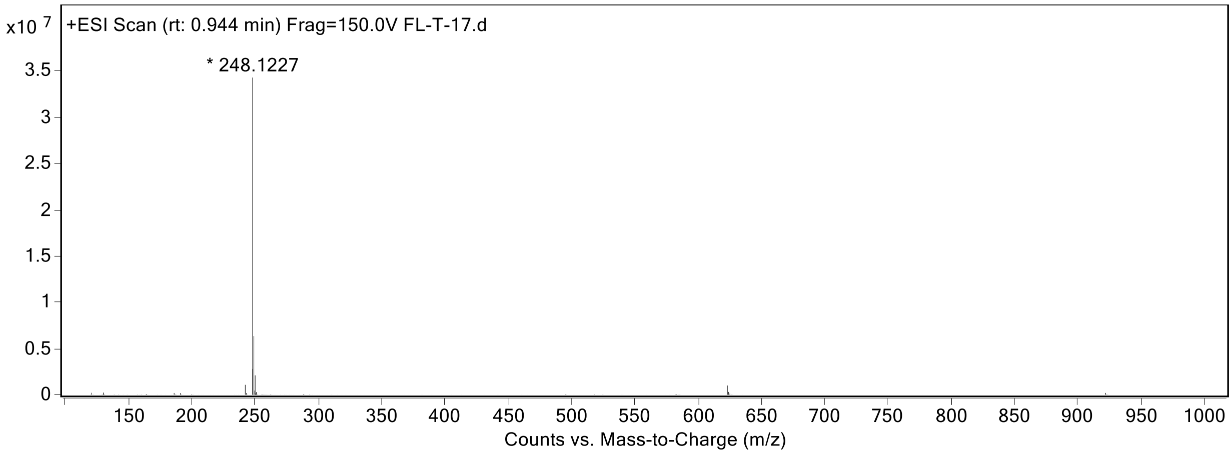

Figure S85. HR-ESI-MS (*m/z*) **5b**. Measured peak for $[C_{13}H_{18}N_3S_1]^+$

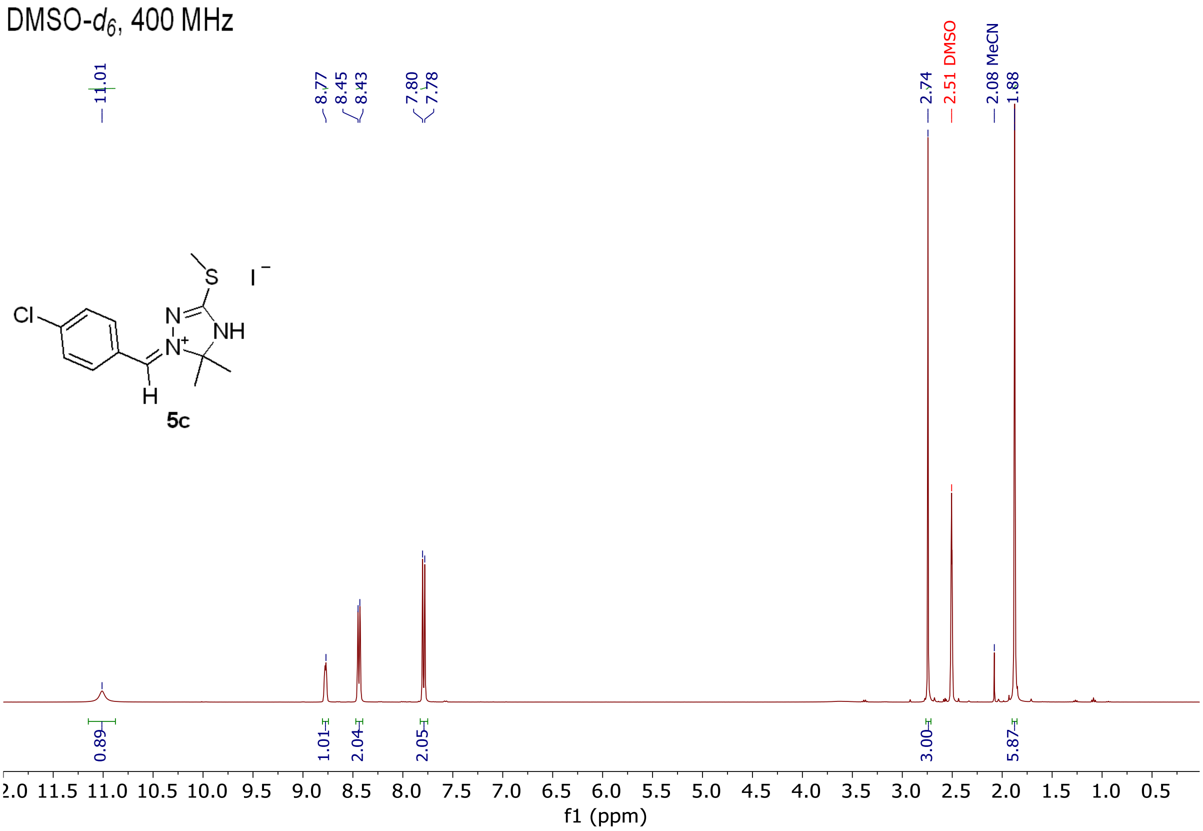

Figure S86. ¹H NMR spectrum (DMSO-d_6, 400 MHz) of **5c**.

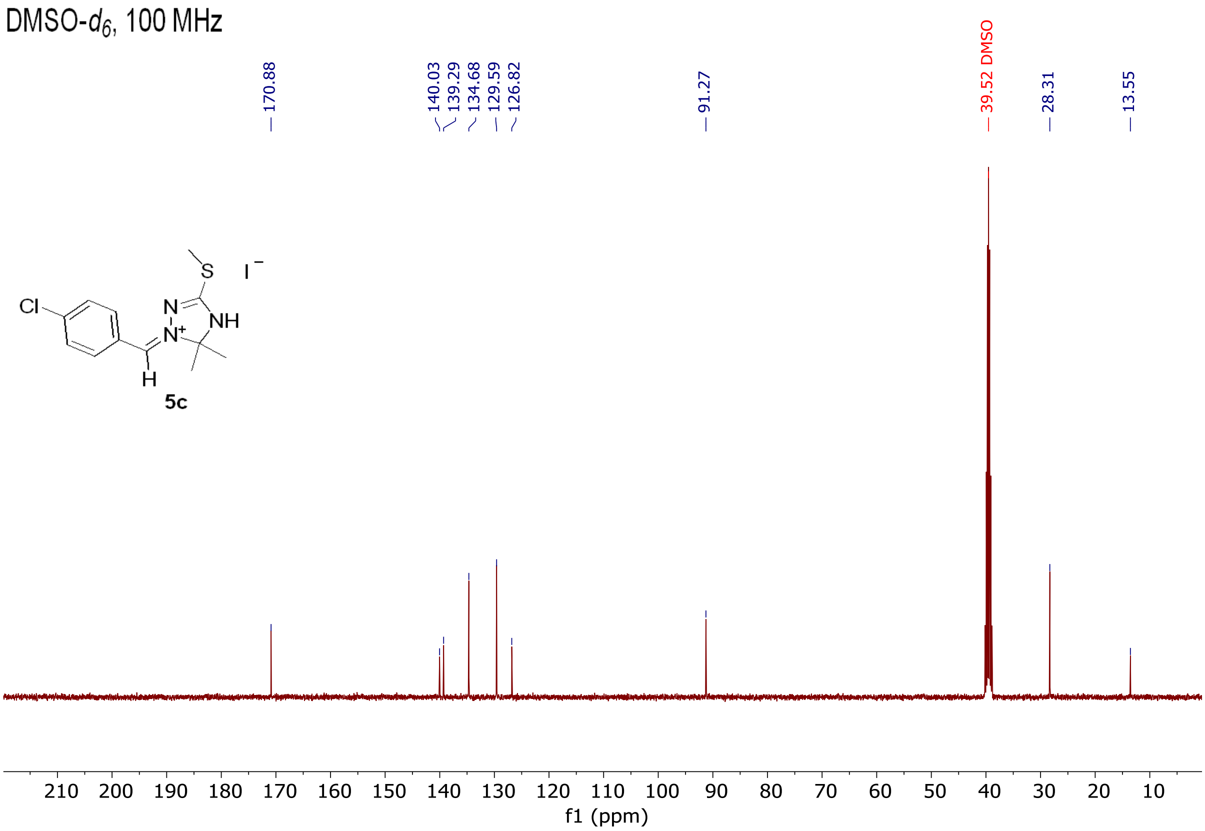

Figure S87. ^{13}C{^{1}H} NMR spectrum (DMSO-d_6, 100 MHz) of **5c**.

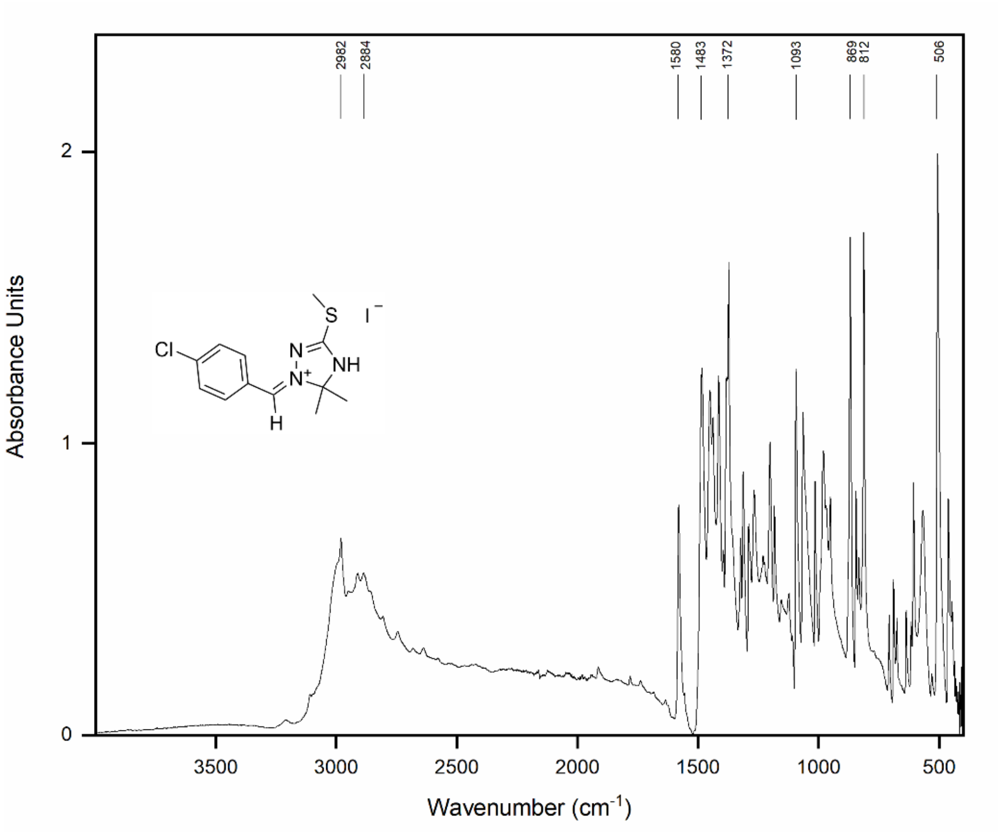

Figure S88. IR- absorption spectrum (neat) of **5c**.

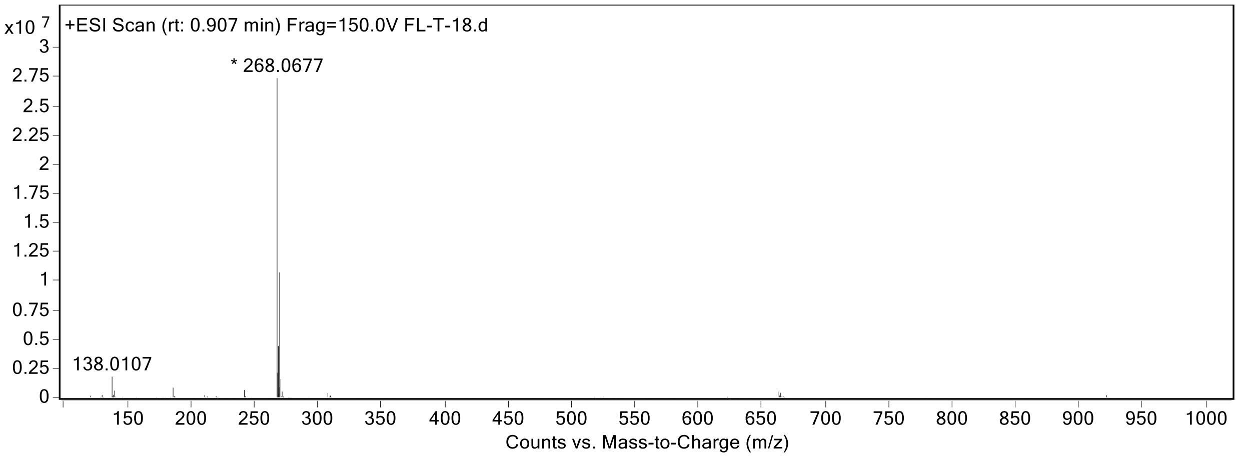

Figure S89. HR-ESI-MS (*m/z*) of **5c**. Measured peak for $[C_{12}H_{15}N_3S_1Cl_1]^+$

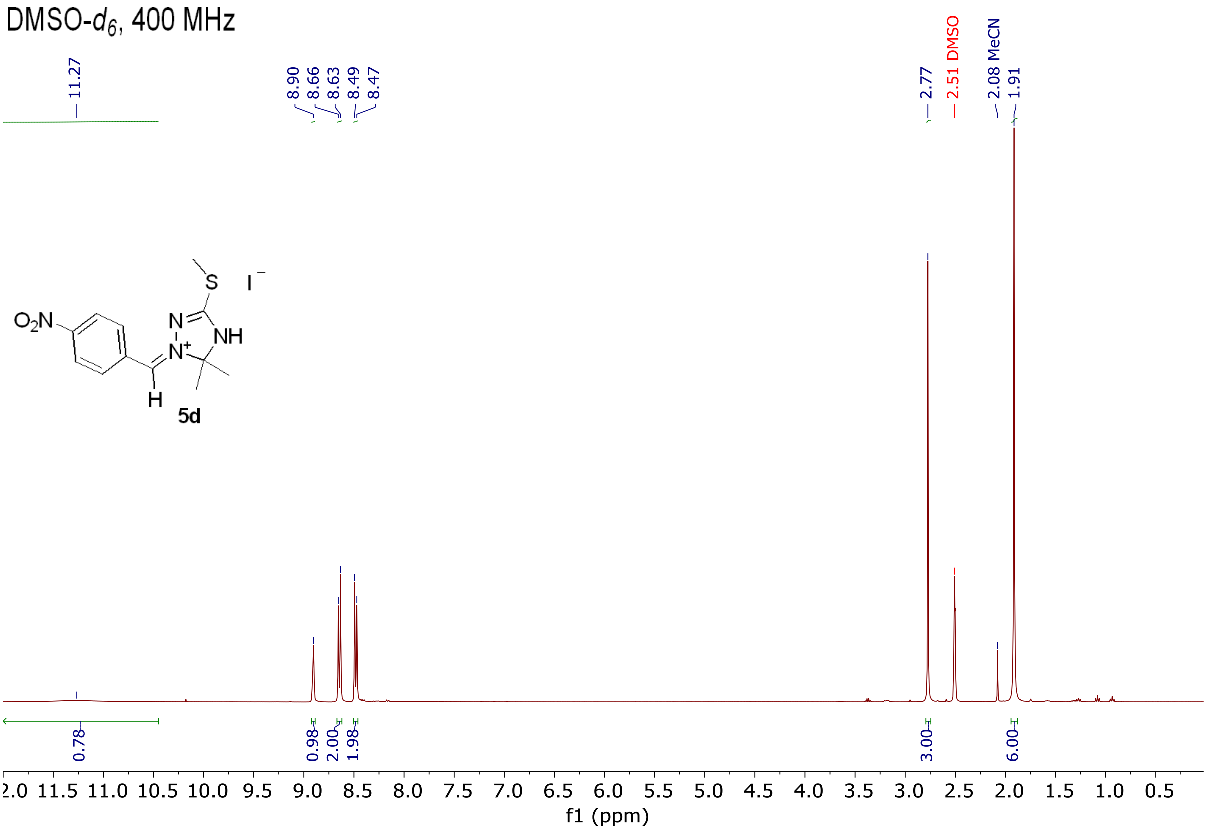

Figure S90. ^{1}H NMR spectrum (DMSO-d_6, 400 MHz) of **5d**.

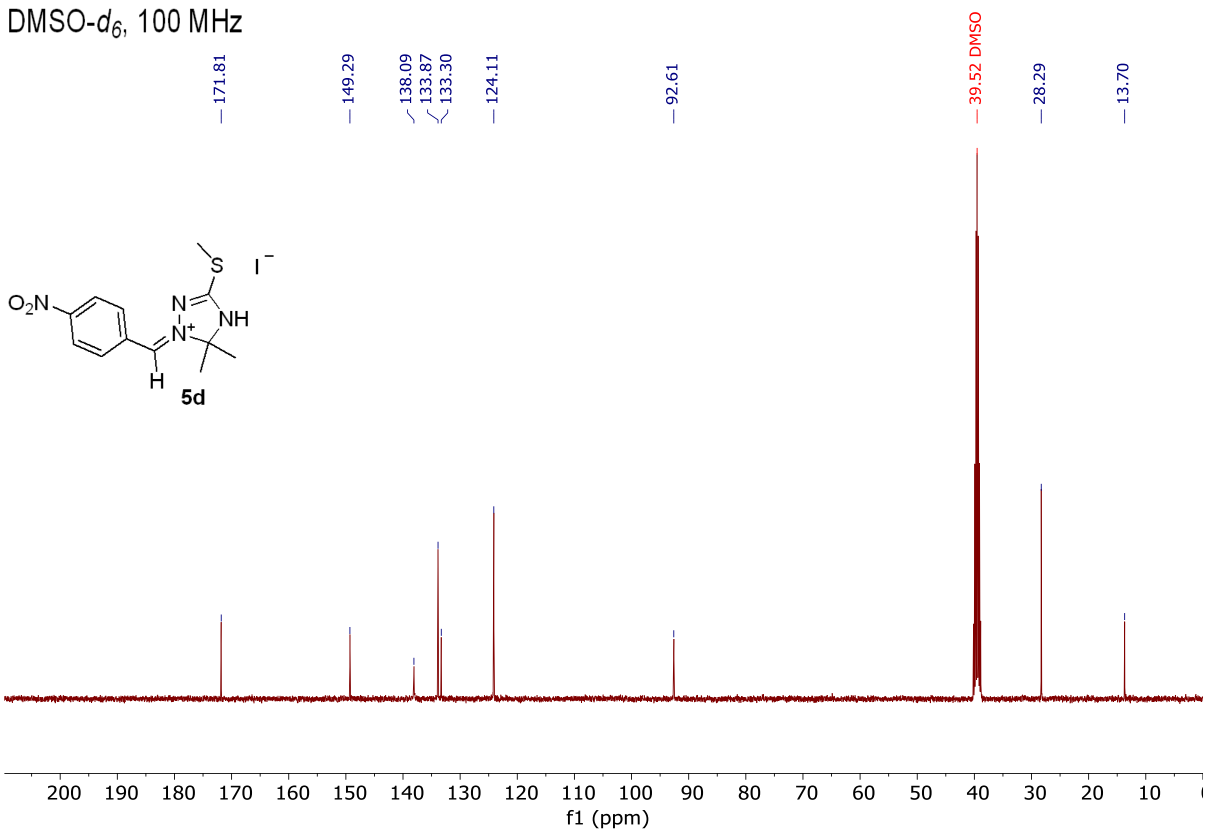

Figure S91. $^{13}C\{^1H\}$ NMR spectrum (DMSO-d_6, 100 MHz) of **5d**.

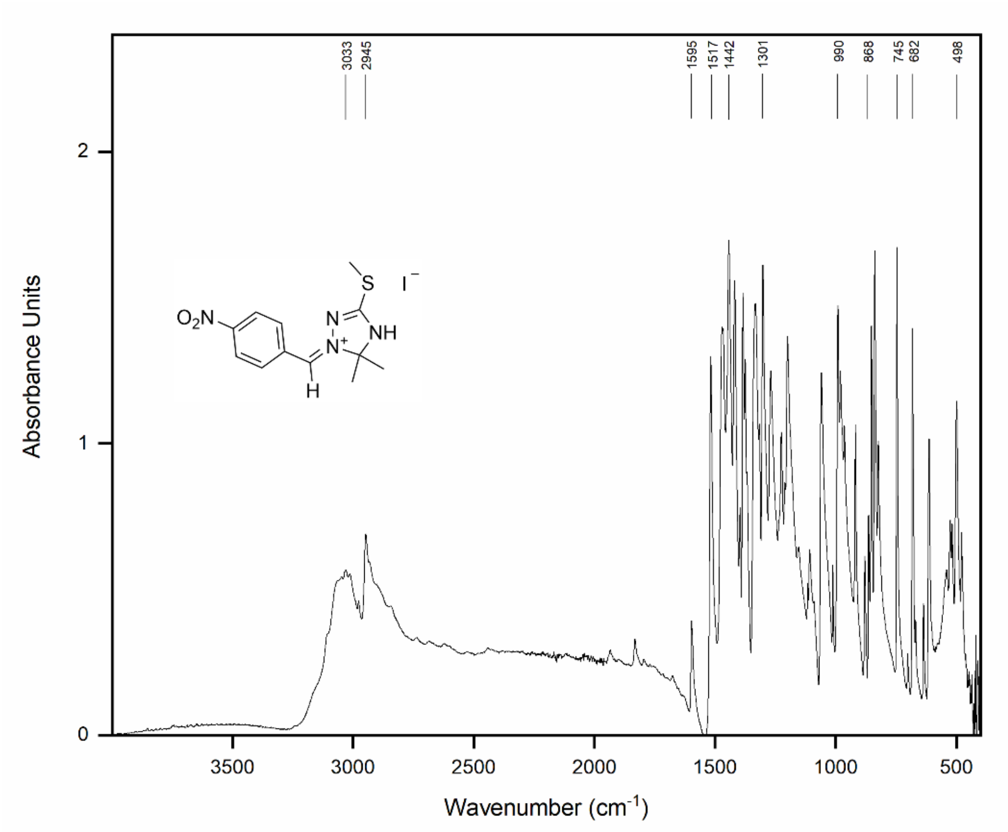

Figure S92. IR- absorption spectrum (neat) of **5d**.

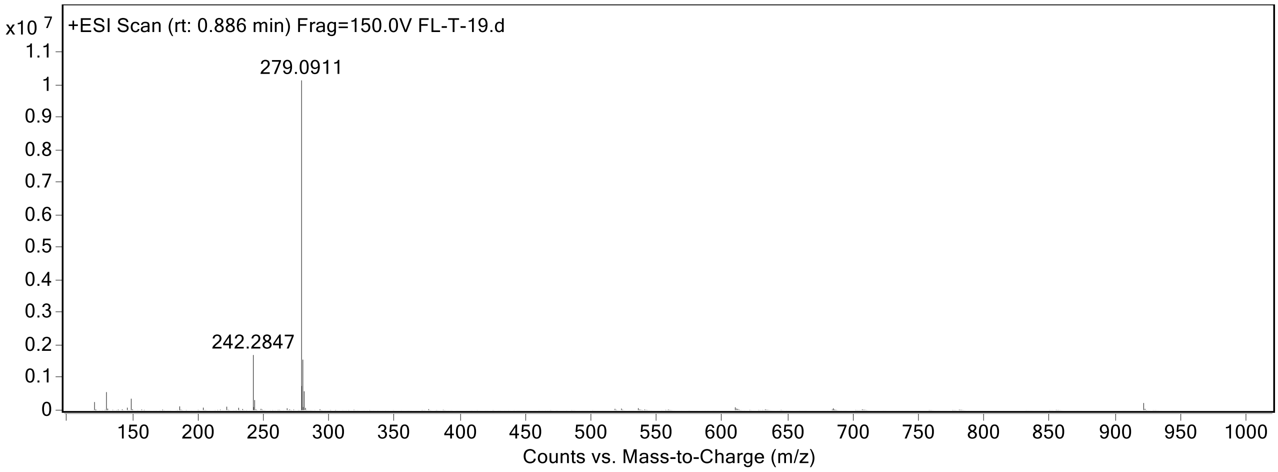

Figure S93. HR-ESI-MS (*m/z*) of **5d**. Measured peak for $[C_{12}H_{15}N_4S_1O_2]^+$

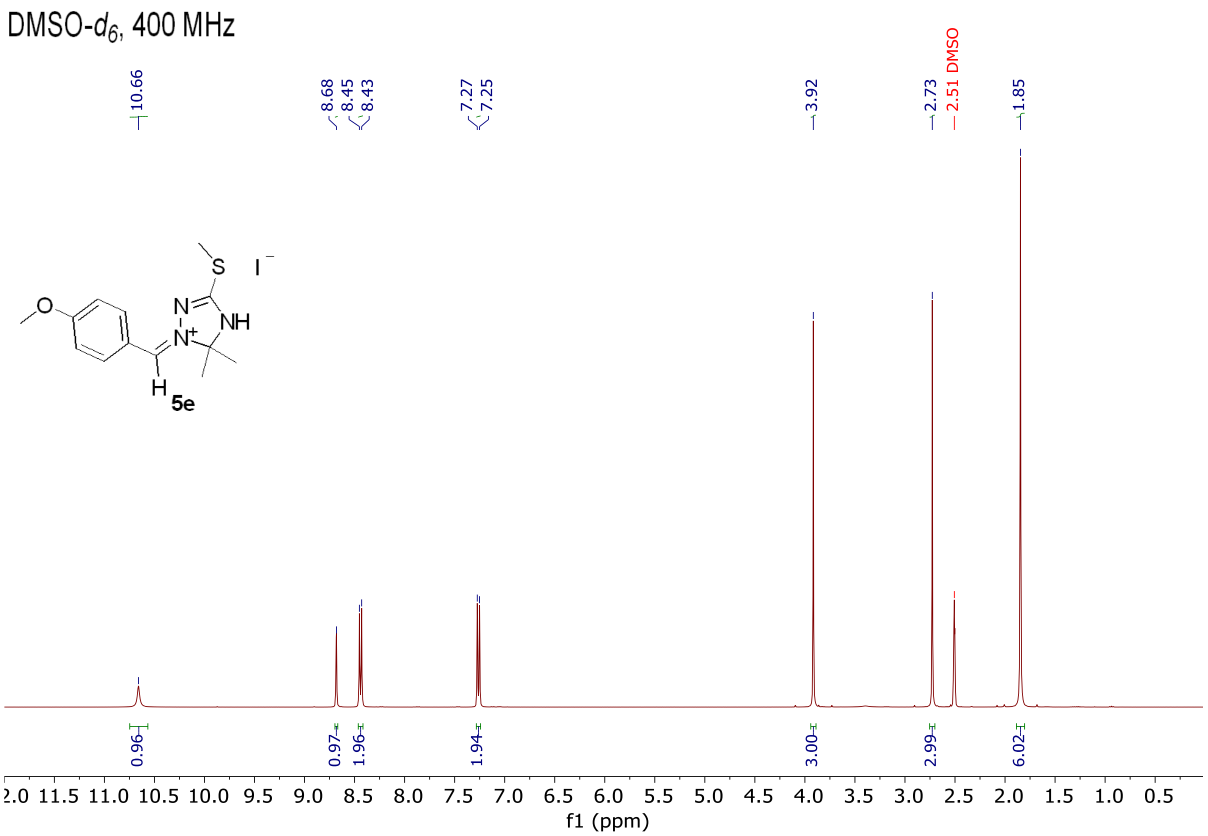

Figure S94. ^{1}H NMR spectrum (DMSO-d_6, 400 MHz) of **5e**.

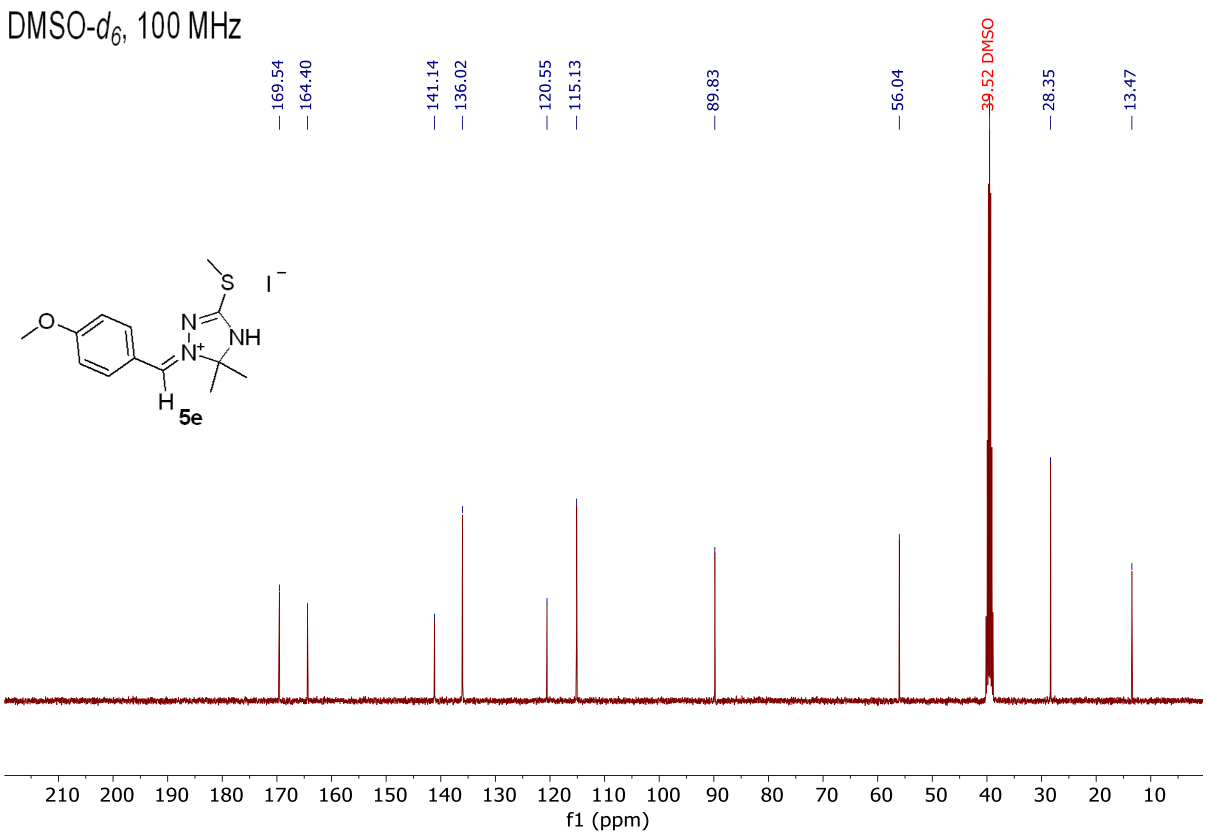

Figure S95. ^{13}C{^{1}H} NMR spectrum (DMSO-d_6, 100 MHz) of **5e**.

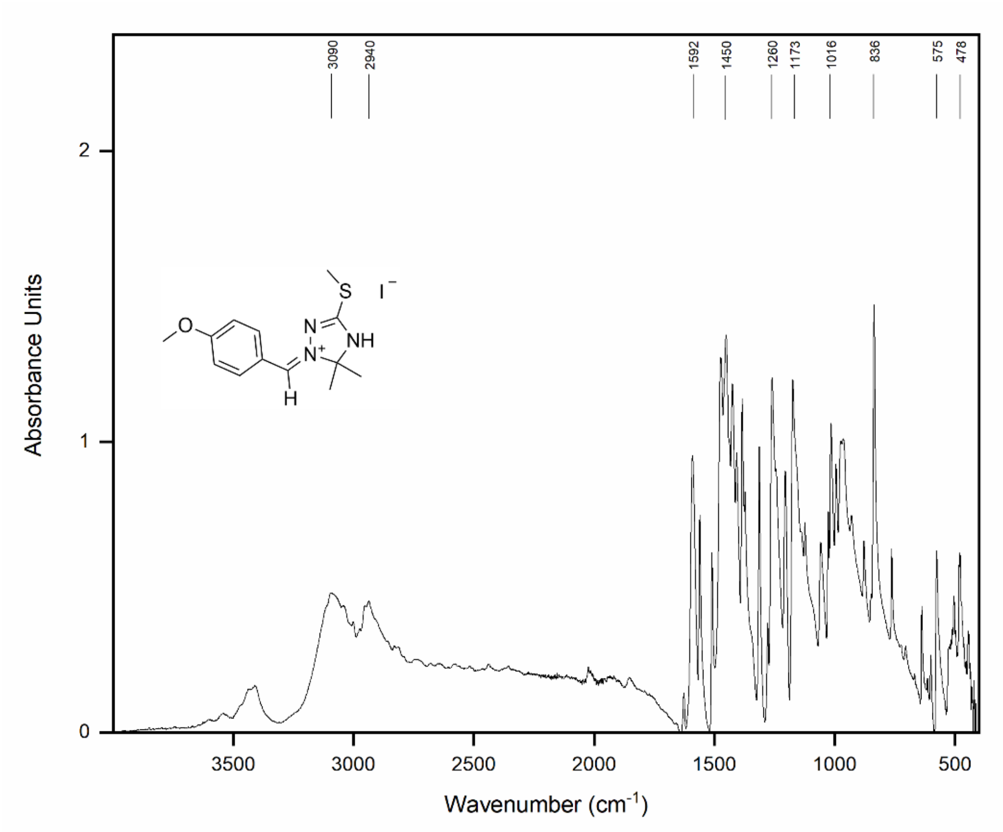

Figure S96. IR- absorption spectrum (neat) of **5e**.

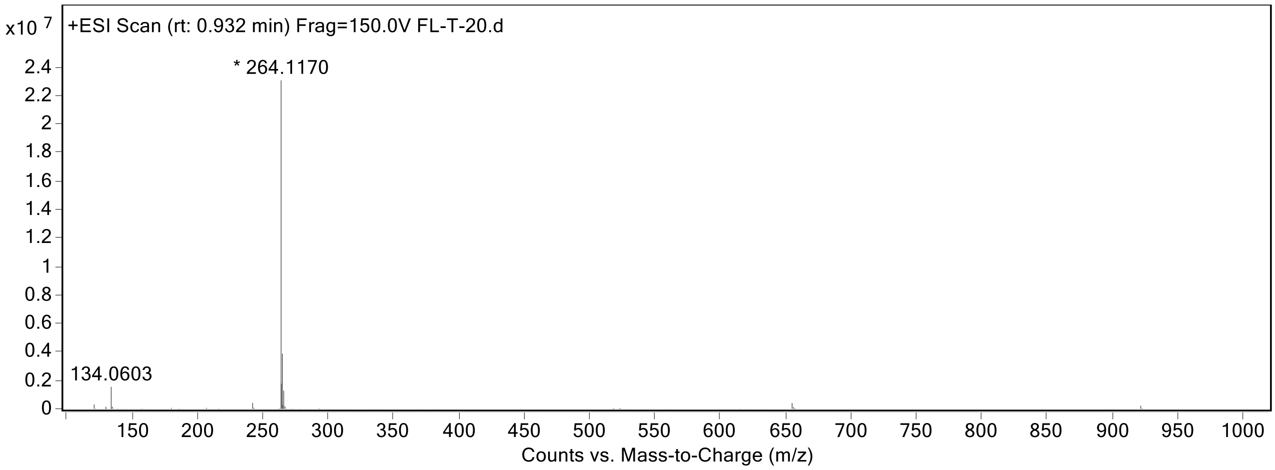

Figure S97. HR-ESI-MS (*m/z*) of **5e**. Measured peak for $[C_{13}H_{18}N_3S_1O_1]^+$

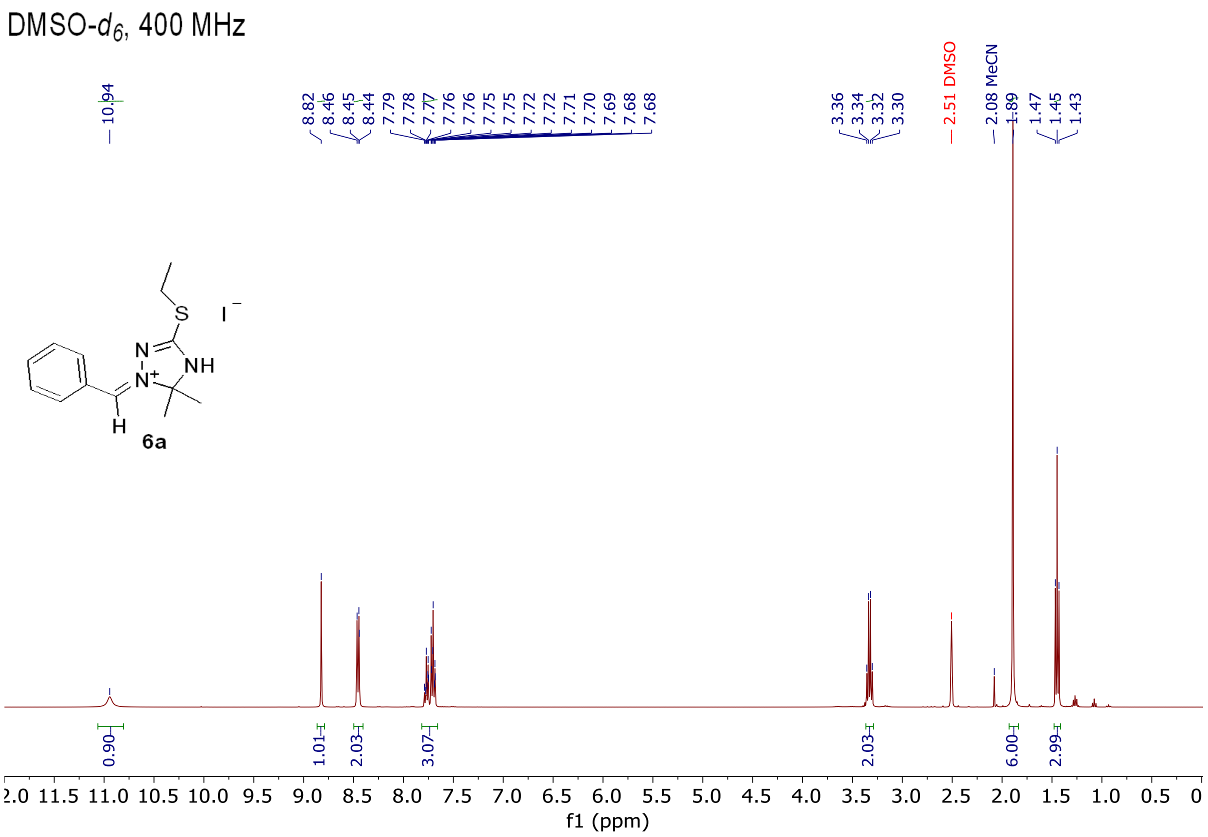

Figure S98. ^{1}H NMR spectrum (DMSO-d_6, 400 MHz) of **6a**.

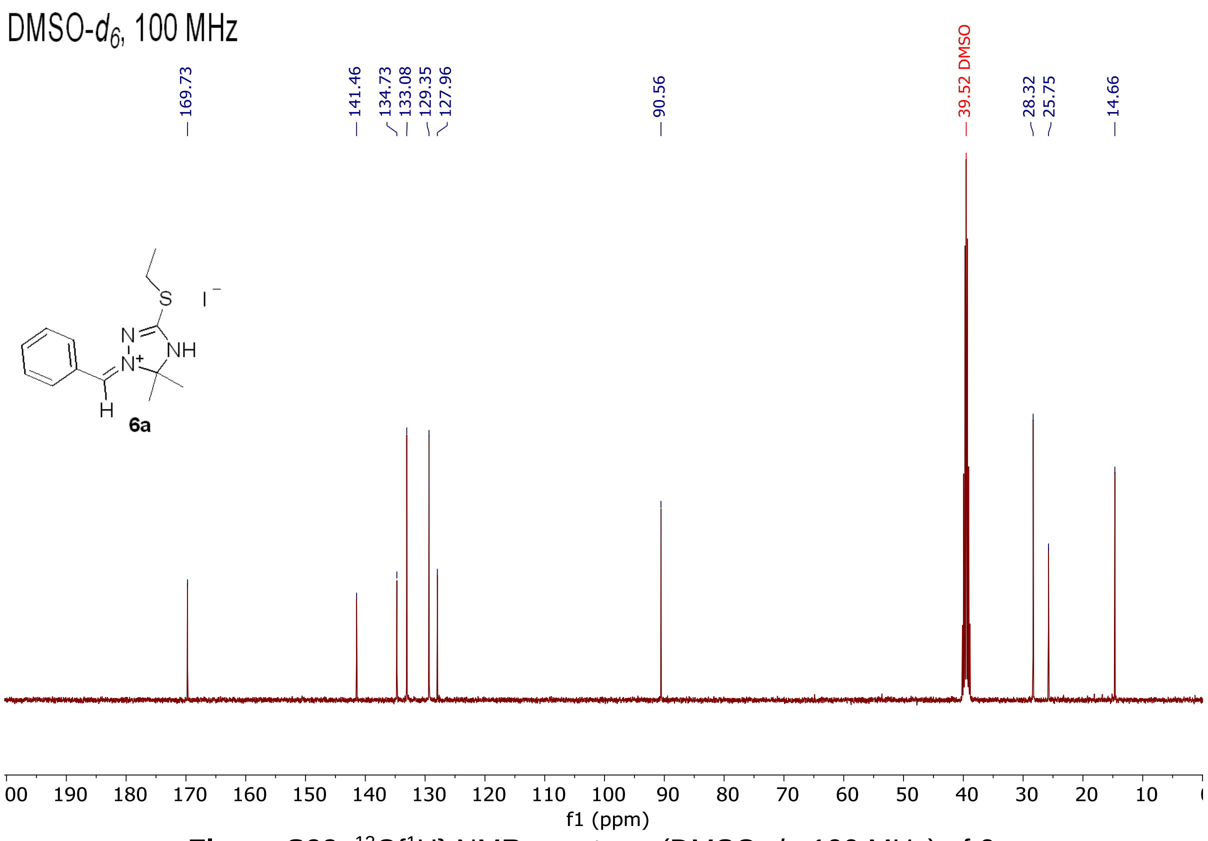

Figure S99. $^{13}C\{^{1}H\}$ NMR spectrum (DMSO-d_6, 100 MHz) of **6a**.

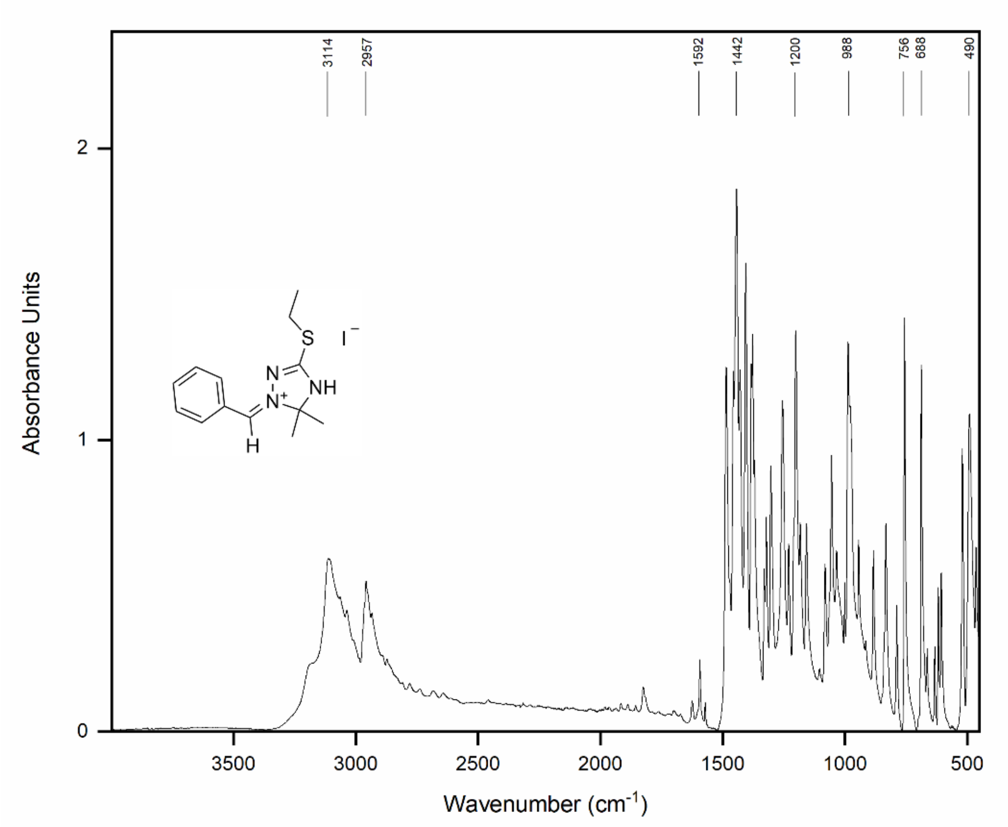

Figure S100. IR- absorption spectrum (neat) of **6a**.

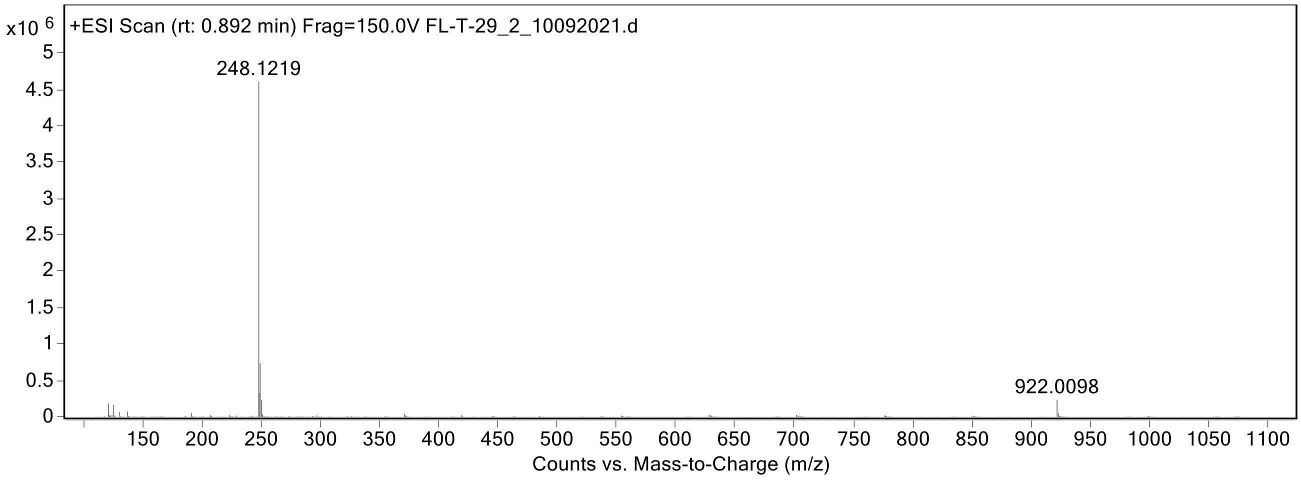

Figure S101. HR-ESI-MS (*m/z*) of **6a**. Measured peak for $[C_{13}H_{18}N_3S_1]^+$

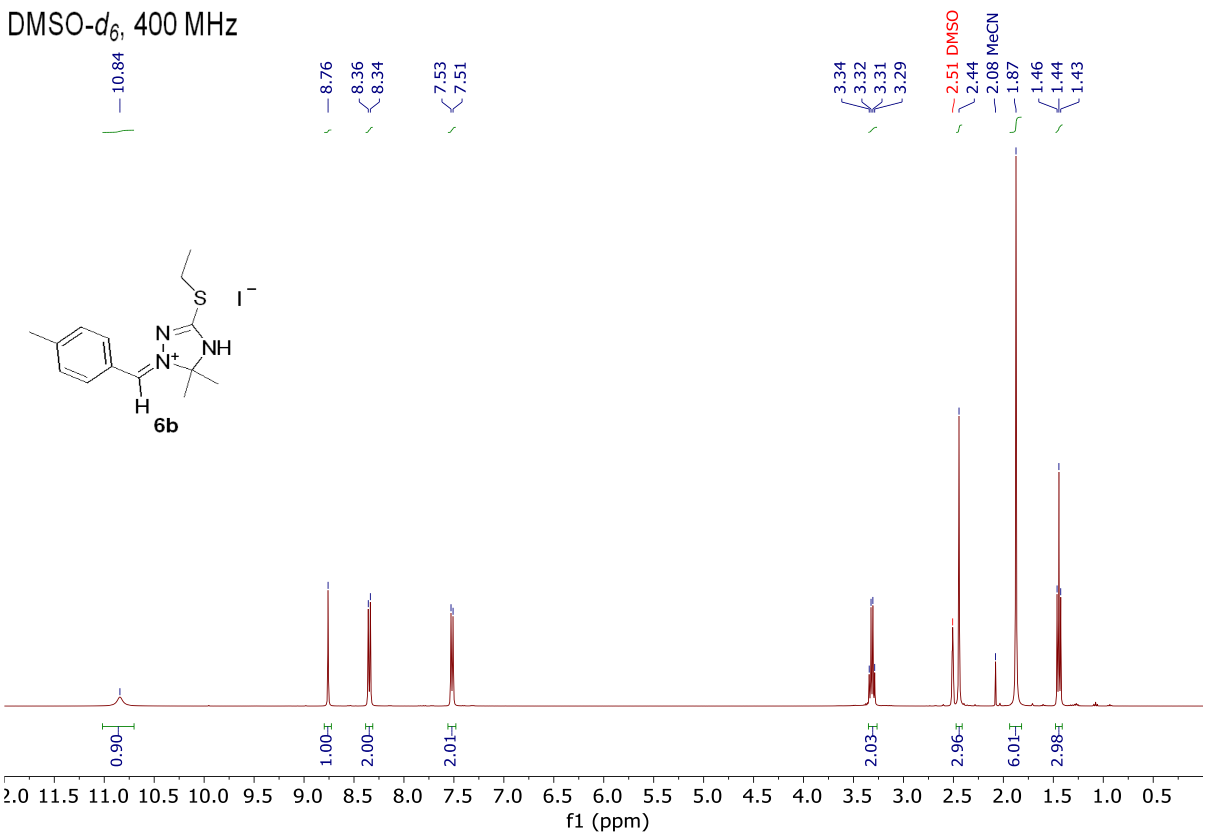

Figure S102. ^{1}H NMR spectrum (DMSO-d_6, 400 MHz) of **6b**.

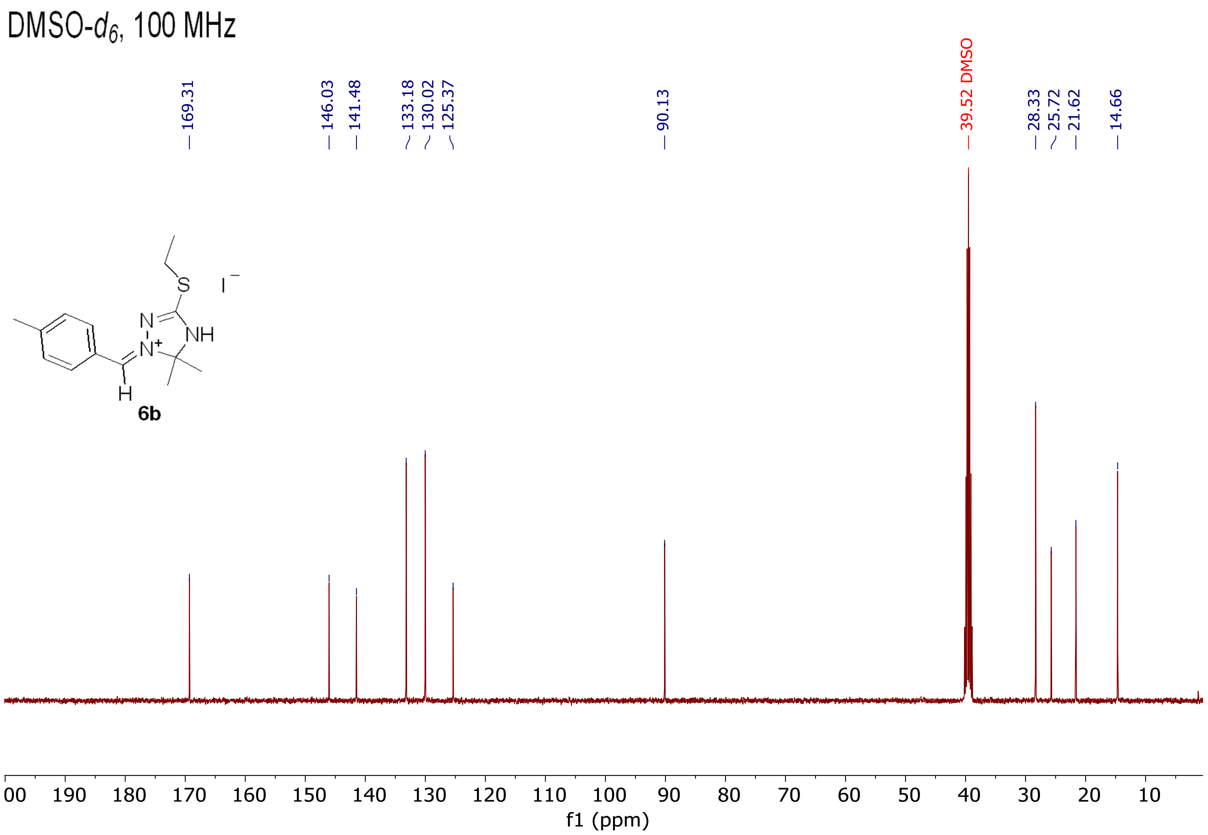

Figure S103. $^{13}C\{^{1}H\}$ NMR spectrum (DMSO-d_6, 100 MHz) of **6b**.

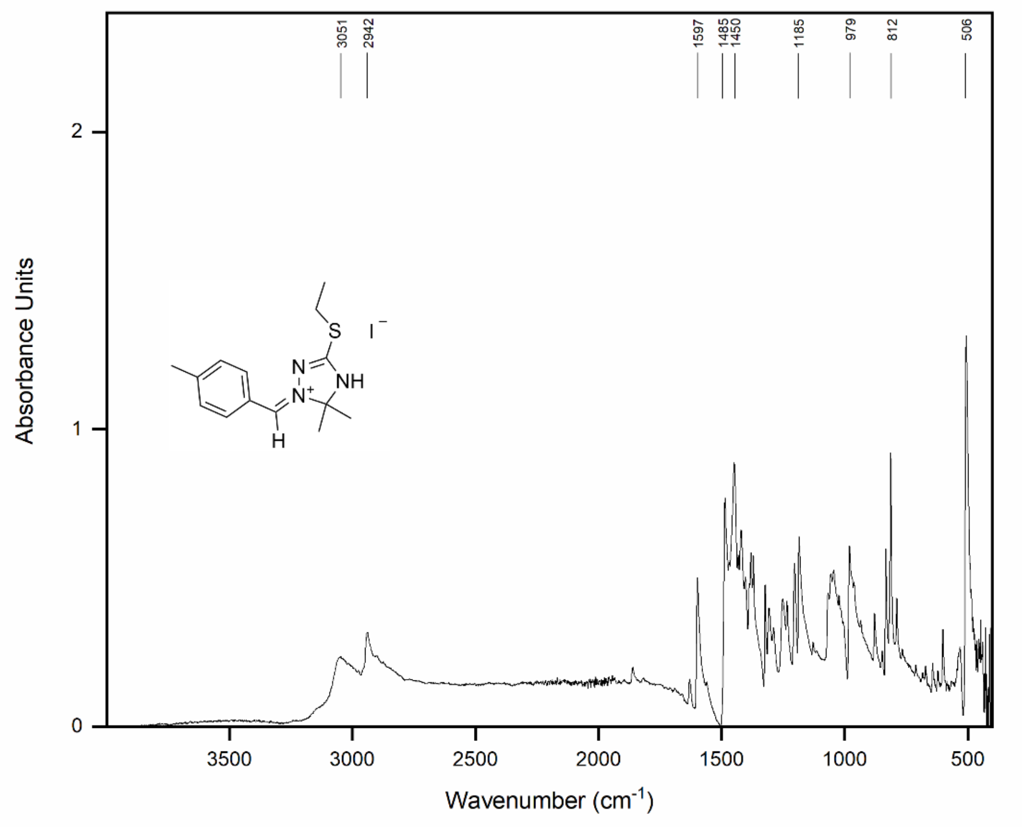

Figure S104. IR- absorption spectrum (neat) of **6b**.

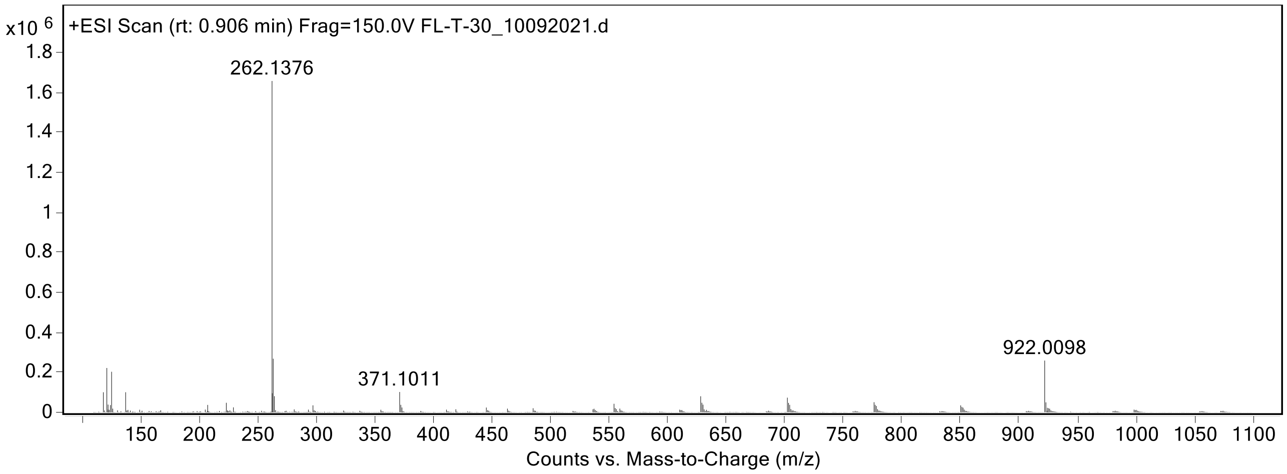

Figure S105. HR-ESI-MS (*m/z*) of **6b**. Measured peak for $[C_{14}H_{20}N_3S_1]^+$

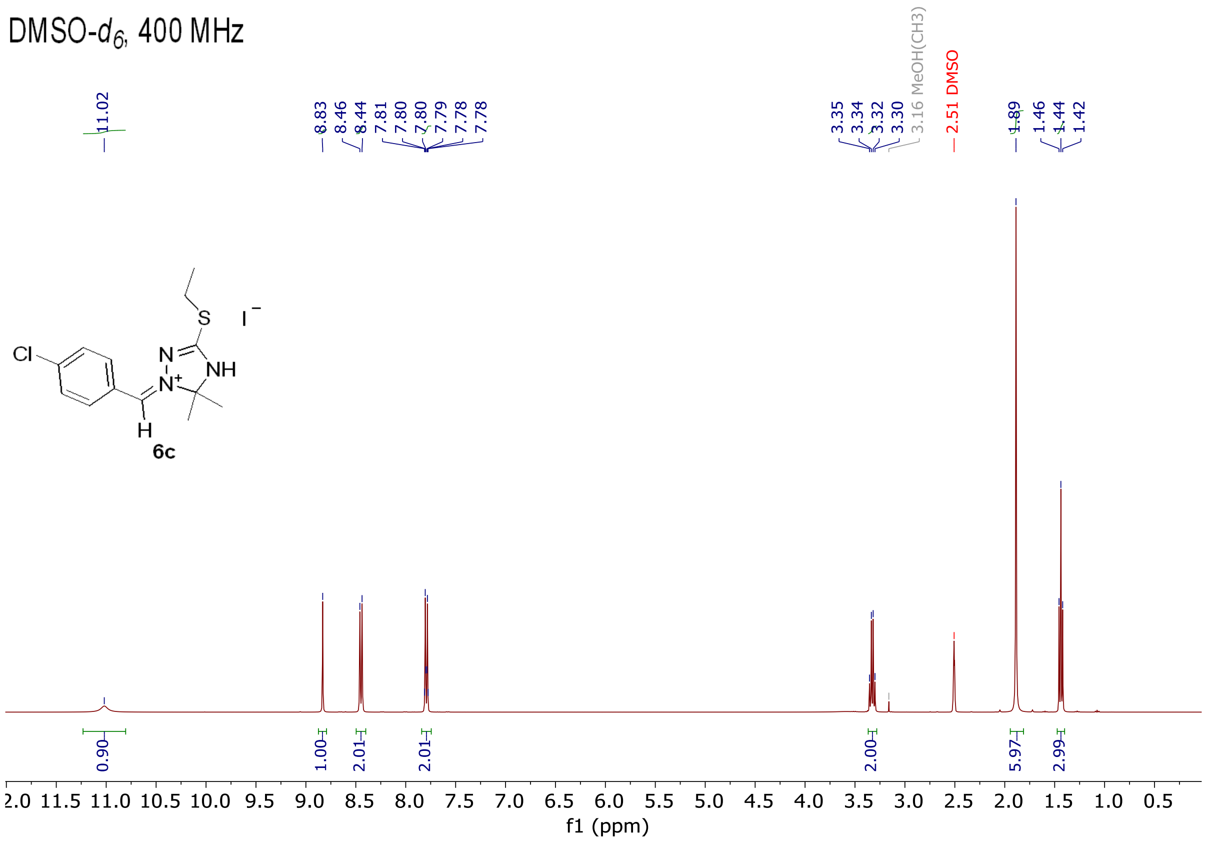

Figure S106. ^{1}H NMR spectrum (DMSO-d_6, 400 MHz) of **6c**.

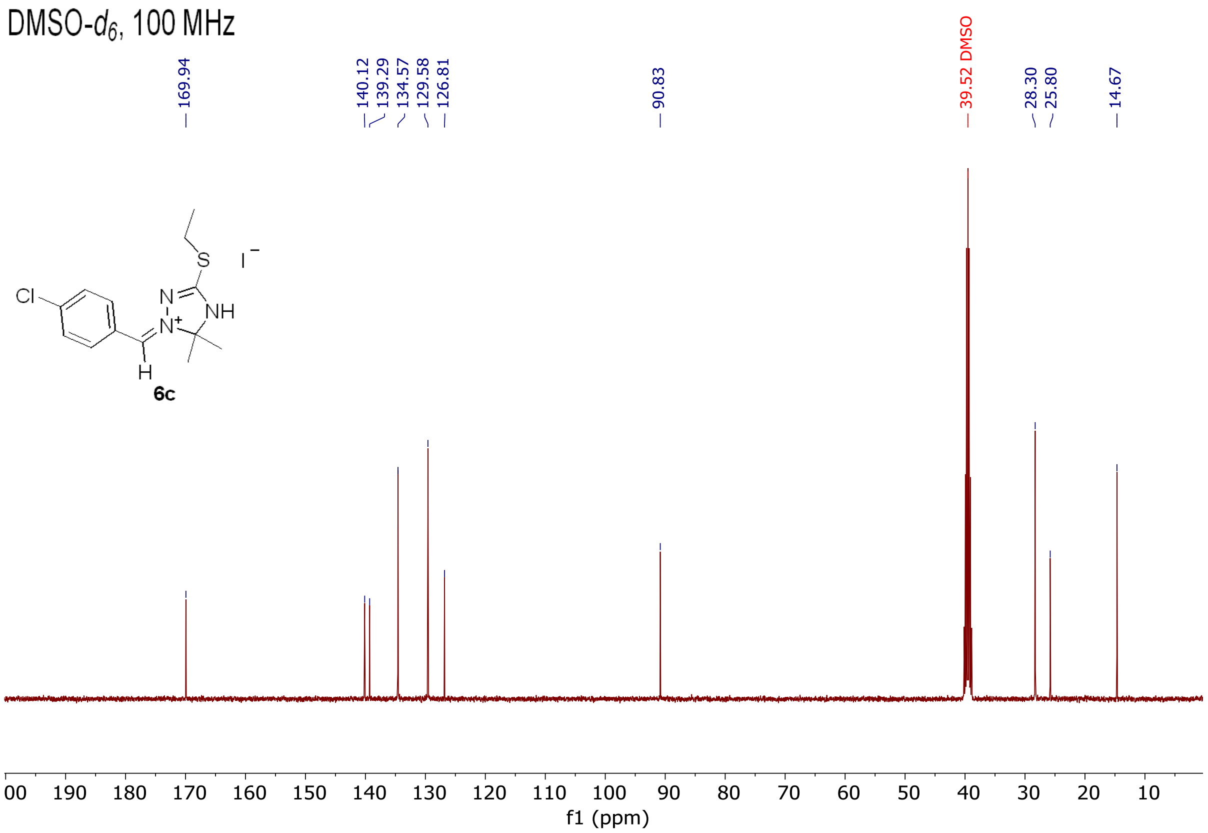

Figure S107. $^{13}C\{^{1}H\}$ NMR spectrum (DMSO-d_6, 100 MHz) of **6c**.

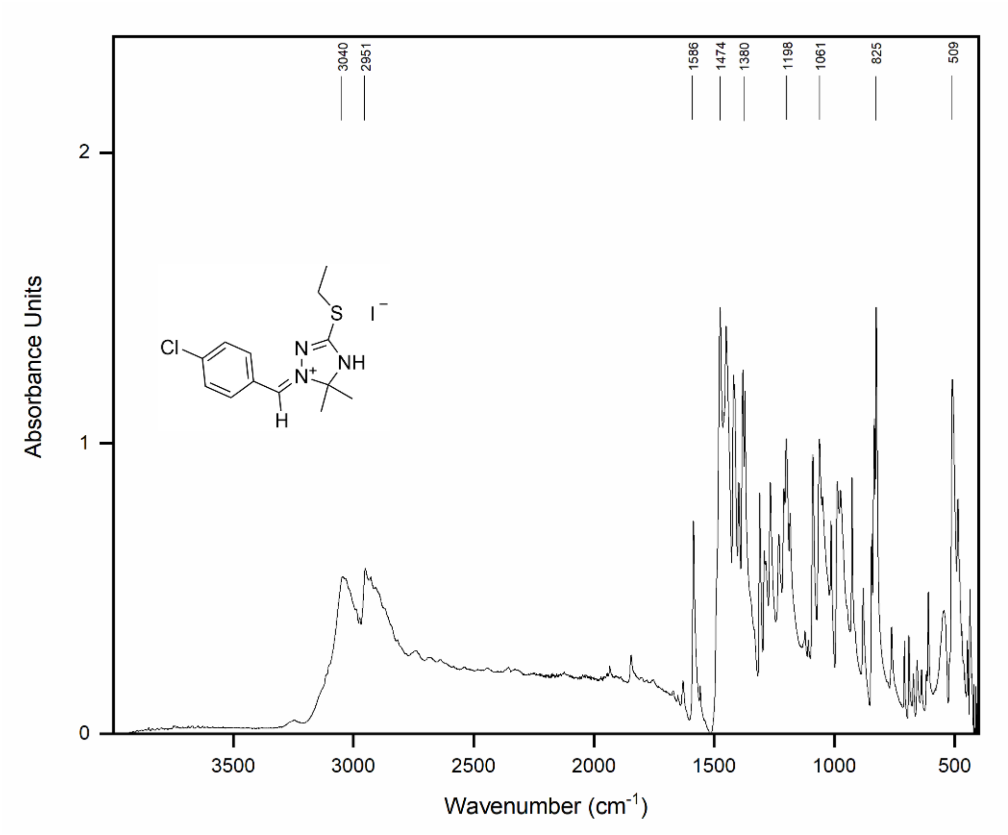

Figure S108. IR- absorption spectrum (neat) of **6c**.

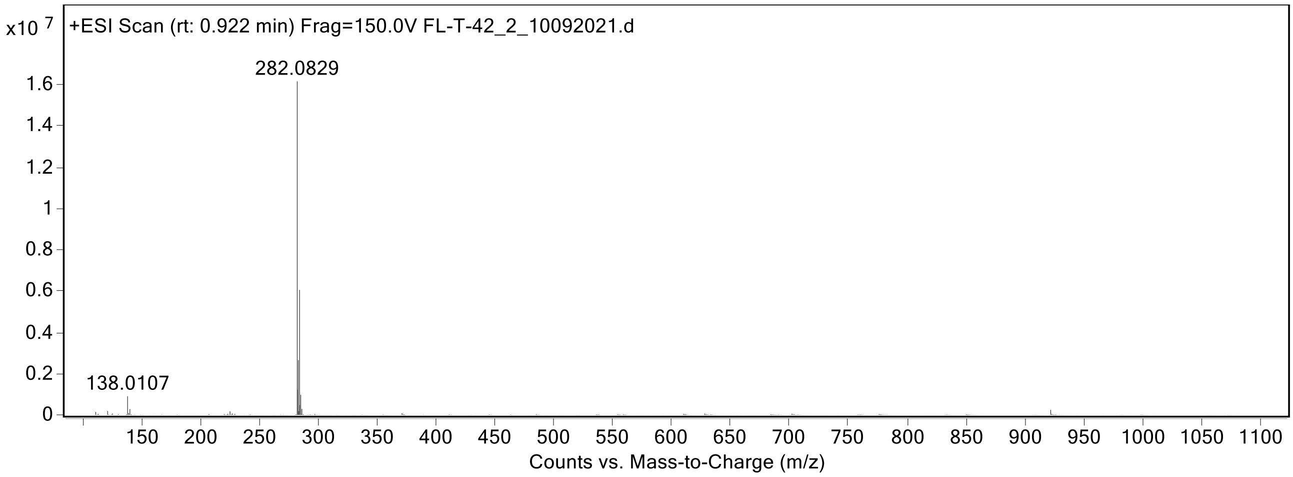

Figure S109. HR-ESI-MS (*m/z*) of **6c**. Measured peak for $[C_{13}H_{17}N_3S_1Cl_1]^+$

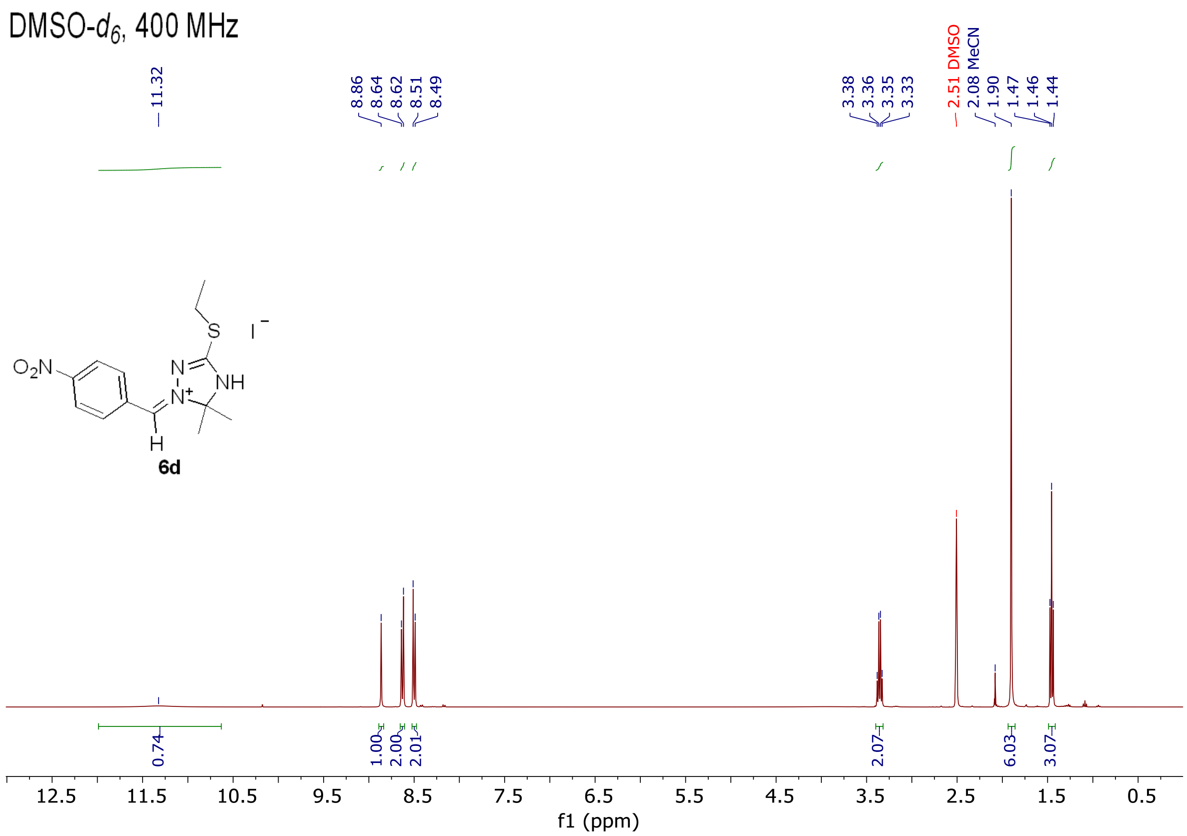

Figure S110. ^{1}H NMR spectrum (DMSO-d_6, 400 MHz) of **6d.**

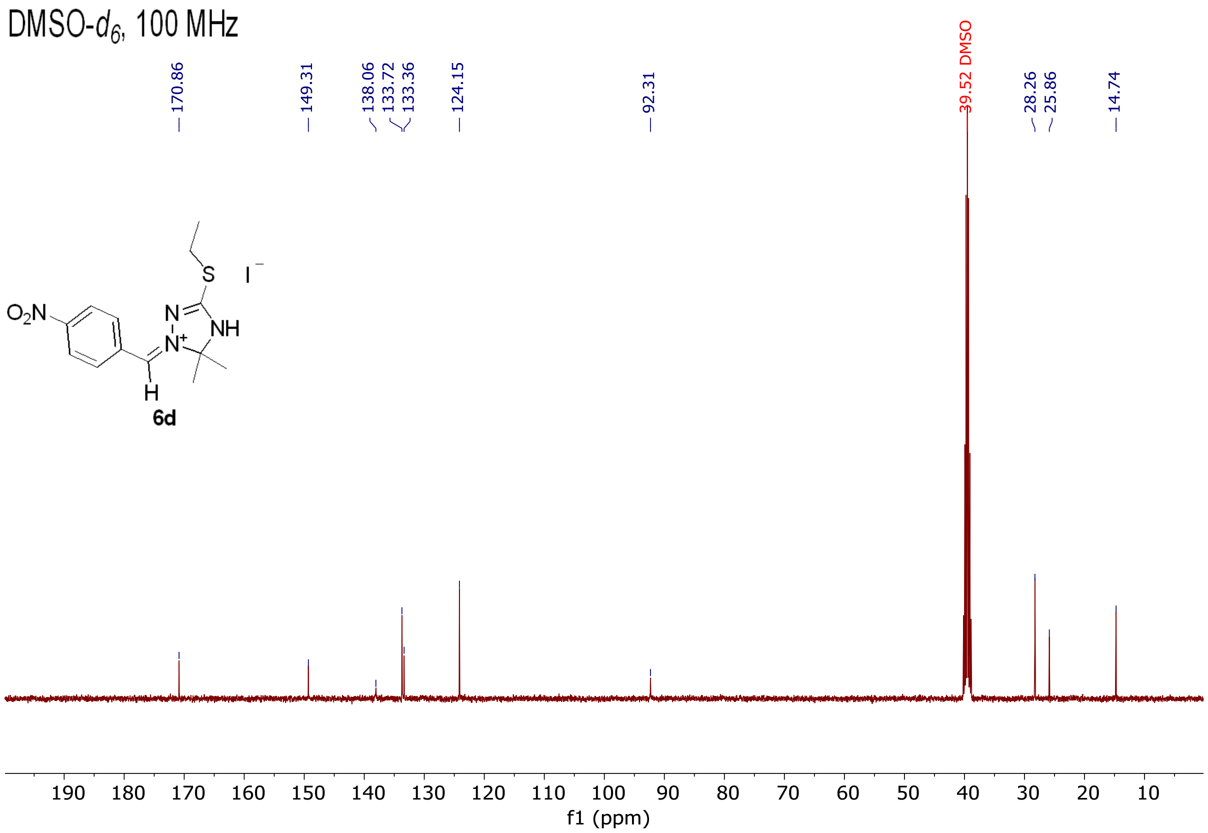

Figure S111. ^{13}C{^{1}H} NMR spectrum (DMSO-d_6, 100 MHz) **6d**.

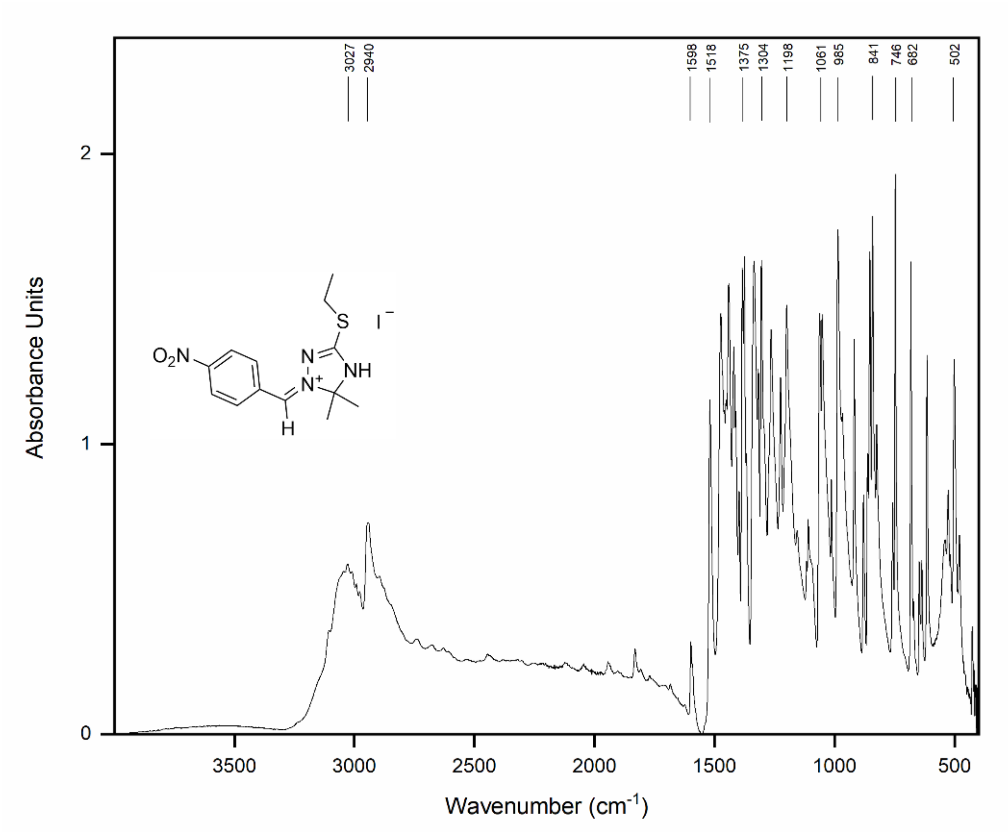

Figure S112. IR- absorption spectrum (neat) of **6d**.

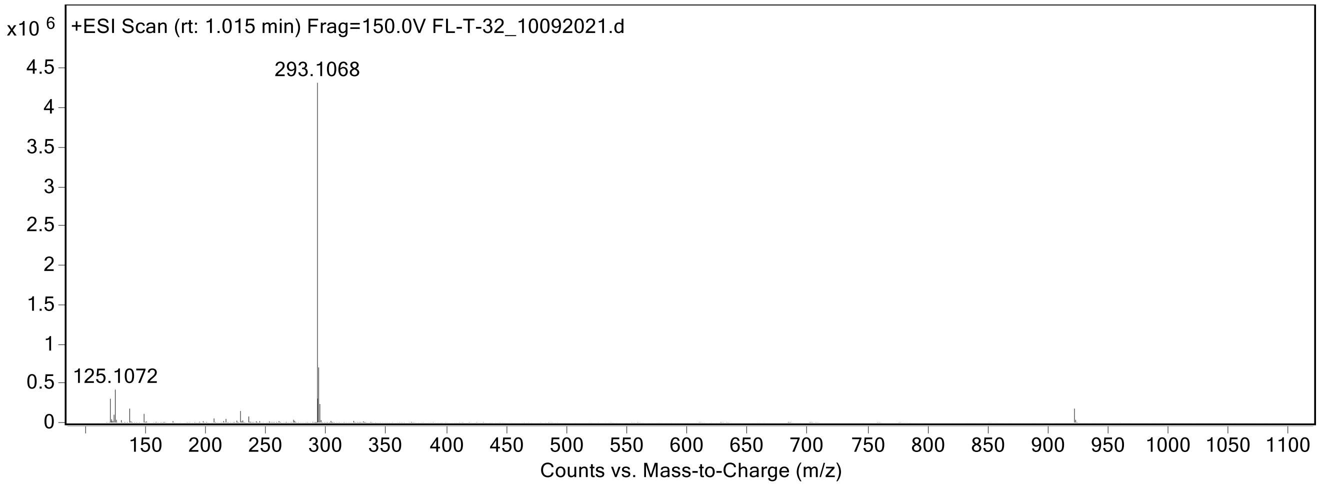

Figure S113. HR-ESI-MS (*m/z*) of **6d**. Measured peak for $[C_{13}H_{17}N_4S_1O_2]^+$

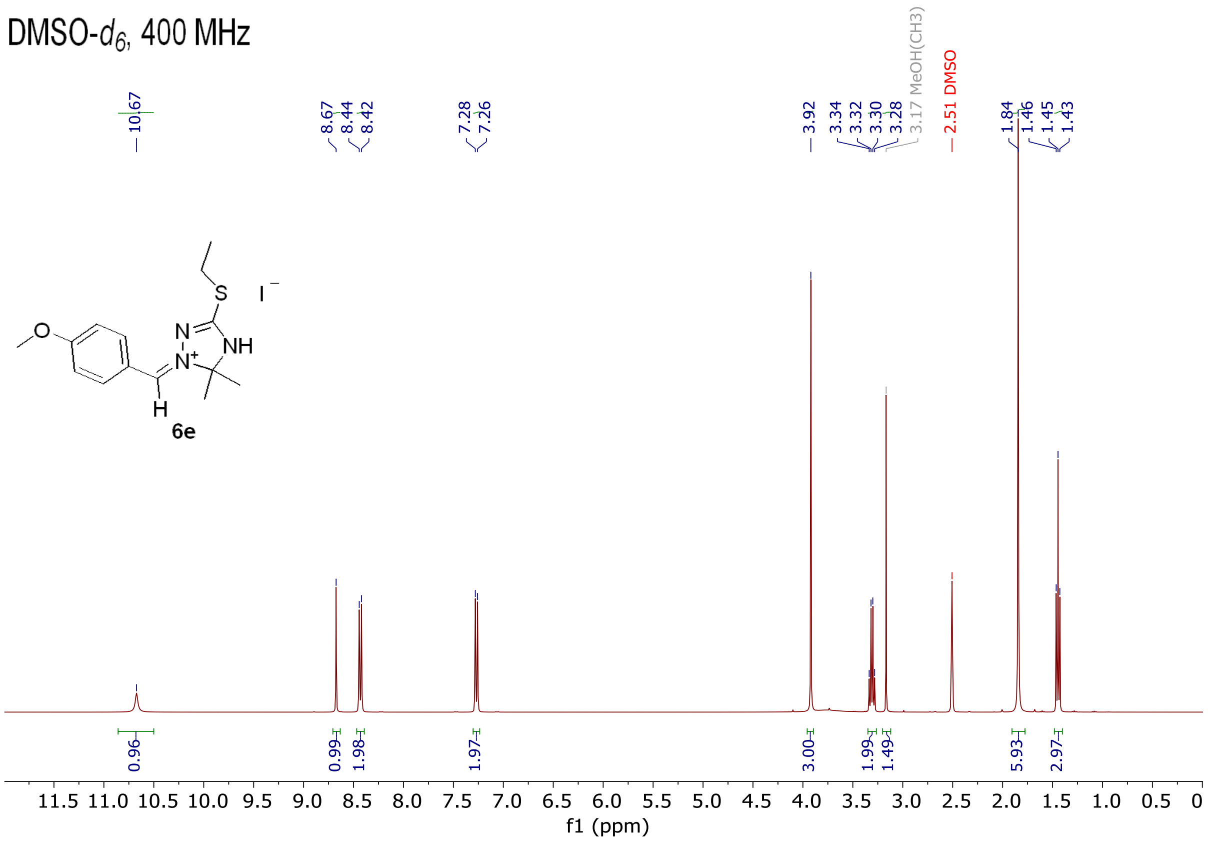

Figure S114. ^{1}H NMR spectrum (DMSO-d_6, 400 MHz) of **6e**.

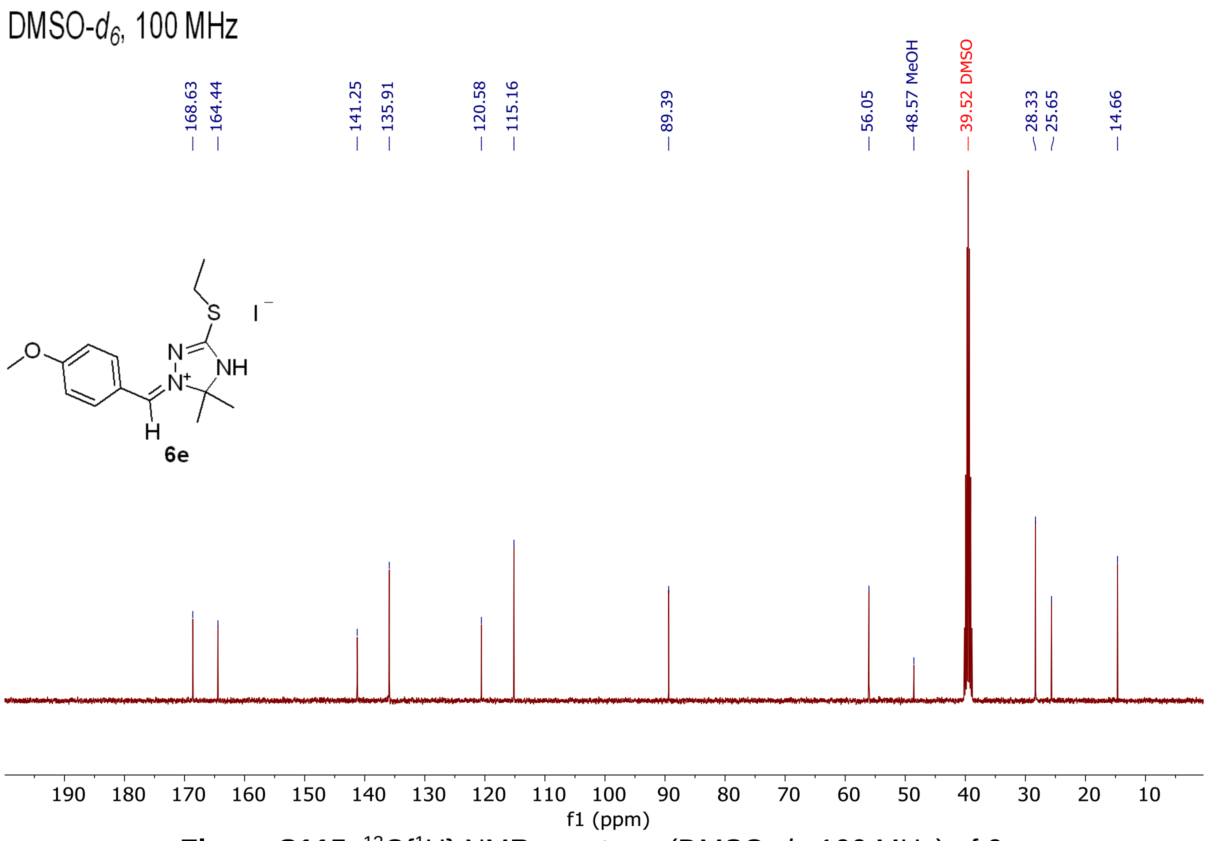

Figure S115. $^{13}C\{^1H\}$ NMR spectrum (DMSO-d_6, 100 MHz) of **6e**.

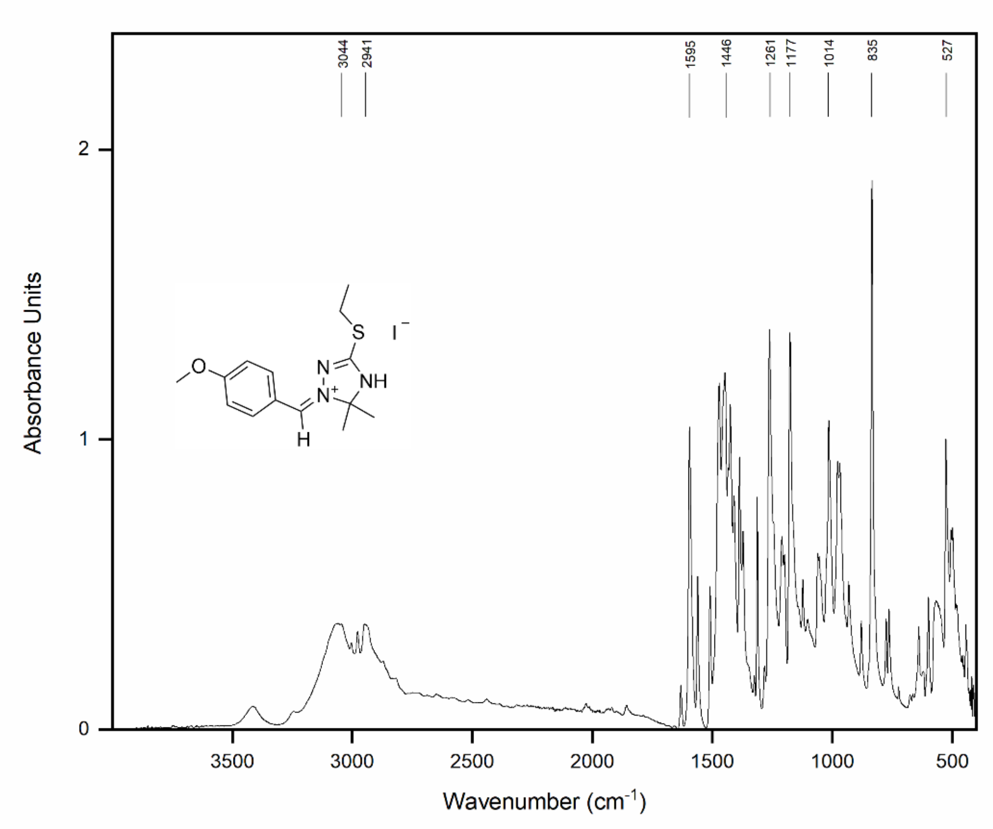

Figure S116. IR- absorption spectrum (neat) of **6e**.

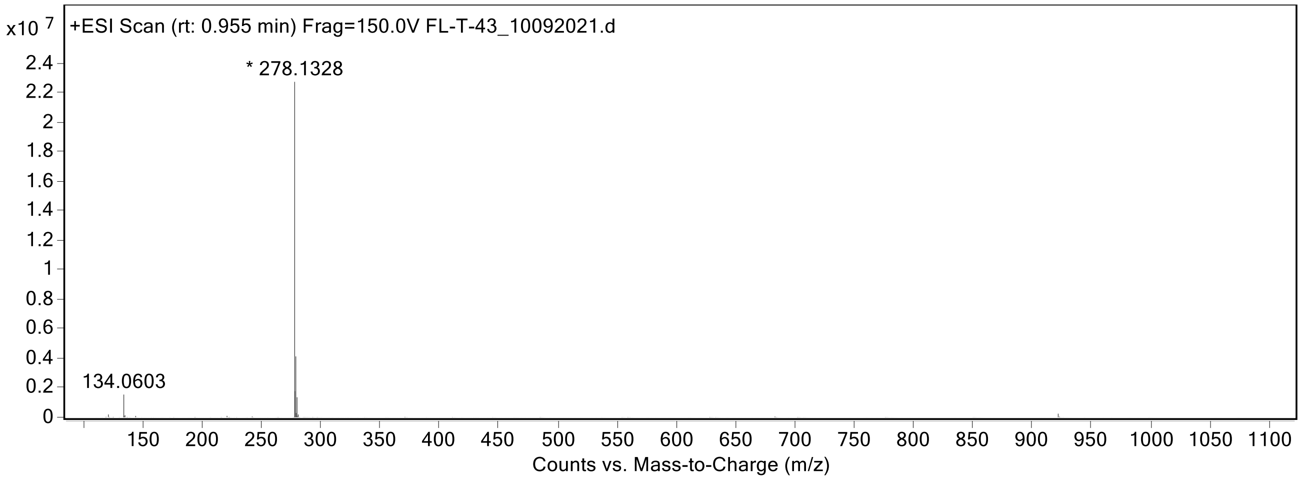

Figure S117. HR-ESI-MS (*m/z*) of **6e**. Measured peak for $[C_{14}H_{20}N_{3}S_{1}O_{1}]^{+}$

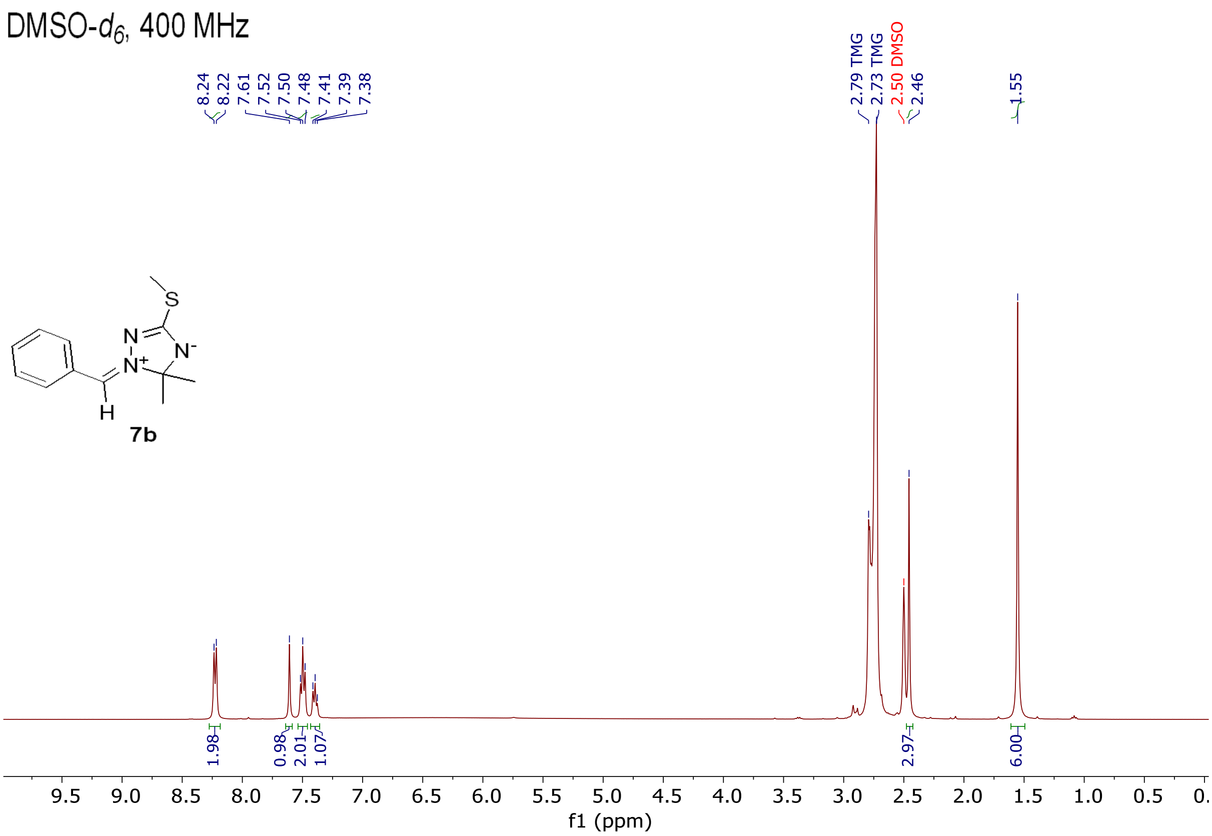

Figure S118. ^{1}H NMR spectrum (DMSO-d_6, 400 MHz) of **7b** obtained through *in-situ* deprotonation of **2b** with TMG.

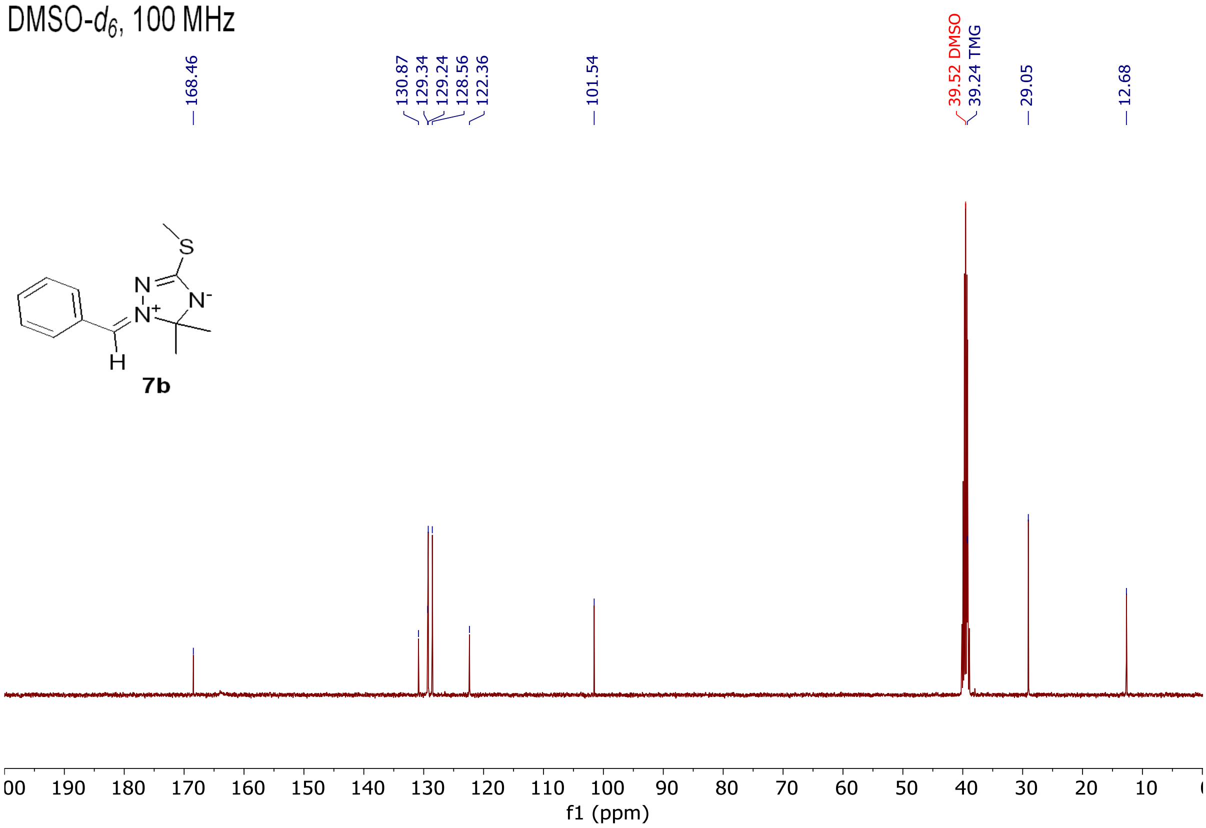

Figure S119. ^{13}C{^{1}H} NMR spectrum (DMSO-d_6, 100 MHz) of **7b** obtained through *in situ* deprotonation of **2b** with TMG.

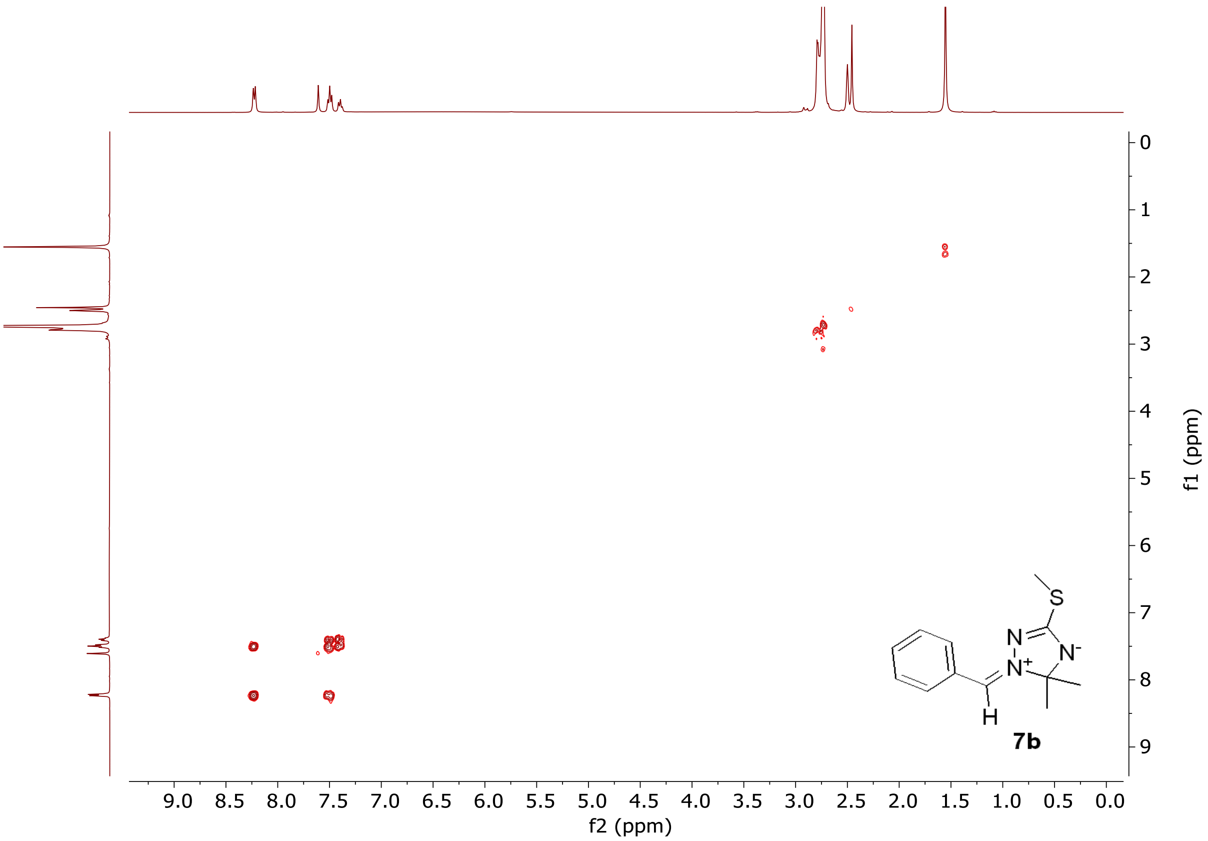

Figure S120. COSY spectrum (DMSO-d_6) of **7b** obtained through *in situ* deprotonation of **2b** with TMG.

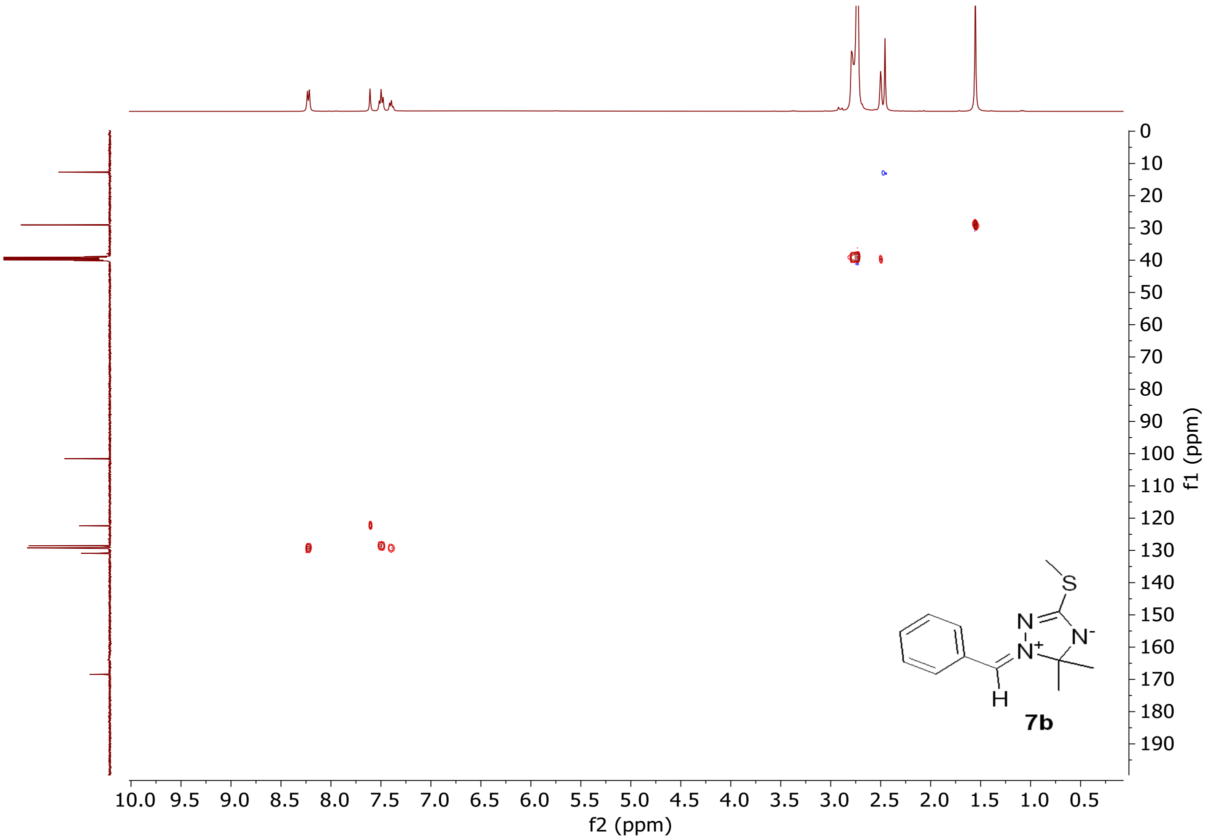

Figure S121. HSQC spectrum (DMSO-d_6) of **7b** obtained through *in situ* deprotonation of **2b** with TMG.

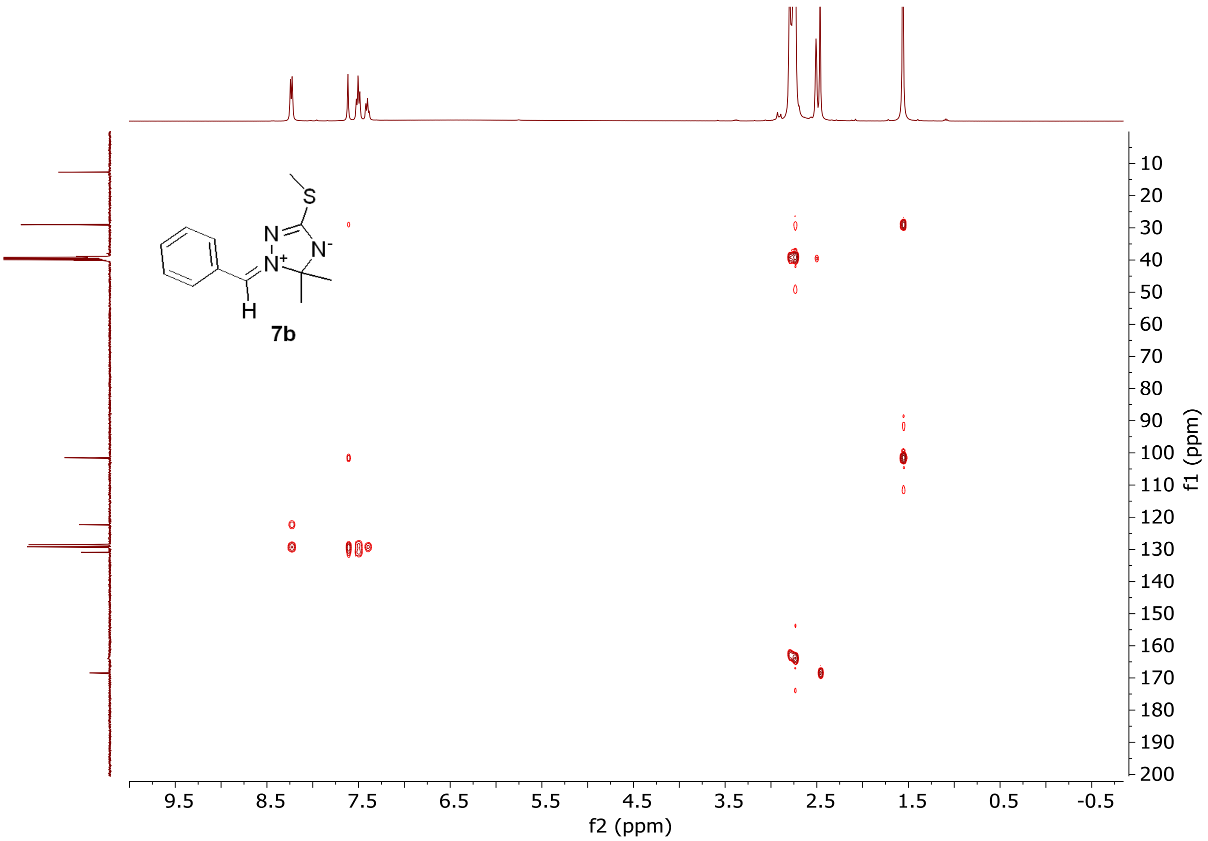

Figure S122. HMBC spectrum (DMSO-d_6) of **7b** obtained through *in situ* deprotonation of **2b** with TMG.

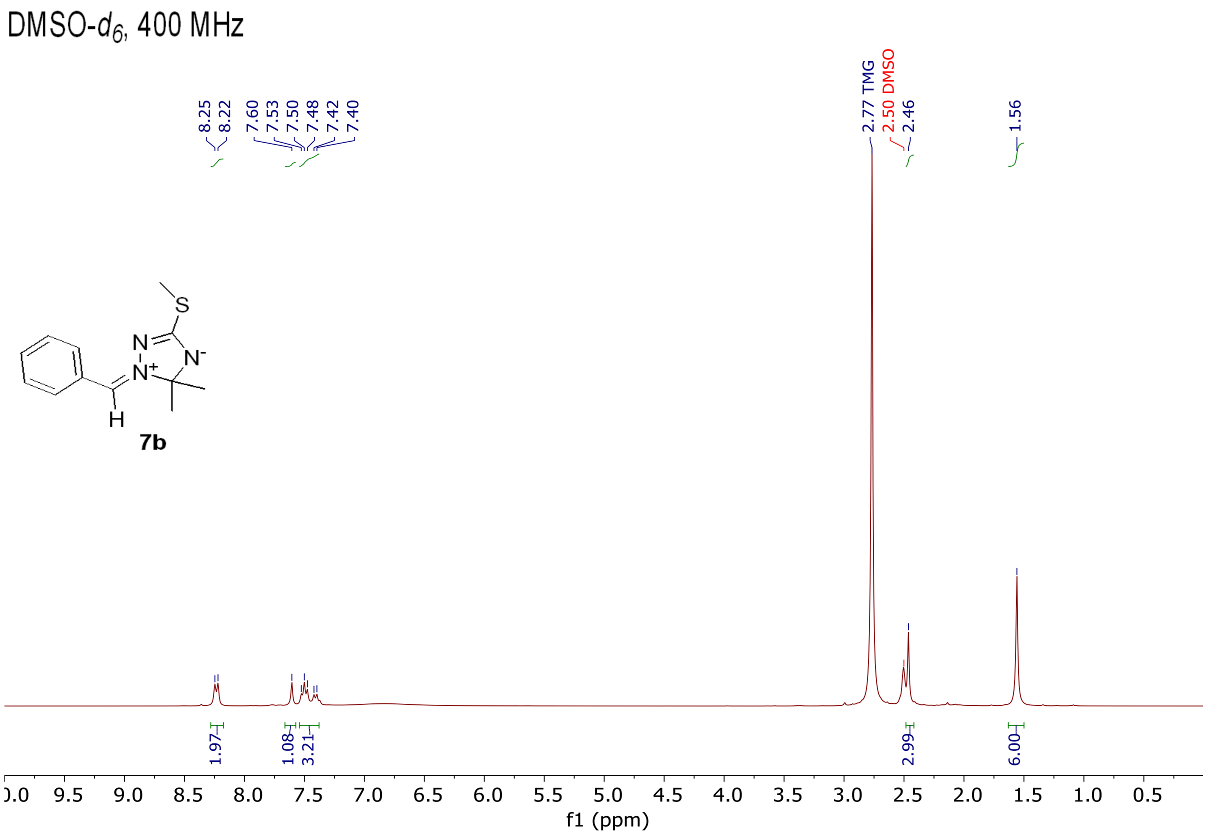

Figure S123. ^{1}H NMR spectrum (DMSO-d_6, 400 MHz) of **7b** after heating to 95 °C for 1h. No significant changes were discernable.

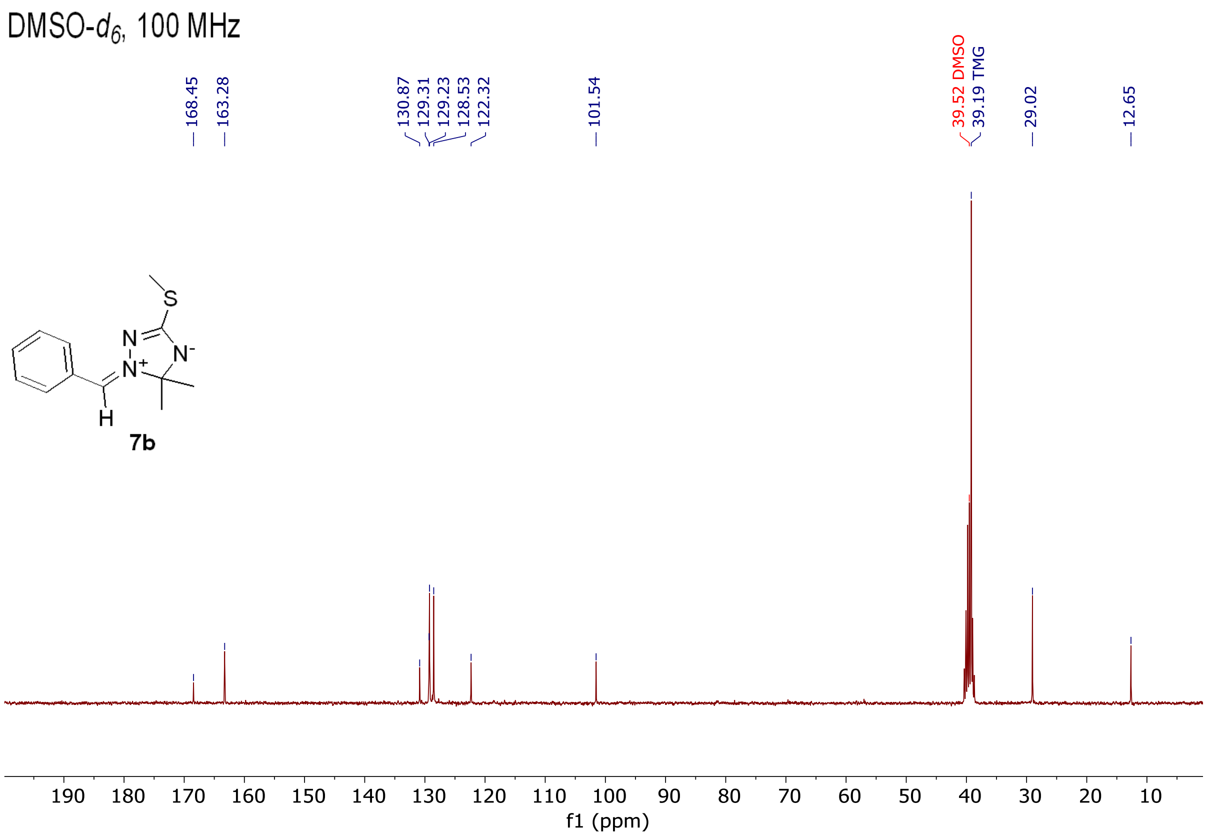

Figure S124. $^{13}C\{^{1}H\}$ NMR spectrum (DMSO-d_6, 100 MHz) of **7b** after incubation at 95 °C for 1h. No significant changes were discernable.

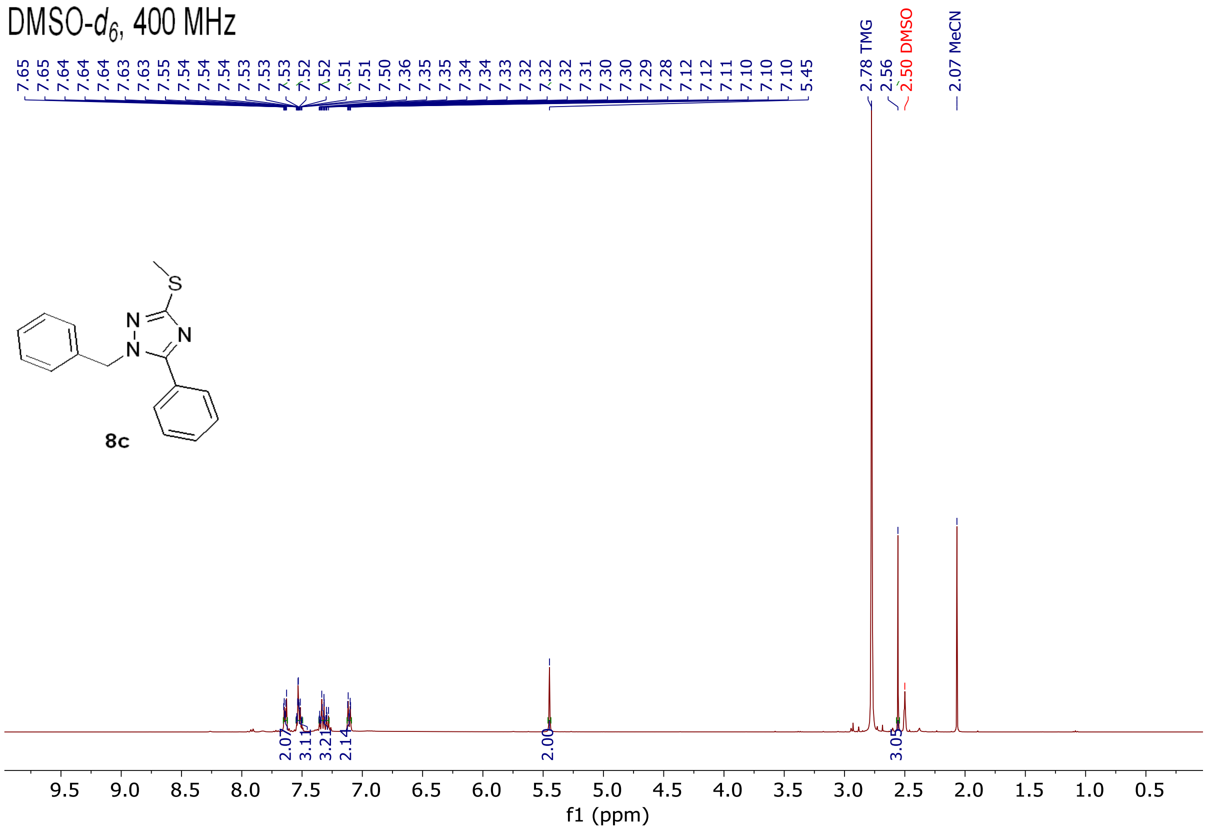

Figure S125. ^{1}H NMR spectrum (DMSO-d_6, 400 MHz) of **8c** obtained through *in-situ* deprotonation of **2c** with TMG followed by a hydride shift reaction.

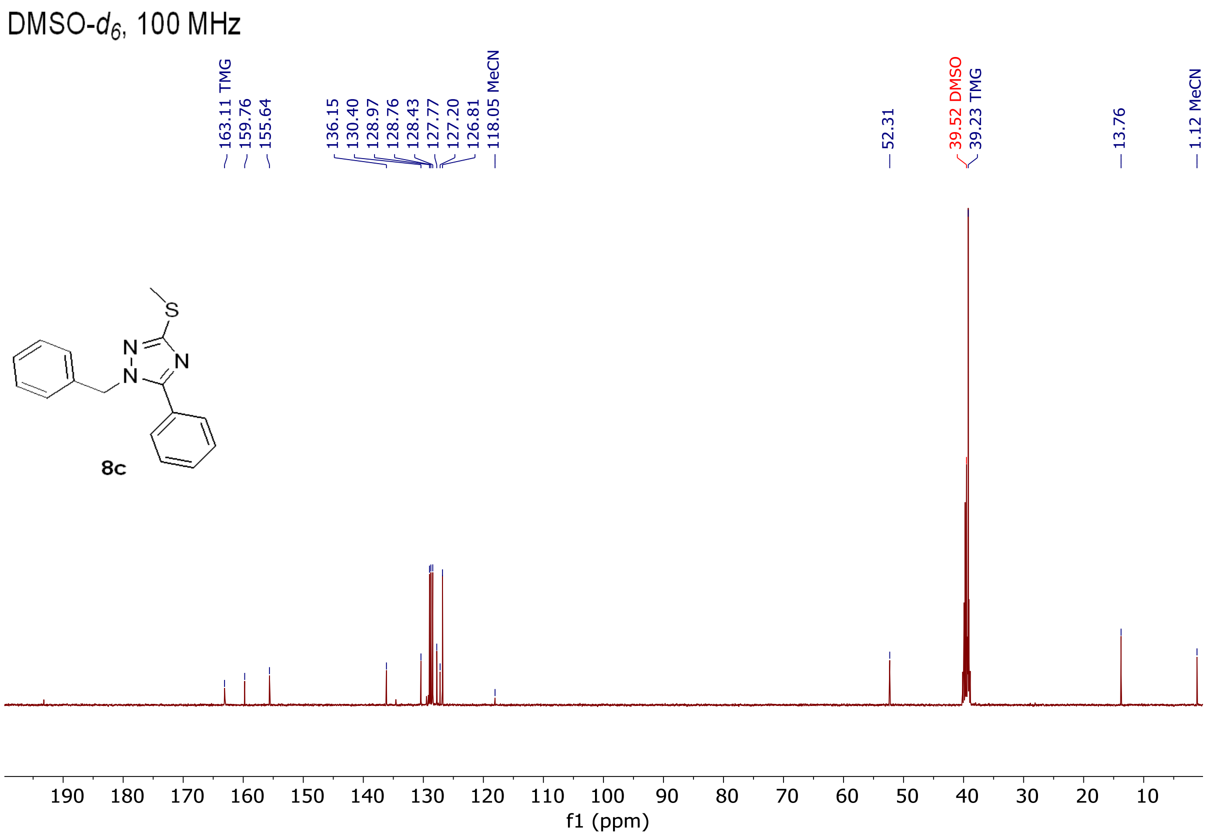

Figure S126. $^{13}C\{^1H\}$ NMR spectrum (DMSO-d_6, 100 MHz) of **8c** obtained through *in situ* deprotonation of **2c** with TMG followed by a hydride shift reaction.

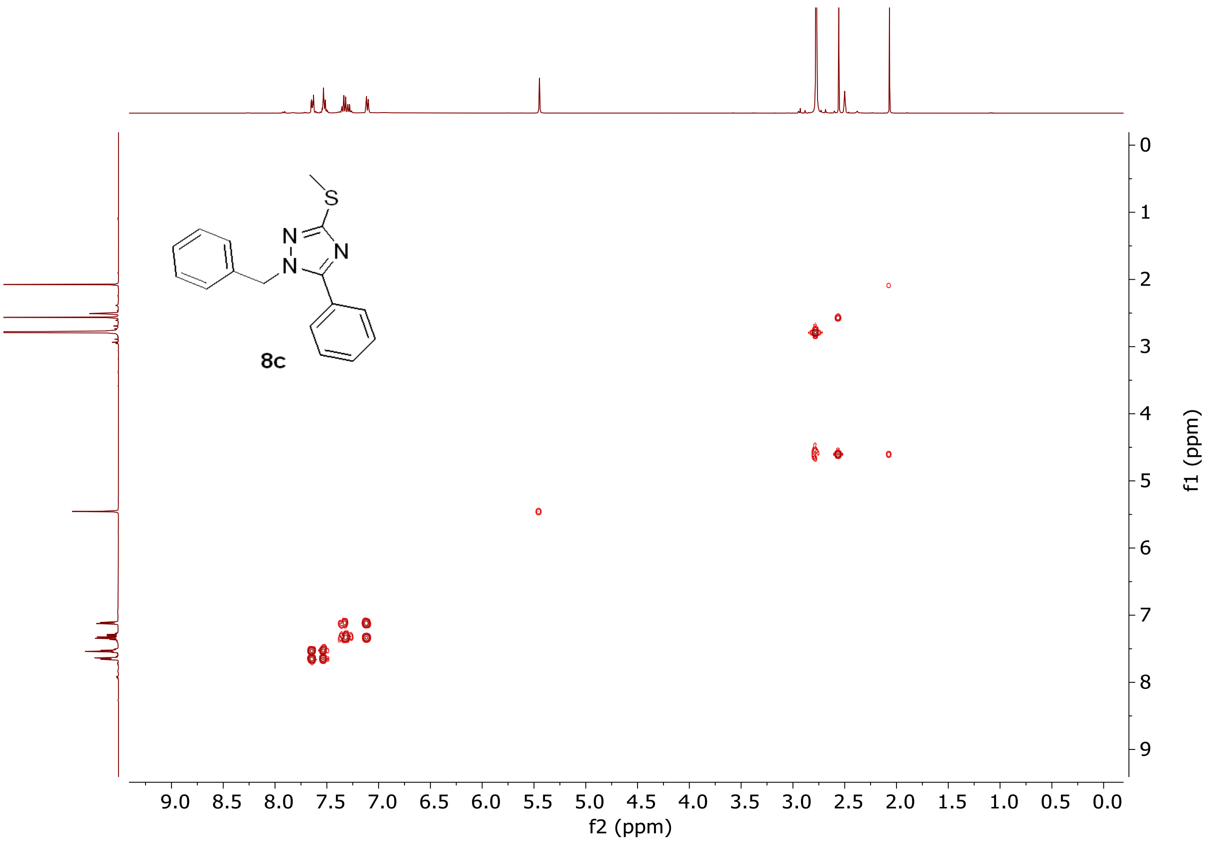

Figure S127. COSY spectrum (DMSO-d_6) of **8c** obtained through *in situ* deprotonation of **2c** with TMG followed by a hydride shift reaction.

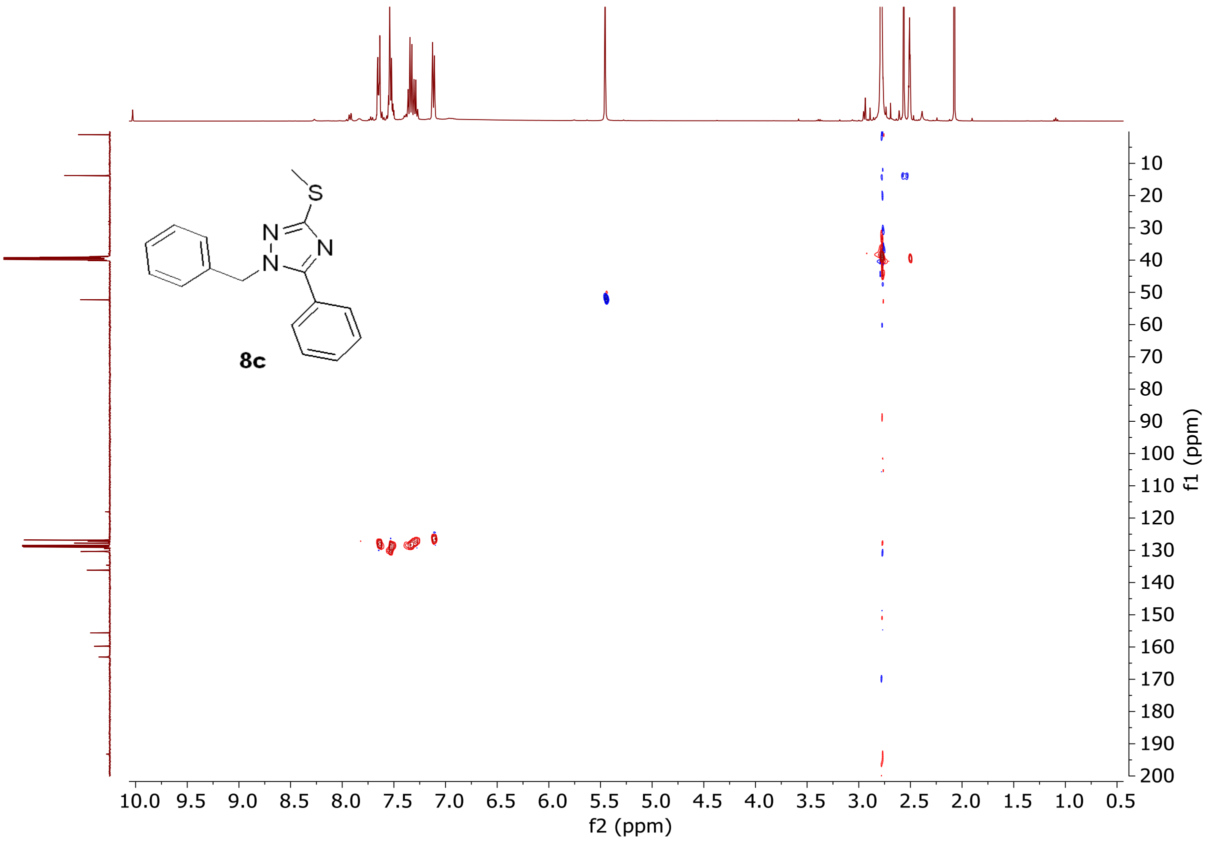

Figure S128. HSQC spectrum (DMSO-d_6) of **8c** obtained through *in situ* deprotonation of **2c** with TMG followed by a hydride shift reaction.

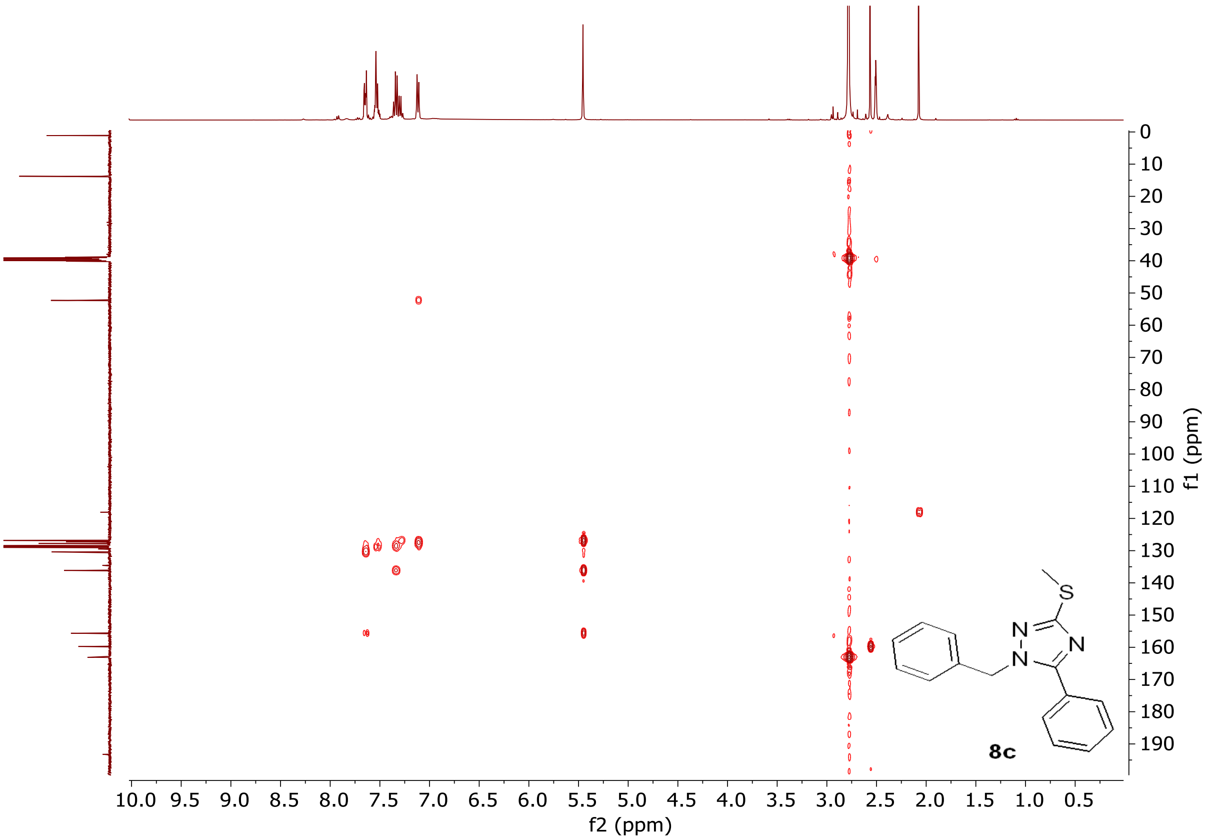

Figure S129. HMBC spectrum (DMSO-d_6) of **8c** obtained through *in situ* deprotonation of **2c** with TMG followed by a hydride shift reaction.

6) DFT Computational Data

Isothiosemicarbazonium Educts:

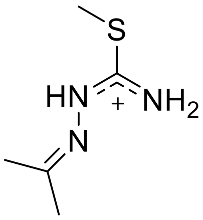

1a

1a E(RB3LYP) = -759.515671495 A.U. after 7 cycles Number of imaginary frequencies: 0

Center Number	Atomic Number	Coordinates (Angstroms) X	Y	Z
1	7	-1.363543	0.517310	-0.000096
2	6	0.948995	0.244141	-0.000001
3	16	2.302833	-0.858582	0.000078
4	6	3.741360	0.262078	0.000164
5	6	-2.534387	-0.011941	-0.000071
6	6	-3.704782	0.918567	-0.000192
7	6	-2.791174	-1.497165	0.000073
8	7	1.047951	1.560471	-0.000072
9	1	4.605095	-0.410139	0.000256
10	1	3.774000	0.869963	-0.909500
11	1	3.773844	0.870018	0.909796
12	1	-3.384797	1.963420	-0.000232
13	1	-4.334606	0.730924	-0.881228
14	1	-4.334730	0.731018	0.880773
15	1	-2.360804	-1.978348	-0.890602
16	1	-2.360779	-1.978181	0.890825
17	1	-3.862780	-1.710977	0.000106
18	1	0.175376	2.087964	-0.000134
19	1	1.936197	2.040892	-0.000146
20	1	-0.352879	-1.328091	0.000086
21	7	-0.267057	-0.312814	0.000013

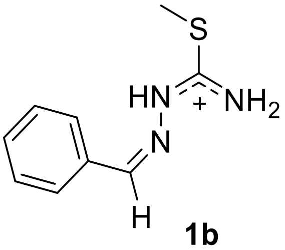

1b

1b E(RB3LYP) = -911.846562713 A.U. after 7 cycles Number of imaginary frequencies: 0

Center Number	Atomic Number	Coordinates (Angstroms) X	Y	Z	NMR shielding tensors isotropic	anisotropic
1	7	0.106672	-0.034920	-0.000335	-47.6990	336.9637
2	6	2.436559	0.109684	-0.000052	6.8712	161.5598
3	16	3.957322	-0.751098	0.000052	456.3967	351.7332
4	6	5.185674	0.596223	0.000354	162.3689	31.5664
5	6	-0.922587	-0.805489	-0.000430	18.7466	119.5649
6	7	2.314974	1.426296	-0.000015	148.2806	83.2450
7	1	6.150333	0.079126	0.000604	28.7163	12.4966
8	1	5.115976	1.201027	-0.909293	28.8974	10.8824
9	1	5.115499	1.201047	0.909949	28.8976	10.8813
10	1	1.371030	1.807994	-0.000102	23.8556	13.3007
11	1	3.112321	2.045720	0.000009	26.1142	8.4423
12	1	1.417169	-1.660323	-0.000165	24.3141	4.9625
13	7	1.333038	-0.641951	-0.000181	75.5796	79.7632
14	6	-2.292252	-0.323518	-0.000181	47.1573	181.1152
15	6	-3.333256	-1.270394	0.000120	37.1178	186.0298
16	6	-2.607952	1.049532	-0.000228	46.9699	192.1968
17	6	-4.661185	-0.855579	0.000345	42.2403	197.2829
18	1	-3.098138	-2.334202	0.000167	24.0474	6.6448
19	6	-3.933796	1.457255	0.000003	42.5660	197.0679

20	1	-1.806855	1.784451	-0.000451	23.3686	8.2575
21	6	-4.962022	0.506772	0.000289	33.5800	208.5378
22	1	-5.461353	-1.592779	0.000568	23.7541	4.8493
23	1	-4.175901	2.518223	-0.000038	23.7743	5.5136
24	1	-6.000584	0.832954	0.000471	23.5935	4.9096
25	1	-0.794541	-1.898563	-0.000144	24.0696	7.5655

1,2,4-Triazolinium Products:

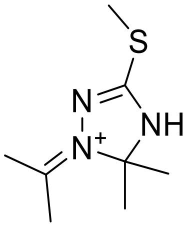

2a

2a E(RB3LYP) = -876.166082348 A.U. after 7 cycles Number of imaginary frequencies: 0

Center Number	Atomic Number	Coordinates (Angstroms) X	Y	Z	NMR shielding tensors isotropic	anisotropic
1	16	2.763071	-1.037443	0.001456	478.2051	279.4851
2	7	0.122437	-1.125698	0.000469	-9.5600	183.1785
3	7	-0.985500	-0.297919	0.000087	-28.9379	159.4418
4	7	0.867946	0.974694	-0.001837	129.1267	89.8627
5	1	1.525193	1.740801	0.004361	26.9421	7.4705
6	6	1.168651	-0.340491	0.000142	5.4982	125.4219
7	6	-1.016435	1.887581	1.285791	152.3962	37.4654
8	1	-2.095504	2.058126	1.323206	29.4492	7.6373
9	1	-0.713755	1.311762	2.166658	29.8380	7.7675
10	1	-0.529412	2.868943	1.331481	29.8087	8.9233
11	6	3.827864	0.445159	-0.002850	162.7622	31.0323
12	1	4.849439	0.053145	-0.003865	29.0333	12.7027
13	1	3.686953	1.041135	-0.910280	29.2431	9.9726
14	1	3.690159	1.044319	0.903001	29.2468	9.9791
15	6	-2.176878	-0.831981	-0.000380	2.0011	190.3357
16	6	-2.269388	-2.321719	-0.000988	151.2045	30.4949
17	1	-1.749468	-2.735873	0.873134	28.7770	8.0620
18	1	-1.748119	-2.735215	-0.874626	28.7773	8.0573
19	1	-3.311933	-2.648545	-0.001841	29.4207	7.6992
20	6	-3.445569	-0.049512	-0.000229	156.1799	29.4635
21	1	-4.039608	-0.340839	-0.878232	28.8820	8.4880
22	1	-3.333454	1.032475	0.001488	29.0274	6.4282
23	1	-4.040854	-0.343456	0.876014	28.8833	8.4893
24	6	-1.019359	1.889140	-1.283569	152.3693	37.4422
25	1	-0.530545	2.869576	-1.329978	29.8052	8.9228
26	1	-2.098185	2.061811	-1.317596	29.4482	7.6317
27	1	-0.720052	1.313599	-2.165744	29.8355	7.7683
28	6	-0.582915	1.180120	0.000164	86.1643	42.6674

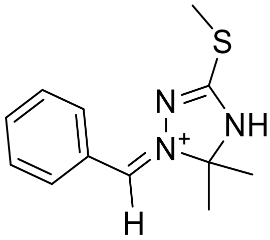

2b-Z

2b-Z E(RB3LYP) = -1028.50300754 A.U. after 6 cycles Number of imaginary frequencies: 0

Center Number	Atomic Number	Coordinates (Angstroms) X	Y	Z
1	16	2.848944	-2.014406	0.002330
2	7	0.595785	-0.631851	0.001141
3	7	0.138578	0.658853	0.000323
4	7	2.377959	0.710948	-0.003588
5	1	3.348408	0.987977	0.006219

6	6	1.905051	-0.554000	0.000081
7	6	1.258436	2.510480	1.278535
8	1	0.388430	3.176611	1.299891
9	1	1.239717	1.869997	2.166388
10	1	2.154744	3.140729	1.317805
11	6	4.555483	-1.367275	-0.005388
12	1	5.190724	-2.258161	-0.007459
13	1	4.764592	-0.792431	-0.913295
14	1	4.772709	-0.791228	0.899893
15	6	1.275100	1.672655	0.000218
16	6	1.254601	2.515634	-1.274583
17	1	2.151524	3.144961	-1.314289
18	1	1.232583	1.878624	-2.164809
19	1	0.385307	3.182825	-1.290428
20	6	-2.300289	0.171512	-0.000194
21	6	-2.303605	-1.244260	0.000395
22	6	-3.538837	0.860772	-0.000883
23	6	-3.510886	-1.929525	0.000240
24	1	-1.362712	-1.781243	0.000973
25	6	-4.736672	0.163572	-0.001076
26	1	-3.550261	1.949992	-0.001280
27	6	-4.724072	-1.234387	-0.000514
28	1	-3.509756	-3.017642	0.000727
29	1	-5.681219	0.703324	-0.001662
30	1	-5.663700	-1.784229	-0.000648
31	6	-1.124804	0.998230	-0.000135
32	1	-1.287482	2.074298	-0.000600

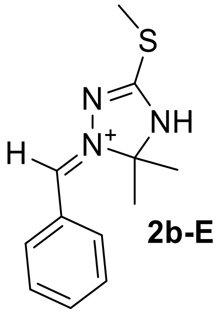

2b-E

2b-E E(RB3LYP) = -1028.49184879 A.U. after 7 cycles Number of imaginary frequencies: 0

Center Number	Atomic Number	Coordinates (Angstroms) X	Y	Z
1	16	4.197551	-0.294584	0.042187
2	7	1.545709	-0.819204	-0.030866
3	7	0.348080	-0.111075	-0.010100
4	7	2.057885	1.343080	0.144305
5	1	2.636233	2.166481	0.043254
6	6	2.499735	0.069481	0.045257
7	6	0.007687	2.028048	1.296663
8	1	-1.083525	2.030510	1.302777
9	1	0.365661	1.501689	2.187150
10	1	0.345196	3.069365	1.355307
11	6	4.161614	-2.112462	-0.124013
12	1	5.213326	-2.412530	-0.139375
13	1	3.654753	-2.562713	0.732268
14	1	3.674482	-2.401159	-1.057987
15	6	0.598337	1.393437	0.036087
16	6	0.196952	2.062211	-1.282769
17	1	0.515904	3.111012	-1.261389
18	1	0.689601	1.566452	-2.125870
19	1	-0.881030	2.046790	-1.454846
20	6	-2.170899	-0.558671	-0.021615
21	6	-2.963843	-1.730139	0.078540
22	6	-2.841101	0.682393	-0.124242
23	6	-4.350466	-1.665537	0.092018
24	1	-2.475423	-2.700322	0.149526
25	6	-4.226949	0.740205	-0.116157
26	1	-2.294411	1.610705	-0.220673
27	6	-4.987458	-0.428706	-0.004667
28	1	-4.934125	-2.579906	0.174255
29	1	-4.723694	1.704616	-0.200107
30	1	-6.074364	-0.371412	0.001713

31	6	-0.749985	-0.825516	-0.019119
32	1	-0.492861	-1.884310	-0.020268

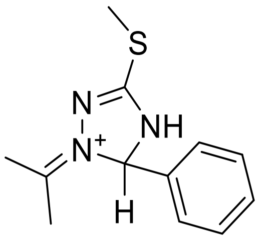

2b'

2b' E(RB3LYP) = -1028.50171160 A.U. after 6 cycles Number of imaginary frequencies: 0

Center Number	Atomic Number	Coordinates (Angstroms) X	Y	Z
1	16	3.093148	-1.711414	-0.232926
2	7	1.690750	0.576902	0.121700
3	7	0.463772	1.089103	-0.265169
4	7	0.677798	-0.956350	-1.156696
5	1	0.448130	-1.903583	-1.429985
6	6	1.758904	-0.618156	-0.411364
7	6	4.198057	-0.740809	0.848584
8	1	5.068755	-1.383541	1.007341
9	1	4.499973	0.183754	0.351598
10	1	3.710260	-0.527312	1.802223
11	6	0.123031	2.312517	0.021814
12	6	1.082092	3.150784	0.796543
13	1	2.024913	3.255595	0.242101
14	1	1.343980	2.658477	1.741973
15	1	0.663673	4.139887	0.996986
16	6	-1.181915	2.886573	-0.406163
17	1	-1.790907	3.099651	0.485027
18	1	-1.766788	2.248054	-1.069137
19	1	-1.002612	3.854026	-0.894346
20	6	-1.608397	-0.387967	-0.333605
21	6	-2.795044	-0.501724	-1.065703
22	6	-1.588193	-0.751088	1.020185
23	6	-3.955393	-0.972450	-0.450268
24	1	-2.817231	-0.226372	-2.120189
25	6	-2.749473	-1.214889	1.632379
26	1	-0.669814	-0.670268	1.601088
27	6	-3.932939	-1.326983	0.897749
28	1	-4.875429	-1.060753	-1.024646
29	1	-2.732982	-1.492511	2.684540
30	1	-4.838207	-1.691979	1.379265
31	6	-0.349029	0.070338	-1.034979
32	1	-0.592545	0.495039	-2.016889

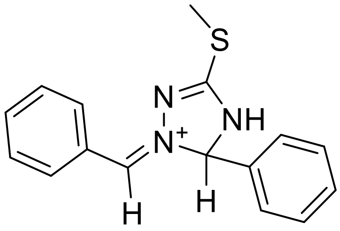

2c-Z

2c-Z E(RB3LYP) = -1180.89584270 A.U. after 1 cycles Number of imaginary frequencies: 0

Center Number	Atomic Number	Coordinates (Angstroms) X	Y	Z	NMR shielding tensors isotropic	anisotropic
1	16	0.112429	3.949354	0.072620	484.3031	313.5202
2	7	-0.632667	1.361550	-0.194117	0.9041	217.2319
3	7	-0.053331	0.173335	-0.561829	-17.1091	154.1627
4	7	1.513481	1.769619	-0.669582	135.7976	105.7544

5	1	2.329099	2.317797	-0.911271	25.8833	8.9577
6	6	0.319669	2.263203	-0.285023	0.4176	127.8945
7	6	-1.644276	3.986984	0.562682	159.4826	29.2454
8	1	-1.846977	5.036523	0.792865	29.1499	12.5201
9	1	-2.277428	3.654790	-0.262804	28.5438	9.8842
10	1	-1.806219	3.370032	1.449200	28.5238	9.6942
11	6	2.368477	-0.554558	-0.301310	39.7811	178.0603
12	6	3.043163	-1.526705	-1.046928	40.9932	181.6563
13	6	2.607154	-0.442566	1.075375	44.8806	197.1964
14	6	3.945201	-2.388754	-0.421025	43.7882	194.2495
15	1	2.868700	-1.607304	-2.118913	23.8263	7.4842
16	6	3.507323	-1.303898	1.696423	42.4045	197.3708
17	1	2.095281	0.319277	1.660820	23.8917	10.0600
18	6	4.176378	-2.278232	0.949387	39.8311	201.2914
19	1	4.468569	-3.142195	-1.006560	23.8323	4.5629
20	1	3.691253	-1.214536	2.765449	23.8270	5.8256
21	1	4.881194	-2.948545	1.438355	23.7711	4.1443
22	6	1.391495	0.362237	-0.995506	88.3562	23.4012
23	1	1.430618	0.200936	-2.081076	24.9605	6.9934
24	6	-2.005186	-1.323289	-0.226540	47.8441	184.0360
25	6	-2.985411	-0.406559	0.217894	39.3576	204.1534
26	6	-2.351864	-2.689207	-0.353472	34.5294	187.0192
27	6	-4.264517	-0.856221	0.522670	43.5048	195.5870
28	1	-2.729457	0.641367	0.317527	22.0666	11.0028
29	6	-3.633330	-3.125481	-0.046858	43.8185	194.2776
30	1	-1.604179	-3.402482	-0.695916	24.0058	8.4658
31	6	-4.592556	-2.208986	0.392492	34.5008	208.0967
32	1	-5.014941	-0.146339	0.865060	23.7519	6.0769
33	1	-3.887233	-4.178492	-0.149029	23.8656	5.2979
34	1	-5.598104	-2.549350	0.633834	23.6443	4.7069
35	6	-0.646836	-0.986359	-0.572049	28.0020	141.4843
36	1	-0.000708	-1.802319	-0.890041	24.3460	12.1891

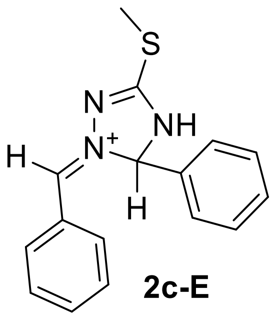

2c-E

2c-E E(RB3LYP) = -1180.83006480 A.U. after 7 cycles Number of imaginary frequencies: 0

Center Number	Atomic Number	Coordinates (Angstroms) X	Y	Z	NMR shielding tensors isotropic	anisotropic
1	16	1.384087	3.559374	0.397322	475.6306	276.6744
2	7	-0.222349	1.510170	-0.067502	-3.9491	225.0190
3	7	-0.191516	0.218041	-0.522769	-10.6038	134.2596
4	7	1.879440	1.064971	-0.688486	137.8051	97.7050
5	1	2.886860	1.128178	-0.651606	26.6933	6.3806
6	6	1.007652	1.954797	-0.154979	1.5661	120.3995
7	6	3.143104	3.720240	-0.064699	162.1971	30.9424
8	1	3.407268	4.748220	0.201032	28.9914	12.8137
9	1	3.778727	3.041737	0.513663	29.1746	9.6822
10	1	3.286110	3.589472	-1.141627	29.0347	10.1221
11	6	1.749793	-1.412412	-0.315933	42.7638	173.1927
12	6	2.101218	-2.522741	-1.091472	42.7168	176.2442
13	6	1.922114	-1.451877	1.075621	47.4243	192.0697
14	6	2.620013	-3.666384	-0.482762	42.0351	197.7855
15	1	1.977357	-2.494614	-2.173865	24.0357	7.3854
16	6	2.438508	-2.594402	1.679000	40.4061	201.6267
17	1	1.649910	-0.594353	1.690264	24.1889	9.8309
18	6	2.788130	-3.701734	0.900323	37.0294	206.8631
19	1	2.894769	-4.526410	-1.090225	23.7256	4.7031
20	1	2.569942	-2.624566	2.758901	23.7185	6.0565
21	1	3.193473	-4.592863	1.376041	23.5869	4.2683
22	6	1.194999	-0.184274	-0.991547	90.0212	19.4530
23	1	1.143481	-0.345688	-2.077048	25.3759	6.7398
24	6	-2.589951	-0.354618	-0.222801	49.6904	182.0272

25	6	-3.105453	0.865711	0.276864	38.3425	206.0991
26	6	-3.469896	-1.451229	-0.400436	34.8077	183.2712
27	6	-4.455578	0.968324	0.582160	42.0358	198.6033
28	1	-2.440683	1.709975	0.416476	22.0502	11.5828
29	6	-4.815841	-1.334724	-0.090299	42.8678	196.1407
30	1	-3.084353	-2.394783	-0.784247	24.2445	8.5112
31	6	-5.310715	-0.123393	0.401667	30.7373	213.9180
32	1	-4.849305	1.907641	0.965125	23.6643	5.8861
33	1	-5.482629	-2.182843	-0.230289	23.8122	5.4391
34	1	-6.367647	-0.029538	0.645295	23.4864	4.8088
35	6	-1.218609	-0.588339	-0.579102	28.2427	138.1116
36	1	-0.964636	-1.579496	-0.953374	24.6644	12.1737

Carbinolamine intermediates:

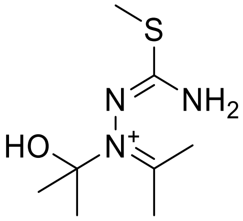

2a-ii

2a-ii E(RB3LYP) = -952.537542851 A.U. after 6 cycles Number of imaginary frequencies: 0

Center Number	Atomic Number	Coordinates (Angstroms) X	Y	Z
1	7	-0.928356	0.269501	-0.137580
2	7	0.339373	0.238175	-0.696015
3	6	1.367335	0.086941	0.090542
4	16	2.896379	0.032847	-0.794546
5	6	4.140192	-0.210453	0.516606
6	6	-1.600387	-1.188883	-0.071279
7	6	-1.506379	1.428639	0.006748
8	6	-2.951409	1.634282	0.324184
9	6	-0.684046	2.660318	-0.211035
10	8	-0.554358	-2.082271	-0.080167
11	6	-2.306403	-1.401655	1.262762
12	6	-2.479613	-1.330402	-1.309302
13	7	1.346375	0.027583	1.435058
14	1	5.095254	-0.256055	-0.016291
15	1	4.178031	0.636789	1.209549
16	1	4.000665	-1.159923	1.044667
17	1	-3.574364	0.744818	0.293021
18	1	-3.041359	2.098201	1.317305
19	1	-3.352557	2.368494	-0.387292
20	1	-0.252813	2.634113	-1.222108
21	1	-1.284435	3.564494	-0.085727
22	1	0.165655	2.696876	0.482061
23	1	-0.160854	-2.132715	-0.962710
24	1	-2.645943	-2.442412	1.285636
25	1	-3.171955	-0.759530	1.422189
26	1	-1.604606	-1.273580	2.094254
27	1	-3.325497	-0.637489	-1.317115
28	1	-1.886367	-1.158818	-2.216117
29	1	-2.874264	-2.352182	-1.338536
30	1	2.183274	-0.168460	1.964818
31	1	0.467442	-0.089540	1.920872

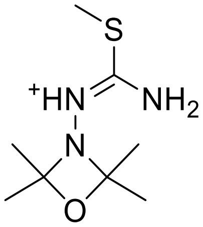

2a-v

2a-v E(RB3LYP) = -952.533904289 A.U. after 6 cycles Number of imaginary frequencies: 0

Center Number	Atomic Number	Coordinates (Angstroms) X	Y	Z	NMR shielding tensors isotropic	anisotropic
1	6	1.805465	1.044808	-0.020729	72.0403	52.3599
2	7	0.747326	0.000001	0.258075	133.2852	83.1694
3	6	1.805545	-1.044760	-0.020546	72.0418	52.3589
4	8	2.784309	0.000052	-0.120860	228.1760	134.5775
5	6	-1.600439	-0.000028	0.188151	-2.5123	174.6266
6	7	-1.602503	0.000222	1.507315	145.0274	88.6774
7	16	-3.034777	-0.000222	-0.807939	455.3392	354.4112
8	6	-4.386150	0.000148	0.417128	162.3102	32.2176
9	1	-2.450598	0.000156	2.055570	26.1016	8.8101
10	1	-4.373998	0.909892	1.025597	29.0017	10.9367
11	1	-4.373900	-0.909151	1.026260	29.0021	10.9356
12	1	-5.297698	-0.000120	-0.188761	28.7739	13.0063
13	6	1.649014	-1.814585	-1.322668	156.9865	31.9283
14	1	2.578291	-2.363606	-1.510819	29.5687	8.9507
15	1	0.833611	-2.545999	-1.266322	30.4221	7.9065
16	1	1.490003	-1.154369	-2.182195	30.0661	6.3789
17	6	2.062238	-1.952220	1.167036	148.4118	44.9207
18	1	2.177561	-1.363775	2.082372	29.7777	6.4942
19	1	1.241313	-2.668649	1.296983	30.2191	7.1535
20	1	2.986129	-2.519656	1.005567	29.6811	9.3220
21	6	2.062127	1.952484	1.166694	148.4121	44.9211
22	1	2.986105	2.519767	1.005177	29.6810	9.3219
23	1	1.241283	2.669045	1.296423	30.2190	7.1539
24	1	2.177311	1.364221	2.082164	29.7778	6.4939
25	6	1.648834	1.814394	-1.322977	156.9857	31.9292
26	1	0.833249	2.545616	-1.266777	30.4221	7.9056
27	1	2.577979	2.363622	-1.511177	29.5687	8.9507
28	1	1.490014	1.153997	-2.182399	30.0661	6.3792
29	1	-0.687187	0.000256	1.958475	23.7065	10.2516
30	7	-0.430175	-0.000118	-0.460333	108.9387	92.5332
31	1	-0.429858	-0.000299	-1.480156	24.9358	6.6246

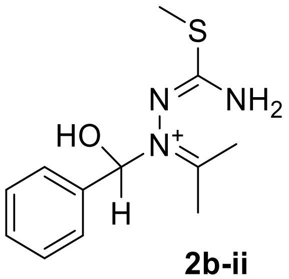

2b-ii

2b-ii E(RB3LYP) = -1104.87326062 A.U. after 7 cycles Number of imaginary frequencies: 0

Center Number	Atomic Number	Coordinates (Angstroms) X	Y	Z
1	7	-0.137056	0.899329	-0.103982
2	7	-1.056220	0.032030	0.467987
3	6	-2.198560	-0.165164	-0.132416
4	16	-3.346198	-1.039281	0.887459
5	6	-4.668195	-1.526089	-0.272569
6	6	0.857824	0.265167	-1.086441
7	6	-0.024888	2.108670	0.366832
8	6	1.076529	3.036567	-0.021826
9	6	-1.009993	2.577273	1.385058
10	8	0.159377	-0.643642	-1.865001
11	7	-2.548101	0.275034	-1.357831
12	1	-5.332097	-2.165110	0.318364
13	1	-5.251735	-0.667042	-0.621334
14	1	-4.278661	-2.113529	-1.109942
15	1	1.726458	2.691404	-0.824912
16	1	0.648680	4.010823	-0.291295
17	1	1.702178	3.210547	0.866727
18	1	-1.106258	1.828319	2.182286
19	1	-0.715469	3.543148	1.802656
20	1	-2.007547	2.678722	0.935365
21	1	0.263943	-1.533708	-1.500258

22	1	-3.472191	0.087801	-1.720604
23	1	-1.809408	0.379262	-2.046104
24	6	2.058345	-0.308382	-0.370610
25	6	3.321498	-0.144009	-0.953901
26	6	1.929848	-1.058015	0.808025
27	6	4.445664	-0.722429	-0.367699
28	1	3.428826	0.430282	-1.873892
29	6	3.058705	-1.633007	1.389994
30	1	0.953661	-1.185395	1.272802
31	6	4.314776	-1.467171	0.804703
32	1	5.423637	-0.591093	-0.826687
33	1	2.956534	-2.211229	2.306404
34	1	5.192878	-1.917564	1.263845
35	1	1.161057	1.076792	-1.750872

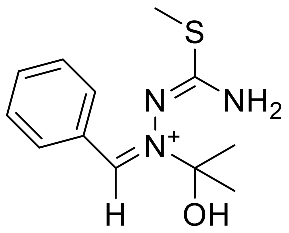

2b-ii'

2b-ii' E(RB3LYP) = -1104.88060704 A.U. after 1 cycles Number of imaginary frequencies: 0

Center Number	Atomic Number	Coordinates (Angstroms) X	Y	Z	NMR shielding tensors isotropic	anisotropic
1	7	0.546461	-0.942906	-0.033816	-26.2265	183.9514
2	7	0.994232	0.330999	-0.324493	-8.1106	141.3094
3	6	1.118327	1.172330	0.660357	-1.3258	152.8246
4	16	1.748420	2.788481	0.335405	449.1339	345.6473
5	6	2.270663	2.659317	-1.404190	161.2537	31.4455
6	6	1.652374	-2.054418	-0.148886	82.0344	25.0536
7	6	-0.706995	-1.294356	0.026347	-8.2416	203.4030
8	8	1.094922	-3.247952	0.283974	155.2305	31.1484
9	7	0.788100	0.959140	1.951463	153.5914	30.8033
10	1	2.661907	3.651318	-1.650386	232.1826	88.5578
11	1	3.059263	1.911961	-1.517995	162.3391	70.2646
12	1	1.422807	2.421428	-2.049722	29.1236	12.6759
13	1	1.288455	-3.397212	1.218996	29.2248	10.7058
14	1	0.922693	1.684057	2.643833	29.1974	9.9642
15	1	0.270206	0.138880	2.232865	29.1265	6.9757
16	1	-0.827181	-2.370159	0.149084	29.0405	9.1022
17	6	-1.918610	-0.511597	-0.053038	29.2966	7.3279
18	6	-3.114766	-1.225921	0.199459	28.6774	6.0236
19	6	-2.004765	0.857226	-0.399921	29.4963	8.3595
20	6	-4.348770	-0.595451	0.129406	29.4592	7.3181
21	1	-3.064763	-2.284857	0.448647	28.8990	11.5796
22	6	-3.245685	1.474172	-0.482668	27.0349	10.1809
23	1	-1.110641	1.424382	-0.628196	26.0464	11.5217
24	6	-4.415504	0.757091	-0.212263	40.7363	178.9216
25	1	-5.258962	-1.156209	0.330932	44.9869	175.5492
26	1	-3.305895	2.524399	-0.760860	49.5225	188.8570
27	1	-5.382103	1.254163	-0.274155	42.1455	197.5025
28	6	1.977080	-2.185205	-1.631696	24.1148	7.8579
29	1	1.103998	-2.563602	-2.174185	40.9456	200.5644
30	1	2.277366	-1.223018	-2.054390	23.5000	10.2793
31	1	2.792509	-2.906120	-1.751353	38.2693	205.1134
32	6	2.845659	-1.639860	0.701685	23.8055	4.9716
33	1	3.600196	-2.431618	0.640481	23.8101	6.2094
34	1	3.299748	-0.712603	0.345110	23.6864	4.4050
35	1	2.560715	-1.507855	1.753388	25.1905	4.0572

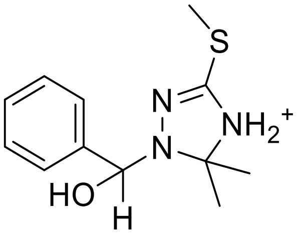

2b-iii

2b-iii E(RB3LYP) = -1104.84245479 A.U. after 1 cycles Number of imaginary frequencies: 0

Center Number	Atomic Number	Coordinates (Angstroms) X	Y	Z	NMR shielding tensors isotropic	anisotropic
1	16	2.884676	2.146296	-0.300716	-26.1027	189.0242
2	7	0.709023	0.490836	0.103662	13.2242	122.5205
3	7	0.355641	-0.808658	-0.120622	2.7380	149.6478
4	6	1.911681	0.702233	-0.228116	458.3370	365.4661
5	6	1.431914	-2.781459	-1.222131	161.9913	30.2020
6	1	0.712709	-3.518780	-0.851015	74.3945	42.1355
7	1	1.065758	-2.409665	-2.186065	16.2951	153.4403
8	1	2.384076	-3.306696	-1.362509	237.8216	69.0503
9	6	1.642861	3.368943	0.247232	157.5945	61.5142
10	1	2.162621	4.330884	0.225719	29.2592	12.7818
11	1	0.794583	3.378654	-0.441635	29.1220	10.2642
12	1	1.313765	3.139775	1.263733	29.0831	10.6524
13	6	1.568423	-1.655986	-0.209780	28.3418	14.0481
14	6	2.115381	-2.116705	1.141788	26.5326	9.9389
15	1	3.082239	-2.622045	1.016117	26.3600	9.8073
16	1	2.218512	-1.281137	1.841975	23.2943	11.9448
17	1	1.412269	-2.821398	1.591963	48.5698	182.1734
18	6	-2.060114	-0.433581	0.163492	31.9938	190.4069
19	6	-2.431248	0.651510	0.964340	40.9074	208.3399
20	6	-2.805202	-0.731100	-0.982270	43.3276	195.2515
21	6	-3.537127	1.428376	0.620316	23.6662	8.0553
22	1	-1.853795	0.885180	1.855985	43.0771	196.2484
23	6	-3.907505	0.048447	-1.327573	22.7033	10.5755
24	1	-2.530996	-1.582703	-1.605081	33.0493	210.5020
25	6	-4.274689	1.130118	-0.525660	23.7197	4.8842
26	1	-3.824693	2.268492	1.250098	23.7386	6.5503
27	1	-4.486873	-0.194059	-2.216543	23.5410	4.8296
28	1	-5.140186	1.735030	-0.790095	153.8739	34.0987
29	6	-0.859569	-1.290810	0.524169	29.6340	7.6459
30	1	-1.016561	-2.296149	0.101166	29.4819	8.3314
31	1	3.491608	-0.720614	-0.419844	29.5243	9.1478
32	1	2.548448	-0.555500	-1.777073	148.8553	47.7251
33	7	2.530386	-0.572142	-0.747204	29.8401	9.2182
34	8	-0.620525	-1.368425	1.912157	29.9380	7.8812
35	1	-1.458277	-1.512770	2.371609	29.4122	6.0712

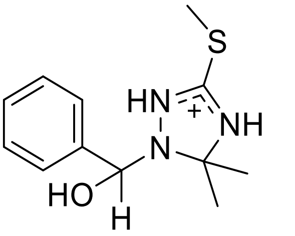

2b-iv

2b-iv E(RB3LYP) = -1104.84245479 A.U. after 1 cycles Number of imaginary frequencies: 0

Center Number	Atomic Number	Coordinates (Angstroms) X	Y	Z
1	16	2.884676	2.146296	-0.300716
2	7	0.709023	0.490836	0.103662
3	7	0.355641	-0.808658	-0.120622
4	6	1.911681	0.702233	-0.228116

5	6	1.431914	-2.781459	-1.222131
6	1	0.712709	-3.518780	-0.851015
7	1	1.065758	-2.409665	-2.186065
8	1	2.384076	-3.306696	-1.362509
9	6	1.642861	3.368943	0.247232
10	1	2.162621	4.330884	0.225719
11	1	0.794583	3.378654	-0.441635
12	1	1.313765	3.139775	1.263733
13	6	1.568423	-1.655986	-0.209780
14	6	2.115381	-2.116705	1.141788
15	1	3.082239	-2.622045	1.016117
16	1	2.218512	-1.281137	1.841975
17	1	1.412269	-2.821398	1.591963
18	6	-2.060114	-0.433581	0.163492
19	6	-2.431248	0.651510	0.964340
20	6	-2.805202	-0.731100	-0.982270
21	6	-3.537127	1.428376	0.620316
22	1	-1.853795	0.885180	1.855985
23	6	-3.907505	0.048447	-1.327573
24	1	-2.530996	-1.582703	-1.605081
25	6	-4.274689	1.130118	-0.525660
26	1	-3.824693	2.268492	1.250098
27	1	-4.486873	-0.194059	-2.216543
28	1	-5.140186	1.735030	-0.790095
29	6	-0.859569	-1.290810	0.524169
30	1	-1.016561	-2.296149	0.101166
31	1	3.491608	-0.720614	-0.419844
32	1	2.548448	-0.555500	-1.777073
33	7	2.530386	-0.572142	-0.747204
34	8	-0.620525	-1.368425	1.912157
35	1	-1.458277	-1.512770	2.371609

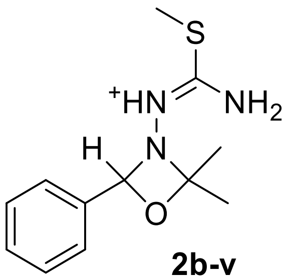

2b-v

2b-v E(RB3LYP) = -1104.86024302 A.U. after 6 cycles Number of imaginary frequencies: 0

Center Number	Atomic Number	Coordinates (Angstroms) X	Y	Z	NMR shielding tensors isotropic	anisotropic
1	6	-1.047790	0.828658	-0.796633	73.9249	31.9219
2	7	0.207793	0.922846	0.023903	125.0324	87.1310
3	8	-1.379469	2.151677	-0.396965	238.8691	125.5084
4	6	2.056690	-0.514849	0.177318	-2.0289	175.0308
5	16	3.546399	-1.067200	-0.546919	456.4087	354.1998
6	6	4.094913	-2.380698	0.593698	162.3834	32.0848
7	1	3.368393	-3.197934	0.639829	29.1870	10.4554
8	1	4.332372	-1.977723	1.583297	29.1223	11.0684
9	1	5.017803	-2.759411	0.143292	28.8731	13.2177
10	6	-2.029005	-0.240198	-0.427194	40.0510	185.2743
11	6	-2.182287	-1.358385	-1.254700	46.4043	175.6338
12	6	-2.783562	-0.141600	0.749360	46.5280	196.9289
13	6	-3.063267	-2.380333	-0.900898	43.8266	194.4549
14	1	-1.623469	-1.425990	-2.188296	24.4360	7.7751
15	6	-3.669020	-1.158643	1.096737	42.1272	197.9399
16	1	-2.695874	0.748299	1.368973	23.4323	9.7274
17	6	-3.805631	-2.281071	0.275570	39.0225	201.2873
18	1	-3.182725	-3.244847	-1.551330	23.9566	5.0625
19	1	-4.265366	-1.072528	2.003375	23.7218	5.6123
20	1	-4.503433	-3.071431	0.546706	23.7555	4.3224
21	1	-0.803537	0.765734	-1.873862	25.8688	5.4111
22	6	-0.048969	2.411391	0.088587	70.5218	51.8486
23	6	-0.046945	2.975991	1.491078	149.9735	44.2560
24	1	0.977230	3.053184	1.877042	30.1204	7.3503
25	1	-0.483645	3.981530	1.486424	29.5685	9.4247
26	1	-0.637671	2.343408	2.160369	29.6529	6.9322

27	6	0.755925	3.242104	-0.899947	161.3097	27.3717
28	1	0.329186	4.250290	-0.937166	29.5164	9.2381
29	1	1.805425	3.334442	-0.594274	30.3361	7.9867
30	1	0.705187	2.835977	-1.917869	29.7347	7.6358
31	7	1.565382	-0.959189	1.317172	144.0990	90.2595
32	1	0.672951	-0.560827	1.612584	24.0666	11.1584
33	1	2.026164	-1.663316	1.875494	26.4502	8.5614
34	7	1.388278	0.431491	-0.493275	105.4780	91.7402
35	1	1.737882	0.742691	-1.400620	25.2087	7.1578

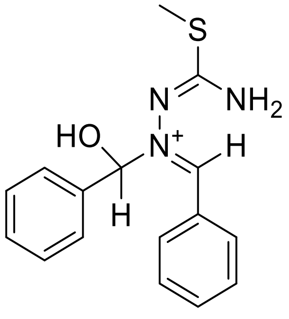

2c-ii

2c-ii E(RB3LYP) = -1257.19437375 A.U. after 11 cycles Number of imaginary frequencies: 0

Center Number	Atomic Number	Coordinates (Angstroms) X	Y	Z	NMR shielding tensors isotropic	anisotropic
1	7	0.160460	-0.338805	-0.216557	-16.1656	205.7780
2	7	-1.048421	-0.850628	-0.672988	-13.7405	152.0824
3	6	-1.574144	-1.828138	0.014486	0.2047	150.4954
4	16	-2.952387	-2.570837	-0.804502	447.8528	346.1499
5	6	-3.676102	-3.650206	0.474869	161.6609	30.6689
6	6	0.107288	0.862604	0.688885	85.4114	39.1160
7	6	1.266538	-0.840992	-0.696219	4.4623	150.2495
8	8	-0.172774	0.415009	1.984635	247.3388	57.8994
9	7	-1.138209	-2.298557	1.199558	166.3577	69.5049
10	1	-4.585572	-4.052089	0.016830	29.2699	12.8133
11	1	-3.025095	-4.496080	0.722754	29.3874	10.5847
12	1	-3.961746	-3.086958	1.368949	29.3860	9.8770
13	1	-1.089483	0.636051	2.205429	28.4558	13.5123
14	1	-1.588740	-3.080465	1.651339	27.1993	10.3619
15	1	-0.498692	-1.737189	1.753986	25.3974	13.5262
16	6	-0.839720	1.937742	0.186445	42.6747	166.5899
17	6	-1.136510	2.107519	-1.171144	46.0813	189.2738
18	6	-1.337197	2.860139	1.117230	52.5214	183.7005
19	6	-1.938438	3.168748	-1.583552	42.6712	197.4526
20	1	-0.766796	1.403661	-1.911347	24.0232	9.7053
21	6	-2.144907	3.918169	0.699469	42.5460	197.9660
22	1	-1.078820	2.778184	2.172026	24.1958	10.0234
23	6	-2.448266	4.073435	-0.651054	40.0042	202.1146
24	1	-2.171286	3.285601	-2.640193	23.8847	5.7732
25	1	-2.525976	4.625940	1.433062	23.8251	5.3068
26	1	-3.076633	4.899439	-0.978689	23.7846	4.1647
27	1	1.127103	1.254574	0.687367	24.4179	8.7927
28	6	2.646776	-0.552825	-0.363413	49.7027	175.0399
29	6	3.596364	-0.796345	-1.380690	34.9829	194.5989
30	6	3.098656	-0.140383	0.911775	43.6620	189.3839
31	6	4.947105	-0.565770	-1.154301	40.8917	201.1996
32	1	3.264453	-1.151155	-2.355232	23.7574	7.9815
33	6	4.453746	0.063188	1.134350	41.7268	199.3266
34	1	2.402792	-0.026759	1.739440	23.3196	9.8705
35	6	5.376668	-0.132727	0.101775	30.9964	215.2810
36	1	5.667753	-0.735916	-1.951323	23.5349	4.9682
37	1	4.797712	0.365095	2.121456	23.6083	6.2400
38	1	6.436673	0.034036	0.284859	23.3837	4.8011
39	1	1.088788	-1.591673	-1.469310	23.2262	8.7460

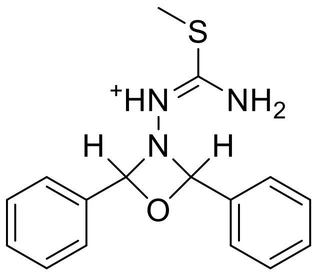

2c-v

2c-v E(RB3LYP) = -1257.18544564 A.U. after 6 cycles Number of imaginary frequencies: 0

Center Number	Atomic Number	Coordinates (Angstroms) X	Y	Z	NMR shielding tensors isotropic	anisotropic
1	6	0.915960	-1.127310	-0.346254	69.0470	35.2876
2	7	0.122175	-0.161849	0.491730	130.6781	99.9541
3	6	-0.656139	-1.366538	0.974880	74.4789	41.3531
4	8	0.323047	-2.208136	0.375597	256.8441	108.6865
5	6	-0.263591	2.131198	0.182201	-0.7382	173.4500
6	7	0.686557	2.371302	1.065723	145.4476	90.9857
7	16	-1.199391	3.363167	-0.628622	456.7787	353.3316
8	6	-0.489939	4.910759	0.025632	162.6202	32.1980
9	1	0.948427	3.304726	1.348064	26.5320	8.9258
10	1	0.566173	5.011472	-0.243682	29.2814	10.8037
11	1	-0.654937	5.003035	1.103745	29.2206	11.0417
12	1	-1.054467	5.700297	-0.480236	29.0039	13.3561
13	1	1.157474	1.557532	1.462868	24.1998	11.0446
14	7	-0.547008	0.865815	-0.140568	102.5750	86.3020
15	1	-1.307903	0.659748	-0.791665	25.7803	9.1553
16	6	2.405908	-1.026508	-0.263300	39.7402	184.5511
17	6	3.139659	-0.606562	-1.378335	45.7812	175.5642
18	6	3.074510	-1.335588	0.929531	46.1025	197.2604
19	6	4.526442	-0.476731	-1.299611	43.8077	194.4139
20	1	2.628718	-0.392645	-2.317110	24.3978	7.9307
21	6	4.459020	-1.211918	1.004211	42.0228	198.1529
22	1	2.508559	-1.700193	1.784700	23.2659	9.6200
23	6	5.185615	-0.778132	-0.108388	38.7465	201.7921
24	1	5.092844	-0.155349	-2.171709	23.9349	5.0875
25	1	4.977666	-1.465800	1.927002	23.6563	5.4789
26	1	6.268929	-0.688757	-0.048653	23.7166	4.2838
27	6	-2.053756	-1.511971	0.433881	45.9204	175.2253
28	6	-3.124996	-0.931374	1.127631	46.1539	184.1138
29	6	-2.298825	-2.210002	-0.755631	47.3021	195.6661
30	6	-4.421799	-1.029185	0.626741	42.5491	197.6283
31	1	-2.948445	-0.418394	2.073371	24.3919	7.8539
32	6	-3.599141	-2.310739	-1.251252	41.7137	199.4442
33	1	-1.474774	-2.706362	-1.263723	23.3586	9.6207
34	6	-4.659225	-1.717326	-0.564903	39.1388	202.1632
35	1	-5.250670	-0.584433	1.174439	23.8018	5.1364
36	1	-3.786634	-2.867883	-2.167351	23.6249	5.3592
37	1	-5.673797	-1.804769	-0.949466	23.6717	4.2274
38	1	0.583280	-1.100435	-1.398706	25.6260	6.4818
39	1	-0.644857	-1.436686	2.070901	24.8692	5.1093

Aziridine intermediates:

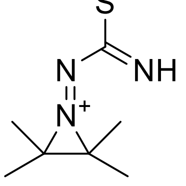

2a-aziridine

2a-aziridine E(RB3LYP) = -876.073967059 A.U. after 8 cycles Number of imaginary frequencies: 0

Center Number	Atomic Number	Coordinates (Angstroms) X	Y	Z	NMR shielding tensors isotropic	anisotropic
1	6	1.685008	0.825005	0.001458	109.4592	73.1859
2	7	0.750739	-0.307423	-0.002185	-164.3208	560.7808
3	6	2.154099	-0.661737	-0.000077	123.1507	75.3411
4	7	-0.365734	-0.805283	-0.002212	-330.2901	774.0274
5	6	-1.504952	0.100764	-0.001612	0.7553	104.8520
6	7	-1.319398	1.349499	-0.001839	-68.9838	477.9884
7	16	-2.923754	-0.934933	-0.000656	304.7647	304.6709
8	6	-4.254395	0.303013	0.000503	159.0382	30.4985
9	1	-2.169451	1.913080	-0.001271	19.9839	13.4330
10	1	-4.215457	0.921236	-0.903031	28.9843	10.3382
11	1	-4.214304	0.920923	0.904200	28.9850	10.3367
12	1	-5.188527	-0.266348	0.001017	28.5303	11.3867
13	6	2.672471	-1.314257	1.271888	158.2551	35.9171
14	1	2.487816	-2.393692	1.235432	29.4861	9.6932
15	1	3.755733	-1.156518	1.330343	29.3748	9.1264
16	1	2.215375	-0.918073	2.180822	30.0720	8.8869
17	6	2.678031	-1.309563	-1.272187	158.2661	35.8708
18	1	3.761444	-1.151126	-1.325602	29.3711	9.1176
19	1	2.493701	-2.389197	-1.240190	29.4865	9.6969
20	1	2.224522	-0.910485	-2.181637	30.0664	8.8857
21	6	1.749108	1.653798	-1.268182	158.6841	35.1723
22	1	0.994128	2.444097	-1.226212	29.3182	8.7934
23	1	2.741893	2.117223	-1.322948	29.5874	9.4230
24	1	1.589543	1.071580	-2.177890	30.3715	8.6736
25	6	1.743728	1.649774	1.273927	158.6862	35.2007
26	1	2.736385	2.112805	1.334343	29.5896	9.4316
27	1	0.989152	2.440421	1.231364	29.3183	8.7823
28	1	1.580288	1.064669	2.181108	30.3784	8.6761

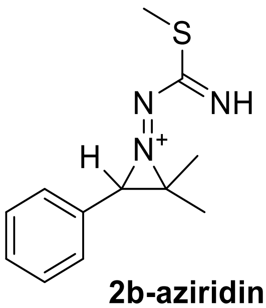

2b-aziridine

2b-aziridine E(RB3LYP) = -1028.40113008 A.U. after 8 cycles Number of imaginary frequencies: 0

Center Number	Atomic Number	Coordinates (Angstroms) X	Y	Z	NMR shielding tensors isotropic	anisotropic
1	6	1.110249	0.933275	-0.778664	119.9613	86.2428
2	7	-0.280720	0.667004	-0.574438	-144.1312	522.3156
3	7	-1.106105	-0.226227	-0.727746	-337.3001	777.3578
4	6	-2.403661	-0.050932	-0.100118	0.5625	105.7572
5	7	-2.670708	1.017694	0.517553	-71.5533	481.1108
6	16	-3.290813	-1.528605	-0.446409	301.5090	305.1107
7	6	-4.864218	-1.191825	0.398474	158.8330	30.5664
8	1	-3.609717	1.066067	0.912651	19.8235	13.4847
9	1	-5.367850	-0.325530	-0.044307	28.8901	10.6222
10	1	-4.711107	-1.052559	1.474239	28.9552	10.0429
11	1	-5.482002	-2.080546	0.239437	28.4732	11.4027
12	6	2.164095	-0.042654	-0.369913	51.0865	165.9037
13	6	3.499214	0.348198	-0.551053	43.8044	181.3679
14	6	1.877092	-1.299188	0.176747	51.6229	187.6774
15	6	4.533049	-0.498452	-0.160468	41.0288	199.4609
16	1	3.731111	1.315901	-0.994140	23.8853	7.9443
17	6	2.917714	-2.144130	0.561086	42.1830	198.4621
18	1	0.849972	-1.641178	0.284370	24.8099	9.4333
19	6	4.244017	-1.744513	0.397795	38.2400	204.5112
20	1	5.566555	-0.187696	-0.300210	23.6633	5.0061
21	1	2.689755	-3.122223	0.979985	23.8824	5.8868
22	1	5.053086	-2.409102	0.694603	23.6672	4.4173
23	1	1.291060	1.424719	-1.740590	26.1777	7.6972
24	6	0.234863	1.818062	0.168006	102.8127	90.3057

25	6	-0.171927	3.183802	-0.343778	152.8516	43.8811
26	1	-1.139377	3.464618	0.084415	29.0896	9.2243
27	1	0.582679	3.913234	-0.026742	29.1122	8.9473
28	1	-0.247544	3.212519	-1.435818	30.1657	9.1372
29	6	0.322990	1.651931	1.665675	162.5655	28.8508
30	1	1.092338	2.341469	2.035158	30.1999	9.9302
31	1	-0.632903	1.910074	2.131393	29.5185	8.6607
32	1	0.608836	0.637166	1.957783	30.7695	7.2897

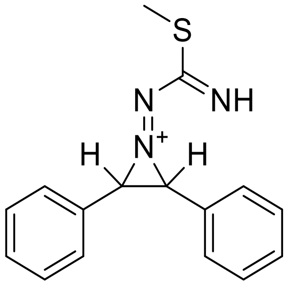

2c-aziridine

2c-aziridine E(RB3LYP) = -1180.72504243 A.U. after 8 cycles Number of imaginary frequencies: 0

Center Number	Atomic Number	Coordinates (Angstroms) X	Y	Z	NMR shielding tensors isotropic	anisotropic
1	6	-0.135156	-0.854344	0.442045	113.8659	84.0455
2	7	-0.174445	0.393335	-0.307446	-131.6795	511.3296
3	6	-1.236636	-0.545424	-0.592669	126.5803	91.3320
4	7	0.277004	1.495749	-0.579256	-312.5675	760.4882
5	6	1.432619	1.928286	0.189174	0.5303	103.6284
6	7	1.696258	1.352656	1.280946	-76.5624	490.4472
7	16	2.146148	3.254096	-0.720158	322.8475	325.9325
8	6	3.624541	3.593141	0.282651	159.5439	30.1487
9	1	2.500336	1.724603	1.786058	20.1294	13.0181
10	1	4.271619	2.711309	0.340926	29.3472	9.6085
11	1	3.345970	3.945416	1.281731	29.0301	10.7067
12	1	4.158168	4.392983	-0.239102	28.7218	11.8316
13	6	-2.644417	-0.194997	-0.238777	47.5081	163.4887
14	6	-2.967061	0.472448	0.951789	51.5271	184.4706
15	6	-3.663005	-0.564062	-1.125447	40.9434	176.5777
16	6	-4.296107	0.758199	1.250249	41.0784	199.7545
17	1	-2.190937	0.782059	1.651133	24.6414	10.0474
18	6	-4.992738	-0.281269	-0.818151	41.5510	197.4129
19	1	-3.420090	-1.079386	-2.053335	23.7813	7.8025
20	6	-5.310181	0.379938	0.367591	37.4594	205.6830
21	1	-4.542179	1.277965	2.174023	23.7792	5.9304
22	1	-5.780560	-0.578348	-1.507460	23.7101	4.9648
23	1	-6.348466	0.603006	0.605527	23.6082	4.4336
24	6	0.975979	-1.823293	0.202813	49.7998	161.4025
25	6	1.515110	-2.497529	1.305028	39.6681	179.9854
26	6	1.470965	-2.087571	-1.082361	52.1474	185.6238
27	6	2.525189	-3.439794	1.121686	41.4661	197.6391
28	1	1.139662	-2.292476	2.305922	23.8550	7.9477
29	6	2.485659	-3.024431	-1.259135	41.5037	199.5023
30	1	1.077960	-1.567666	-1.955663	24.7026	9.8178
31	6	3.011764	-3.703676	-0.158479	38.0185	204.9623
32	1	2.931070	-3.969556	1.981259	23.7524	4.9414
33	1	2.865275	-3.226705	-2.258697	23.8125	5.7501
34	1	3.801306	-4.439166	-0.300446	23.6669	4.2717
35	1	-0.409863	-0.707584	1.488228	26.5432	6.8790
36	1	-1.108506	-1.027917	-1.565327	26.7530	8.3993

Deprotonated structures:

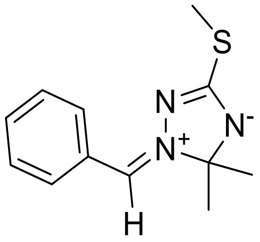

7b

7b E(RB3LYP) = -1028.10139385 A.U. after 7 cycles Number of imaginary frequencies: 0

Center Number	Atomic Number	Coordinates (Angstroms) X	Y	Z
1	16	2.876623	-1.986509	0.000046
2	7	0.592576	-0.620733	0.000270
3	7	0.147051	0.631612	0.000116
4	7	2.467655	0.702008	-0.000090
5	6	1.958892	-0.486112	0.000102
6	6	1.325230	2.454394	1.275410
7	1	0.453699	3.120235	1.300859
8	1	1.306472	1.807232	2.158546
9	1	2.233692	3.064640	1.316254
10	6	4.566399	-1.306277	-0.000191
11	1	5.241513	-2.167612	-0.000271
12	1	4.734262	-0.695322	-0.891063
13	1	4.734511	-0.695292	0.890613
14	6	1.356370	1.610488	-0.000028
15	6	1.324959	2.454387	-1.275416
16	1	2.232624	3.065832	-1.315772
17	1	1.307519	1.807225	-2.158567
18	1	0.452578	3.119090	-1.301376
19	6	-2.299772	0.206601	-0.000045
20	6	-2.302413	-1.205909	-0.000079
21	6	-3.543758	0.879039	-0.000003
22	6	-3.508652	-1.901912	-0.000044
23	1	-1.352674	-1.729023	-0.000110
24	6	-4.739547	0.174312	0.000021
25	1	-3.561105	1.968729	0.000033
26	6	-4.727908	-1.223579	0.000000
27	1	-3.494011	-2.990965	-0.000091
28	1	-5.685680	0.713480	0.000040
29	1	-5.664858	-1.778362	0.000044
30	6	-1.106211	1.024734	-0.000028
31	1	-1.237018	2.103068	-0.000158

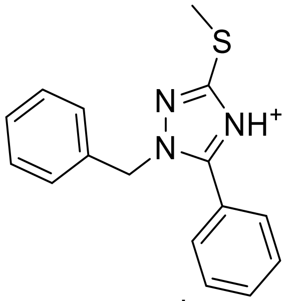

8c + H⁺

8c + H⁺ E(RB3LYP) = -1180.85978128 A.U. after 8 cycles Number of imaginary frequencies: 0

Center Number	Atomic Number	Coordinates (Angstroms) X	Y	Z	NMR shielding tensors isotropic	anisotropic
1	16	1.744856	3.551260	0.570842	496.1157	365.9513
2	7	1.228948	1.133816	-0.625354	-59.8843	306.6217
3	7	0.141127	0.324199	-0.750632	0.8240	167.4730

4	7	-0.510404	2.006835	0.388556	76.0635	82.0576
5	1	-1.106055	2.670683	0.873329	23.5335	5.7608
6	6	0.824775	2.161646	0.074804	15.6276	121.6786
7	6	3.372642	3.115324	-0.134177	159.3338	29.1062
8	1	4.028551	3.944959	0.144445	29.0733	12.7331
9	1	3.738751	2.182960	0.302093	28.7534	10.2291
10	1	3.308429	3.036671	-1.222014	28.6020	10.1979
11	6	-0.932294	0.836880	-0.151869	20.7556	128.8990
12	6	1.211685	-1.912083	-0.576086	44.9047	179.4165
13	6	2.586719	-1.964926	-0.830541	41.8577	178.6776
14	6	0.668212	-2.709379	0.439130	45.2741	173.5713
15	6	3.407830	-2.802337	-0.077044	42.6547	195.5450
16	1	3.015910	-1.353938	-1.623028	23.8276	7.6457
17	6	1.490389	-3.544569	1.193335	43.0351	194.5809
18	1	-0.404015	-2.690634	0.634604	24.8329	8.9756
19	6	2.861288	-3.590572	0.936229	40.0203	202.7135
20	1	4.475193	-2.845096	-0.285837	23.8713	5.1146
21	1	1.060156	-4.167985	1.974946	24.1300	6.1065
22	1	3.502566	-4.248223	1.520283	23.8655	4.2488
23	6	-2.296719	0.314179	-0.054529	57.2251	154.8907
24	6	-2.934245	0.290731	1.198440	45.3493	183.8155
25	6	-2.986466	-0.120273	-1.199508	44.4185	187.1889
26	6	-4.241054	-0.176577	1.301901	40.5060	203.5062
27	1	-2.402121	0.610157	2.093176	24.0391	8.9511
28	6	-4.294156	-0.582490	-1.084135	40.7621	203.2799
29	1	-2.516955	-0.068044	-2.179692	23.8783	8.7522
30	6	-4.920194	-0.613919	0.163367	34.6206	212.1015
31	1	-4.728624	-0.204376	2.274196	23.4927	5.2573
32	1	-4.829884	-0.909830	-1.972791	23.4795	5.5404
33	1	-5.942656	-0.977199	0.247848	23.3202	4.4530
34	6	0.317377	-1.003860	-1.386382	121.5289	30.5556
35	1	0.731642	-0.811029	-2.380665	26.5212	6.4024
36	1	-0.681018	-1.429828	-1.499971	26.1918	7.5969

NMR reference compound:

Tetramethylsilane E(RB3LYP) = -448.973434777 A.U. after 7 cycles Number of imaginary frequencies: 0

Center Number	Atomic Number	Atomic Type	Coordinates (Angstroms) X	Y	Z	NMR shielding tensors isotropic	anisotropic
1	14	0	0.001381	-0.000018	-0.000004	334.9273	2.6397
2	6	0	-0.638918	-1.614023	-0.754667	179.5361	6.7104
3	6	0	-0.634844	1.461981	-1.020945	179.5346	6.7111
4	6	0	-0.635950	0.154580	1.776050	179.5358	6.7108
5	6	0	1.906291	-0.002518	-0.000437	183.4969	12.7593
6	1	0	-0.290363	-2.484742	-0.183119	31.4540	9.5146
7	1	0	-0.289624	-1.734217	-1.789006	31.4538	9.5144
8	1	0	-1.736646	-1.641833	-0.768164	31.5145	9.8303
9	1	0	-0.283862	2.417040	-0.607447	31.4539	9.5138
10	1	0	-0.285446	1.401689	-2.060460	31.4540	9.5145
11	1	0	-1.732497	1.489951	-1.039286	31.5145	9.8303
12	1	0	-0.284939	1.084221	2.243881	31.4541	9.5146
13	1	0	-0.287304	-0.681778	2.396741	31.4538	9.5142
14	1	0	-1.733617	0.158871	1.808486	31.5148	9.8302
15	1	0	2.307219	-0.928550	-0.432097	31.4555	9.5879
16	1	0	2.309424	0.833486	-0.586257	31.4556	9.5872
17	1	0	2.308840	0.085995	1.016778	31.4556	9.5875

7) References

[1] R. Dennington, T. A. Keith, J. M. Millam. "GaussView, version 6.0. 16." *Semichem Inc. Shawnee Mission KS*, **2016**

[2] J. R. Cheeseman, G. W. Trucks, T. A. Keith, M. J. Frisch, *J. Chem. Phys.* **1996**, *104*, 5497–5509.

[3] K. Wolinski, J. F. Hinton, P. Pulay, *J. Am. Chem. Soc.* **1990**, *112*, 8251–8260.

[4] R. Ditchfield, *Mol. Phys.* **1974**, *27*, 789–807.

[5] L. Fliri, G. Partl, K. Wurst, T. Gelbrich, H. Schottenberger, S. Nerdinger, *Heterocycles* **2020**, *101*, 593.

[6] M. B. Foreiter, H. Q. N. Gunaratne, P. Nockemann, K. R. Seddon, P. J. Stevenson, D. F. Wassell, *New J. Chem.* **2013**, *37*, 515–533.

[7] G. A. E. Mostafa, H. A. Ghabbour, H. A. Abdel-Aziz, *Z. Krist.-New Cryst. St.* **2016**, *231*, 631–633.

[8] Y. A. Al-Soud, P. B. Shrestha-Dawadi, M. Winkler, W. Wirschun, J. C. Jochims, *J. Chem. Soc. Perk. T. 1* **1998**, 3759–3766.

[9] C. Yamazaki, M. Ohno, *J. Heterocyclic Chem.* **1997**, *34*, 733–737.

[10] K. N. Zelenin, O. B. Kuznetsova, V. V. Alekseev, V. P. Sergutina, P. V. Terent'ev, V. V. Ovcharenko, *Chem. Heterocycl. Compd.* **1991**, *27*, 1223–1227.
